# Electrochemical synthesis of cyclic biaryl λ^3^-bromanes from 2,2’-dibromobiphenyls

**DOI:** 10.3762/bjoc.21.32

**Published:** 2025-02-27

**Authors:** Andrejs Savkins, Igors Sokolovs

**Affiliations:** 1 Latvian Institute of Organic Synthesis, Aizkraukles 21, 1006 Riga, Latvia; 2 Faculty of Medicine and Life Sciences, University of Latvia, Jelgavas 1, 1004 Riga, Latvia

**Keywords:** anodic oxidation, cyclic biaryl λ^3^-bromane, cyclic voltammetry, electrochemistry, hypervalent bromine

## Abstract

The remarkable nucleofugality of bromoarenes in diarylbromonium species renders them particularly suitable for the generation of arynes for subsequent use in a wide range of synthetic applications. The common approach to generate cyclic biaryl λ^3^-bromanes is based on thermal decomposition of hazardous diazonium salts. Herein, we disclose a mild and straightforward approach to diarylbromonium species by direct anodic oxidation of 2,2'-dibromo-1,1'-biphenyl. The electrochemical method provides access to a range of symmetrically and non-symmetrically substituted cyclic biaryl λ^3^-bromanes in moderate yields.

## Introduction

Chemistry of hypervalent bromine(III) species has experienced rapid advancements during the recent years [1–2]. The remarkable nucleofugality of aryl bromides in hypervalent bromine(III) compounds has been exploited in the generation of arynes from cyclic diaryl λ^3^-bromanes under remarkably mild conditions with subsequent applications of the in situ-generated arynes in cycloaddition reactions [3], *meta*-selective reactions with oxygen and nitrogen nucleophiles [4–5], regiodivergent *meta* or *ortho*-alkynylations [6], and regioselective (di)halogenation [7]. In addition, cyclic diaryl λ^3^-bromanes have been successfully employed as halogen-bonding organocatalysts in Michael addition [8] and their chiral variants were efficient in catalyzing enantioselective Mannich reactions of ketimines with cyanomethyl coumarins [9] and malonic esters [10]. These notable examples underscore the remarkable potential of cyclic diaryl λ^3^-bromanes in the development of efficient synthetic transformations.

**Scheme 1:**
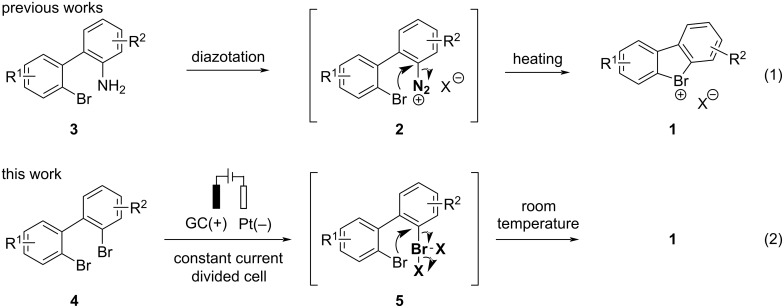
Synthesis of cyclic diarylbromonium compounds.

Cyclic diaryl λ^3^-bromanes **1** are typically synthesized using a method developed by Sandin and Hay in 1952 [11] that relies on the excellent nucleofugality of molecular nitrogen in diazonium compounds. Accordingly, pre-formed [12] or in situ-generated 2,2’-bromodiazonium salts **2** [11,13–14] furnish cyclic bromine(III) species **1** under thermal decomposition conditions (Scheme 1, reaction 1). The diazonium intermediates **2** are obtained by diazotation of 2'-bromo-[1,1'-biphenyl]-2-amines **3** with sodium nitrite and an acid under aqueous conditions. Recently, Wencel-Delord and co-workers disclosed an improved protocol toward diazonium intermediates **2** under non-aqueous conditions (*t*-BuONO and MsOH in acetonitrile) [4]. Nevertheless, the improved method still required thermal decomposition of the diazonium salt to effect the cyclization. In the quest for mild (room temperature) and scalable conditions toward cyclic diaryl λ^3^-bromanes **1** we realized that bromanyl units possess leaving group abilities comparable to the diazonium moiety [1,15]_._ Hence, the oxidation of 2,2'-dibromo-[1,1'-biphenyl] into mono-λ^3^-bromane **5** would set the stage for the key cyclization event (Scheme 1, reaction 2). We also reasoned that an anodic oxidation of the aryl bromide is perfectly suited for the generation of mono-λ^3^-bromane **5** under mild conditions [16–18]. This approach would conceptually differ from previously reported anodic syntheses of cyclic diaryl iodonium compounds, where an electrochemically generated acyclic iodine(III) intermediate undergoes an intramolecular S_E_Ar-type reaction to form the cyclic product [19–20]. Herein, we report on the development of an electrochemical synthesis of cyclic diaryl λ^3^-bromanes under anodic oxidation conditions.

## Results and Discussion

Symmetric 2,2'-dibromo-1,1'-biphenyl **4a** possessing ethoxycarbonyl groups *ortho* to the bromine was chosen as a model compound for our study. We anticipated that the presence of the ester moiety would help to stabilize the key λ^3^-bromane(III) intermediate **5**, as demonstrated in the work of Miyamoto et al. [21], thus facilitating its formation in anodic oxidation. The anodic oxidation of **4a** under previously published conditions for the synthesis of Br(III) species [16,18] (GC as working electrode, Pt foil as counter electrode, 0.1 M tetrabutylammonium tetrafluoroborate (TBA-BF_4_) in HFIP electrolyte and 2 F passed charge at a current density of 10 mA·cm^−2^) afforded the desired Br(III) product **1a** in poor 14% NMR yield (Table 1, entry 1). The cyclic diarylbromonium salt **1a** was isolated by extractive workup followed by reversed-phase chromatography and its structure was unambiguously confirmed by X-ray crystallography (Table 1, graphic). Gratifyingly, a two-fold increase in the yield was achieved by a slight reduction of the current density to *j* = 8 mA·cm^−2^ (entry 2 vs entry 1 in Table 1). A further increase in the yield of **1a** to 45% was possible by increasing the amount of passing charge (6.0 F; Table 1, entry 3). Tetraethylammonium tetrafluoroborate (TEA-BF_4_) appeared to be somewhat superior as the electrolyte to TBA-BF_4_ and tetramethylammonium tetrafluoroborate (TMA-BF_4_) (entry 4 vs entries 3 and 5, Table 1). In all experiments with a passed charge of 6.0 F (Table 1, entries 3–5), nearly complete conversion of starting **4a** and moderate yield of the desired product **1a** was observed pointing at a possible degradation of either starting material or product. Linear sweep voltammetry (LVS) experiments (0.1 M TBA-BF_4_ in HFIP on a Pt disk electrode) revealed that the reduction current increases almost 4 times upon the addition of 5 mM **1a** to the electrolyte (see Supporting Information File 1, Figure S1). At the same time, passing 6.0 F through a solution of **1a** in 50 mM TBA-BF_4_/HFIP at *j* = 8 mA·cm^−2^ led to 60% λ^3^-bromane **1a** degradation, suggesting that cationic **1a**, formed on the anode, decomposes on the cathode. To avoid the undesired cathodic decomposition of **1a**, the cathode and anode chambers were separated, and further experiments were performed in a divided cell. Gratifyingly, the change of the cell type allowed for more than a two-fold increase in the yield of product **1a** from 28% (entry 2, undivided cell, Table 1) to 60% (entry 6, divided cell). Interestingly, higher amounts of passed charge did not improve the reaction outcome (Table 1, entry 7). Neither successful was also a change of the anode material from GC to RVC or BDD (entries 8 and 9, Table 1), variation of electrolyte amount (entries 10 and 11) or altering of counter anions in the supporting electrolyte (entries 12 and 13; for complete optimization results, see Supporting Information File 1, Table S1).

**Table 1:** Optimization of electrochemical oxidation/cyclization conditions.^a^

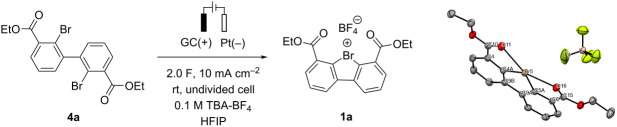				

Entry	Deviation from starting conditions	**1a** [%]	**4a** [%]	Mass balance [%]

1	none	14	61	75
2	*j =* 8 mA cm^−2^	28	49	77
3	*j =* 8 mA cm^−2^, *q*/mol = 6.0 F	45	5	50
4	TEA-BF_4_, *j =* 8 mA cm^−2^, *q*/mol = 6.0 F	48	5	53
5	TMA -BF_4_, *j =* 8 mA cm^−2^, *q*/mol = 6.0 F	42	6	48
**6**	**divided cell, 0.25 M TEA-BF** **_4_** **, ** ***j = *** **8 mA cm** **^−2^**	**60**	**24**	**84**
7	divided cell, 0.25 M TEA-BF_4_, *j =* 8 mA cm^−2^, *q*/mol = 3.0 F	41	19	60
8	divided cell, RVC, 0.25 M TEA-BF_4_, *j =* 8 mA cm^−2^	62	0	62
9	divided cell, BDD, 0.25 M TEA-BF_4_, *j =* 8 mA cm^−2^	57	10	67
10	divided cell, 0.20 M TEA-BF_4_, *j =* 8 mA cm^−2^	15	55	70
11	divided cell, 0.30 M TEA-BF_4_, *j =* 8 mA cm^−2^	15	63	78
12	divided cell, 0.25 M TEA-ClO_4_, *j =* 8 mA cm^−2^	31	26	57
13	divided cell, 0.25 M TEA-PF_6_, *j =* 8 mA cm^−2^	46	19	65

^a^Reactions were performed on a 0.15 mmol scale. Yields and mass balance were determined by ^1^H NMR in the crude reaction mixture using 1,2,3,4-tetrafluorobenzene as an internal standard. ^b^Ellipsoids are shown at 50% probability, with hydrogen atoms omitted for clarity. Selected bond distances (Å) and angles (deg) for **1a**: Br5–O11, 2.657(2); Br5–O16, 2.647(2); Br5–C4A, 1.917(3); Br5–C5A, 1.915(2); Br5–F4, 3.081(5); C4A–Br5–C5A, 86.6(1); C4A–C9B–C9A-C5A, 1.0(3); C4A–C4–C10–O11, 0.1(4), and C5A–C6–C15–O16, 7.8(4).

**Scheme 2:**
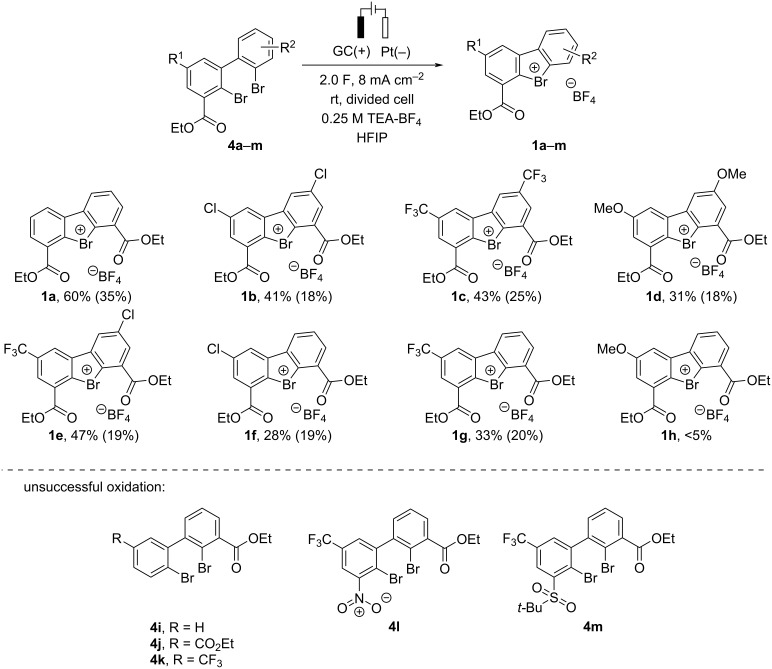
Substrate scope. Reactions were performed on a 0.15 mmol scale. Yields were determined by ^1^H NMR spectroscopy of the reaction mixture using 1,2,3,4-tetrafluorobenzene as an internal standard. Isolated yields are given in parentheses.

With the optimized reaction conditions in hand, next, the substrate scope was evaluated (Scheme 2). Symmetrical biaryls with electron-deficient substituents such as Cl (**4b**) or CF_3_ (**4c**) afforded the respective Br(III) products **1b**,**c** in slightly reduced yields as compared to that of **1a**. Gratifyingly, electron-rich MeO-substituted **1d** could be also obtained under the developed conditions. Unsymmetrically substituted **4e** and monosubstituted dibromides **4f**,**g** demonstrated reactivity similar to that of their symmetrical analogues **4b**,**c** with exception of the mono-MeO-substituted dibromide **4h**. Notably, the presence of two stabilizing ester moieties is critical for the synthesis of Br(III) species: the removal of one ester group (**4i**–**k**), or its replacement by NO_2_ (**4l**) or SO_2_*t*-Bu (**4m**) substituents in 2,2'-dibromo-1,1'-biphenyls resulted in starting material degradation with no formation of the desired product (for a complete list of substrates that do not form the desired electrochemical oxidation product see Supporting Information File 1, Scheme S1).

**Scheme 3:**
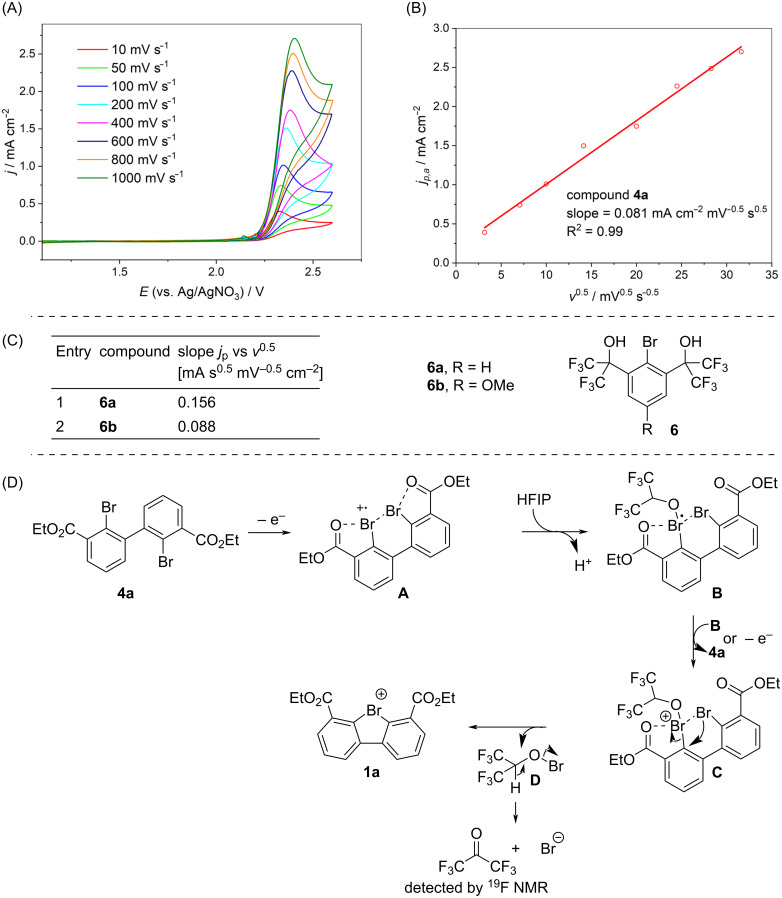
A: Background and *iR* drop-corrected CVs of 5 mM **4a** at different scan rates (solvent: HFIP, working electrode: glassy carbon, supporting electrolyte: 0.1 M TBA-BF_4_). B: Plot of the peak current densities (*j*_p_) vs *v*^0.5^. C: Representative *j*_p_ vs *v*^0.5^ slope values for oxidation of Martin’s bromane precursor **6** (ref. [17]). D: Plausible reaction mechanism.

A series of control experiments was performed to rationalize the observed reactivity trends (Scheme 2). The measured redox potentials *E*_P/2_ for 2,2'-dibromo-1,1'-biphenyls **4a**–**g** (from 1.77 V to 2.88 V) and unsuccessful substrates **4i**–**m**, **S9a**, **S9b** (from 1.73 V to 2.54 V) (Table S2 in Supporting Information File 1) span similar potential regimes suggesting that the success of the anodic oxidation likely depends on the structural rather than the electrochemical properties of the starting 2,2'-dibromo-1,1'-biphenyls **4a**–**m**. A closer inspection of the electrochemical behaviour of compound **4a** revealed an irreversible electron transfer at scan rates up to 1 V s^−1^ (Scheme 3A) indicating that one or more rapid chemical steps are following the electrochemical step [22–23]. Besides, the observed linear correlation between *E*_p_ of the redox event and the square root of the scan rate (Scheme 3B) suggested that compound **4a** is not significantly adsorbed on the electrode surface [23]. Comparison of the *j*_p_ vs *v*^0.5^ slope with our previously obtained results for the anodic oxidation of aryl bromides **6a** (two-electron oxidation) and **6b** (one-electron oxidation) into the respective bromine(III) species (Scheme 3C) [17] demonstrated a more similar behaviour to **6b** suggesting that revealed oxidation is a single-electron-transfer process. It is important to note that this comparison assumes that the diffusion coefficients of **4a** and **6**, parameters that influence the *j*_p_ vs *v*^0.5^ slope, are similar. On the other hand, anodic oxidation of **4a** under optimized conditions (entry 6, Table 1) using 1 F returned only 35% of **1a** (NMR yield), whereas passing 2 F charge delivered **1a** in 60% yield. The latter data provides an evidence that the overall oxidation of **4a** to **1a** likely is a two-electron process, suggesting that the second oxidation step may involve disproportionation of putative Br(II) species (see Scheme 3D).

Based on the control experiments described above we propose a plausible mechanism as shown in Scheme 3D. The reaction starts with a single-electron oxidation of **4a** on the electrode surface to form cation radical **A**, in which Br(II) is chelation-stabilized by the carboxyl group [21] and the neighbouring Br substituent [24]. Intermediate **A** rapidly undergoes irreversible chemical reaction by HFIP coordination to transient Br(II) followed by subsequent deprotonation to generate radical **B**. A following disproportionation of radical **B** would lead to the formation of Br(III) species **C** (anodic oxidation cannot be fully excluded), which undergoes intramolecular S_N_Ar-type substitution to form cyclic λ^3^-bromane **1a** and hypobromite **D**. The latter decomposes into bromide and hexafluoroacetone, with the latter observed by ^19^F NMR [25]. Assuming that intermediate **B** is sufficiently stable to leave the diffusion layer and undergoes disproportionation to the species **C** in the bulk electrolyte provides a reasonable explanation for why the reaction appears as a one-electron oxidation in CV experiments, but still as a two-electron oxidation in electrolysis.

## Conclusion

In conclusion, we have demonstrated a conceptual approach to cyclic diaryl λ^3^-bromanes by electrochemical oxidative cyclization of 2,2'-dibromo-1,1'-biphenyls. The developed method represents a safe and inexpensive alternative to the commonly used thermal decomposition of potentially explosive diazonium salts. The successful electrochemical oxidation requires the presence of two chelating ester groups that stabilize the formed Br(III) species. Further work towards improving the substrate scope and understanding the reaction mechanism are in progress in our laboratory.

## Supporting Information

Crystallographic data for the structure reported in this paper have been deposited with the Cambridge Crystallographic Data Centre and allocated the deposition number CCDC 2404146.

File 1 Experimental procedures, analytical and spectroscopic data for new compounds, copies of NMR spectra, and X-ray crystallographic data.

## Data Availability

All data that supports the findings of this study is available in the published article and/or the supporting information of this article.
