# Supplementary material for: Electrochemical synthesis of cyclic biaryl λ3-bromanes from 2,2’-dibromobiphenyls
Source: Beilstein J Org Chem. 2025 Feb 27;21:451–7. doi: 10.3762/bjoc.21.32 (PMC11878128; doi:10.3762/bjoc.21.32)

BEILSTEIN JOURNAL OF ORGANIC CHEMISTRY

Supporting Information

for

Electrochemical synthesis of cyclic biaryl λ^3-bromanes from 2,2'-dibromobiphenyls

Andrejs Savkins and Igors Sokolovs

Beilstein J. Org. Chem. **2025,** *21,* 451–457. doi:10.3762/bjoc.21.32

Experimental procedures, analytical and spectroscopic data for new compounds, copies of NMR spectra, and X-ray crystallographic data

License and Terms: This is a supporting information file under the terms of the Creative Commons Attribution License (https://creativecommons.org/licenses/by/4.0). Please note that the reuse, redistribution and reproduction in particular requires that the author(s) and source are credited and that individual graphics may be subject to special legal provisions.
The license is subject to the *Beilstein Journal of Organic Chemistry* terms and conditions: (https://www.beilstein-journals.org/bjoc/terms)

Table of contents

General information S2

Synthesis of bromobiphenyls 4 for electrochemical oxidation S2

General procedure A for esterification of carboxylic acids **S1** S2
General procedure B for iodide S4 synthesis from nitrobenzoates **S2** S3
Synthesis of ethyl 2-bromo-5-chloro-3-iodobenzoate (**S4d**). S4
Synthesis of ethyl 2-bromo-3-iodo-5-(trifluoromethyl)benzoate (**S4f**). S6
Synthesis of (2-bromo-3-iodo-5-(trifluoromethyl)phenyl)(*tert*-butyl)sulfone (**S4m**). . S10
General procedure C for boronic acid S7 synthesis from iodide **S4** S12
Synthesis of ethyl 2-bromo-5-methoxy-3-(4,4,5,5-tetramethyl-1,3,2-dioxaborolan-2-yl)benzoate (**S7e**). S14
General procedure D for trimethylstannane **S8** synthesis from iodide **S4** S15
General procedure E for Suzuki–Miyaura reaction S16
General procedure F for Stille reaction S20
Synthesis of diethyl 2,2'-dibromo-5,5'-dimethoxy-[1,1'-biphenyl]-3,3'-dicarboxylate (**4d**). S25

Optimization of electrochemical oxidation/cyclization of 2,2'-dibromo-1,1'-biphenyl **4a** S28

λ^3-Bromane 1 synthesis via electrochemical oxidation of bromobiphenyls **4** S31

General procedure G for electrochemical oxidation of bromobiphenyls **4** S31

Crystal data and structure refinement for biaryl λ^3-bromane **1a** S35

Cyclic voltammetry S36

Anodic oxidation of bromobiphenyls **4**, **S9a**,**b** S36
Cathodic reduction of λ^3-bromane **1a** S43

References S44

Spectra S46

General information

Unless otherwise noted, all chemicals were used as received from commercial sources. Anhydrous THF was obtained by passing commercially available anhydrous solvents through activated alumina columns. TEA-BF_4 was dried under reduced pressure at 90 °C for 5 h prior to use. The glassy carbon electrodes (SIGRADUR G) were purchased from HTW GmbH, Germany. The solvent 1,1,1,3,3,3-hexafluoro-2-propanol (99%) was purchased from Fluorochem, UK, and used as received. Analytical thin-layer chromatography (TLC) was performed on pre-coated silica gel F-254 plates. Nuclear magnetic resonance spectra were recorded on Bruker Avance 400 or Bruker Fourier 300 NMR spectrometers at the following frequencies: ^{1}H, 400 or 300 MHz; ^{13}C{^{1}H}, 100.6 or 75 MHz; ^{19}F, 376.3 MHz. Chemical shifts are reported in parts per million (ppm) with the residual solvent peak as an internal reference. High-resolution mass spectra (HRMS) were recorded on mass spectrometers with a time-of-flight (TOF) mass analyzer using ESI techniques.

Synthesis of bromobiphenyls 4 for electrochemical oxidation

General procedure A for esterification of carboxylic acids S1

A flame-dried round-bottomed flask was flushed with a stream of argon and charged with carboxylic acid **S1** (1.0 equiv) and absolute EtOH (1.5 mL per mmol of carboxylic acid **S1**). Thionyl chloride (3.0 equiv) was then added dropwise, and the resulting yellowish solution was heated under reflux for 3 h. Then, it was cooled to room temperature and all volatiles were removed by distillation under reduced pressure. Saturated aqueous $NaHCO_3$ solution (30 mL) was added, and the yellowish semi-solid residue was extracted with CH_2Cl_2 (3 × 20 mL). The combined organic extracts were washed with water, brine, dried over Na_2SO_4, and concentrated under reduced pressure to afford an oil.

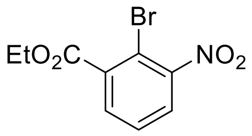

Ethyl 2-bromo-3-nitrobenzoate (S2a).
Following General Procedure A, 2-bromo-3-nitrobenzoic acid **S1a** (2.50 g, 10.16 mmol) was converted into **S2a**. Yellowish oil (2.64 g, 95% yield).
^{1}H NMR (300 MHz, $CDCl_3$, ppm) δ 7.83 (1H, dd, *J* = 7.9, 1.7 Hz), 7.75 (1H, dd, *J* = 7.9, 1.7 Hz), 7.51 (1H, t, *J* = 7.9 Hz), 4.43 (2H, q, *J* = 7.1 Hz), 1.41 (3H, t, *J* = 7.1 Hz);

$^{13}C\{^1H\}$ **NMR** (75 MHz, $CDCl_3$, ppm) δ 165.3, 152.1, 136.4, 133.0, 128.3, 126.6, 112.8, 62.6, 14.2;

Elemental analysis (%): calculated for $C_9H_8BrNO_4$: C, 39.44; H, 2.94; N, 5.11; found: C, 39.47; H, 2.96; N, 5.10.

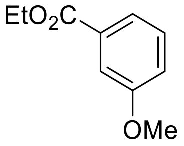

Ethyl 3-methoxybenzoate (S2c).

Following General Procedure A, 3-methoxybenzoic acid **S1c** (800 mg, 5.26 mmol) was converted into **S2c**. Pale yellow oil (900 mg, 94% yield).

^{1}**H NMR** (300 MHz, $CDCl_3$, ppm) δ 7.64 (1H, dt, *J* = 7.7 Hz, 1.4 Hz), 7.58 – 7.55 (1H, m), 7.34 (1H, t, *J* = 7.7 Hz), 7.13 – 7.06 (1H, m), 4.38 (2H, q, *J* = 7.1 Hz), 3.86 (3H, s), 1.39 (3H, t, *J* = 7.1 Hz). ^{1}H NMR spectrum was consistent with that reported in the literature[1].

General procedure B for iodide S4 synthesis from nitrobenzoates S2

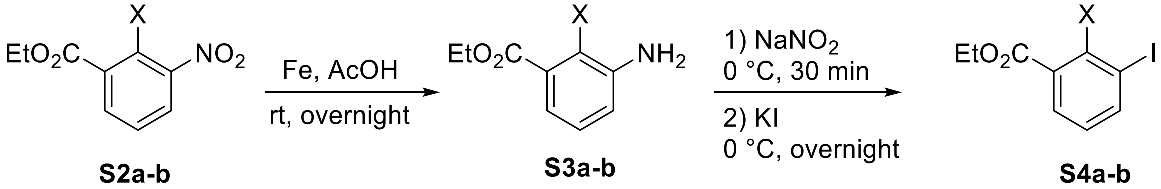

Following a reported procedure[2], a round-bottomed flask was charged with ester **S2** (1.0 equiv), glacial AcOH (3.0 mL per mmol of ester **S2**), and iron powder (5.0 equiv). The red reaction mixture was well-stirred overnight at room temperature, the remaining iron powder was filtered off from the dark-red suspension and washed with AcOH on the filter. Water (30 mL) and brine (30 mL) was added to the red reaction mixture, and it was extracted with EtOAc (3 × 40 mL). The combined organic extracts were dried over Na_2SO_4 and concentrated under reduced pressure. The resulting crude aminobenzoate **S3** (red amorphous solid) was used in the further step without additional purification.

Following a reported procedure[3], a suspension of aminobenzoate **S3** from above (1.0 equiv) in a 1:1 (v/v) mixture of conc. HCl and H_2O (4 mL per mmol of aminobenzoate) was cooled in an ice bath. $NaNO_2$ (1.5 equiv) solution in water (1 mL per mmol of $NaNO_2$) was added dropwise to the well-stirred pale yellow reaction mixture. The resulting suspension was stirred for 30 minutes, then it was added dropwise to KI (3.0 equiv) solution in water (2 mL per mmol of KI) which was cooled in an ice bath. The dark-red solution was stirred overnight. Crude Na_2SO_3 was added to the dark-

green reaction mixture until the color disappeared, and the resulting pale yellow suspension was extracted with EtOAc (3 × 50 mL). The combined organic extracts were washed with aqueous $NaHCO_3$ solution, brine, dried over Na_2SO_4, and concentrated under reduced pressure. The resulting crude product was purified by flash chromatography using gradient elution from 100% petroleum ether to 20% EtOAc/petroleum ether yielding iodide **S4**.

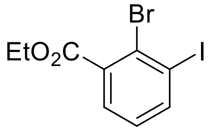

Ethyl 2-bromo-3-iodobenzoate (S4a).

Following General Procedure B, ester **S2a** (2.64 g, 9.63 mmol) was converted into **S4a**. Pale yellow oil (2.81 g, 84% yield); analytical TLC on silica gel, 1:9 EtOAc/petroleum ether, R_f=0.26.

^{1}H NMR (400 MHz, $CDCl_3$, ppm) δ 7.98 (1H, dd, J = 7.8, 1.6 Hz), 7.56 (1H, dd, J = 7.8, 1.6 Hz), 7.06 (1H, t, J = 7.8 Hz), 4.40 (2H, q, J = 7.1 Hz), 1.39 (3H, t, J = 7.1 Hz);

^{13}C{^{1}H} NMR (101 MHz, $CDCl_3$, ppm) δ 166.6, 142.6, 135.9, 129.6, 128.4, 127.6, 104.6, 62.2, 14.3;

HRMS (ESI/Q-TOF) *m/z*: $[M+H]^+$ calculated for $C_9H_9BrIO_2^+$ 354.8831, found 354.8835.

NMR spectrums were consistent with that reported in the literature[4]

Synthesis of ethyl 2-bromo-5-chloro-3-iodobenzoate (S4d).

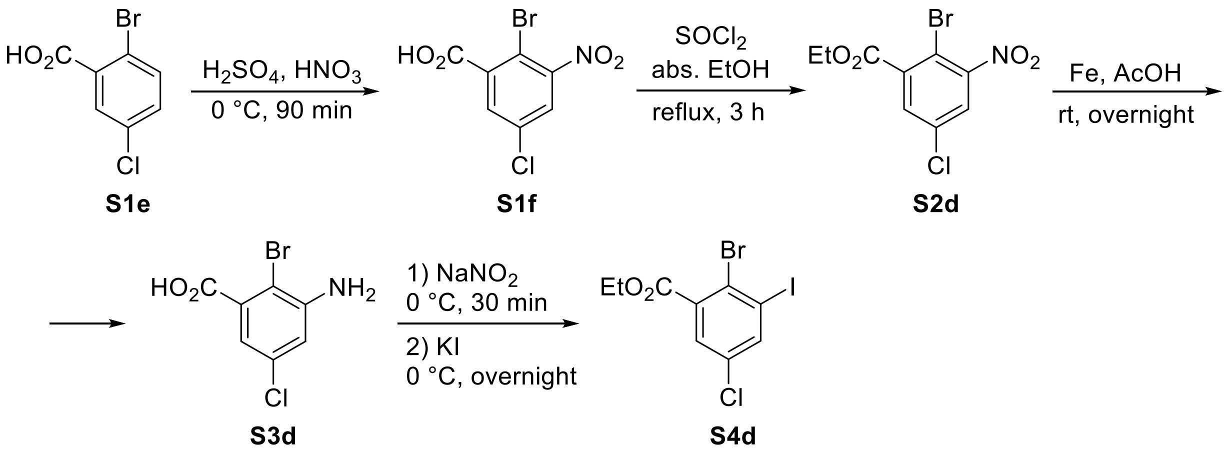

Following a reported procedure[5], a suspension of 2-bromo-5-chlorobenzoic acid (**S1e**, 5.00 g, 21.2 mmol, 1.0 equiv) in 98% sulfuric acid (12 mL) was cooled to 0 °C (crushed ice bath). Fuming nitric acid (1.18 mL, 23.4 mmol, 1.1 equiv) was then added dropwise, and the resulting brown suspension was stirred at 0 °C for 90 min, then poured into ice water (50 mL) and extracted with

ethyl acetate (3 × 20 mL). The combined organic extracts were dried over Na_2SO_4, and concentrated under reduced pressure. The resulting crude nitrobenzoic acid **S1f** (brown solid) was used in the further step without additional purification.

A 100 mL flame-dried round-bottomed flask was flushed with a stream of argon and charged with nitrobenzoic acid **S1f** from above (5.82 g, 20.8 mmol, 1.0 equiv) and absolute EtOH (30 mL). Thionyl chloride (4.62 mL, 63.7 mmol, 3.0 equiv) was then added dropwise, and the resulting yellowish solution was heated under reflux for 3 h. It was cooled to room temperature and all volatiles were removed by distillation under reduced pressure. Saturated aqueous $NaHCO_3$ solution (30 mL) was added, and the yellowish semi-solid residue was extracted with CH_2Cl_2 (3 × 10 mL). The combined organic extracts were washed with water, brine, dried over Na_2SO_4, and concentrated under reduced pressure. The resulting crude nitrobenzoate **S2d** (reddish amorphous solid) was used in the further step without additional purification.

Following a reported procedure[2], a round-bottomed flask was charged with nitrobenzoate **S2d** from above (6.16 g, 20.0 mmol, 1.0 equiv), glacial AcOH (50 mL), and iron powder (7.84 g, 0.140 mol, 7.0 equiv). The red reaction mixture was well-stirred overnight at room temperature, the remaining iron powder was filtered off from the dark-red suspension and washed with AcOH on filter. The filtrate was concentrated under reduced pressure. The resulting crude aminobenzoate **S3d** (red amorphous solid) was used in the further step without additional purification.

Following a reported procedure[3], a suspension of aminobenzoate **S3d** from above (5.00 g, 20.0 mmol, 1.0 equiv), in a 1:1 (v/v) mixture of conc. HCl and H_2O (80 mL) was cooled in an ice bath. $NaNO_2$ (2.07 g, 31.9 mmol, 1.5 equiv) solution in water (25 mL) was added dropwise to the well-stirred pale yellow reaction mixture. The resulting suspension was stirred for 30 minutes, then it was added dropwise to KI (9.96 g, 63.7 mmol, 3.0 equiv) solution in water (100 mL) which was cooled in an ice bath. The dark-red solution was stirred overnight. Crude Na_2SO_3 was added to the dark-green reaction mixture until the color disappeared, and the resulting pale-yellow suspension was extracted with EtOAc (3 × 70 mL). The combined organic extracts were washed with aqueous $NaHCO_3$ solution, brine, dried over Na_2SO_4, and concentrated under reduced pressure. The resulting crude product was purified by flash chromatography using gradient elution from 100% petroleum ether to 30% EtOAc/petroleum ether. Colorless oil (2.10 g, 25% yield); analytical TLC on silica gel, 1:9 EtOAc/petroleum ether, R_f=0.35.

^{1}H NMR (400 MHz, $CDCl_3$, ppm) δ 7.96 (1H, d, *J* = 2.4 Hz), 7.56 (1H, d, *J* = 2.4 Hz), 4.40 (2H, q, *J* = 7.1 Hz), 1.39 (3H, t, *J* = 7.1 Hz);

^{13}C{^{1}H} NMR (101 MHz, $CDCl_3$, ppm) δ 165.4, 141.7, 136.2, 133.8, 129.8, 126.1, 105.0, 62.6, 14.2;

HRMS (ESI): m/z $[M+H]^+$ calculated for $C_9H_8O_2ClBrI^+$ 388.8441, found 388.8453.

Synthesis of ethyl 2-bromo-3-iodo-5-(trifluoromethyl)benzoate (S4f).

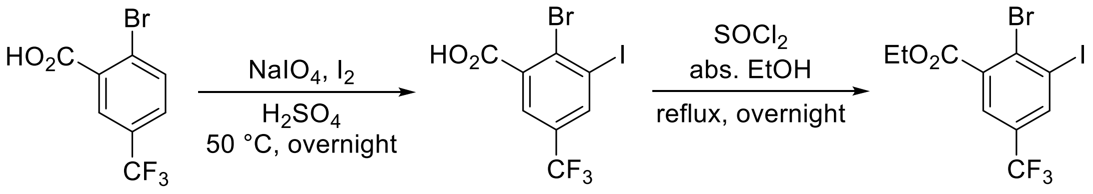

S1g **S4e** **S4f**

Following a reported procedure[6], sodium periodate (280 mg, 1.31 mmol, 0.16 equiv) was added gradually within 5 min to a well-stirred suspension of powdered iodine (976 mg, 3.84 mmol, 0.47 equiv) in 95% sulfuric acid (25 mL). The stirring was continued for 30 min at room temperature to afford a dark-brown solution. 2-Bromo-5-(trifluoromethyl)benzoic acid (**S1g**, 2.20 g, 8.18 mmol, 1.0 equiv) was added neat in one portion to the solution and the resulting dark-brown reaction mixture was stirred for 18 h at 50 °C, whereupon it was cooled to room temperature and poured into 200 g of crushed ice (*Caution! Heat evolution!*). The resulted suspension was extracted with DCM (3 × 80 mL), the combined pink organic layers were washed with brine, dried (Na_2SO_4), and concentrated. The resulting crude iodobenzoic acid **S4e** (colorless oil) was used in the further step without additional purification.

A flame-dried round-bottomed flask was flushed with a stream of argon and charged with iodobenzoic acid **S4e** from above (3.14 g, 8.00 mmol, 1.0 equiv) and absolute EtOH (15 mL). Thionyl chloride (1.73 mL, 24 mmol, 3.0 equiv) was then added dropwise, and the resulting yellowish solution was heated under reflux overnight. It was cooled to room temperature and all volatiles were removed by distillation under reduced pressure. Saturated aqueous $NaHCO_3$ solution (70 mL) was added, and the yellowish semi-solid residue was extracted with CH_2Cl_2 (3 × 50 mL). The combined organic extracts were washed with water, brine, dried over Na_2SO_4, and concentrated under reduced pressure. The resulting crude product was purified by flash chromatography using gradient elution from 5% EtOAc/petroleum ether to 30% EtOAc/petroleum

ether, after that reversed phase column chromatography on C18 silica gel using gradient elution from 10% MeCN in 0.1% TFA in water to 95% MeCN in 0.1% TFA in water afforded product as colorless oil (2.30 g, 67% yield); analytical TLC on silica gel, 1:9 EtOAc/petroleum ether, R_f=0.40.
^{1}**H NMR** (400 MHz, $CDCl_3$, ppm) δ 8.19 (1H, dd, J = 2.2, 0.7 Hz), 7.81 (1H, dd, J = 2.2, 0.7 Hz), 4.43 (2H, q, J = 7.1 Hz), 1.41 (3H, t, J = 7.1 Hz);
^{13}**C{^{1}H} NMR** (101 MHz, $CDCl_3$, ppm) δ 165.4, 138.9 (q, $^3J_{C-F}$ = 3.6 Hz), 136.2, 132.2, 130.8 (q, $^2J_{C-F}$ = 33.9 Hz), 126.5 (q, $^3J_{C-F}$ = 3.6 Hz), 121.1 (q, $^1J_{C-F}$ = 273.1 Hz), 105.0, 62.8, 14.2;
^{19}**F NMR** (376 MHz, $CDCl_3$, ppm) δ -62.9;
HRMS (ESI): m/z $[M+H]^+$ calculated for $C_{10}H_8O_2F_3BrI^+$ 422.8704, found 422.8696.

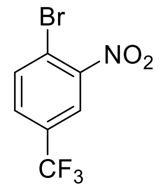

1-Bromo-2-nitro-4-(trifluoromethyl)benzene (S5a).
Following a reported procedure[5], a mixture of 4-bromotrifluoromethylbenzene (**S5b**, 4.20 mL, 30.00 mmol, 1.0 equiv) in 95% sulfuric acid (15.0 mL) was cooled in an ice bath, followed by dropwise addition of fuming nitric acid (2 mL). The resulted yellowish suspension was stirred for 90 min at 0 °C, whereupon it was poured into crushed ice (100 g) and extracted with DCM (3 × 50 mL). The combined organic layers were dried with Na_2SO_4 and concentrated. The resulting crude product was purified by flash chromatography using gradient elution from 5% EtOAc/petroleum ether to 15% EtOAc/petroleum ether to afford product as yellow oil (6.78 g, 96% yield); analytical TLC on silica gel, 1:10 EtOAc/petroleum ether, R_f=0.41.
^{1}**H NMR** (300 MHz, $CDCl_3$, ppm) δ 8.11 (1H, d, J = 2.2 Hz), 7.92 (1H, d, J = 8.4 Hz), 7.69 (1H, dd, J = 8.4, 2.2 Hz). ^{1}H NMR spectrum was consistent with that reported in the literature[7].

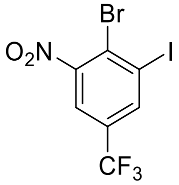

2-Bromo-1-iodo-3-nitro-5-(trifluoromethyl)benzene (S4g).
Following a reported procedure[6], sodium periodate (636 mg, 2.97 mmol, 0.16 equiv) was added gradually within 5 min to a well-stirred suspension of powdered iodine (2.22 g, 8.74 mmol, 0.47 equiv) in 95% sulfuric acid (80 mL). The stirring was continued for 30 min at room temperature to afford a dark-brown solution. 4-Bromo-3-nitrobenzotrifluoride (**S5a**, 5.00 g, 18.58 mmol, 1.0 equiv) was added neat in one portion to the solution and the resulting dark brown reaction mixture was stirred for 4 days at 50 °C. The dark-brown reaction suspension was cooled to room temperature and poured into 200 g

of crushed ice (*Caution! Heat evolution!*). The resulted suspension was extracted with DCM (3 × 80 mL). The combined pink organic layers were washed with brine, dried (Na_2SO_4) and concentrated. The resulting crude product was purified by flash chromatography using isocratic elution with 5% Et_2O/petroleum ether to afford product as pale yellow powder (4.64 g, 63% yield); analytical TLC on silica gel, petroleum ether, R_f=0.18.

^{1}H NMR (400 MHz, $CDCl_3$, ppm) δ 8.29 (1H, dd, *J* = 2.0, 0.7 Hz), 7.89 (1H, dd, *J* = 2.0, 0.7 Hz);

^{13}C{^{1}H} NMR (101 MHz, $CDCl_3$, ppm) δ 151.1, 139.5 (q, $^3J_{C\text{-}F}$ = 3.6 Hz), 131.9 (q, $^2J_{C\text{-}F}$ = 34.9 Hz), 126.2, 121.7 (q, $^1J_{C\text{-}F}$ = 273.8 Hz), 121.5 (q, $^3J_{C\text{-}F}$ = 3.6 Hz), 105.7;

^{19}F NMR (376 MHz, $CDCl_3$, ppm) δ -63.0;

Elemental analysis (%): calculated for $C_7H_2BrF_3INO_2$: N, 3.54; C, 21.24; H, 0.51; found: N, 3.49; C, 21.40; H, 0.56.

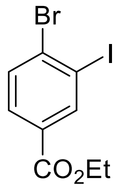

Ethyl 4-bromo-3-iodobenzoate (S4h).

Following a reported procedure[6], sodium periodate (323 mg, 1.51 mmol, 0.16 equiv) was added gradually within 5 min to a well-stirred suspension of powdered iodine (1.13 g, 4,45 mmol, 0.47 equiv) in 95% sulfuric acid (40 mL). The stirring was continued for 30 min at room temperature to afford a dark-brown solution. 4-Bromobenzoic acid (**S5c**, 1.90 g, 9.45 mmol, 1.0 equiv) was added neat in one portion to the solution and the resulting dark brown reaction mixture was stirred for 18 h at room temperature, whereupon it was poured into 200 g of crushed ice (*Caution! Heat evolution!*). The resulted suspension was extracted with EtOAc (3 × 80 mL). The combined pink organic layers were washed with brine, dried (Na_2SO_4) and concentrated. The resulting crude iodobenzoic acid **S4i** (brownish powder) was used in the further step without additional purification.

A flame-dried round-bottomed flask was flushed with a stream of argon and charged with iodobenzoic acid (**S4i**) from above (3.09 g, 9.44 mmol, 1.0 equiv) and absolute EtOH (20 mL). Thionyl chloride (1.37 mL, 18.88 mmol, 2.0 equiv) was then added dropwise, and the resulting yellowish solution was heated under reflux overnight. It was cooled to room temperature and all volatiles were removed by distillation under reduced pressure. Saturated aqueous $NaHCO_3$ solution (70 mL) was added, and the yellowish semi-solid residue was extracted with CH_2Cl_2 (3 × 50 mL). The combined organic extracts were washed with water, brine, dried over Na_2SO_4, and concentrated under reduced pressure. The resulting crude product was purified by flash

chromatography using isocratic elution with 5% Et_2O/petroleum ether to afford product as colorless powder (3.27 g, 97% yield); analytical TLC on silica gel, 1:10 Et_2O/petroleum ether, R_f=0.43.

^{1}H NMR (400 MHz, $CDCl_3$, ppm) δ 8.49 (1H, d, *J* = 2.0 Hz), 7.85 (1H, dd, *J* = 8.3, 2.0 Hz), 7.68 (1H, d, *J* = 8.3 Hz), 4.37 (2H, q, *J* = 7.1 Hz), 1.39 (3H, t, *J* = 7.1 Hz);

^{13}C{^{1}H} NMR (101 MHz, $CDCl_3$, ppm) δ 164.7, 141.3, 135.3, 132.8, 130.8, 130.4, 101.1, 61.7, 14.4;

Elemental analysis (%): calculated for $C_9H_8BrIO_2$: C, 30.45; H, 2.27; found: C, 30.50; H, 2.26.

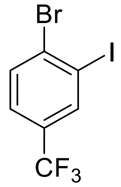

1-Bromo-2-iodo-4-(trifluoromethyl)benzene (**S4j**).

Following a reported procedure[6], to a suspension of $NaIO_4$ (5.13 g, 24.0 mmol, 1.2 equiv) and I_2 (6.09 g, 24.0 mmol, 1.2 equiv) in a 2:1 mixture of acetic acid and acetic anhydride (30 mL) a 95% sulfuric acid (30.0 mL) was added dropwise at 10 °C (cold water bath) followed by dropwise addition of 4-bromotrifluorotoluene (**S5d**, 2.8 mL, 20.0 mmol, 1.0 equiv). The resulted dark suspension was stirred at room temperature for 18 h, whereupon it was poured into 50 mL crushed ice (*Caution! Heat evolution!*). Crude Na_2SO_3 was added to dark-brown emulsion until the color disappeared. Resulted yellow emulsion was extracted with DCM (3 × 50 mL). The combined organic extracts were washed with water, brine, dried over Na_2SO_4, and concentrated under reduced pressure. The resulting crude product was purified by flash chromatography using isocratic elution with 5% Et_2O/petroleum ether to afford product as colorless oil (6.78 g, 96%); analytical TLC on silica gel, petroleum ether, R_f=0.62.

^{1}H NMR (400 MHz, $CDCl_3$, ppm) δ 8.09 (1H, d, *J* = 2.0 Hz), 7.74 (1H, d, *J* = 8.4 Hz), 7.46 (1H, dd, *J* = 8.4, 2.0 Hz). ^{1}H NMR spectrum was consistent with that reported in the literature[8].

Synthesis of (2-bromo-3-iodo-5-(trifluoromethyl)phenyl)(*tert*-butyl)sulfone (S4m).

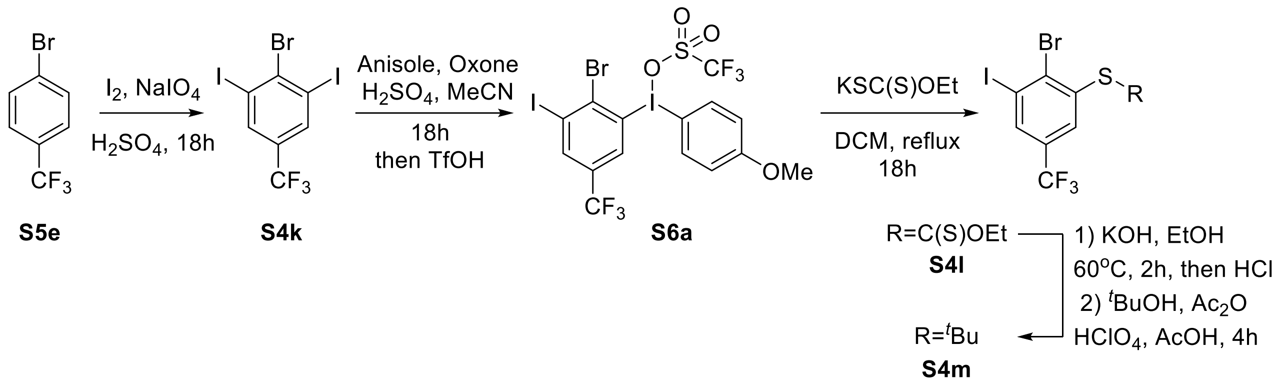

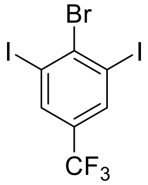

2-Bromo-1,3-diiodo-5-(trifluoromethyl)benzene (S4k).

Following a reported procedure[6], sodium periodate (1.36 g, 6.36 mmol, 0.32 equiv) was added gradually within 5 min to a well stirred suspension of powdered iodine (4.8 g, 18.9 mmol, 0.95 equiv) in 95% H_2SO_4 (100 mL). The stirring was continued for 30 min at room temperature to afford a dark-brown solution. 2-Bromo-5-(trifluoromethyl)benzene (**S5e**, 2.8 mL, 20.0 mmol, 1.0 equiv) was added dropwise within ≈2 min to the solution and the resulting dark brown reaction mixture was stirred for 18 h at room temperature, whereupon it was poured into 100 mL of crushed ice (*Caution! Heat evolution!*). The formed pink precipitate was filtered and carefully washed with water until pH 6. The crude product was recrystallized from methanol to afford colorless needles (8.53 g, 89%); mp 88-89 °C; analytical TLC on silica gel, petroleum ether, R_f=0.67.

^{1}H NMR (400 MHz, $CDCl_3$, ppm) δ 8.06 (2H, q, $^4J_{H-F}$ = 0.6 Hz). ^{1}H NMR spectrum was consistent with that reported in the literature[9].

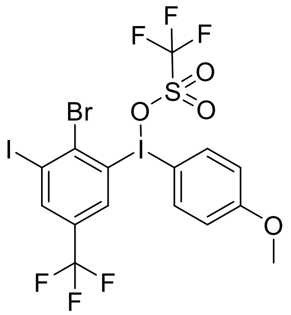

(2-Bromo-3-iodo-5-(trifluoromethyl)phenyl)(4-methoxyphenyl)-λ^3-iodaneyl trifluoromethanesulfonate (S6a).

Following a reported procedure[10], diiodobrombenzene **S4k** (13.0 g, 27.3 mmol, 1.0 equiv) and anisole (4.4 mL, 40.9 mmol, 1.5 equiv) were dissolved in MeCN (150 mL) followed by Oxone® (16.8 g, 27.3 mmol, 1.0 equiv). To the resulting colorless suspension, cooled in ice-bath and well stirred, 95% sulfuric acid (11 mL, 204.5 mmol, 7.5 equiv) was added dropwise within 5 min. The dark blue suspension was stirred at room temperature for 18 h, whereupon TfOH (4.8 mL,

54.5 mmol, 2.0 equiv) solution in water (500 mL) was added and extracted with DCM (3 × 150 mL). The combined organic extracts were washed with water (200 mL), dried over Na_2SO_4, and concentrated under reduced pressure. The dark brown oil was triturated with petroleum ether (3 × 20 mL), dissolved in small amount of DCM and Et_2O (150 mL) was added. Filtration of precipitate afforded the title compound (8.25 g, 41%) as off white powder.

^{1}H NMR (400 MHz, DMSO-*d*$_6$, ppm) δ 9.04 – 9.00 (1H, m), 8.54 – 8.50 (1H, m), 8.23 – 8.17 (2H, m), 7.15 – 7.11 (2H, m), 3.81 (3H, s);

^{13}C{^{1}H} NMR (101 MHz, DMSO-*d*$_6$, ppm) δ 162.4, 139.9 (q, $^3J_{C-F}$ = 3.8 Hz), 138.9, 137.3, 134.7 (q, $^3J_{C-F}$ = 3.4 Hz), 131.1 (q, $^2J_{C-F}$ = 33.6 Hz), 123.0, 121.7 (q, $^1J_{C-F}$ = 273.9 Hz), 120.7 (q, $^1J_{C-F}$ = 322.0 Hz), 117.8, 106.4, 104.4, 55.8;

^{19}F NMR (376 MHz, DMSO-*d*$_6$, ppm) δ -63.0, -77.7;

HRMS (ESI): m/z $[M\text{-}OSO_2CF_3]^+$ calculated for $C_{16}H_{19}O_2^+$: 243.1385, found 243.1386.

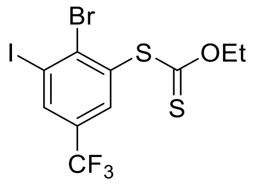

S-(2-Bromo-3-iodo-5-(trifluoromethyl)phenyl) O-ethyl carbonodithioate (S4l).

Following a reported procedure[11], iodane (**S6a**) (6.0 g, 8.19 mmol, 1.0 equiv) and potassium ethyl xanthate (2.6 g, 16.37 mmol, 2.0 equiv) were suspended in DCM (80 mL) and stirred under reflux for 18 h. The resulted yellow suspension was cooled to room temperature and concentrated under reduced pressure. The resulting crude product was purified by flash chromatography using isocratic elution with 5% DCM/petroleum ether to afford product as yellow oil (2.4 g, 62% yield); analytical TLC on silica gel, petroleum ether, R_f=0.30.

^{1}H NMR (400 MHz, $CDCl_3$, ppm) δ 8.13 (1H, dq, *J* = 2.2, 0.7 Hz), 7.85 (1H, dq, *J* = 2.2, 0.7 Hz), 4.63 (2H, q, *J* = 7.1 Hz), 1.34 (3H, t, *J* = 7.1 Hz);

^{13}C{^{1}H} NMR (101 MHz, $CDCl_3$, ppm) δ 208.2, 141.5, 138.6 (q, $^3J_{C-F}$ = 3.8 Hz), 134.0, 133.3 (q, $^3J_{C-F}$ = 3.4 Hz), 131.4 (q, $^2J_{C-F}$ = 33.8 Hz), 122.3 (q, $^1J_{C-F}$ = 273.6 Hz), 103.1, 71.1, 13.7;

^{19}F NMR (376 MHz, $CDCl_3$, ppm) δ -62.8;

HRMS (ESI): m/z $[M\text{-}H]^-$ calculated for $C_{10}H_6F_3BrOS_2I^+$: 468.8040, found 468.8036.

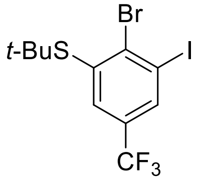

(2-Bromo-3-iodo-5-(trifluoromethyl)phenyl)(*tert*-butyl)sulfone (S4m). Following a reported procedure[11], the xanthate ester **S4l** (2.0 g, 4.24 mmol, 1.0 equiv) was dissolved in EtOH (20 mL). Argon was bubbled through the solution for 15 min, whereupon KOH (702 mg, 12.74 mmol, 3.0 equiv) was added. The resulted pale yellow suspension was stirred at 60 °C for 2 h under argon atmosphere, then cooled to room temperature and acidified to pH 5 with 4 M HCl solution in water. The resulting yellowish emulsion was extracted with Et_2O (3 × 30 mL). The combined organic extracts were washed with brine (30 mL), dried over Na_2SO_4 and concentrated under reduced pressure to afford a colorless oily residue, which was used in subsequent step without additional purification. Following a reported procedure[12], the oily residue from above and *tert*-butanol (487 μL, 5.09 mmol, 1.2 equiv) were dissolved in glacial acetic acid (7 mL). The clear reaction solution was cooled in an ice bath followed by addition of acetic anhydride (440 μL, 4.67 mmol, 1.1 equiv) and perchloric acid (70 wt % in water, 350 μL, 3.89 mmol, 0.9 equiv). The resulted reaction mixture was stirred for 4 h at room temperature, whereupon it was diluted with water (50 mL) and extracted with Et_2O (3 × 30 mL). The combined organic extracts were washed with water, brine, dried over Na_2SO_4, and concentrated under reduced pressure. The resulting crude product was purified by flash chromatography using isocratic elution with 100% petroleum ether to afford product as colorless oil (915 mg, 49%); analytical TLC on silica gel, petroleum ether, R_f=0.46.

^{1}H NMR (400 MHz, $CDCl_3$, ppm) δ 8.03 (1H, dd, J = 2.2, 0.8 Hz), 7.84 (1H, dd, J = 2.2, 0.8 Hz), 1.38 (9H, s);

^{13}C{^{1}H} NMR (101 MHz, $CDCl_3$, ppm) δ 143.0, 137.6, 137.0 (q, $^3J_{C-F}$ = 3.7 Hz), 134.1 (q, $^3J_{C-F}$ = 3.7 Hz), 130.6 (q, $^2J_{C-F}$ = 33.4 Hz), 122.6 (q, $^1J_{C-F}$ = 273.2 Hz), 102.9, 50.2, 31.2;

^{19}F NMR (376 MHz, $CDCl_3$, ppm) δ -62.9;

Elemental analysis (%): calculated for $C_{11}H_{11}BrF_3IS$: C, 30.09; H, 2.53; S, 7.30; found: C, 30.02; H, 2.54; S, 7.32.

General procedure C for boronic acid S7 synthesis from iodide S4

Following a reported procedure[13], a flame-dried 20 mL pressure vial was flushed with a stream of argon and charged with iodide **S4** (1.0 equiv) and dry THF (3 mL per 1 mmol of iodide **S4**). The reaction mixture was cooled to −78 °C (dry ice/acetone bath) under argon atmosphere and iPrMgCl·LiCl solution in THF (1.3 M, 1.1 equiv) was added dropwise within 30 minutes. Then

the resulting yellow solution was stirred for 1 hour at −78 °C, whereupon trimethylborate (1.3 equiv) was added dropwise within 5 min. The stirring at −78 °C was continued for 1 hour, whereupon the white suspension was allowed to warm to room temperature. After stirring overnight, the reaction was quenched with 1 M HCl (15 mL) and extracted with Et_2O (3 × 50 mL). The combined organic extracts were dried over Na_2SO_4 and concentrated under reduced pressure. The resulting crude product was purified by reversed-phase column chromatography on C18 silica gel using a gradient elution from 15% MeCN in 0.1% TFA in water to 95% MeCN in 0.1% TFA in water, to afford an amorphous solid.

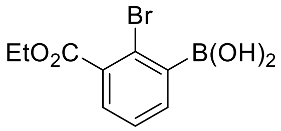

(2-Bromo-3-(ethoxycarbonyl)phenyl)boronic acid (S7a). Following general procedure C, iodide **S4a** (1.50 g, 4.27 mmol) was converted into **S7a**. Pale yellow amorphous solid (925 mg, 80% yield);

^{1}H NMR (300 MHz, DMSO-d_6, ppm) δ 7.58 – 7.54 (1H, m), 7.42 (1H, s), 7.40 (1H, d, J = 1.6 Hz), 4.31 (2H, q, J = 7.1 Hz), 1.31 (3H, t, J = 7.1 Hz);

^{13}C{^{1}H} NMR (75 MHz, DMSO-d_6, ppm) δ 166.9, 135.1, 133.5, 129.4, 126.8, 126.8, 121.9, 61.4, 14.1;

HRMS (ESI): m/z $[M+H]^+$ calculated for $C_9H_{11}BrBO_4^+$: 272.9934, found 272.9944.

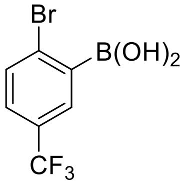

(2-Bromo-5-(trifluoromethyl)phenyl)boronic acid (S7b). Following general procedure C, iodide **S4j** (1,50 g, 4,23 mmol) was converted into **S7b**. White amorphous solid (920 mg, 80% yield);

^{1}H NMR (400 MHz, DMSO-d_6, ppm) δ 8.55 (2H, br s), 7.77 (1H, d, J = 8.3 Hz), 7.66 (1H, d, J = 2.4 Hz), 7.60 (1H, ddd, J = 8.3, 2.4, 0.8 Hz);

^{13}C{^{1}H} NMR (101 MHz, DMSO-d_6, ppm) δ 132.4, 129.8 (q, $^3J_{C\text{-}F}$ = 4.0 Hz), 129.6 (q, $^3J_{C\text{-}F}$ = 1.9 Hz), 127.3 (q, $^2J_{C\text{-}F}$ = 31.8 Hz), 126.7 (q, $^3J_{C\text{-}F}$ = 4.0 Hz), 122.9 (q, $^1J_{C\text{-}F}$ = 272.0 Hz);

^{19}F NMR (376 MHz, $CDCl_3$, ppm) δ -56.4;

HRMS (ESI): m/z $[M+H]^+$ calculated for $C_7H_4BBrF_3O_2$: 266.9440, found 266.9447.

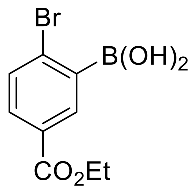

(2-Bromo-5-(ethoxycarbonyl)phenyl)boronic acid (S7c). Following general procedure C, iodide **S4h** (1.50 g, 4.23 mmol) was converted into **S7c**. White amorphous solid (656 mg, 57% yield);

^{1}H NMR (400 MHz, DMSO-d_6, ppm) δ 8.49 (2H, br s), 7.89 (1H, d, J = 2.3 Hz), 7.79 (1H, dd, J = 8.3, 2.3 Hz), 7.68 (1H, d, J = 8.3 Hz), 4.31 (2H, q, J = 7.1 Hz), 1.32 (3H, t, J = 7.1 Hz);

^{13}C{^{1}H} NMR (101 MHz, DMSO-d$_6$, ppm) δ 165.4, 134.0, 132.0, 130.7, 130.7, 128.0, 61.0, 14.2;

HRMS (ESI): m/z [M+H]$^+$ calculated for $C_9H_{11}O_4BrB^+$ 272.9934, found 272.9937.

Synthesis of ethyl 2-bromo-5-methoxy-3-(4,4,5,5-tetramethyl-1,3,2-dioxaborolan-2-yl)benzoate (S7e).

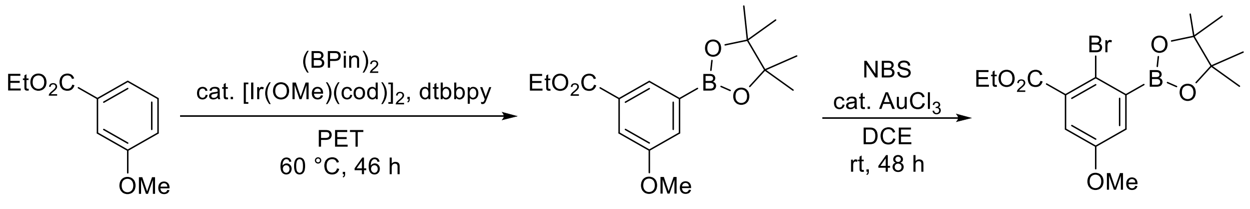

Following a reported procedure[14], a 20 mL pressure vial was charged with ester **S2c** (900 mg, 5.00 mmol, 1.0 equiv), [Ir(OMe)(cod)]$_2$ (65 mg, 0.10 mmol, 0.02 equiv), 4,4'-di-*tert*-butyl-2,2'-bipyridine (40 mg, 0.15 mmol, 0.03 equiv), bis(pinacolato)diboron (1.90 g, 7.49 mmol, 1.50 equiv), and *n*-hexane (5 mL). The reaction mixture was stirred for 46 hours at 60 °C, cooled to room temperature, and all volatiles were removed by distillation under reduced pressure. The resulting crude product was purified by flash chromatography using gradient elution from 5% EtOAc/petroleum ether to 40% EtOAc/petroleum ether. The afforded boronic acid pinacol ester **S7d** (white amorphous solid) was used in the further step without additional purification.

Following a reported procedure[15], the boronic acid pinacol ester **S7d** from above (600 mg, 1.96 mmol, 1 equiv), NBS (349 mg, 1.96 mmol, 1 equiv), and AuCl$_3$ (9 mg, 0.02 mmol, 0.01 equiv) were weighted in a 25 mL flask, then DCE (4 mL) was added. The reaction mixture was stirred at room temperature for 48 h. The solution was then concentrated under reduced pressure. The resulting crude product was purified by flash chromatography using gradient elution from 5% EtOAc/petroleum ether to 20% EtOAc/petroleum ether. Pale green viscous oil (371 mg, 30 % yield); analytical TLC on silica gel, 1:10 EtOAc/petroleum ether, R_f=0.30.

^{1}H NMR (300 MHz, $CDCl_3$, ppm) δ 8.05 (1H, dd, *J* = 1.5, 0.9 Hz), 7.66 (1H, dd, *J* = 2.8, 1.5 Hz), 7.51 (1H, dd, *J* = 2.8, 0.9 Hz), 4.38 (2H, q, *J* = 7.1 Hz), 3.87 (3H, s), 1.40 (3H, t, *J* = 7.1 Hz), 1.35 (12H, s);

^{13}C{^{1}H} NMR (75 MHz, $CDCl_3$, ppm) δ 167.2, 157.9, 135.2, 123.5, 117.5, 115.6, 84.73, 61.8, 55.8, 24.9, 14.3;

HRMS (ESI): m/z $[M+H]^+$ calculated for $C_{16}H_{23}O_5BrB^+$ 385.0822, found 385.0812.

General procedure D for trimethylstannane S8 synthesis from iodide S4

A flame-dried 20 mL pressure vial was flushed with a stream of argon and charged with iodide **S4** (1.0 equiv) and dry THF (2 mL per 1 mmol of iodide **S4**). The reaction mixture was cooled to −40 °C (dry ice/acetone bath) under argon atmosphere and iPrMgCl·LiCl solution in THF (1.3 M, 1.2 equiv) was added dropwise within 30 minutes. Then the resulting colorless solution was stirred for 1 hour at −40 °C, whereupon a solution of trimethyltin chloride (1.5 equiv) in dry THF (0.5 mL per 1 mmol of trimethyltin chloride) was added dropwise within 20 min. The pale-yellow reaction mixture was stirred at −78 °C for 1 hour, whereupon it was allowed to warm to room temperature. After stirring overnight, all volatiles were removed by distillation under reduced pressure. The resulting crude product was purified by reversed phase column chromatography on C18 silica gel using gradient elution from 50% MeCN in water to 95% MeCN in water, to afford an oil.

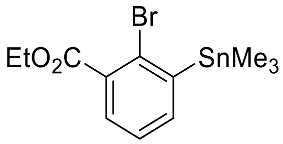

Ethyl 2-bromo-3-(trimethylstannyl)benzoate (S8a).

Following general procedure D, iodide **S4a** (2.00 g, 5.63 mmol) was converted into **S8a**. Colorless oil (1.20 g, 54% yield);

^{1}H NMR (400 MHz, $CDCl_3$, ppm) δ 7.59 (1H, dd, *J* = 7.3, 1.8 Hz), 7.42 (1H, dd, *J* = 7.3, 1.8 Hz), 7.31 (1H, t, *J* = 7.3 Hz), 4.39 (2H, q, *J* = 7.1 Hz), 1.40 (3H, t, *J* = 7.1 Hz), 0.40 (9H, s);

^{13}C{^{1}H} NMR (101 MHz, $CDCl_3$, ppm) δ 167.6, 149.9, 139.6, 133.5, 130.8, 130.4, 126.5, 61.8, 14.3, -7.3;

HRMS (ESI): m/z $[M+H]^+$ calculated for $C_{12}H_{18}O_2Br_2Sn^+$ 392.9512, found 392.9510.

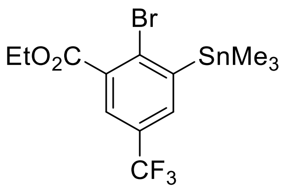

Ethyl 2-bromo-5-(trifluoromethyl)-3-(trimethylstannyl)benzoate (S8b).

Following general procedure D, iodide **S4f** (500 mg, 2.51 mmol) was converted into **S8b**. Colorless oil (375 mg, 33% yield);

^{1}H NMR (400 MHz, $CDCl_3$, ppm) δ 7.62 (1H, dd, J = 2.4, 0.7 Hz), 7.83 (1H, dd, J = 2.4, 0.7 Hz)., 4.42 (2H, q, J = 7.1 Hz), 1.42 (3H, t, J = 7.1 Hz), 0.45 (9H, s);

^{13}C{^{1}H} NMR (101 MHz, $CDCl_3$, ppm) δ 166.3, 151.9, 135.5 (q, $^3J_{C\text{-}F}$ = 3.7 Hz), 134.3, 133.8, 129.0 (q, $^2J_{C\text{-}F}$ = 32.7 Hz), 127.8 (q, $^3J_{C\text{-}F}$ = 3.7 Hz), 123.9 (q, $^1J_{C\text{-}F}$ = 272.8 Hz), 62.3, 14.3, -7.2;

^{19}F NMR (376 MHz, $CDCl_3$, ppm) δ -62.7;

HRMS (ESI): m/z $[M+H]^+$ calculated for $C_{13}H_{17}O_2F_3BrSn^+$ 460.9386, found 460.9376.

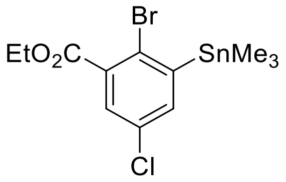

Ethyl 2-bromo-5-chloro-3-(trimethylstannyl)benzoate (S8c).

Following general procedure D, iodide **S4d** (1.00 g, 2.57 mmol) was converted into **S8c**. Colorless oil (416 mg, 38% yield);

^{1}H NMR (400 MHz, $CDCl_3$, ppm) δ 7.58 – 7.56 (1H, m), 7.36 – 7.34 (1H, m), 4.39 (2H, q, J = 7.2 Hz), 1.40 (3H, t, J = 7.2 Hz), 0.42 (9H, s);

^{13}C{^{1}H} NMR (101 MHz, $CDCl_3$, ppm) δ 166.2, 152.4, 139.0, 134.5, 133.3, 130.7, 128.1, 62.1, 14.3, -7.2;

HRMS (ESI): m/z $[M+H]^+$ calculated for $C_{12}H_{17}O_2ClBrSn^+$ 426.9122, found 426.9101.

General procedure E for Suzuki–Miyaura reaction

A flame-dried 20 mL pressure vial was flushed with a stream of argon and charged with iodide **S4** (1.0 equiv), boronic acid or ester **S7** (2.0 equiv), $PdCl_2$(dppf) (0.05 equiv), CsF (3 equiv), and dry dioxane (10 mL per 1 mmol of iodide **S4**). The reaction mixture was stirred for 3 hours at 80 °C under argon atmosphere, whereupon it was cooled to room temperature. H_2O (30 mL) was added to the reaction mixture, and the resulting white suspension was extracted with EtOAc (3 × 30 mL). The combined organic extracts were dried over Na_2SO_4, and concentrated under reduced pressure. The resulting crude product was purified by flash chromatography to afford an oil.

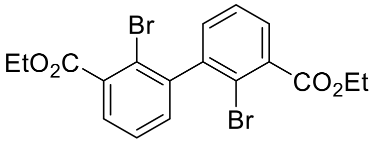

Diethyl 2,2'-dibromo-[1,1'-biphenyl]-3,3'-dicarboxylate (4a). Following general procedure E, iodide **S4a** (300 mg, 0.845 mmol) and boronic acid **S7a** (461 mg, 1.69 mmol) were converted into **4a**. The resulting crude product was purified by flash chromatography using gradient elution from 100% petroleum ether to 30% Et_2O/petroleum ether. Pale yellow viscous oil (230 mg, 60% yield); analytical TLC on silica gel, 1:8 Et_2O/petroleum ether, R_f=0.28.

^{1}H NMR (400 MHz, $CDCl_3$, ppm) δ 7.71 (2H, dd, J = 7.6, 1.8 Hz), 7.44 (2H, t, J = 7.6 Hz), 7.32 (2H, dd, J = 7.6, 1.8 Hz), 4.43 (4H, q, J = 7.2 Hz), 1.42 (6H, t, J = 7.2 Hz);

^{13}C{^{1}H} NMR (101 MHz, $CDCl_3$, ppm) δ 167.0, 143.9, 134.6, 133.3, 130.2, 127.2, 122.1, 62.0, 14.4;

HRMS (ESI): m/z $[M+H]^+$ calculated for $C_{18}H_{17}Br_2O_4$: 454.9494, found 454.9501.

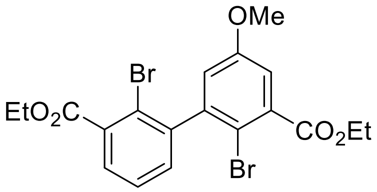

Diethyl 2,2'-dibromo-5-methoxy-[1,1'-biphenyl]-3,3'-dicarboxylate (4h).

Following general procedure E, iodide **S4a** (50 mg, 0.14 mmol) and boronic acid ester **S7e** (108 mg, 0.281 mmol) were converted into **4h**. The resulting crude product was purified by reversed phase column chromatography on C18 silica gel using gradient elution from 25% MeCN in 0.1% TFA in water to 95% MeCN in 0.1% TFA. Pale yellow oil (33 mg, 48% yield).

^{1}H NMR (400 MHz, $CDCl_3$, ppm) δ 7.70 (1H, dd, J = 7.7, 1.8 Hz), 7.43 (1H, t, J = 7.7 Hz), 7.31 (1H, dd, J = 7.7, 1.8 Hz), 7.25 (1H, d, J = 3.1 Hz), 6.87 (1H, d, J = 3.1 Hz), 4.46 – 4.38 (4H, m), 3.83 (3H, s), 1.44 – 1.39 (6H, m);

^{13}C{^{1}H} NMR (101 MHz, $CDCl_3$, ppm) δ 167.0, 166.9, 158.3, 144.6, 143.9, 135.2, 134.6, 133.2, 130.2, 127.2, 122.0, 119.1, 115.9, 112.4, 62.1, 62.0, 55.9, 14.3;

HRMS (ESI): m/z $[M+H]^+$ calculated for $C_{19}H_{19}Br_2O_5$: 484.9599, found 484.9598.

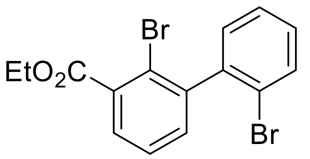

Ethyl 2,2'-dibromo-[1,1'-biphenyl]-3-carboxylate (4i). Following general procedure E, iodide **S4a** (300 mg, 0.845 mmol) and (2-bromo-3-(ethoxycarbonyl)phenyl)boronic acid **S7e** (339 mg, 1.69 mmol) were converted into **4i**. The resulting crude product was

purified by reversed phase column chromatography on C18 silica gel using gradient elution from 15% MeCN in 0.1% TFA in water to 95% MeCN in 0.1% TFA. Black oil (180 mg, 55% yield).

^{1}H NMR (300 MHz, $CDCl_3$, ppm) δ 7.74 – 7.63 (2H, m), 7.46 – 7.20 (5H, m), 4.43 (2H, q, *J* = 7.2 Hz), 1.42 (3H, t, *J* = 7.2 Hz);

^{13}C{^{1}H} NMR (75 MHz, $CDCl_3$, ppm) δ 167.1, 144.0, 142.1, 134.6, 133.3, 132.8, 131.1, 130.0, 129.7, 127.3, 127.1, 123.6, 122.1, 62.0, 14.4;

HRMS (ESI): m/z $[M+H]^+$ calculated for $C_{15}H_{13}Br_2O_2$: 382.9282, found 382.9280.

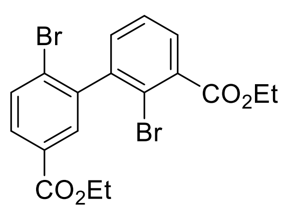

Diethyl 2,6'-dibromo-[1,1'-biphenyl]-3,3'-dicarboxylate (4j).

Following general procedure E, iodide **S4a** (400 mg, 1.13 mmol) and boronic acid **S7c** (615 mg, 2.25 mmol) were converted into **4j**. The resulting crude product was purified by flash chromatography using gradient elution from 100% petroleum ether to 30% Et_2O/petroleum ether. Colorless oil (233 mg, 45% yield), analytical TLC on silica gel, 1:8 Et_2O/petroleum ether, R_f=0.35.

^{1}H NMR (400 MHz, $CDCl_3$, ppm) δ 7.93 (1H, dd, *J* = 8.3, 2.1 Hz), 7.90 (1H, d, *J* = 2.1 Hz), 7.75 (1H, d, *J* = 8.3 Hz), 7.72 (1H, dd, *J* = 7.7, 1.8 Hz), 7.45 (1H, t, *J* = 7.7 Hz), 7.33 (1H, dd, *J* = 7.7, 1.8 Hz), 4.43 (2H, q, *J* = 7.1 Hz), 4.37 (2H, q, *J* = 7.1 Hz), 1.42 (3H, t, *J* = 7.1 Hz), 1.38 (3H, t, *J* = 7.1 Hz);

^{13}C{^{1}H} NMR (101 MHz, $CDCl_3$, ppm) δ 166.9, 165.8, 143.2, 142.3, 134.7, 133.2, 133.0, 132.0, 130.6, 130.4, 129.9, 129.0, 127.2, 122.1, 62.0, 61.5, 14.4, 14.3;

HRMS (ESI): m/z $[M+H]^+$ calculated for $C_{18}H_{17}Br_2O_4$: 454.9494, found 454.9490.

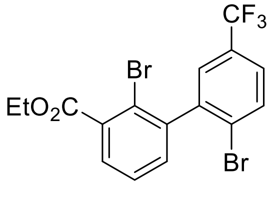

Ethyl 2,2'-dibromo-5'-(trifluoromethyl)-[1,1'-biphenyl]-3-carboxylate (4k).

Following general procedure E, iodide **S4a** (300 mg, 0.845 mmol) and boronic acid **S7b** (454 mg, 1.69 mmol) were converted into **4c**. The resulting crude product was purified by reversed phase column chromatography on C18 silica gel using gradient elution from 25% MeCN in 0.1% TFA in water to 95% MeCN in 0.1% TFA. Black oil (121 mg, 32% yield).

^{1}H NMR (400 MHz, $CDCl_3$, ppm) δ 7.81 (1H, d, *J* = 8.3 Hz), 7.74 (1H, dd, *J* = 7.7, 1.8 Hz), 7.58 – 7.47 (2H, m), 7.46 (1H, t, *J* = 7.7 Hz), 7.33 (1H, dd, *J* = 7.7, 1.8 Hz), 4.43 (2H, q, *J* = 7.1 Hz), 1.42 (3H, t, *J* = 7.1 Hz);

^{13}C{^{1}H} NMR (101 MHz, $CDCl_3$, ppm) δ 166.8, 142.8, 142.6, 134.8, 133.5, 133.1, 130.6, 130.0 (q,$^2J_{C\text{-}F}$ = 33.1 Hz), 128.0 (q, $^3J_{C\text{-}F}$ = 3.7 Hz), 127.8, 127.3, 126.4 (q, $^3J_{C\text{-}F}$ = 3.6 Hz), 123.8 (q, $^1J_{C\text{-}F}$ = 272.3 Hz), 121.9, 62.1, 14.4;

^{19}F NMR (376 MHz, $CDCl_3$, ppm) δ -62.6.

HRMS (ESI): m/z $[M+H]^+$ calculated for $C_{16}H_{12}Br_2F_3O_2$: 450.9156, found 450.9157.

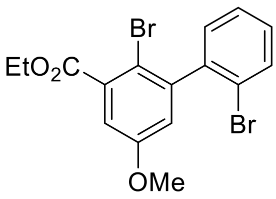

Ethyl 2,2'-dibromo-5-methoxy-[1,1'-biphenyl]-3-carboxylate (S9a). Following general procedure E, 1-bromo-2-iodobenzene **S4n** (200 mg, 0.707 mmol) and boronic acid ester **S7e** (544 mg, 1.42 mmol) were converted into **S9a**. The resulting crude product was purified by flash chromatography using gradient elution from 100% petroleum ether to 30% Et_2O/petroleum ether. Pale green oil (126 mg, 43% yield), analytical TLC on silica gel, 1:8 Et_2O/petroleum ether, R_f=0.35.

^{1}H NMR (400 MHz, $CDCl_3$, ppm) δ 7.67 (1H, dd, *J* = 7.7, 1.2 Hz), 7.38 (1H, td, *J* = 7.7, 1.2 Hz), 7.31 – 7.19 (3H, m), 6.89 (1H, d, *J* = 3.1 Hz), 4.42 (2H, q, *J* = 7.2 Hz), 3.84 (3H, s), 1.42 (3H, t, *J* = 7.2 Hz);

^{13}C{^{1}H} NMR (101 MHz, $CDCl_3$, ppm) δ 167.0, 158.2, 144.7, 142.1, 135.2, 132.8, 131.0, 129.7, 127.3, 123.5, 119.2, 115.7, 112.5, 62.1, 55.9, 14.3;

HRMS (ESI): m/z $[M+H]^+$ calculated for $C_{16}H_{15}Br_2O_3$: 412.9378, found 412.9388.

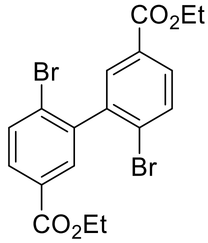

Diethyl 6,6'-dibromo-[1,1'-biphenyl]-3,3'-dicarboxylate (**S9b).** Following general procedure E, iodide **S4h** (319 mg, 0.90 mmol) and boronic acid **S7c** (491 mg, 1.80 mmol) were converted into **S9b**. The resulting crude product was purified by flash chromatography using gradient elution from 10% EtOAc/petroleum ether to 40% EtOAc/petroleum ether. Colorless powder (277 mg, 68% yield), analytical TLC on silica gel, 1:10 Et_2O/petroleum ether, R_f=0.25.

^{1}H NMR (400 MHz, $CDCl_3$, ppm) δ 7.95 (2H, dd, *J* = 8.3, 2.1 Hz), 7.91 (2H, d, *J* = 2.1 Hz), 7.76 (2H, d, *J* = 8.3 Hz), 4.38 (4H, q, *J* = 7.1 Hz), 1.39 (6H, t, *J* = 7.1 Hz);

$^{13}C\{^1H\}$ NMR (101 MHz, $CDCl_3$, ppm) δ 165.8, 141.6, 133.0, 131.9, 130.7, 130.0, 129.0, 61.6, 14.4;

HRMS (ESI): m/z $[M+H]^+$ calculated for $C_{18}H_{17}O_4Br_2^+$ 454.9494, found 454.9500.

General procedure F for Stille reaction

Following a reported procedure[16], a flame-dried 20 mL pressure vial was flushed with a stream of argon and charged with iodide **S4** (1.0 equiv), trimethylstannane **S8** (1.0 equiv), CuI (0.75 equiv), dry DMF (15 mL per 1 mmol of iodide **S4**), and $Pd_2(dba)_3$ (0.10 equiv) with PPh_3 (0.40 equiv) or $Pd(PPh_3)_4$ (0.10 equiv). The reaction mixture was stirred overnight at 50 °C under argon atmosphere, whereupon it was cooled to room temperature. H_2O (30 mL) was added to the reaction mixture, and the resulting white suspension was extracted with Et_2O (3 × 30 mL). The combined organic extracts were dried over Na_2SO_4, and concentrated under reduced pressure. The resulting crude product was purified by flash chromatography to afford an oil.

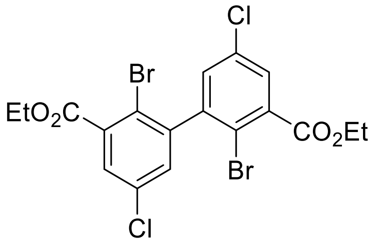

Diethyl 2,2'-dibromo-5,5'-dichloro-[1,1'-biphenyl]-3,3'-dicarboxylate (4b).

Following general procedure F and using $Pd(PPh_3)_4$ (173 mg, 0.15 mmol), iodide **S4d** (584 mg, 1.5 mmol) and trimethylstannane **S8c** (639 mg, 1.5 mmol) were converted into **4b**. The resulting crude product was purified by flash chromatography using gradient elution from 100% petroleum ether to 30% Et_2O/petroleum ether, then by reversed phase column chromatography on C18 silica gel using gradient elution from 30% MeCN in 0.1% TFA in water to 95% MeCN in 0.1% TFA. Colorless oil (329 mg, 42% yield), analytical TLC on silica gel, 1:10 EtOAc/petroleum ether, R_f=0.31.

^1H NMR (400 MHz, $CDCl_3$, ppm) δ 7.72 (2H, d, J = 2.6 Hz), 7.30 (2H, d, J = 2.6 Hz), 4.42 (4H, q, J = 7.2 Hz), 1.42 (6H, t, J = 7.2 Hz);

$^{13}C\{^1H\}$ NMR (101 MHz, $CDCl_3$, ppm) δ 165.5, 144.1, 135.8, 133.5, 132.9, 130.6, 120.2, 62.5, 14.3;

HRMS (ESI): m/z $[M+H]^+$ calculated for $C_{18}H_{15}O_4Cl_2Br_2^+$ 522.8714, found 522.8704.

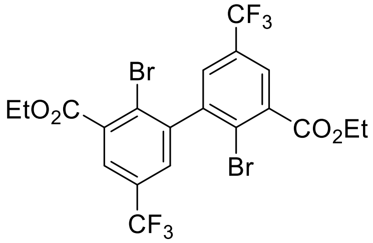

Diethyl 2,2'-dibromo-5,5'-bis(trifluoromethyl)-[1,1'-biphenyl]-3,3'-dicarboxylate (4c).

Following general procedure F and using $Pd(PPh_3)_4$ (75 mg, 0.07 mmol), iodide **S4f** (275 mg, 0.65 mmol), and trimethylstannane **S8b** (299 mg, 0.65 mmol) were converted into **4c**. The resulting crude product was purified by flash chromatography using gradient elution from 100% petroleum ether to 30% Et_2O/petroleum ether, then by reversed phase column chromatography on C18 silica gel using gradient elution from 30% MeCN in 0.1% TFA in water to 95% MeCN in 0.1% TFA. Colorless oil (298 mg, 77% yield), analytical TLC on silica gel, 1:10 EtOAc/petroleum ether, R_f=0.29.

^{1}H NMR (400 MHz, $CDCl_3$, ppm) δ 7.58 (2H, dd, J = 2.3, 0.7 Hz), 8.01 (2H, dd, J = 2.3, 0.7 Hz), 4.46 (4H, q, J = 7.1 Hz), 1.44 (6H, t, J = 7.1 Hz);

^{13}C{^{1}H} NMR (101 MHz, $CDCl_3$, ppm) δ 165.4, 143.6, 135.7, 130.2 (q, $^2J_{C\text{-}F}$ = 33.9 Hz), 129.6 (q, $^3J_{C\text{-}F}$ = 3.7 Hz), 127.7 (q, $^3J_{C\text{-}F}$ = 3.7 Hz), 126.3, 123.2 (q, $^1J_{C\text{-}F}$ = 272.9 Hz), 62.7, 14.3;

^{19}F NMR (376 MHz, $CDCl_3$, ppm) δ -62.8;

HRMS (ESI): m/z $[M+H]^+$ calculated for $C_{20}H_{15}O_4F_6Br_2^+$ 590.9241, found 590.9233.

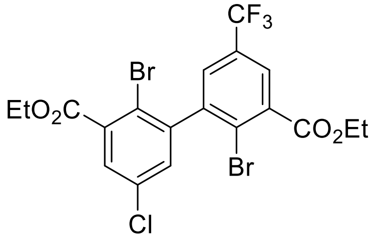

Diethyl 2,2'-dibromo-5-chloro-5'-(trifluoromethyl)-[1,1'-biphenyl]-3,3'-dicarboxylate (4e).

Following general procedure F and using $Pd_2(dba)_3$ (130 mg, 0.142 mmol) and PPh_3 (149 mg, 0.567 mmol), iodide **S4f** (600 mg, 1.42 mmol), and trimethylstannane **S8c** (605 mg, 1.42 mmol) were converted into **4e**. The resulting crude product was purified by flash chromatography using gradient elution from 100% petroleum ether to 30% Et_2O/petroleum ether, then by reversed phase column chromatography on C18 silica gel using gradient elution from 30% MeCN in 0.1% TFA in water to 95% MeCN in 0.1% TFA. Pale brown oil (429 mg, 54% yield), analytical TLC on silica gel, 1:8 Et_2O/petroleum ether, R_f=0.39. Analytically pure material was obtained by preparative HPLC, using Chiralpak IG column and 20% DCM in heptane elution.

^{1}H NMR (400 MHz, $CDCl_3$, ppm) δ 7.98 (1H, dd, J = 2.5, 0.7 Hz), 7.75 (1H, d, J = 2.5 Hz), 7.56 (1H, dd, J = 2.5, 0.7 Hz), 7.33 (1H, d, J = 2.5 Hz), 4.49 – 4.40 (4H, m), 1.46 – 1.39 (6H, m);

$^{13}C\{^1H\}$ NMR (101 MHz, $CDCl_3$, ppm) δ 165.5, 165.4, 144.0, 143.8, 135.9, 135.5, 133.6, 132.9, 130.9, 130.1 (q, $^2J_{C\text{-}F}$ = 33.9 Hz), 129.6 (q, $^3J_{C\text{-}F}$ = 3.6 Hz), 127.5 (q, $^3J_{C\text{-}F}$ = 3.7 Hz), 126.3 (q, $^3J_{C\text{-}F}$ = 1.6 Hz), 123.2 (q, $^1J_{C\text{-}F}$ = 272.8 Hz), 120.2, 62.7, 62.5, 14.3;
^{19}F NMR (376 MHz, $CDCl_3$, ppm) δ -62.8;
HRMS (ESI): m/z $[M+H]^+$ calculated for $C_{19}H_{15}O_4F_3ClBr_2^+$ 556.8978, found 556.8992.

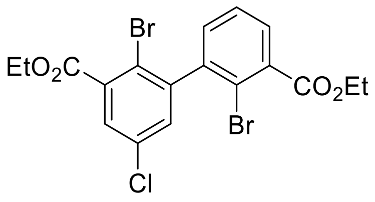

Diethyl 2,2'-dibromo-5-chloro-[1,1'-biphenyl]-3,3'-dicarboxylate (4f).

Following general procedure F and using $Pd_2(dba)_3$ (35 mg, 0.039 mmol) and PPh_3 (40 mg, 0.15 mmol), iodide **S4d** (150 mg, 0.385 mmol) and trimethylstannane **S8a** (151 mg, 0.385 mmol) were converted into **4f**. The resulting crude product was purified by flash chromatography using gradient elution from 100% petroleum ether to 30% Et_2O/petroleum ether, then by reversed phase column chromatography on C18 silica gel using gradient elution from 30% MeCN in 0.1% TFA in water to 95% MeCN in 0.1% TFA. Colorless oil (67 mg, 36% yield), analytical TLC on silica gel, 1:8 Et_2O/petroleum ether, R_f=0.28.
^1H NMR (400 MHz, $CDCl_3$, ppm) δ 7.73 (1H, dd, *J* = 7.7, 1.8 Hz), 7.70 (1H, d, *J* = 2.6 Hz), 7.44 (1H, t, *J* = 7.7 Hz), 7.32 (1H, d, *J* = 2.6 Hz), 7.29 (1H, dd, *J* = 7.7, 1.8 Hz), 4.46 – 4.39 (4H, m), 1.44 – 1.39 (6H, m);
$^{13}C\{^1H\}$ NMR (101 MHz, $CDCl_3$, ppm) δ 166.8, 165.7, 145.3, 142.7, 135.7, 134.7, 133.3, 133.1, 133.1, 130.6, 130.2, 127.3, 121.9, 120.4, 62.4, 62.1, 14.3, 14.3;
HRMS (ESI): m/z $[M+H]^+$ calculated for $C_{18}H_{16}O_4ClBr_2^+$ 488.9104, found 488.9104.

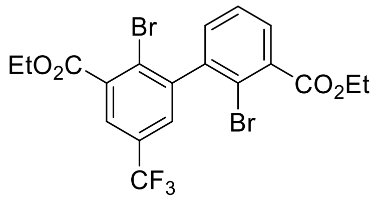

Diethyl 2,2'-dibromo-5-(trifluoromethyl)-[1,1'-biphenyl]-3,3'-dicarboxylate (4g).

Following general procedure F and using $Pd_2(dba)_3$ (65 mg, 0.072 mmol) and PPh_3 (75 mg, 0.29 mmol), iodide **S4f** (280 mg, 0.715 mmol) and trimethylstannane **S8a** (302 mg, 0.715 mmol) were converted into **4g**. The resulting crude product was purified by flash chromatography using gradient elution from 100% petroleum ether to 30% Et_2O/petroleum ether, then by reversed phase column chromatography on C18 silica gel using gradient elution from 30% MeCN in 0.1% TFA in water to 95% MeCN in

0.1% TFA. Colorless oil (189 mg, 51% yield), analytical TLC on silica gel, 1:8 Et_2O/petroleum ether, R_f=0.34.

^{1}H NMR (400 MHz, $CDCl_3$, ppm) δ 7.96 (1H, d, J = 2.0 Hz), 7.76 (1H, dd, J = 7.7, 2.0 Hz), 7.57 (1H, d, J = 2.0 Hz), 7.47 (1H, t, J = 7.7 Hz), 7.32 (1H, dd, J = 7.7, 2.0 Hz), 4.53 – 4.37 (4H, m), 1.49 – 1.37 (6H, m);

^{13}C{^{1}H} NMR (101 MHz, $CDCl_3$, ppm) δ 166.7, 165.7, 144.9, 142.6, 135.4, 134.8, 133.1, 130.8, 130.2 (q, $^2J_{C-F}$ = 33.6 Hz), 129.8 (q, $^3J_{C-F}$ = 3.5 Hz), 127.4, 127.0 (q, $^3J_{C-F}$ = 3.5 Hz), 126.5, 123.3 (q, $^1J_{C-F}$ = 272.8 Hz), 121.9, 62.6, 62.1, 14.3, 14.3;

^{19}F NMR (376 MHz, $CDCl_3$, ppm) δ -62.8;

HRMS (ESI): m/z $[M+H]^+$ calculated for $C_{19}H_{16}O_4F_3Br_2^+$ 522.9367, found 522.9374.

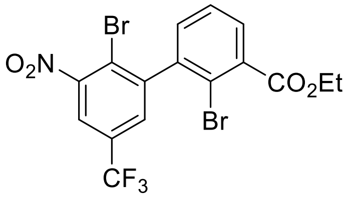

Ethyl 2,2'-dibromo-3'-nitro-5'-(trifluoromethyl)-[1,1'-biphenyl]-3-carboxylate (4l).

Following general procedure F and using $Pd_2(dba)_3$ (69 mg, 0.076 mmol) and PPh_3 (80 mg, 0.30 mmol), iodide **S4g** (300 mg, 0.758 mmol) and trimethylstannane **S8a** (297 mg, 0.758 mmol) were converted into **4l**. The resulting crude product was purified by flash chromatography using gradient elution from 100% petroleum ether to 30% Et_2O/petroleum ether, then by reversed phase column chromatography on C18 silica gel using gradient elution from 30% MeCN in 0.1% TFA in water to 95% MeCN in 0.1% TFA. Yellow oil (120 mg, 32% yield), analytical TLC on silica gel, 1:8 Et_2O/petroleum ether, R_f=0.30.

^{1}H NMR (400 MHz, $CDCl_3$, ppm) δ 8.03 (1H, d, J = 1.9 Hz), 7.81 (1H, dd, J = 7.7, 1.9 Hz), 7.69 (1H, d, J = 1.9 Hz), 7.51 (1H, t, J = 7.7 Hz), 7.34 (1H, dd, J = 7.7, 1.9 Hz), 4.44 (2H, q, J = 7.1 Hz), 1.43 (3H, t, J = 7.1 Hz);

^{13}C{^{1}H} NMR (101 MHz, $CDCl_3$, ppm) δ 166.4, 151.5, 146.4, 141.4, 135.0, 132.8, 131.5, 131.0 (q, $^2J_{C-F}$ = 34.9 Hz), 130.5 (q, $^3J_{C-F}$ = 3.6 Hz), 127.7, 122.6 (q, $^1J_{C-F}$ = 273.2 Hz), 121.8 (q, $^3J_{C-F}$ = 3.6 Hz), 121.7, 120.1, 62.3, 14.3;

^{19}F NMR (376 MHz, $CDCl_3$, ppm) δ -62.9;

HRMS (ESI): m/z $[M+H]^+$ calculated for $C_{16}H_{11}NO_4F_3Br_2^+$ 495.9007, found 495.9002.

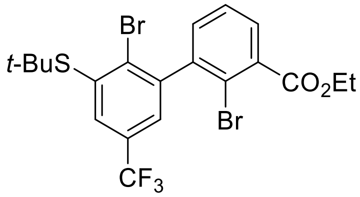

Ethyl 2,2'-dibromo-3'-(*tert*-butylthio)-5'-(trifluoromethyl)-[1,1'-biphenyl]-3-carboxylate (S9c).

Following general procedure F and using $Pd_2(dba)_3$ (63 mg, 0.068 mmol) and PPh_3 (72 mg, 0.27 mmol), iodide **S4m** (300 mg, 0.683 mmol) and trimethylstannane **S8a** (268 mg, 0.683 mmol) were converted into **S9c**. The resulting crude product was purified by flash chromatography using gradient elution from 100% petroleum ether to 20% Et_2O/petroleum ether, then by reversed phase column chromatography on C18 silica gel using gradient elution from 30% MeCN in 0.1% TFA in water to 95% MeCN in 0.1% TFA. White amorphous solid (125 mg, 34% yield), analytical TLC on silica gel, 1:10 Et_2O/petroleum ether, R_f=0.40.

^{1}H NMR (400 MHz, $CDCl_3$, ppm) δ 7.95 (1H, d, J = 1.7 Hz), 7.74 (1H, dd, J = 7.7, 1.7 Hz), 7.46 (1H, t, J = 7.7 Hz), 7.43 (1H, d, J = 1.7 Hz), 7.33 (1H, dd, J = 7.7, 1.7 Hz), 4.43 (2H, q, J = 7.1 Hz), 1.44 – 1.37 (12H, m);

^{13}C{^{1}H} NMR (101 MHz, $CDCl_3$, ppm) δ 166.8, 144.6, 143.7, 137.7, 137.3, 134.8 (q, $^3J_{C\text{-}F}$ = 3.6 Hz), 134.7, 132.8, 130.6, 129.4 (q, $^2J_{C\text{-}F}$ = 33.2 Hz), 127.5 (q, $^3J_{C\text{-}F}$ = 3.6 Hz), 127.4, 123.5 (q, $^1J_{C\text{-}F}$ = 272.8 Hz), 121.9, 62.1, 49.78, 31.2, 14.33;

^{19}F NMR (376 MHz, $CDCl_3$, ppm) δ -62.7;

HRMS (ESI): m/z $[M+H]^+$ calculated for $C_{20}H_{20}O_2F_3SBr_2^+$ 538.9503, found 538.9497.

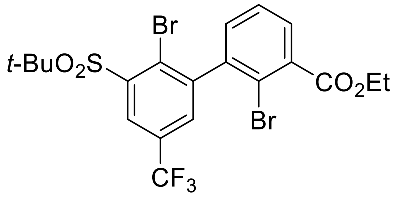

Ethyl 2,2'-dibromo-3'-(*tert*-butylsulfonyl)-5'-(trifluoromethyl)-[1,1'-biphenyl]-3-carboxylate (4m).

Following a reported procedure[17], to a 8 mL pressure vial was added *tert*-butyl sulfide **S9c** (108 mg, 0.200 mmol, 1.00 equiv) and Oxone® (492 mg, 0.800 mmol, 4.00 equiv). The mixture was suspended in 1:1 acetone/water (4.0 mL) and stirred at 50 °C overnight. The white suspension was diluted with water and DCM, then transferred to a separating funnel. The combined organic extracts were dried over Na_2SO_4, and concentrated under reduced pressure. The resulting crude product was purified by flash chromatography using gradient elution from 100% petroleum ether to 40% Et_2O/petroleum ether. White amorphous solid (48 mg, 42% yield), analytical TLC on silica gel, 1:5 Et_2O/petroleum ether, R_f=0.30.

^{1}H NMR (400 MHz, $CDCl_3$, ppm) δ 8.40 (1H, dd, *J* = 2.3, 0.7 Hz), 7.80 (1H, dd, *J* = 7.7, 1.7 Hz), 7.69 (1H, dd, *J* = 2.3, 0.7 Hz), 7.50 (1H, t, *J* = 7.7 Hz), 7.33 (1H, dd, *J* = 7.7, 1.7 Hz), 4.43 (2H, q, *J* = 7.1 Hz), 1.46 (9H, s), 1.42 (3H, t, *J* = 7.1 Hz);
^{13}C{^{1}H} NMR (101 MHz, $CDCl_3$, ppm) δ 166.4, 147.0, 142.5, 137.5, 134.7 132.8, 132.0 (q, $^{3}J_{C\text{-}F}$ = 3.6 Hz), 131.7 (q, $^{3}J_{C\text{-}F}$ = 3.6 Hz), 131.2, 130.2 (q, $^{2}J_{C\text{-}F}$ = 34.3 Hz), 128.9, 127.7, 123.0 (q, $^{1}J_{C\text{-}F}$ = 273.1 Hz), 121.9, 63.6, 62.2, 24.2, 14.3;
^{19}F NMR (376 MHz, $CDCl_3$, ppm) δ -62.8;
HRMS (ESI): m/z $[M+H]^+$ calculated for $C_{20}H_{20}O_4F_3SBr_2^+$ 570.9401, found 570.9374.

Synthesis of diethyl 2,2'-dibromo-5,5'-dimethoxy-[1,1'-biphenyl]-3,3'-dicarboxylate (4d).

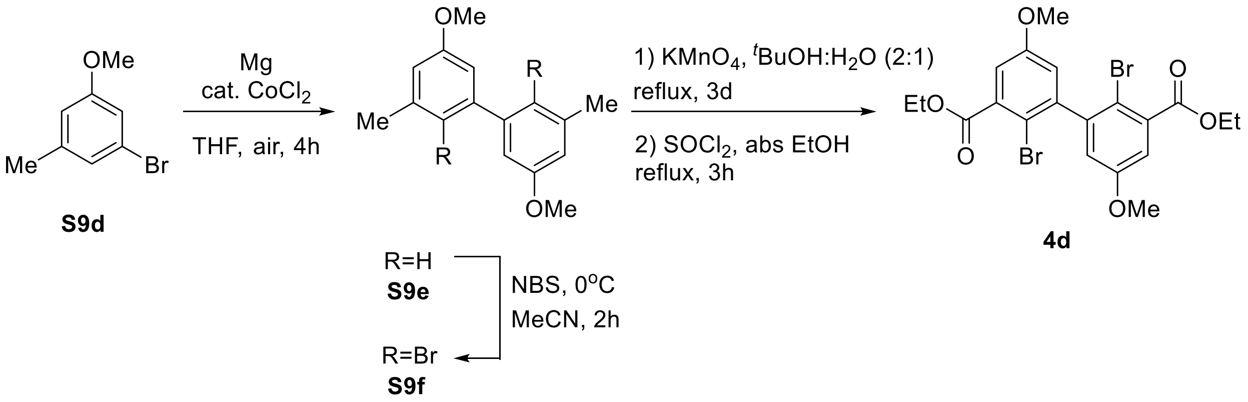

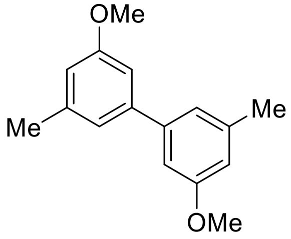

3,3'-Dimethoxy-5,5'-dimethyl-1,1'-biphenyl (S9e).
Following a reported procedure[18], in a flame-dried round-bottomed flask anhydrous $CoCl_2$ (130 mg, 1.00 mmol, 0.05 equiv) and magnesium turnings (632 mg, 24.00. mmol, 1.2 equiv) were suspended in 40 mL of anhydrous THF, followed by addition of 1-bromo-3-methoxy-5-methylbenzene (**S9d**, 4.02 g, 20.00 mmol, 1.0 equiv). The flask was closed with a septum and the blue suspension was stirred under a stream of dry air for 4 h, whereupon a dark suspension was formed. The reaction was quenched with 30 mL of 0.5 M HCl and extracted with EtOAc (3 × 30 mL). The combined organic extracts were dried over Na_2SO_4, and concentrated under reduced pressure. The resulting crude product was purified by flash chromatography using gradient elution from 5% EtOAc/petroleum ether to 15% EtOAc /petroleum ether. Colorless oil (1.6 g, 66% yield), analytical TLC on silica gel, 1:10 EtOAc/petroleum ether, R_f=0.35.

^{1}H NMR (400 MHz, $CDCl_3$, ppm) δ 7.01 – 6.99 (2H, m), 6.94 – 6.91 (2H, m), 6.75 – 6.71 (2H, m), 3.85 (6H, s), 2.42 – 2.39 (6H, m);
^{13}C{^{1}H} NMR (101 MHz, $CDCl_3$, ppm) δ 160.0, 142.8, 139.8, 120.8, 113.8, 110.1, 55.4, 21.8;
HRMS (ESI): m/z $[M+H]^+$ calculated for $C_{16}H_{19}O_2^+$: 243.1385, found 243.1386.

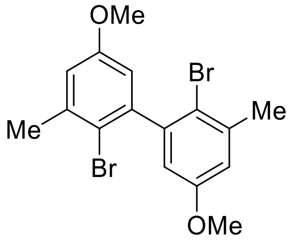

2,2'-Dibromo-5,5'-dimethoxy-3,3'-dimethyl-1,1'-biphenyl (S9f).
Following a reported procedure[19], to a solution of biaryl **S9e** (1.77 g, 7.3 mmol, 1.0 equiv) in MeCN (10 mL) a solution of NBS (2.86 g, 16.1 mmol, 2.2 equiv) in MeCN (20 mL) was added dropwise within 10 min at 0 °C. The resulting pale yellow solution was stirred for 2 h at the same temperature, whereupon colorless sediments were formed. The reaction suspension was quenched with water (50 mL) and extracted with Et_2O (3 × 30 mL). The combined organic extracts were washed with water and brine, dried over Na_2SO_4, and concentrated under reduced pressure. The resulting crude product was purified by flash chromatography using gradient elution from 5% EtOAc/petroleum ether to 20% EtOAc /petroleum ether. Colorless powder (1.94 g, 66% yield), analytical TLC on silica gel, 1:10 EtOAc/petroleum ether, R_f=0.31.
^{1}H NMR (400 MHz, $CDCl_3$, ppm) δ 6.84 (2H, d, J = 3.1 Hz), 6.62 (2H, d, J = 3.1 Hz), 3.79 (6H, s), 2.46 (6H, s).
^{13}C{^{1}H} NMR (101 MHz, $CDCl_3$, ppm) δ 158.2, 144.1, 139.6, 116.6, 116.2, 113.6, 55.6, 24.2;
Elemental analysis (%): calculated for $C_{16}H_{16}Br_2O_2$: C, 48.03; H, 4.03; found: C, 47.94; H, 4.03.

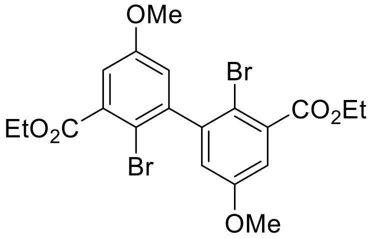

Diethyl 2,2'-dibromo-5,5'-dimethoxy-[1,1'-biphenyl]-3,3'-dicarboxylate (4d).
Dibromobiarene **S9f** (1.8 g, 4.50 mmol, 1.0 equiv) was suspended in 2:1 v/v *t*-BuOH/water (60 mL) and $KMnO_4$ (1.5 g, 9.45 mmol, 2.1 equiv) was added at ambient temperature. The resulting dark suspension was vigorously stirred and heated under reflux for 4 h, whereupon it was cooled to room temperature and additional $KMnO_4$ (1.5 g, 9.45 mmol, 2.1 equiv) was added. Heating at reflux temperature with vigorous stirring was continued for additional 18 h. The addition of additional $KMnO_4$ (1.5 g, 9.45 mmol, 2.1 equiv) to cooled reaction mixture followed by refluxing with vigorous stirring for 24 h was repeated 2 more times (a total of 6.0 g of $KMnO_4$ was used for

this reaction). The resulting brown suspension was hot-filtered thought a plug of Celite. The plug was washed with water (100 mL) and EtOH (50 mL). The combined filtrates were concentrated under reduced pressure to ≈1/3 of the starting volume. The resulting colorless solution was acidified by addition of aqueous 4 M HCl to pH 2, and extracted with EtOAc (3 × 50 mL). The combined organic layers were dried over Na_2SO_4, filtered and concentrated under reduced pressure. The colorless oil was charged in to a flame-dried round-bottomed flask and dissolved in absolute EtOH (10 mL). Thionyl chloride (1.3 mL, 18.0 mmol, 4.0 equiv) was then added dropwise, and the resulting yellowish solution was heated under reflux for 3 h. It was cooled to room temperature and all volatiles were removed under reduced pressure. Saturated aqueous $NaHCO_3$ solution (30 mL) was added, and the yellowish semi-solid residue was extracted with CH_2Cl_2 (3 × 20 mL). The combined organic extracts were washed with water, brine, dried over Na_2SO_4, and concentrated under reduced pressure. The resulting crude product was purified by flash chromatography using gradient elution from 10% Et_2O/petroleum ether to 50% Et_2O/petroleum ether, then by reversed phase column chromatography on C18 silica gel using gradient elution from 50% MeCN/water to 95% MeCN/water. Off-white oil (622 mg, 27 % yield), analytical TLC on silica gel, 1:3 EtOAc/petroleum ether, R_f=0.37.

^{1}H NMR (400 MHz, $CDCl_3$, ppm) δ 7.24 (2H, d, J = 3.1 Hz), 6.86 (2H, d, J = 3.1 Hz), 4.41 (4H, q, J = 7.1 Hz), 3.82 (6H, s), 1.41 (6H, t, J = 7.1 Hz);

^{13}C{^{1}H} NMR (101 MHz, $CDCl_3$, ppm) δ 166.8, 158.3, 144.6, 135.2, 119.0, 115.9, 112.2, 62.1, 55.9, 14.3.

HRMS (ESI): m/z $[M+H]^+$ calculated for $C_{20}H_{21}O_6Br_2^+$ 514.9705, found 514.9706.

Optimization of electrochemical oxidation/cyclization of 2,2'-dibromo-1,1'-biphenyl 4a

Table S1. Summary of optimization experiments.

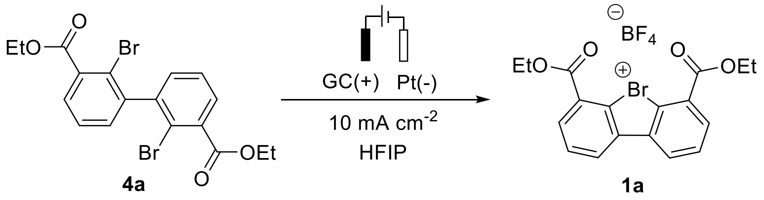

Entry	Current density, j (mA·cm^{-2})	Supporting Electrolyte	Passed charge equiv. (F)	1a, %[a]	4a, %[a]	Mass balance, %[a]
Undivided cell						
1	10	0.1 M TBA-BF_4	2	14	61	75
2	2	0.1 M TBA-BF_4	2	11	66	77
3	3	0.1 M TBA-BF_4	2	19	55	74
4	4	0.1 M TBA-BF_4	2	23	50	73
5	5	0.1 M TBA-BF_4	2	25	55	80
6	6	0.1 M TBA-BF_4	2	22	47	69
7	7	0.1 M TBA-BF_4	2	25	46	71
8	8	0.1 M TBA-BF_4	2	28	49	77
9	9	0.1 M TBA-BF_4	2	15	55	70
10	15	0.1 M TBA-BF_4	2	14	63	77
11	8	0.1 M TBA-BF_4	3	29	33	62
12	8	0.1 M TBA-BF_4	4	38	18	54
13	8	0.1 M TBA-BF_4	5	39	12	51
14	8	0.1 M TBA-BF_4	6	45	5	50
15	8	0.1 M TBA-BF_4	7	45	7	52
16	8	0.1 M TBA-BF_4	8	37	9	46
17	8	0.1 M TBA-BF_4	9	37	7	44
18	8	0.1 M TBA-BF_4	10	35	>5	>40
19	8	0.1 M TEA-BF_4	6	48	5	53
20	8	0.1 M TMA-BF_4	6	42	6	48
Divided cell						
21	8	0.25 M TEA-BF_4	2	60	24	84
22	3	0.25 M TEA-BF_4	2	41	30	71
23	4	0.25 M TEA-BF_4	2	42	32	73
24	5	0.25 M TEA-BF_4	2	47	23	70
25	6	0.25 M TEA-BF_4	2	46	22	68
26	10	0.25 M TEA-BF_4	2	54	24	78
27	13	0.25 M TEA-BF_4	2	42	28	70
28	8	0.25 M TEA-BF_4	2,5	46	21	64

Entry	Current density, j (mA·cm^{-2})	Supporting Electrolyte	Passed charge equiv. (F)	1a, %^{a}	4a, %^{a}	Mass balance, %^{a}
29	8	0.25 M TEA-BF_4	3	41	19	60
30^b	8	0.25 M TEA-BF_4	2	62	0	62
31^c	8	0.25 M TEA-BF_4	2	57	10	67
32	8	0.20 M TEA-BF_4	2	15	55	70
33	8	0.30 M TEA-BF_4	2	15	63	78
34	8	0.25 M TMA-BF_4	2	15	49	68
35	8	0.25 M TEA-ClO_4	2	31	26	57
36	8	0.25 M TEA-PF_6	2	46	19	65

^{a}Yields and mass balance were determined by ^{1}H-NMR in the crude reaction mixture using 1,2,3,4-tetrafluorobenzene as an internal standard; ^{b} Anode material: RVC; ^{c} Anode material: BDD

Published conditions for electrochemical oxidation of bromoarenes into λ^3-bromanes[20,21] were used as the starting point for the preparation of cyclic biarylbromane **1a** from dibromo biphenyl **4a** (Table S1). Accordingly, electrochemical oxidation in an undivided cell using GC as anode and platinum foil as cathode in HFIP in the presence of TBA-BF_4 as a supporting electrolyte afforded the desired biaryl-λ^3-bromane **1a** in 14% yield (entry 1) after passing 2 F per mole of starting material at 10 mA/cm^2 current density.

The following experimental variables were examined:

1) Undivided cell:
   a) Current density. Neither low current density (2 mA/cm^2) nor high current density (15 mA/cm^2) could increase the reaction yield (entry 2 or entry 10 vs entry 1). At average current densities (from 3 mA/cm^2 to 8 mA/cm^2) an increase of product **1a** formation was observed (entries 3–8 vs entry1). Thus 8 mA/cm^2 current density was used in further optimization experiments.
   b) Amount of passed charge. The increase of passed charge equivalents from 2.0 F up to 7.0 F resulted in the substantial increase of λ^3-bromane **1a** yield from 14% to 45% (entry 15 vs 1). However, a further increase of the passed charge amount did not result in further significant improvements (entries 16–18) and concomitantly, formation of degradation products was observed.
   c) Supporting electrolyte. TEA-BF_4 appeared to be somewhat superior as the electrolyte to TBA-BF_4 and TMA-BF_4 (entry 19 vs 8 and 20).

In all experiments with passed charge amount of ≥ 6.0 F per mole (entries 14–20), nearly complete conversion of the starting **4a** and moderate yield of the desired **1a** was observed pointing at a possible degradation of starting material or product. Linear sweep voltammetry (LVS) experiments (0.1 M TBA-BF_4 in HFIP on a Pt disk electrode) revealed that the reduction current increases almost 4 times upon the addition of 5 mM **1a** to the electrolyte (see Figure S1). At the same time, passing 6.0 F per mole through a solution of **1a** in 50 mM TBA-BF_4/HFIP at j = 8 mA/cm^2 led to 60% λ^3-bromane **1a** degradation, suggesting that cationic **1a**, formed on anode, decomposes on a cathode. To avoid the undesired cathodic decomposition of **1a**, cathode and anode chambers were separated, and further experiments were performed in a divided cell.

2) Divided cell:

a) Cell type. The change of the cell type increased the yield of product **1a** from 28% (entry 8, undivided cell) to 60% (entry 21, divided cell).

b) Current density. A lower current density (3 mA/cm^2) resulted in an increase of the reaction time and slightly reduced product yield (entry 22 vs entry 21). Higher current densities (10 and 13 mA/cm^2, entries 26 and 27, respectively) led to increased conversion at the expense of the side product formation. Thus, 8 mA/cm^2 current density was used in further optimization experiments.

c) Amount of passed charge. The increase of passed charge equivalents from 2.0 F up to 3.0 F resulted in the product **1a** yield decrease from 60% to 41% (entry 29 vs 21).

d) Working electrode material. The replacement of working electrode material to BDD or RVC (entry 30 and 31 vs 21) gave no increase in product yield.

e) Amount of electrolyte. Variation of electrolyte amount was not successful (entries 32,33 vs 21).

f) Supporting electrolyte. TEA-BF_4 appeared to be somewhat superior as the electrolyte to TMA-BF_4, TEA-ClO_4 or TEA-PF_6 (entry 21 vs 34–36).

λ^3-Bromane 1 synthesis via electrochemical oxidation of bromobiphenyls 4

General procedure G for electrochemical oxidation of bromobiphenyls 4

An anode chamber of 10 mL divided electrochemical cell *IKA Pro-Divide* was charged with biphenyl **4** (0.15 mmol, 1 equiv), both anode and cathode chamber were charged with TEA-BF_4 (0.75 mmol, 5 equiv) and HFIP (3 mL). A 8 × 5 × 2 glassy carbon plate (immersed electrode surface area $A = 1.0$ cm^2) was used as a working electrode and a 5 × 4 × 0.1 mm Pt sheet (immersed electrode surface area $A = 1.0$ cm^2) as a counter electrode. The electrolysis was carried out under galvanostatic conditions at room temperature, and 2.0 F/mol charge with a current density of 8 mA/cm^2 was passed through the colorless solution. The resulting dark-yellow solution was concentrated under reduced pressure and the crude product was purified by reversed phase column chromatography on C18 silica gel using gradient elution from 5% MeCN in water to 95% MeCN in water.

4,6-Bis(ethoxycarbonyl)dibenzo[*b*,*d*]bromol-5-ium tetrafluoroborate (1a).

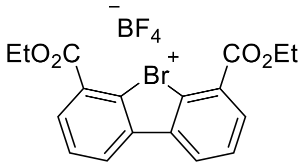

Following general procedure G, biphenyl **4a** (68 mg, 0.15 mmol) was converted into **1a**. White powder (24 mg, 35% yield).

^{1}H NMR (400 MHz, $CDCl_3$, ppm) δ 9.23 (2H, dd, *J* = 7.7, 1.4 Hz), 8.41 (2H, dd, *J* = 7.7, 1.4 Hz), 8.16 (2H, t, *J* = 7.7 Hz), 4.64 (4H, q, *J* = 7.2 Hz), 1.53 (6H, t, *J* = 7.2 Hz);

^{13}C{^{1}H} NMR (101 MHz, $CDCl_3$, ppm) δ 165.1, 135.5, 133.6, 133.5, 132.3, 132.2, 124.6, 64.8, 14.1;

^{19}F NMR (376 MHz, $CDCl_3$, ppm) δ -153.6;

HRMS (ESI): m/z $[M-BF_4]^+$ calculated for $C_{18}H_{16}BrO_4^+$ 375.0232, found 375.0242.

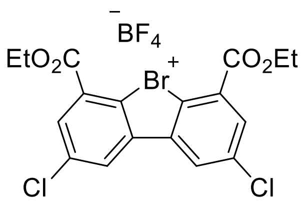

2,8-Dichloro-4,6-bis(ethoxycarbonyl)dibenzo[*b*,*d*]bromol-5-ium tetrafluoroborate (1b).

Following general procedure G, biphenyl **4b** (79 mg, 0.15 mmol) was converted into **1b**. White powder (14 mg, 18% yield).

^{1}H NMR (400 MHz, DMSO-d_6, ppm) δ 9.29 (2H, d, *J* = 2.2 Hz), 8.53 (2H, d, *J* = 2.2 Hz), 4.62 (4H, q, *J* = 7.1 Hz), 1.47 (6H, t, *J* = 7.1 Hz);

$^{13}C\{^1H\}$ NMR (101 MHz, DMSO-d_6, ppm) δ 163.8, 138.0, 135.3, 132.7, 131.6, 131.4, 126.0, 64.8, 13.9;

^{19}F NMR (376 MHz, DMSO-d_6, ppm) δ -148.4;

HRMS (ESI): m/z $[M-BF_4]^+$ calculated for $C_{18}H_{14}BrCl_2O_4^+$ 442.9453, found 442.9458.

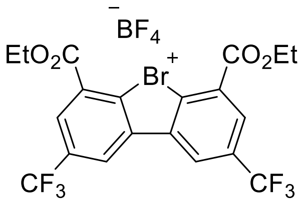

4,6-Bis(ethoxycarbonyl)-2,8-bis(trifluoromethyl)dibenzo[*b*,*d*]bromol-5-ium tetrafluoroborate (1c).

Following general procedure G, biphenyl **4c** (89 mg, 0.15 mmol) was converted into **1c**. White powder (22 mg, 25% yield).

^1H NMR (400 MHz, DMSO-d_6, ppm) δ 9.79 (2H, d, J = 1.4 Hz), 8.76 (2H, d, J = 1.4 Hz), 4.67 (4H, q, J = 7.1 Hz), 1.50 (6H, t, J = 7.1 Hz);

$^{13}C\{^1H\}$ NMR (101 MHz, DMSO-d_6, ppm) δ 163.9, 138.0, 135.4, 133.5 (q, $^2J_{C-F}$ = 33.8 Hz), 129.6 (q, $^3J_{C-F}$ = 3.8 Hz), 128.4 (q, $^3J_{C-F}$ = 3.7 Hz), 126.1, 122.9 (q, $^1J_{C-F}$ = 273.9 Hz), 65.0, 13.9;

^{19}F NMR (376 MHz, DMSO-d_6, ppm) δ -60.8, -148.4.

HRMS (ESI): m/z $[M-BF_4]^+$ calculated for $C_{20}H_{14}BrF_6O_4^+$ 510.9980, found 510.9979.

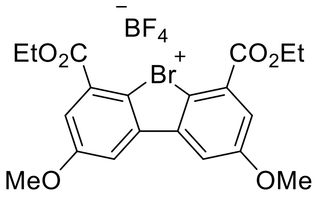

4,6-Bis(ethoxycarbonyl)-2,8-dimethoxydibenzo[*b*,*d*]bromol-5-ium tetrafluoroborate (1d).

Following general procedure G, biphenyl **4d** (77 mg, 0.15 mmol) was converted into **1d**. Pale yellow powder (14 mg, 18% yield).

^1H NMR (400 MHz, $CDCl_3$, ppm) δ 8.57 (2H, s), 7.84 (2H, s), 4.62 (4H, q, J = 7.1 Hz), 4.14 (6H, s), 1.52 (6H, t, J = 7.1 Hz);

$^{13}C\{^1H\}$ NMR (101 MHz, $CDCl_3$, ppm) δ 165.1, 164.1, 136.9, 124.6, 123.0, 121.6, 115.1, 64.7, 58.1, 14.2;

^{19}F NMR (376 MHz, $CDCl_3$, ppm) δ -148.3;

HRMS (ESI): m/z $[M-BF_4]^+$ calculated for $C_{20}H_{20}O_6Br^+$ 435.0443, found 435.0443.

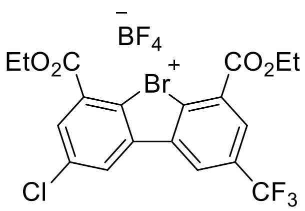

2-Chloro-4,6-bis(ethoxycarbonyl)-8-(trifliuoromethyl) dibenzo[*b,d*]bromol-5-ium tetrafluoroborate (1e).

Following general procedure G, biphenyl **4e** (77 mg, 0.15 mmol) was converted into **1e**. White powder (15 mg, 19% yield).

^{1}H NMR (400 MHz, DMSO-d_6, ppm) δ 9.62 (1H, d, *J* = 1.5 Hz), 9.48 (1H, d, *J* = 2.3 Hz), 8.73 (1H, d, *J* = 1.5 Hz), 8.57 (1H, d, *J* = 2.3 Hz), 4.69 – 4.59 (4H, m), 1.52 – 1.44 (6H, m);

^{13}C{^{1}H} NMR (101 MHz, DMSO-d_6, ppm) δ 163.9, 163.8, 138.1, 137.7, 135.5, 135.3, 133.5 (q, $^2J_{C-F}$ = 33.9 Hz), 133.0, 131.8, 131.7, 129.11(q, $^3J_{C-F}$ = 3.7 Hz), 128.2 (q, $^3J_{C-F}$ = 3.8 Hz), 126.1, 126.0, 122.8 (q, $^1J_{C-F}$ = 273.7 Hz), 64.9, 64.8, 13.9;

^{19}F NMR (376 MHz, DMSO-d_6, ppm) δ -60.8, -148.4;

HRMS (ESI): m/z $[M-BF_4]^+$ calculated for $C_{19}H_{14}O_4ClBrF_3^+$ 476.9716, found 476.9725.

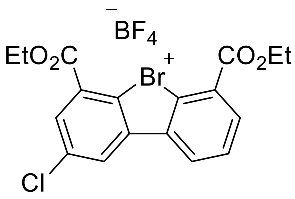

2-Chloro-4,6-bis(ethoxycarbonyl)dibenzo[*b,d*]bromol-5-ium tetrafluoroborate (1f).

Following general procedure G, biphenyl **4f** (74 mg, 0.15 mmol) was converted into **1f**. Pale yellow powder (14 mg, 19% yield).

^{1}H NMR (400 MHz, $CDCl_3$, ppm) δ 8.71 (1H, dd, *J* = 7.7 Hz, 1.4 Hz), 8.61 (1H, d, *J* = 2.1 Hz), 8.40 (1H, d, *J* = 7.7 Hz, 2.1 Hz), 8.29 (1H, d, *J* = 2.1 Hz), 8.08 (1H, t, *J* = 7.7 Hz), 4.70 – 4.60 (4H, m), 1.58 – 1.49 (6H, m);

^{13}C{^{1}H} NMR (101 MHz, $CDCl_3$, ppm) δ 165.2, 164.2, 139.9, 136.9, 134.4, 134.3, 133.7, 132.6, 132.3, 131.8, 131.7, 131.2, 125.9, 124.7, 65.2, 64.9, 14.2, 14.2;

^{19}F NMR (376 MHz, $CDCl_3$, ppm) δ -153.6;

HRMS (ESI): m/z $[M-BF_4]^+$ calculated for $C_{18}H_{15}O_4ClBr^+$ 408.9842, found 408.9840.

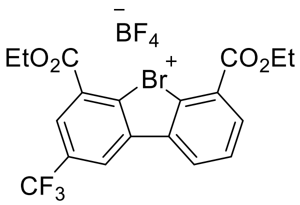

4,6-Bis(ethoxycarbonyl)-2-(trifluoromethyl)dibenzo[*b,d*]bromol-5-ium tetrafluoroborate (1g).

Following general procedure G, biphenyl **4g** (79 mg, 0.15 mmol) was converted into **1g**. White powder (16 mg, 20% yield).

^{1}H NMR (400 MHz, $CDCl_3$, ppm) δ 8.82 (1H, d, *J* = 1.9 Hz), 8.74 (1H, dd, *J* = 7.9, 1.9 Hz), 8.54 (1H, d, *J* = 1.9 Hz), 8.42 (1H, dd, *J* = 7.9, 1.9 Hz), 8.09 (1H, t, *J* = 7.9 Hz), 4.71 – 4.61 (4H, m), 1.58 – 1.50 (6H, m);

^{13}C{^{1}H} NMR (101 MHz, $CDCl_3$, ppm) δ 165.2, 164.1, 136.9, 136.8, 135.6 (q, $^{2}J_{C\text{-}F}$ = 34.5 Hz), 135.0, 134.5, 133.6, 132.8, 131.9, 128.3 (q, $^{3}J_{C\text{-}F}$ = 3.6 Hz), 127.9 (q, $^{3}J_{C\text{-}F}$ = 3.6 Hz), 126.3, 124.9, 122.6 (q, $^{1}J_{C\text{-}F}$ = 274.0 Hz), 65.4, 65.0, 14.2;

^{19}F NMR (376 MHz, $CDCl_3$, ppm) δ -62.4, -153.9;

HRMS (ESI): m/z $[M\text{-}BF_4]^+$ calculated for $C_{19}H_{15}O_4BrF_3^+$ 443.0106, found 443.0109.

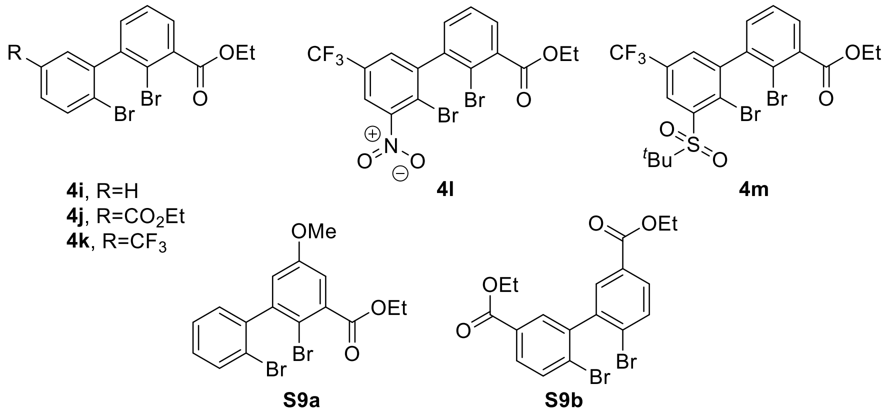

Scheme S1. List of substrates that do not undergo electrochemical oxidation/cyclization reaction.

Crystal data and structure refinement for biaryl λ^3-bromane 1a

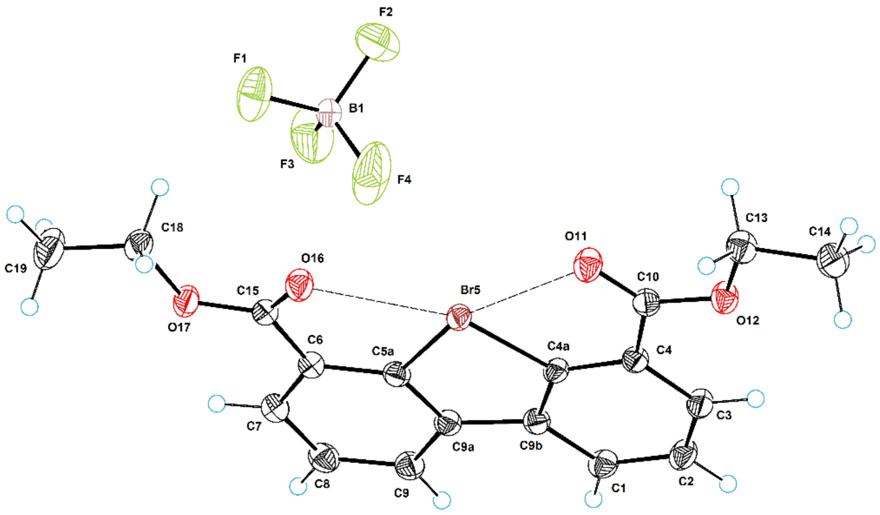

Identification code	ISR-454-7
Empirical formula	$C_{18}H_{16}BBrF_4O_4$
Formula weight	463.05
Temperature/K	160.0(1)
Crystal system	monoclinic
Space group	$P2_1/c$
a/Å	10.2442(1)
b/Å	9.9297(1)
c/Å	18.1776(2)
α/°	90
β/°	99.648(1)
γ/°	90
Volume/Å^3	1822.91(3)
Z	4
ρ_{calc}g/cm^3	1.6870
μ/mm^{-1}	3.659
F(000)	928
Crystal size/mm^3	0.18 × 0.13 × 0.05
Radiation	Cu Kα (λ = 1.54184 Å)
2Θ max. for data collection/°	160
Index ranges	$-12 \leq h \leq 13$, $-12 \leq k \leq 9$, $-23 \leq l \leq 23$
Reflections collected	26364
Independent reflections	3956 [R_{int} = 0.0281, R_{sigma} = 0.0174]
Data/restraints/parameters	3956/0/263
Goodness-of-fit on F^2	1.041
Final R indexes [$I > 2\sigma(I)$]	R_1 = 0.0403, wR_2 = 0.1087
Final R indexes [all data]	R_1 = 0.0409, wR_2 = 0.1093
Largest diff. peak/hole / e Å^{-3}	1.36/-1.00

Cyclic voltammetry

The experiments were carried out in a custom-made three-electrode cell using a PGSTAT 128N (Metrohm, Autolab). A glassy carbon disc (diameter: 1.6 mm) or platinum disc (diameter: 3.0 mm) served as the working electrode, and a platinum wire as the counter electrode. The glassy carbon disk was polished using polishing alumina (0.05 μm) prior to each experiment. As reference, an Ag/$AgNO_3$ electrode [silver wire in 0.1 M TBA-BF_4/CH_3CN solution; c($AgNO_3$) = 0.01 M; E_0 = −87 mV vs. Fc/Fc^+ couple][22] was used, and this compartment was separated from the rest of the cell with a Vycor frit. Unless stated otherwise, TBA-BF_4 (0.1 M, electrochemical grade) was employed as the supporting electrolyte in HFIP solution. The electrolyte was purged with Ar for at least 5 min prior to recording. Compounds were analyzed at a concentration of 5 mM and a scan rate of 100 mV s^{-1}. The half-peak potentials ($E_{P/2}$) and peak potentials E_P were extracted from background-corrected voltammograms.

Anodic oxidation of bromobiphenyls 4, S9a-b

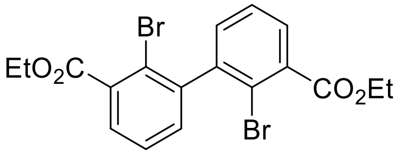

4a

$E_{P/2}$ = +2.28 V

v = 100 mV s^{-1}

c = 5 mM

Solvent: HFIP

Start point = 0.0 V, scanned in positive direction

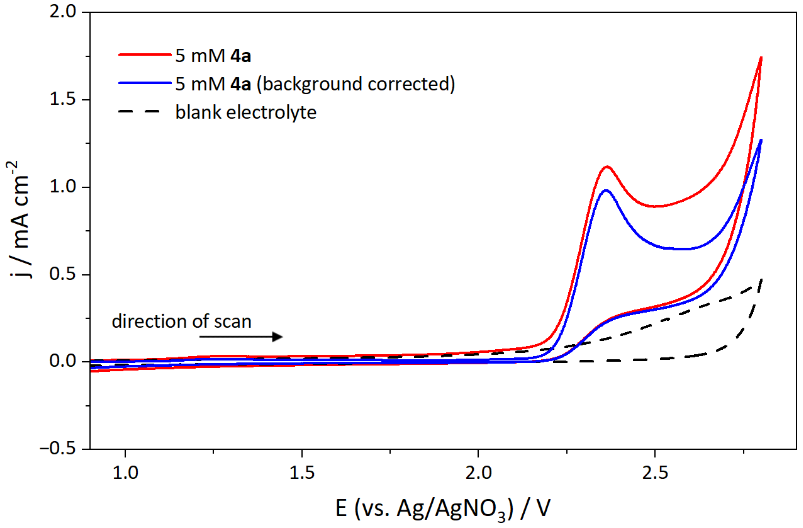

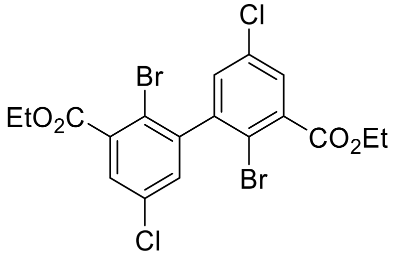

4b

$E_{P/2}$ = +2.35 V
v = 100 mV s^{-1}
c = 5 mM
Solvent: HFIP
Start point = 0.0 V, scanned in positive direction

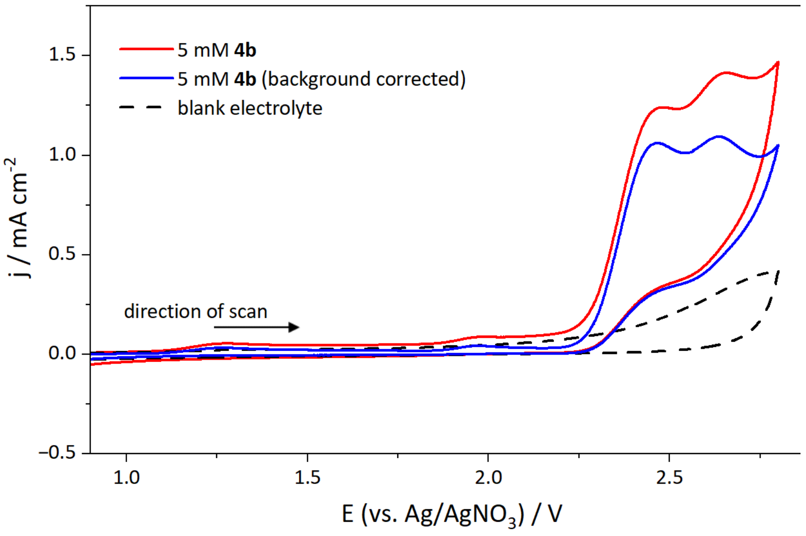

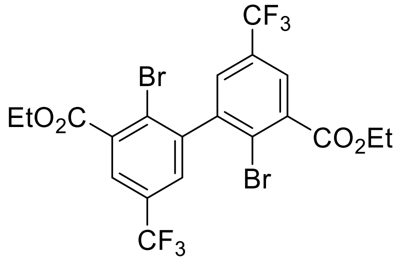

4c

$E_{P/2}$ = +2.88 V
v = 100 mV s^{-1}
c = 5 mM
Solvent: HFIP
Start point = 0.0 V, scanned in positive direction

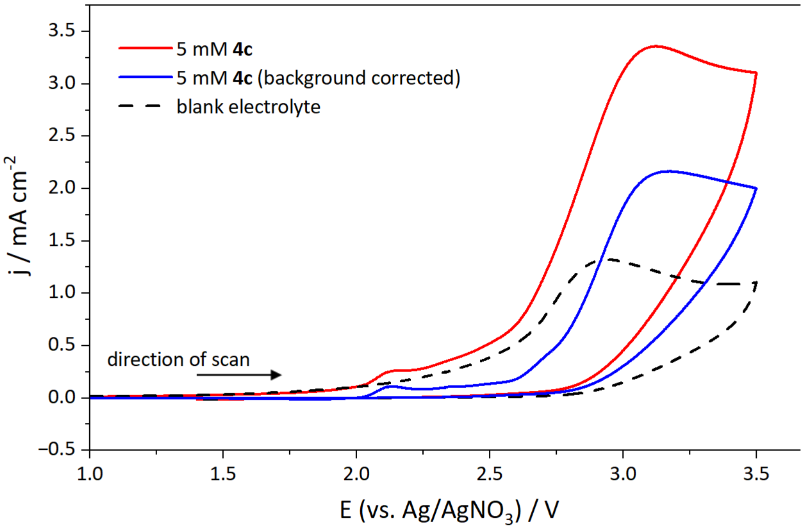

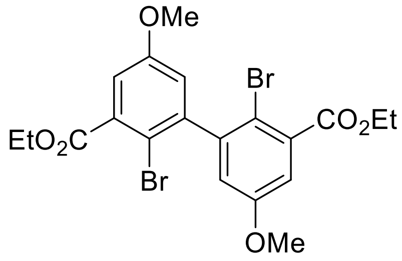

4d

$E_{P/2}$ = +1.77 V
v = 100 mV s^{-1}
c = 5 mM
Solvent: HFIP
Start point = 0.0 V, scanned in positive direction

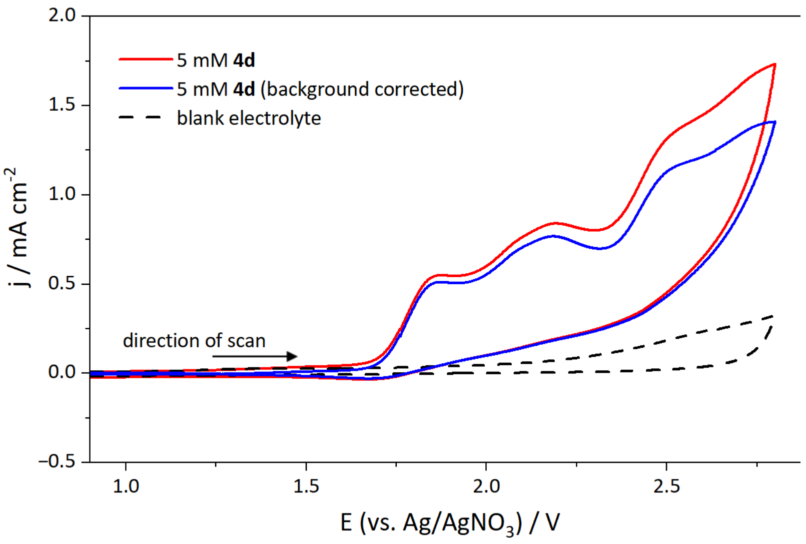

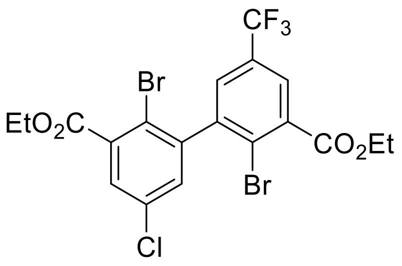

4e

$E_{P/2}$ = +2.39 V

v = 100 mV s^{-1}

c = 5 mM

Solvent: HFIP

Start point = 0.0 V, scanned in positive direction

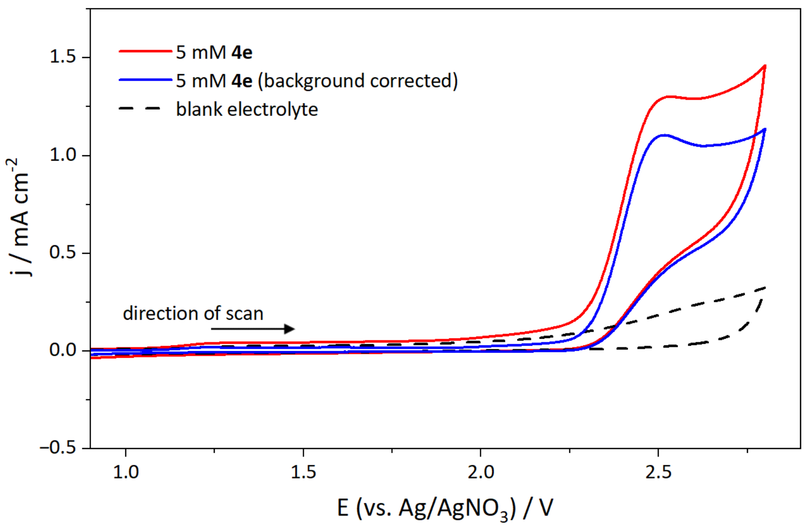

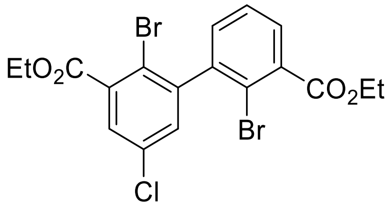

4f

$E_{P/2}$ = +2.30 V

v = 100 mV s^{-1}

c = 5 mM

Solvent: HFIP

Start point = 0.0 V, scanned in positive direction

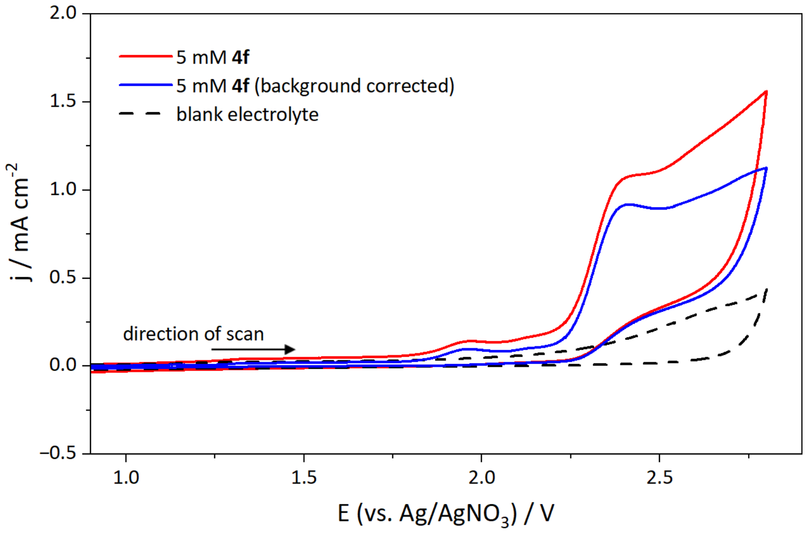

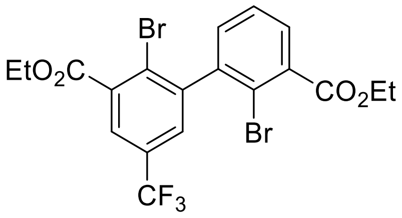

4g

$E_{P/2}$ = +2.45 V

v = 100 mV s^{-1}

c = 5 mM

Solvent: HFIP

Start point = 0.0 V, scanned in positive direction

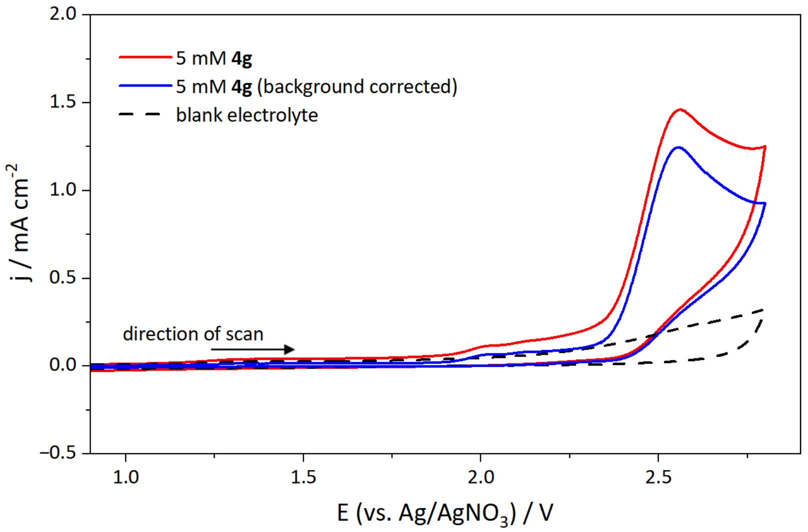

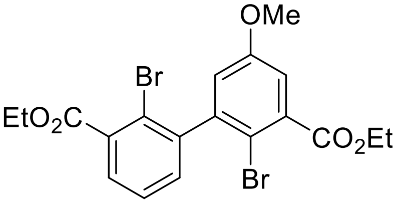

4h

$E_{P/2}$ = +1.75 V

v = 100 mV s^{-1}

c = 5 mM

Solvent: HFIP

Start point = 0.0 V, scanned in positive direction

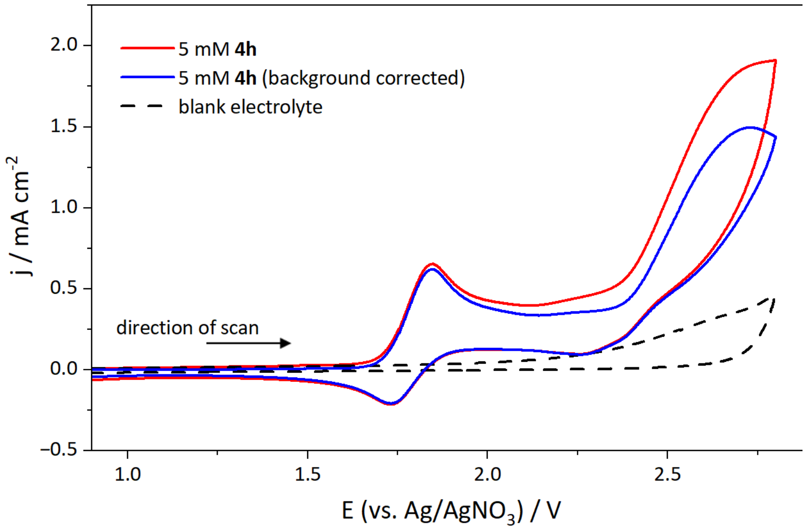

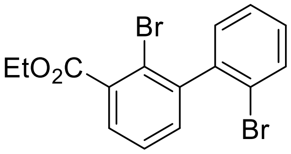

4i

$E_{P/2}$ = +2.15 V

v = 100 mV s^{-1}

c = 5 mM

Solvent: HFIP

Start point = 0.0 V, scanned in positive direction

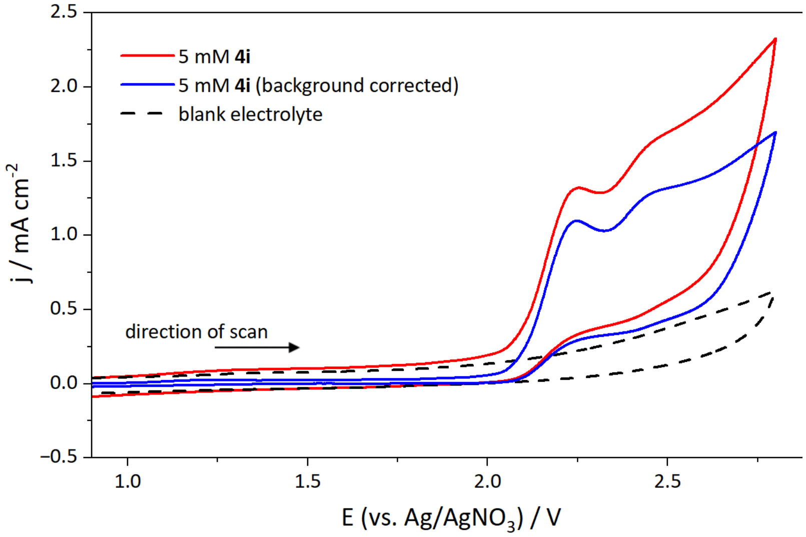

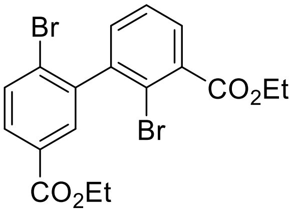

4j

$E_{P/2}$ = +2.34 V

v = 100 mV s^{-1}

c = 5 mM

Solvent: HFIP

Start point = 0.0 V, scanned in
positive direction

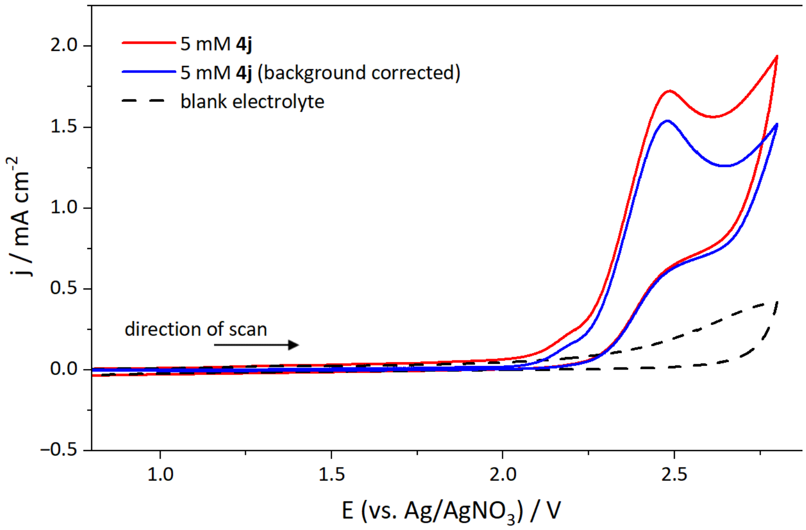

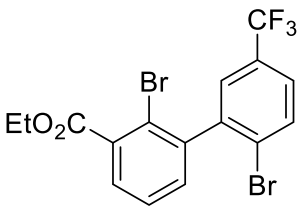

4k

$E_{P/2}$ = +2.37 V

v = 100 mV s^{-1}

c = 5 mM

Solvent: HFIP

Start point = 0.0 V, scanned in positive direction

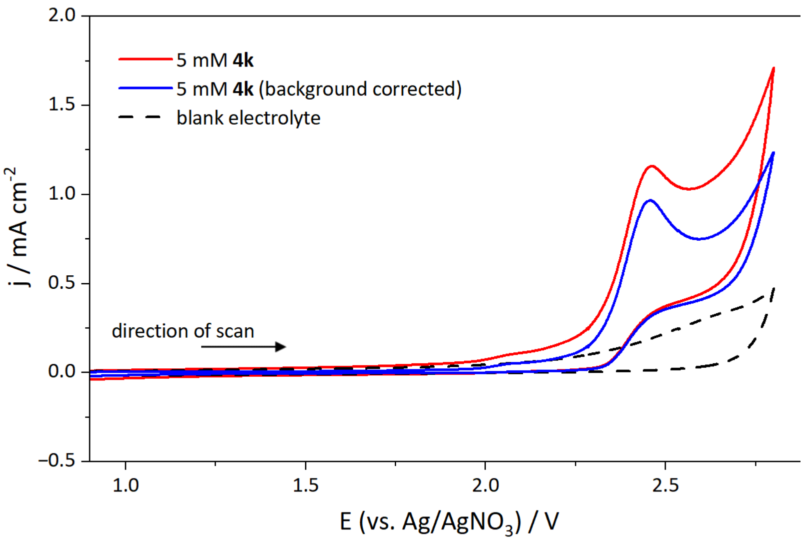

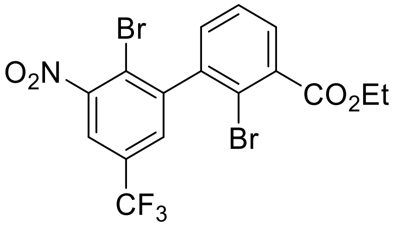

4l

$E_{P/2}$ = +2.53 V

v = 100 mV s^{-1}

c = 5 mM

Solvent: HFIP

Start point = 0.0 V, scanned in positive direction

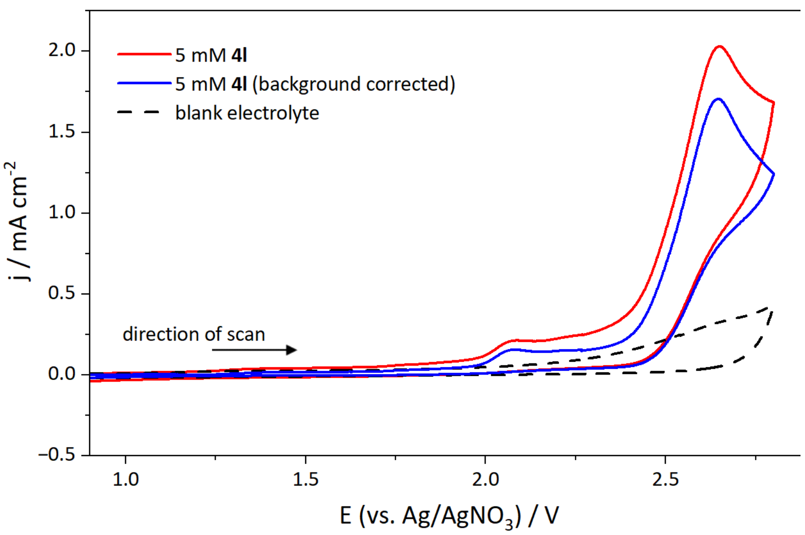

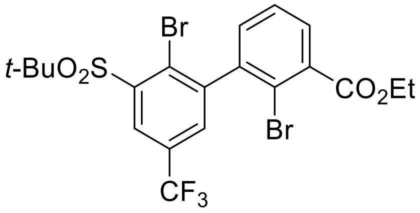

4m

$E_{P/2}$ = +2.54 V

v = 100 mV s^{-1}

c = 5 mM

Solvent: HFIP

Start point = 0.0 V, scanned in positive direction

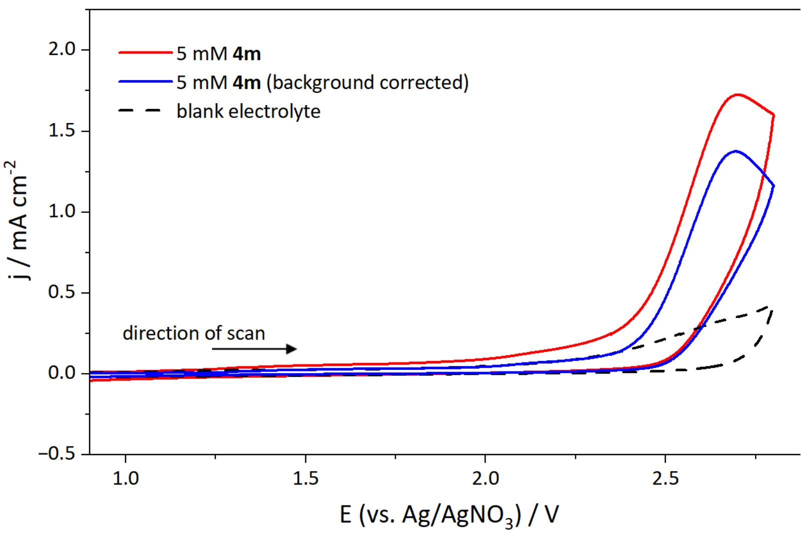

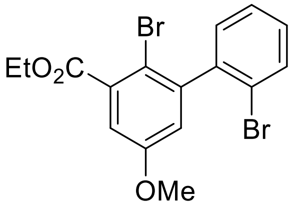

S9a

$E_{P/2}$ = +1.73 V

v = 100 mV s^{-1}

c = 5 mM

Solvent: HFIP

Start point = 0.0 V, scanned in positive direction

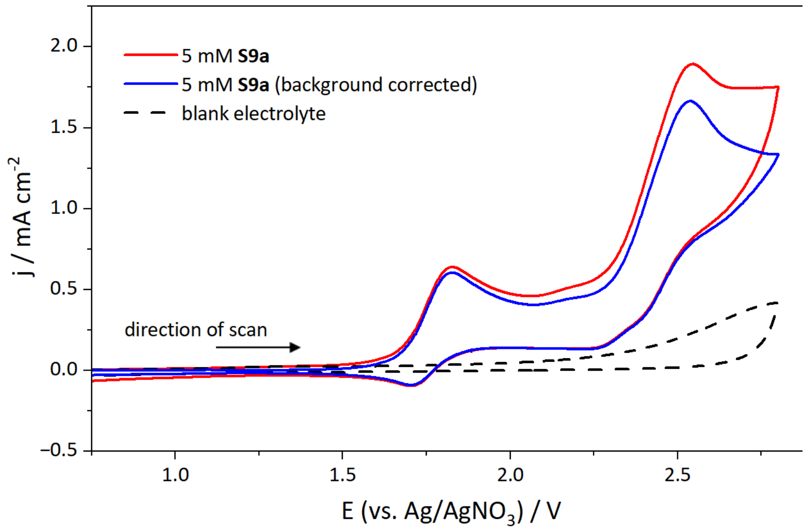

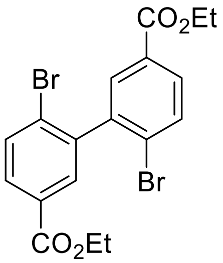

S9b

$E_{P/2}$ = +2.37 V

v = 100 mV s^{-1}

c = 5 mM

Solvent: HFIP

Start point = 0.0 V, scanned in positive direction

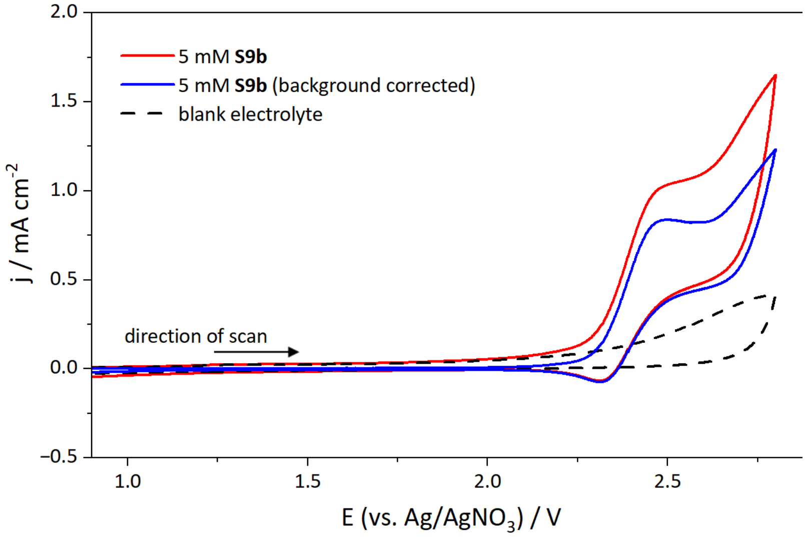

Table S2. Summary of the half-peak potentials of bromobiphenyls **4, S9a-b** in HFIP

**Compound**	$E_{P/2}$ **/ V**
**4a**	+2.28
**4b**	+2.35
**4c**	+2.88
**4d**	+1.77
**4e**	+2.39
**4f**	+2.30
**4g**	+2.45
**4h**	+1.75
**4i**	+2.15
**4j**	+2.34
**4k**	+2.37
**4l**	+2.53
**4m**	+2.54
**S9a**	+1.73
**S9b**	+2.37

Cathodic reduction of λ^3-bromane 1a

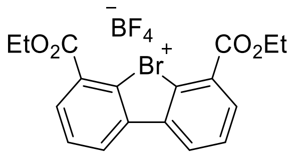

1a
$E_{P/2}$ = -1.40 V
v = 100 mV s^{-1}
c = 5 mM
Solvent: MeCN
Start point = 1.0 V,
scanned in negative
direction

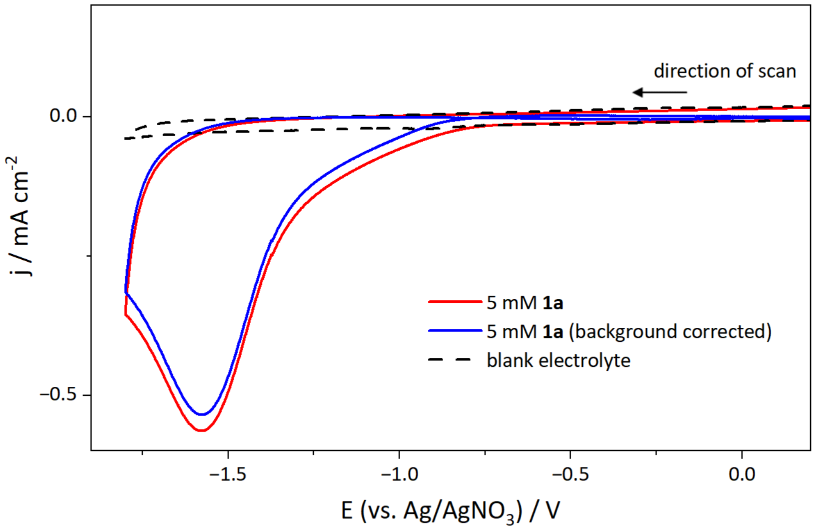

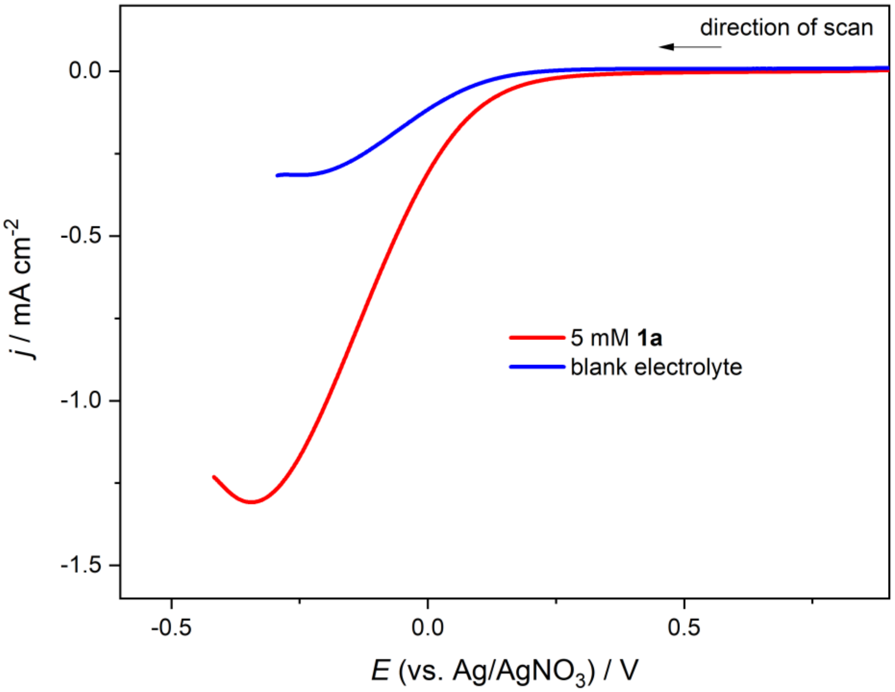

Figure S1. Linear sweep voltammograms (LSV) of blank electrolyte (0.1 M TBA-BF_4 in HFIP) and λ^3-bromane **1a** (c = 5 mM) recorded at 100 mV s^{-1} on Pt disk (diameter: 3.0 mm) working electrode.

References

(1) Zhang, Z.; Chen, Y.; He, S.; Zhang, J.; Xu, X.; Yang, Y.; Nosheen, F.; Saleem, F.; He, W.; Wang, X. Hierarchical Zn/Ni-MOF-2 Nanosheet-Assembled Hollow Nanocubes for Multicomponent Catalytic Reactions. *Angew. Chem. Int. Ed.*, **2014**, *53*, 12517–12521. https://doi.org/10.1002/anie.201406484.

(2) Yuan, H.; Yin, W.; Hu, J.; Li, Y. 3-Sulfonyloxyaryl(Mesityl)Iodonium Triflates as 1,2-Benzdiyne Precursors with Activation via Ortho-Deprotonative Elimination Strategy. *Nat. Commun.*, **2023**, 14. https://doi.org/10.1038/s41467-023-37196-3.

(3) Kotha, S.; Shah, V. R.; Mandal, K. Formation of Arenes via Diallylarenes: Strategic Utilization of Suzuki–Miyaura Cross-Coupling, Claisen Rearrangement and Ring-Closing Metathesis. *Adv. Synth. Catal.*, **2007**, *349*, 1159–1172. https://doi.org/10.1002/adsc.200600469

(4) Pu, F.; Zhang, L.-Y.; Liu, Z.-W.; Shi, X.-Y. Palladium (II)-Catalyzed Decarboxylative Cross-Dehydrogenative Coupling: Direct Synthesis of Meta-Substituted Biaryls from Aromatic Acids. *Adv. Synth. Catal.*, **2018**, *360*, 2644–2649. https://doi.org/10.1002/adsc.201800333

(5) Liu, D.; Yao, R.; Dong, R.; Jia, F.; Fu, M. A Strategy to Increase Phosphorescent Efficiency without Perturbing Emission Color for Benzothiazole-Containing Iridium Phosphors. *Dyes Pigments*, **2017**, *145*, 528–537. https://doi.org/10.1016/j.dyepig.2017.06.048.

(6) Skulski, L.; Kraszkiewicz, L.; Sosnowski, M. Oxidative Iodination of Deactivated Arenes in Concentrated Sulfuric Acid with I2/NaIO4and KI/NaIO4Iodinating Systems. *Synthesis*, **2006**, 1195–1199. https://doi.org/10.1055/s-2006-926374.

(7) Fu, Z.; Jiang, L.; Zuo, Q.; Li, Z.; Liu, Y.; Wei, Z.; Cai, H. Inexpensive NaX (X = I, Br, Cl) as a Halogen Donor in the Practical Ag/Cu-Mediated Decarboxylative Halogenation of Aryl Carboxylic Acids under Aerobic Conditions. *Org. Biomol. Chem.*, **2018**, *16*, 5416–5421. https://doi.org/10.1039/c8ob01095a.

(8) Doye, S.; Severin, R.; Mujahidin, D.; Reimer, J. Synthesis of Benzylisoquinoline Derivatives Possessing Electron-Withdrawing Substituents on the Benzene Ring of the Isoquinoline Skeleton. *Heterocycles*, **2007**, *74*, 683. https://doi.org/10.3987/com-07-s(w)54.

(9) Levi, Z. U.; Tilley, T. D. Versatile Synthesis of Pentalene Derivatives via the Pd-Catalyzed Homocoupling of Haloenynes. *J. Am. Chem. Soc.*, **2009**, *131*, 2796–2797. https://doi.org/10.1021/ja809930f.

(10) Soldatova, N.; Postnikov, P.; Kukurina, O.; Zhdankin, V. V.; Yoshimura, A.; Wirth, T.; Yusubov, M. S. One-Pot Synthesis of Diaryliodonium Salts from Arenes and Aryl Iodides with Oxone–Sulfuric Acid. Beilstein *J. Org. Chem.*, **2018**, *14*, 849–855. https://doi.org/10.3762/bjoc.14.70.

(11) Bugaenko, D. I.; Volkov, A. A.; Andreychev, V. V.; Karchava, A. V. Reaction of Diaryliodonium Salts with Potassium Alkyl Xanthates as an Entry Point to Accessing Organosulfur Compounds. *Org. Lett.* **2023**, *25*, 272–276. https://doi.org/10.1021/acs.orglett.2c04143.

(12) Kerr, W. J.; Knox, G. J.; Reid, M.; Tuttle, T.; Bergare, J.; Bragg, R. A. Computationally-Guided Development of a Chelated NHC-P Iridium(I) Complex for the Directed Hydrogen Isotope

Exchange of Aryl Sulfones. *ACS Catal*. **2020**, *10*, 11120–11126. https://doi.org/10.1021/acscatal.0c03031.

(13) Mistico, L.; Querolle, O.; Meerpoel, L.; Angibaud, P.; Durandetti, M.; Maddaluno, J. Access to Silylated Pyrazole Derivatives by Palladium-Catalyzed C−H Activation of a TMS Group. *Chem. Eur. J.*, **2016**, *22*, 9687–9692. https://doi.org/10.1002/chem.201601533

(14) Hosoya, T.; Yoshida, S.; Nishiyama, Y.; Misawa, Y.; Hazama, Y.; Oya, K. Synthesis of Diverse 3-Azido-5-(Azidomethyl)Benzene Derivatives via Formal C–H Azidation and Functional Group-Selective Transformations. *Heterocycles*, **2019**, *99*, 1053. https://doi.org/10.3987/com-18-s(f)72

(15) Qiu, D.; Mo, F.; Zheng, Z.; Zhang, Y.; Wang, J. Gold(III)-Catalyzed Halogenation of Aromatic Boronates with N-Halosuccinimides. *Org. Lett.*, **2010**, *12*, 5474–5477. https://doi.org/10.1021/ol102350v.

(16) Yoshida, H.; Yoshida, R.; Takaki, K. Synchronous Ar-F and Ar-Sn Bond Formation through Fluorostannylation of Arynes. *Angew. Chem. Int. Ed.*, **2013**, *52*, 8629–8632. https://doi.org/10.1002/anie.201302783.

(17) Kerr, W. J.; Knox, G. J.; Reid, M.; Tuttle, T.; Bergare, J.; Bragg, R. A. Computationally-Guided Development of a Chelated NHC-P Iridium(I) Complex for the Directed Hydrogen Isotope Exchange of Aryl Sulfones. *ACS Catal.*, **2020**, *10*, 11120–11126. https://doi.org/10.1021/acscatal.0c03031.

(18) Chen, S.-Y.; Zhang, J.; Li, Y.-H.; Wen, J.; Bian, S.-Q.; Yu, X.-Q. Cobalt-Catalyzed Homo-Coupling of Aryl and Alkenyl Bromide Using Atmospheric Oxygen as Oxidant. *Tetrahedron Lett.*, **2009**, *50*, 6795–6797. https://doi.org/10.1016/j.tetlet.2009.09.092.

(19) Zysman-Colman, E.; Arias, K.; Siegel, J. S. Synthesis of Arylbromides from Arenes and N-Bromosuccinimide (NBS) in Acetonitrile — A Convenient Method for Aromatic Bromination. *Can. J. Chem.*, **2009**, *87*, 440–447. https://doi.org/10.1139/v08-176.

(20) Sokolovs, I.; Mohebbati, N.; Francke, R.; Suna, E. Electrochemical Generation of Hypervalent Bromine(III) Compounds. *Angew. Chem. Int. Ed*. **2021**, *60*, 15832–15837. https://doi.org/10.1002/anie.202104677

(21) Sokolovs, I.; Suna, E. Electrochemical Synthesis of Dimeric λ^3-Bromane: Platform for Hypervalent Bromine(III) Compounds. *Org. Lett*. **2023**, *25*, 2047–2052. https://doi.org/10.1021/acs.orglett.3c00405.

(22) Pavlishchuk, V. V.; Addison, A. W. Conversion Constants for Redox Potentials Measured versus Different Reference Electrodes in Acetonitrile Solutions at 25°C. *Inorg. Chim. Acta* **2000**, *298*, 97–102. https://doi.org/10.1016/S0020-1693(99)00407-7.

NMR spectra

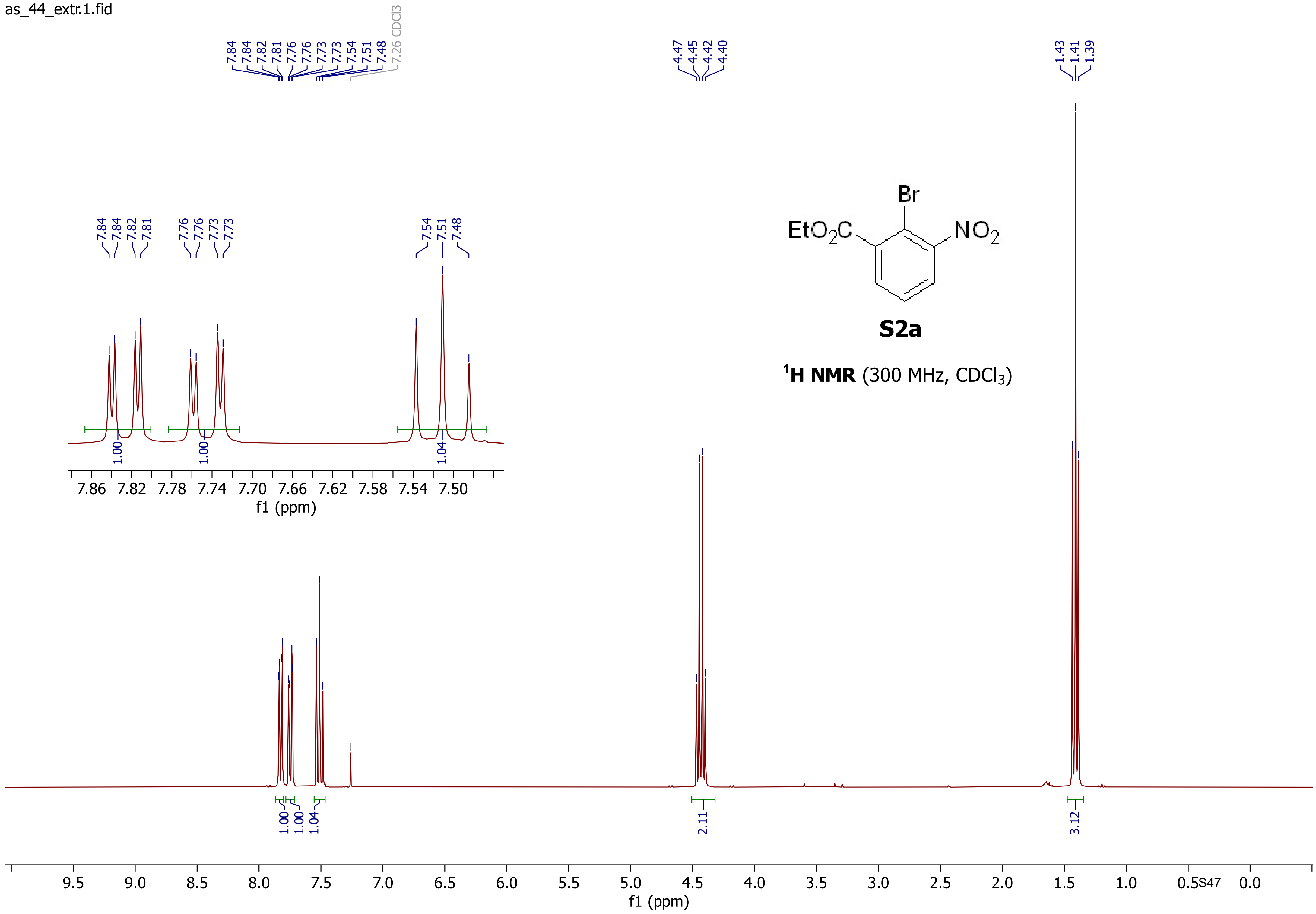
as_44_extr.1.fid
7.84
7.84
7.82
7.81
7.76
7.76
7.73
7.73
7.54
7.51
7.48
7.26 CDCl3
4.47
4.45
4.42
4.40
1.43
1.41
1.39
Br
EtO2C
NO2
S2a
1H NMR (300 MHz, CDCl3)
1.00
1.00
1.04
2.11
3.12
f1 (ppm)

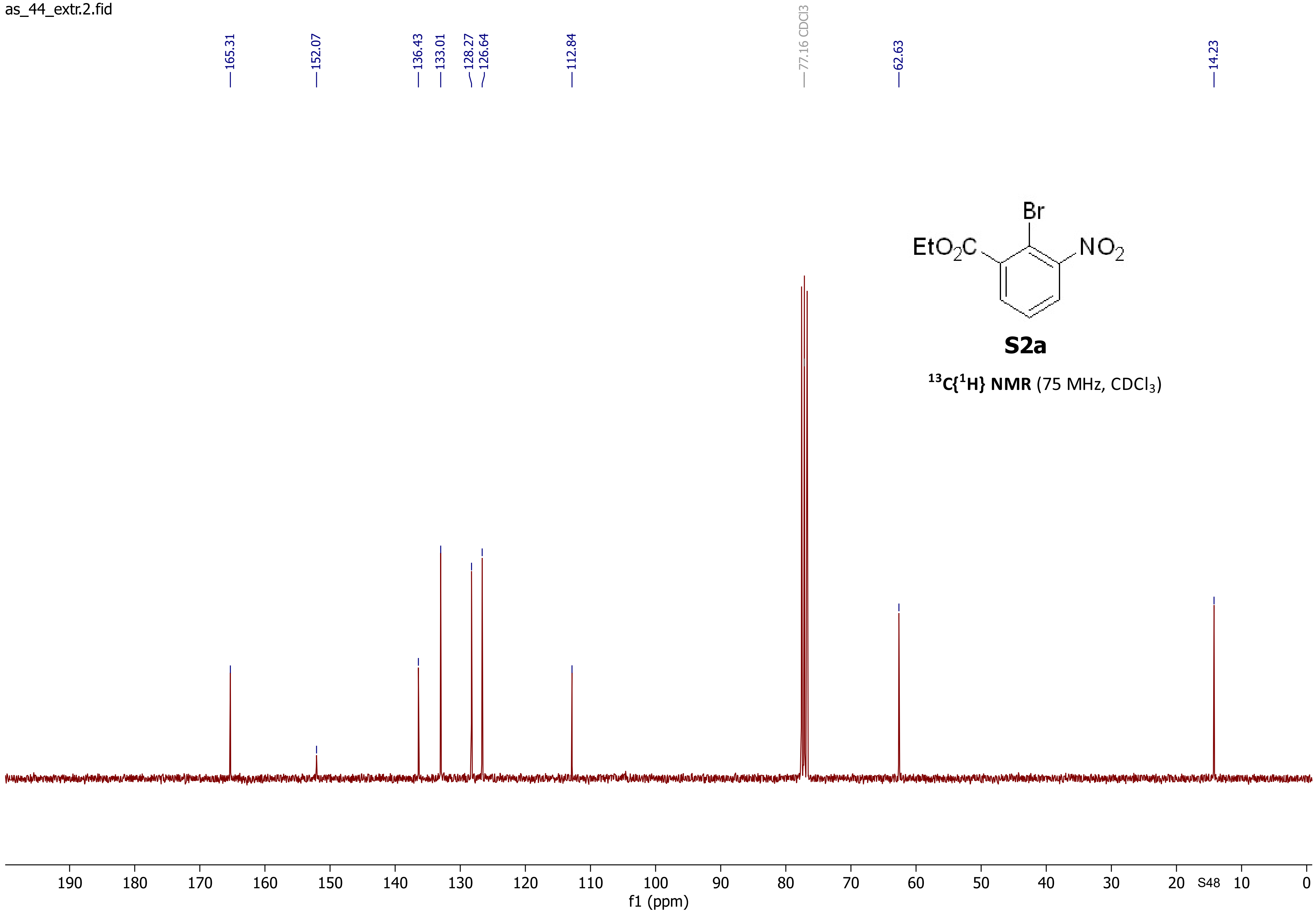
as_44_extr.2.fid
165.31
152.07
136.43
133.01
128.27
126.64
112.84
77.16 CDCl3
62.63
14.23
Br
EtO2C
NO2
S2a
13C{1H} NMR (75 MHz, CDCl3)
190 180 170 160 150 140 130 120 110 100 90 80 70 60 50 40 30 20 10 0
f1 (ppm)

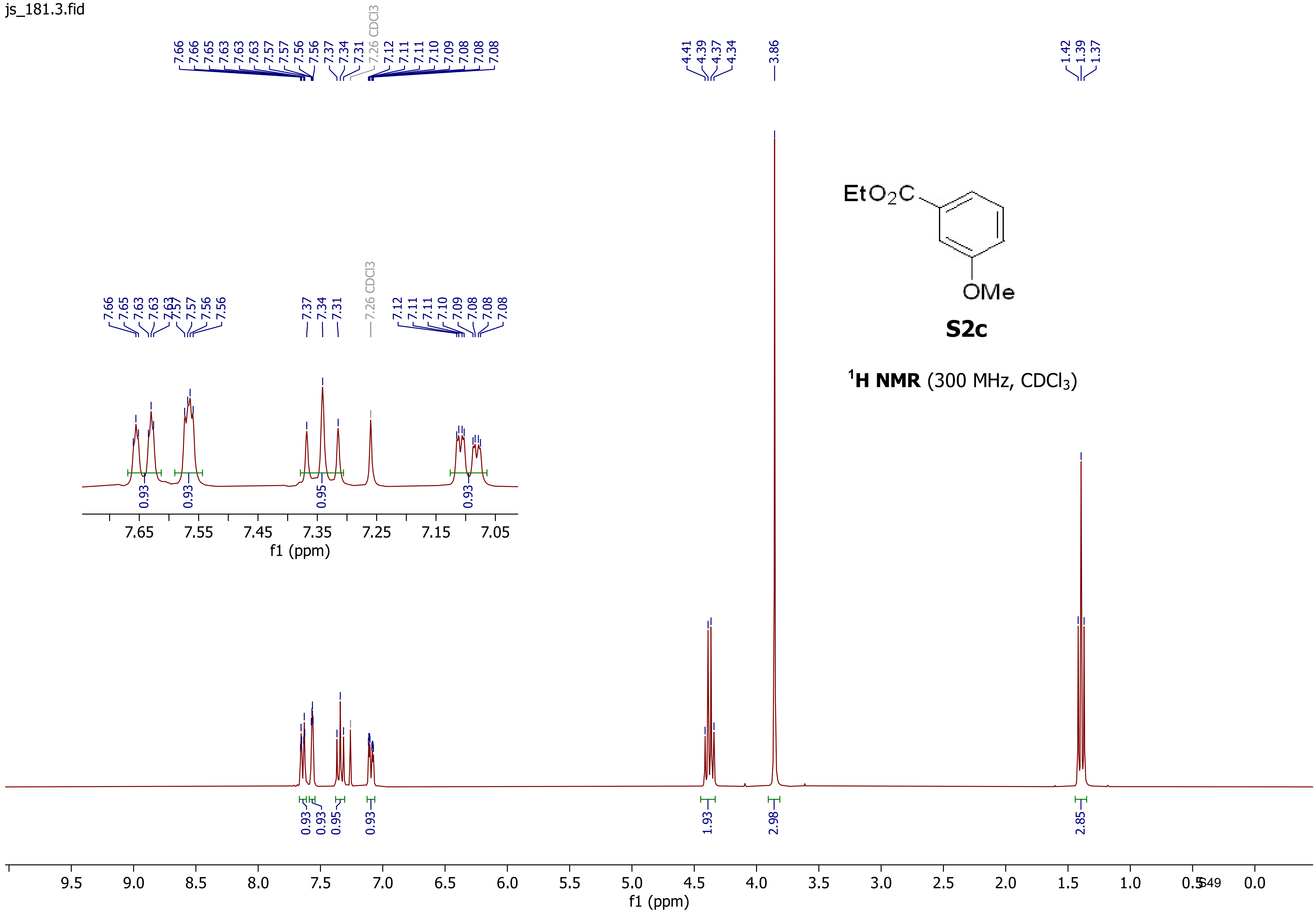

S2c

^{1}H NMR (300 MHz, $CDCl_3$)

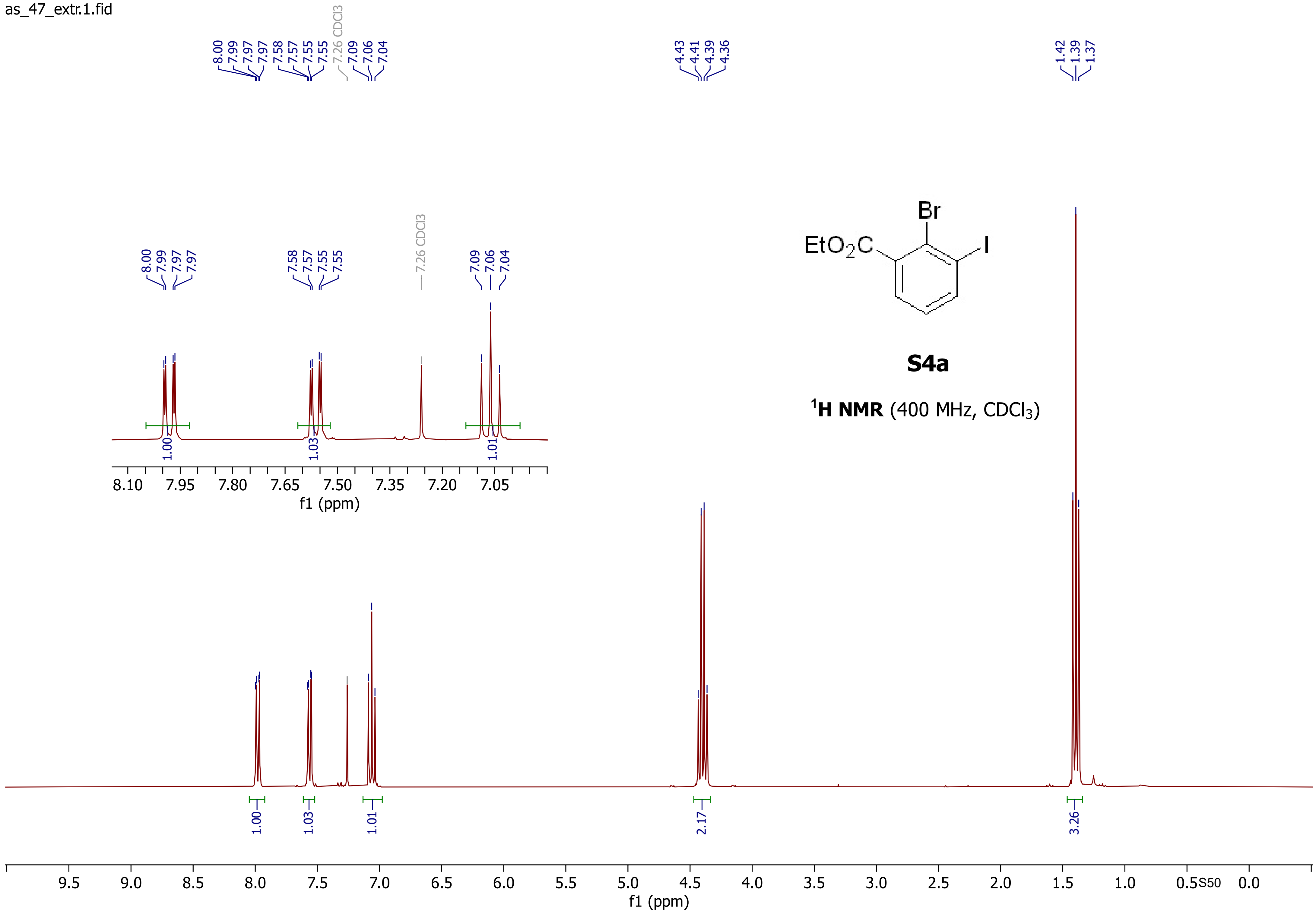

S4a

^{1}H NMR (400 MHz, $CDCl_3$)

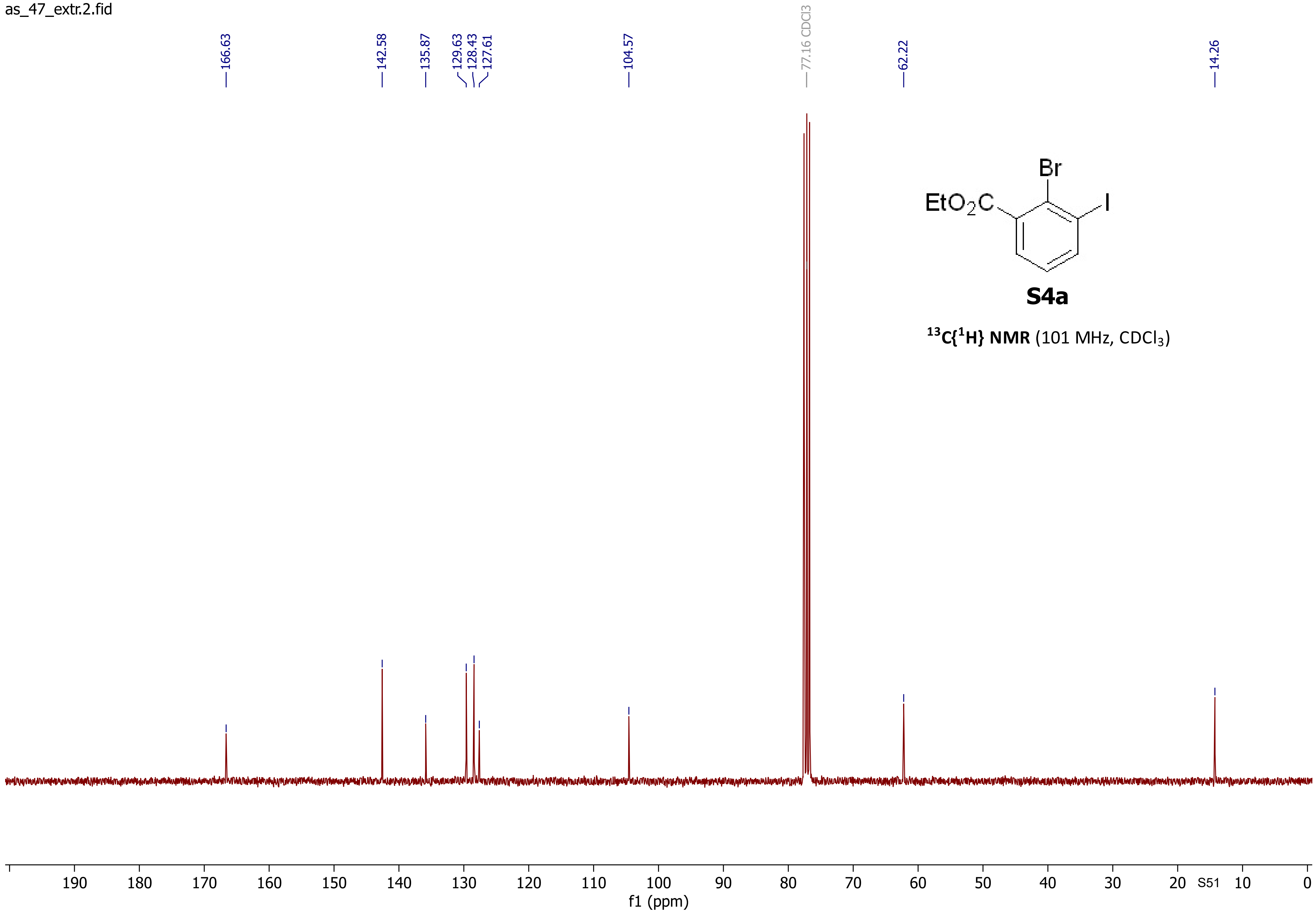
as_47_extr.2.fid
166.63
142.58
135.87
129.63
128.43
127.61
104.57
77.16 CDCl3
62.22
14.26
Br
EtO_2C
I
S4a
$^{13}C\{^1H\}$ NMR (101 MHz, $CDCl_3$)
190 180 170 160 150 140 130 120 110 100 90 80 70 60 50 40 30 20 10 0
f1 (ppm)

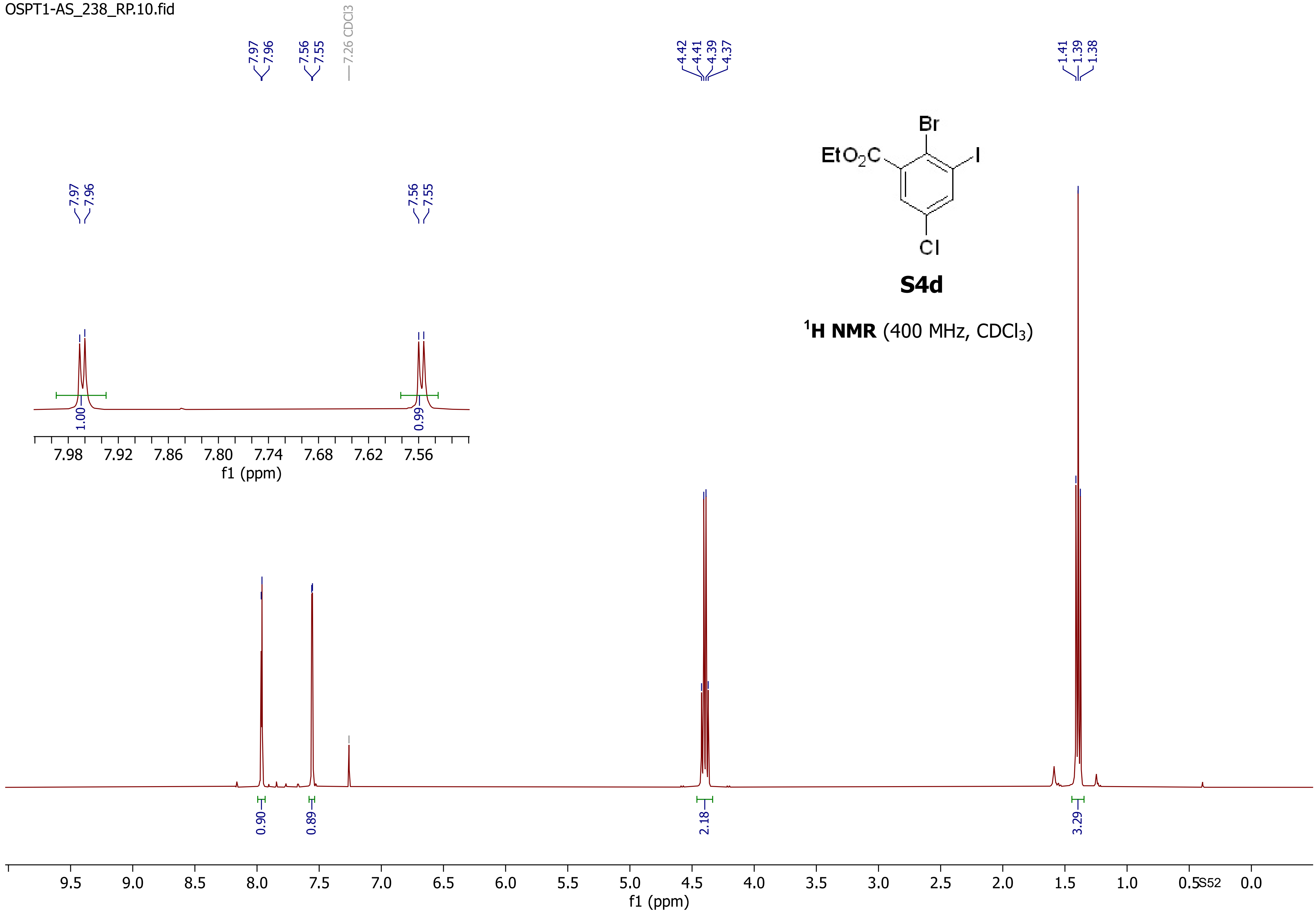

S4d

^{1}H NMR (400 MHz, $CDCl_3$)

OSPT1-AS_238_RP.11.fid

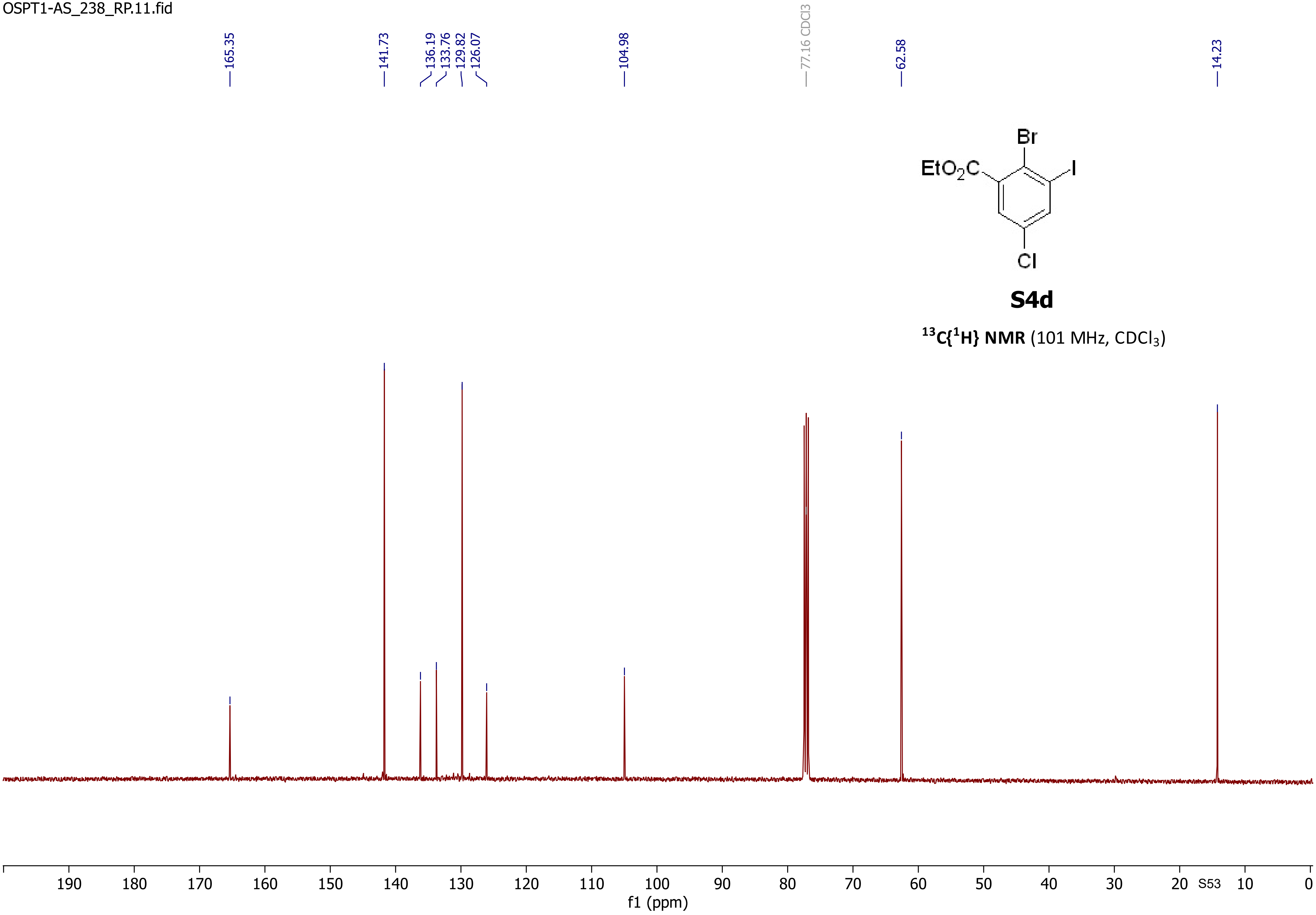

S4d

$^{13}C\{^{1}H\}$ NMR (101 MHz, $CDCl_3$)

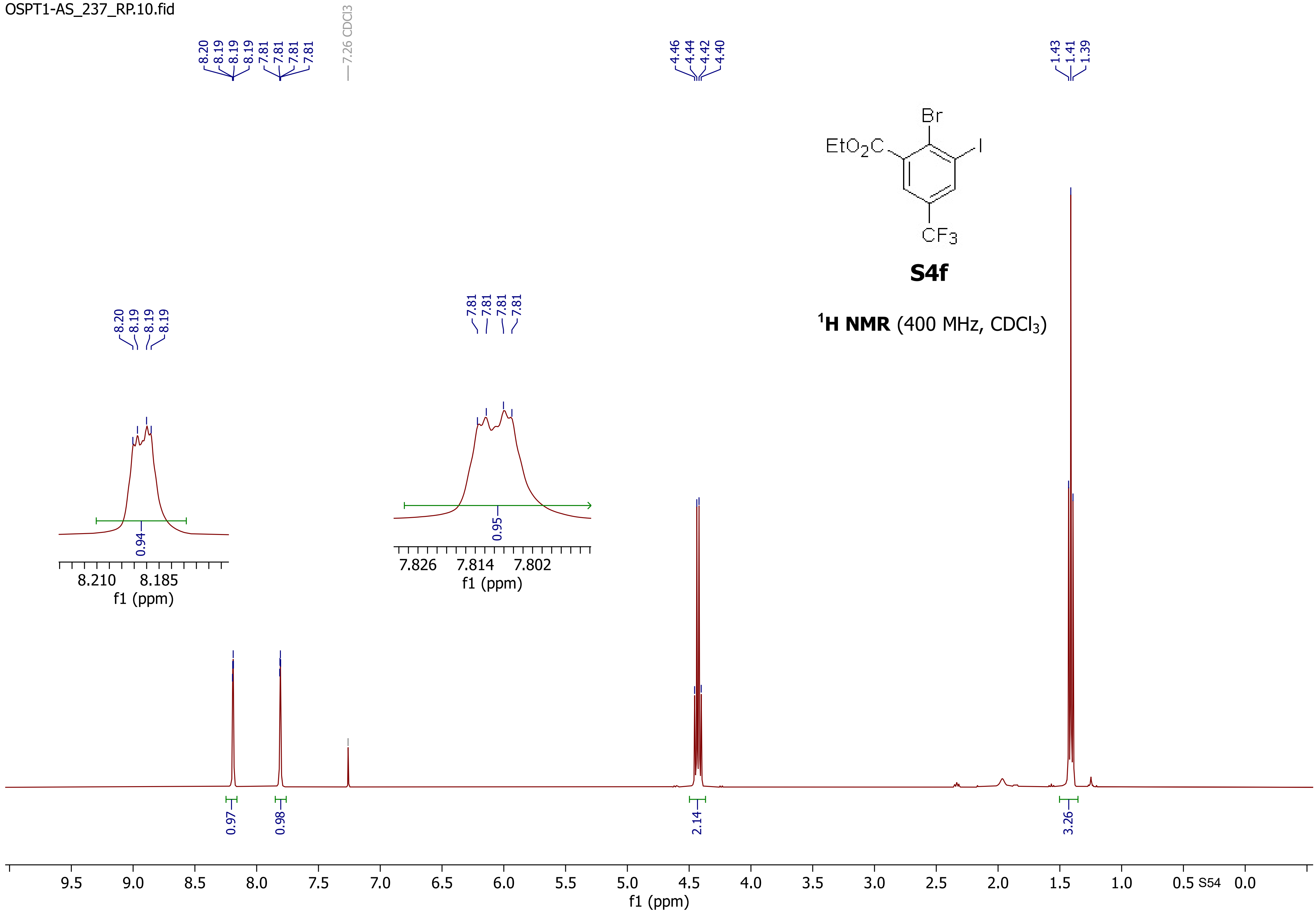
OSPT1-AS_237_RP.10.fid
8.20 8.19 8.19 8.19 7.81 7.81 7.81 7.81
7.26 CDCl3
4.46 4.44 4.42 4.40
1.43 1.41 1.39
Br
EtO2C
I
CF3
S4f
1H NMR (400 MHz, CDCl3)
0.94
8.210 8.185
f1 (ppm)
0.95
7.826 7.814 7.802
f1 (ppm)
0.97
0.98
2.14
3.26
9.5 9.0 8.5 8.0 7.5 7.0 6.5 6.0 5.5 5.0 4.5 4.0 3.5 3.0 2.5 2.0 1.5 1.0 0.5 0.0
f1 (ppm)

OSPT1-AS_237_RP.11.fid

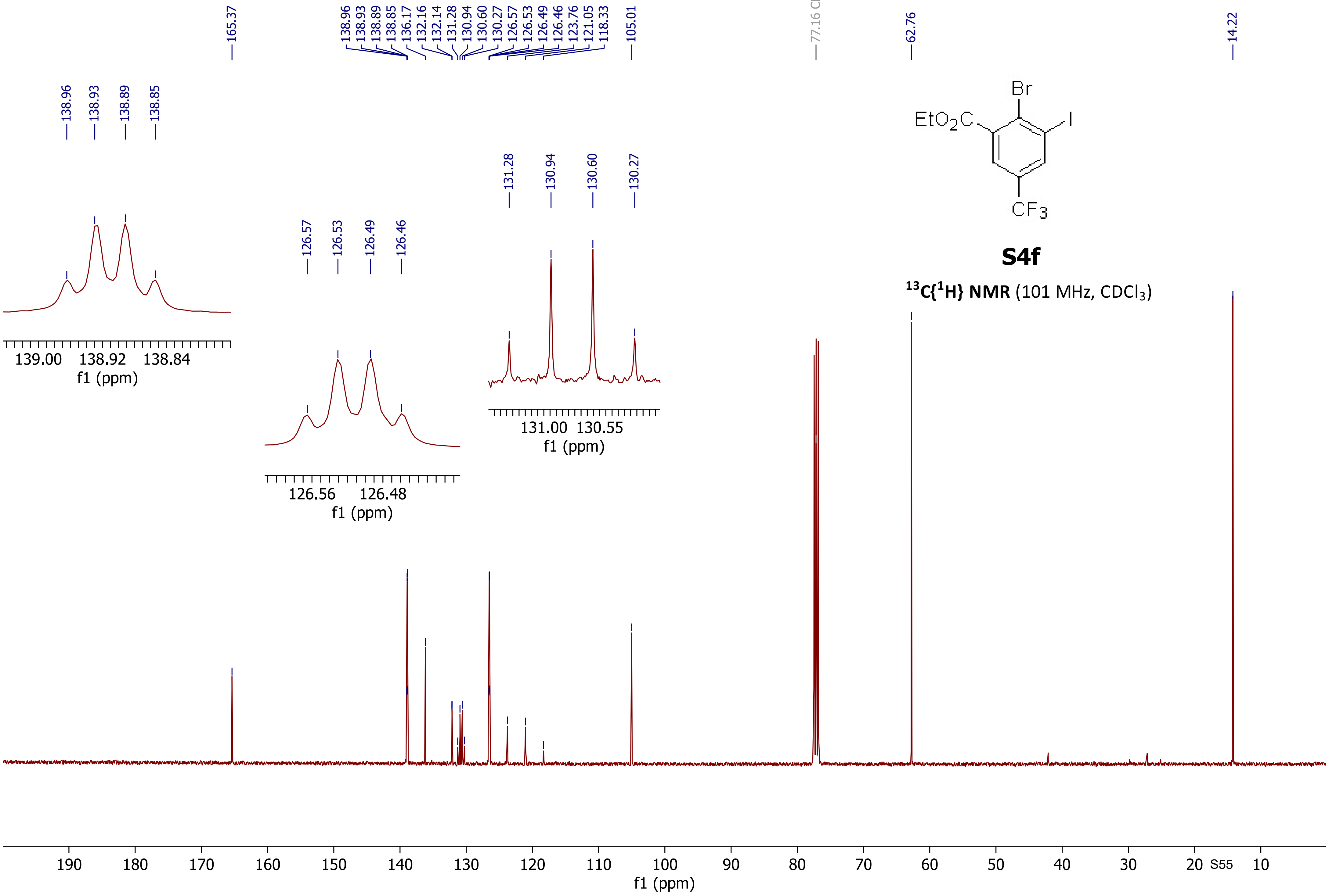

S4f

^{13}**C{**^{1}**H} NMR** (101 MHz, $CDCl_3$)

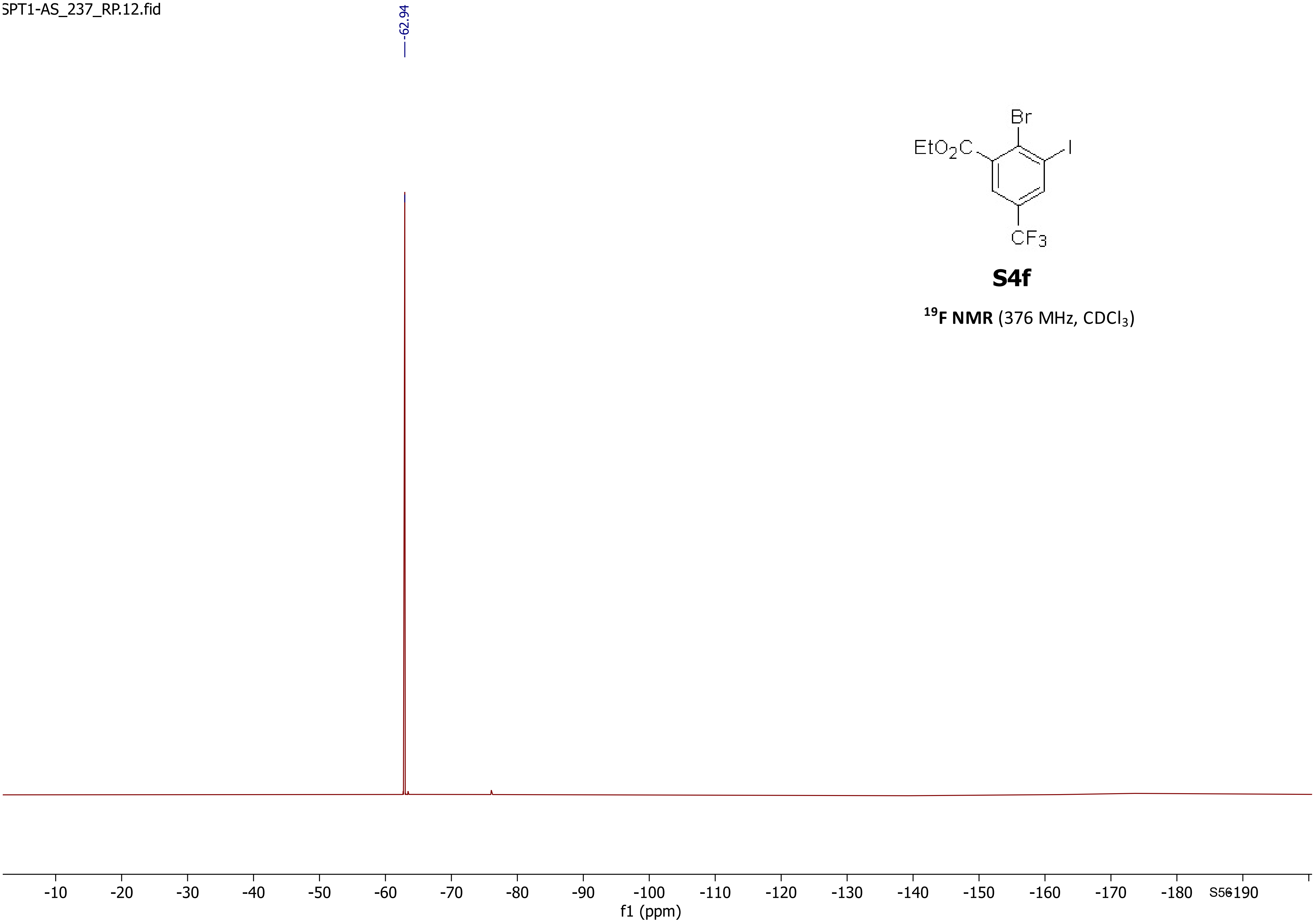

S4f

^{19}F NMR (376 MHz, $CDCl_3$)

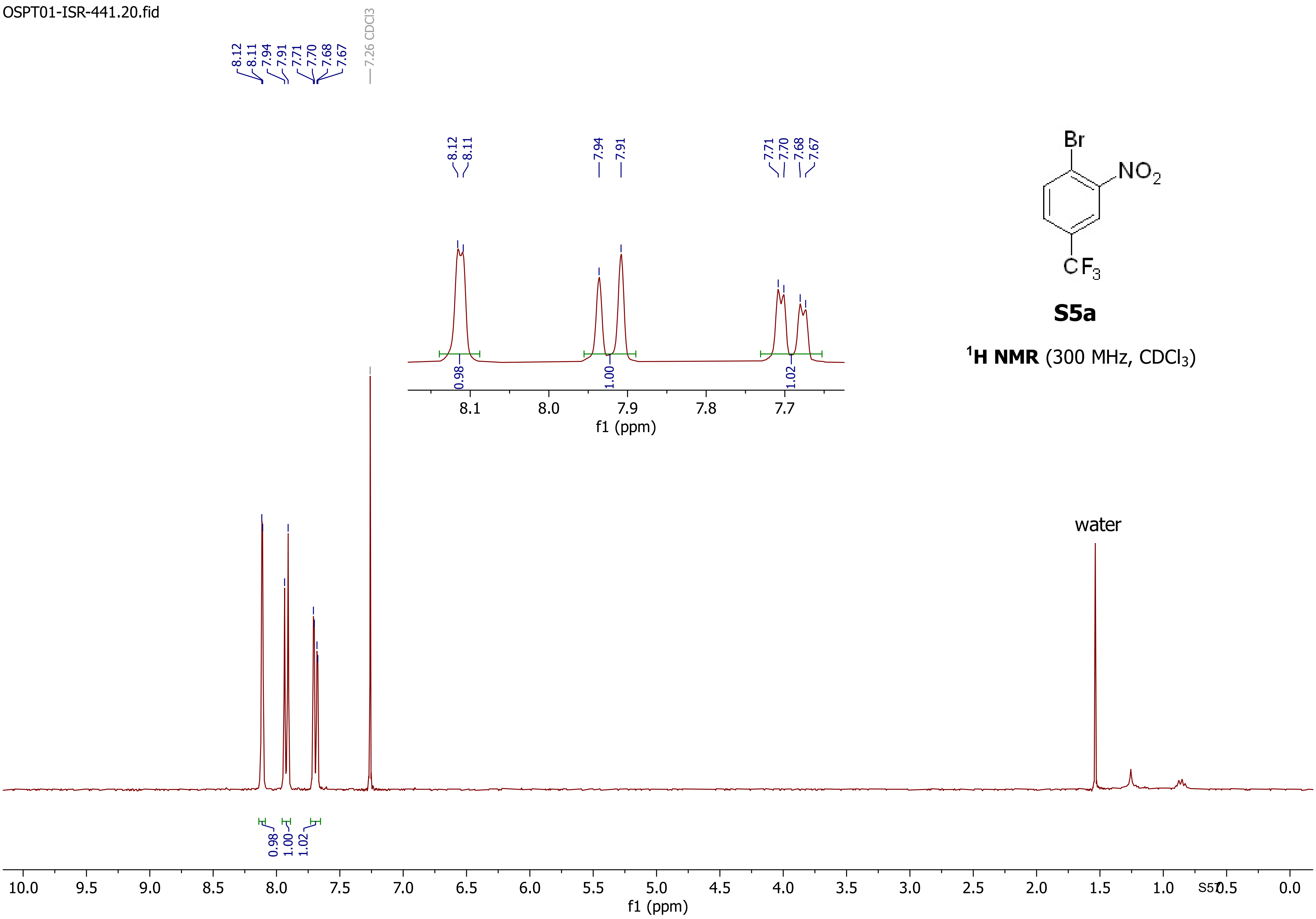

S5a

^{1}H NMR (300 MHz, $CDCl_3$)

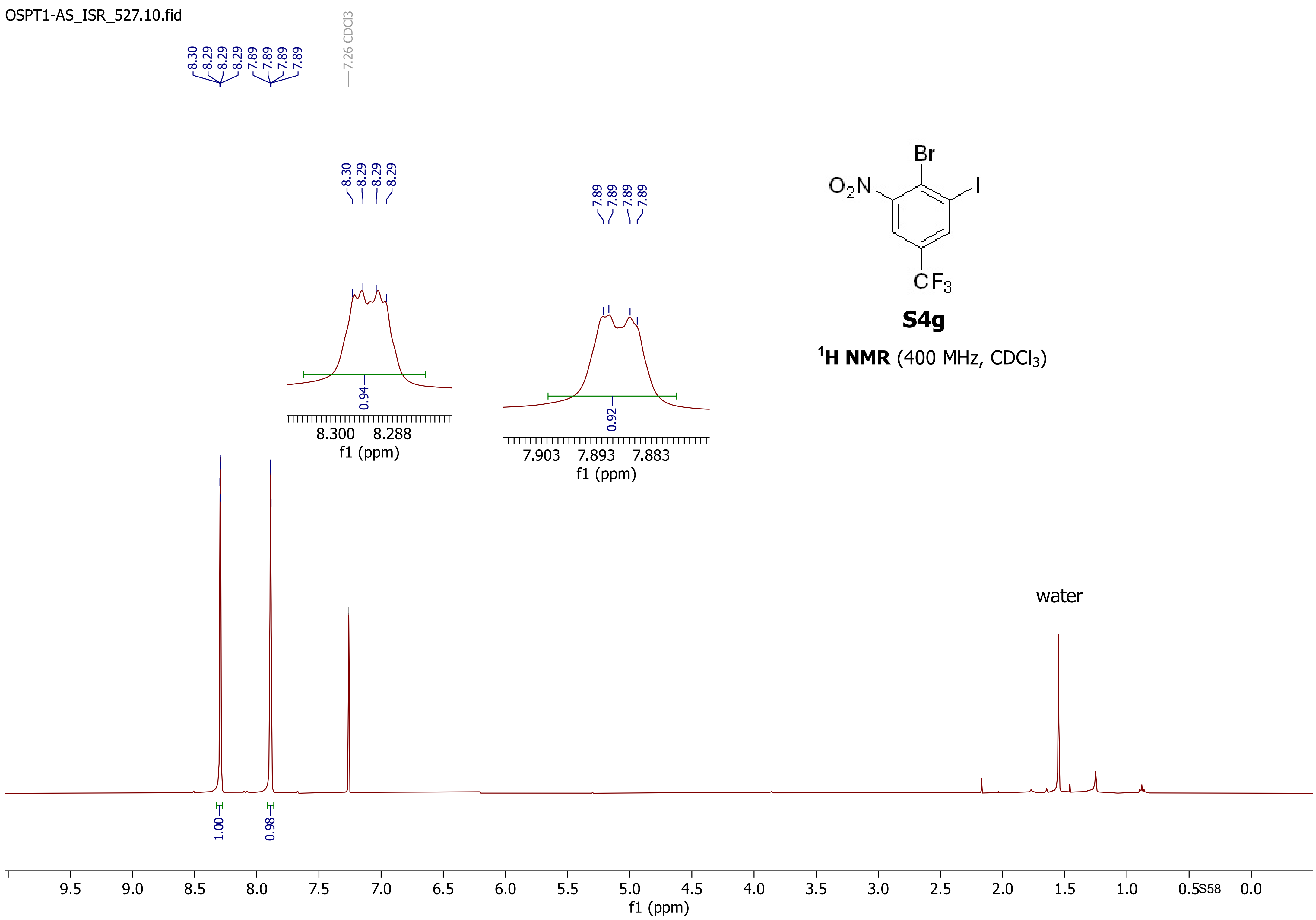

OSPT1-AS_ISR_527.10.fid
8.30
8.29
8.29
8.29
7.89
7.89
7.89
7.89
7.26 CDCl3
0.94
8.300
8.288
f1 (ppm)
0.92
7.903
7.893
7.883
f1 (ppm)
Br
O2N
I
CF3
S4g
1H NMR (400 MHz, CDCl3)
water
1.00
0.98
9.5
9.0
8.5
8.0
7.5
7.0
6.5
6.0
5.5
5.0
4.5
4.0
3.5
3.0
2.5
2.0
1.5
1.0
0.5
0.0
f1 (ppm)

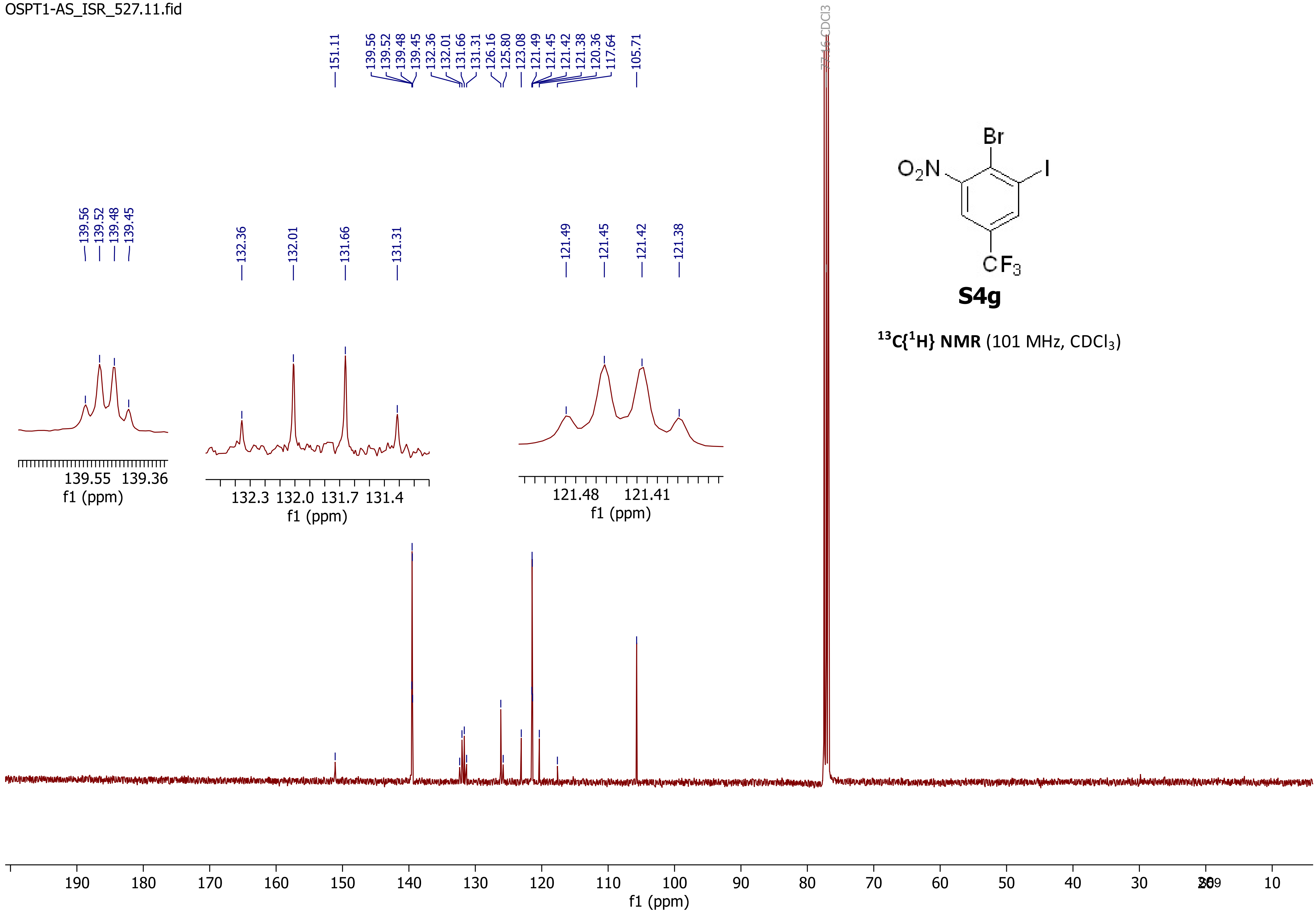
OSPT1-AS_ISR_527.11.fid
151.11
139.56
139.52
139.48
139.45
132.36
132.01
131.66
131.31
126.16
125.80
123.08
121.49
121.45
121.42
121.38
120.36
117.64
105.71
77.16 CDCl3
139.55 139.36
f1 (ppm)
132.3 132.0 131.7 131.4
f1 (ppm)
121.48 121.41
f1 (ppm)
Br
O2N
I
CF3
S4g
13C{1H} NMR (101 MHz, CDCl3)
190 180 170 160 150 140 130 120 110 100 90 80 70 60 50 40 30 20 10
f1 (ppm)

OSPT1-AS_ISR_527.12.1.1r

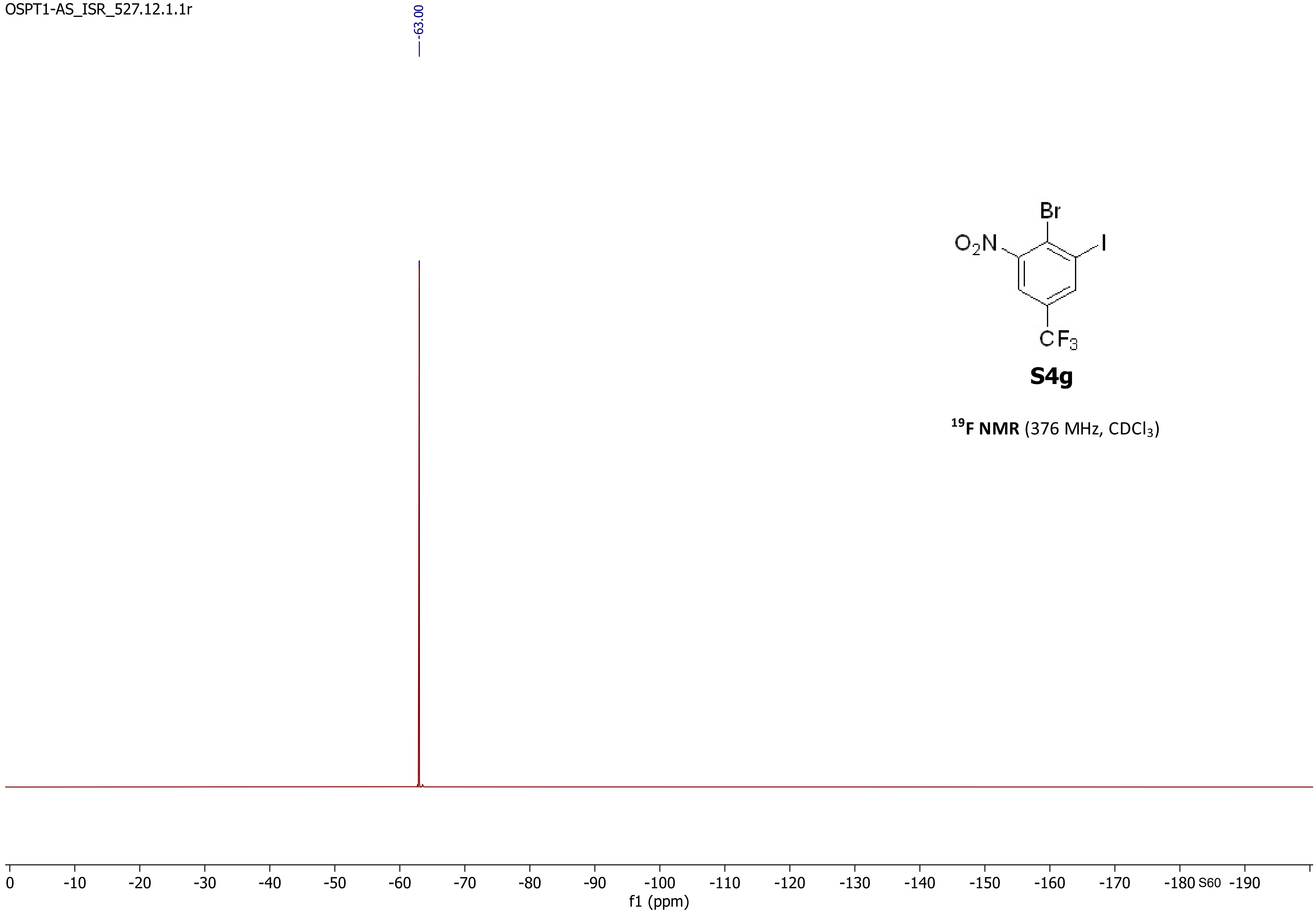

S4g

^{19}F NMR (376 MHz, $CDCl_3$)

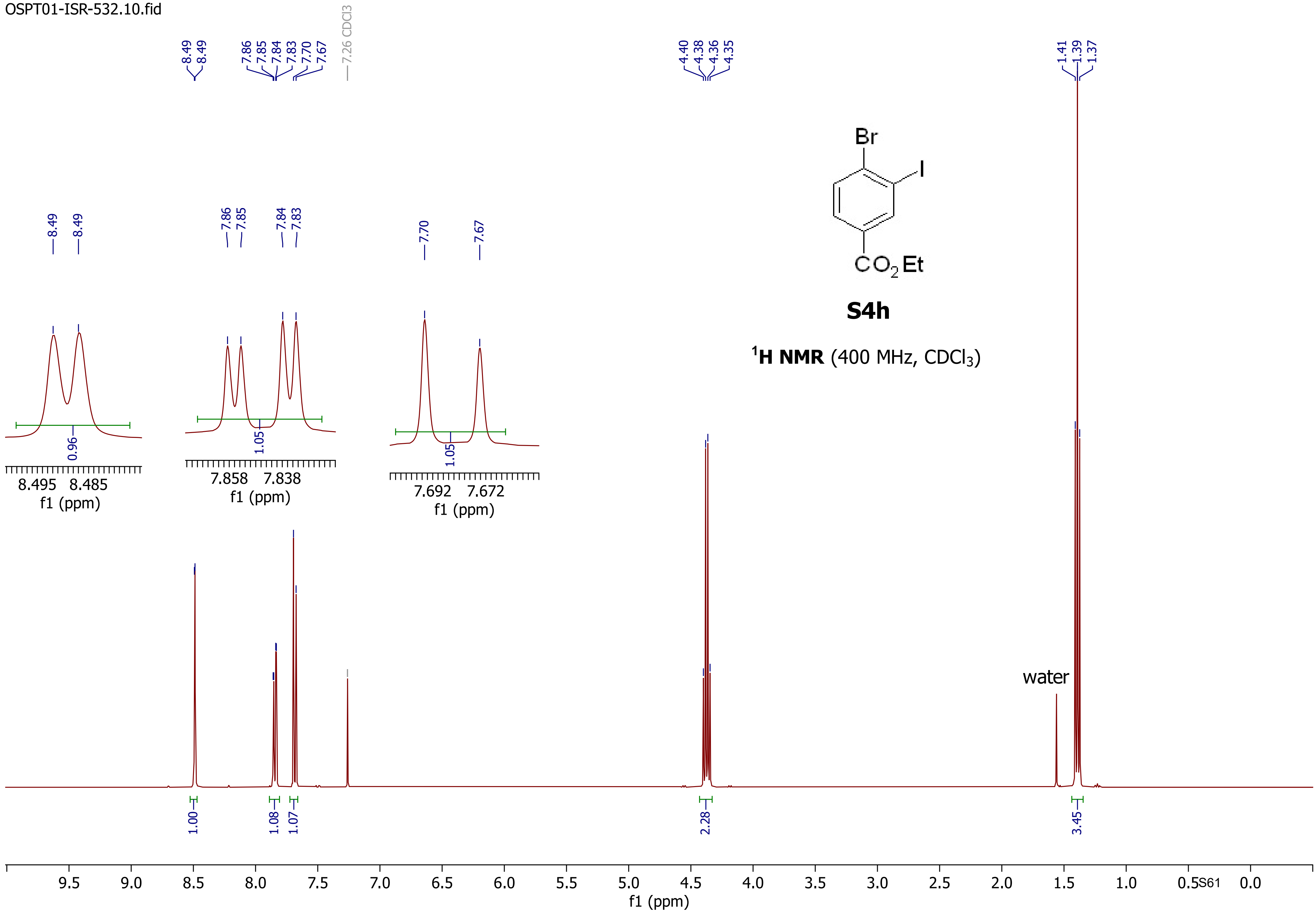
OSPT01-ISR-532.10.fid
8.49
8.49
7.86
7.85
7.84
7.83
7.70
7.67
7.26 CDCl3
4.40
4.38
4.36
4.35
1.41
1.39
1.37
Br
I
CO2Et
S4h
1H NMR (400 MHz, CDCl3)
0.96
1.05
1.05
8.495
8.485
7.858
7.838
7.692
7.672
f1 (ppm)
water
1.00
1.08
1.07
2.28
3.45
9.5
9.0
8.5
8.0
7.5
7.0
6.5
6.0
5.5
5.0
4.5
4.0
3.5
3.0
2.5
2.0
1.5
1.0
0.5
0.0

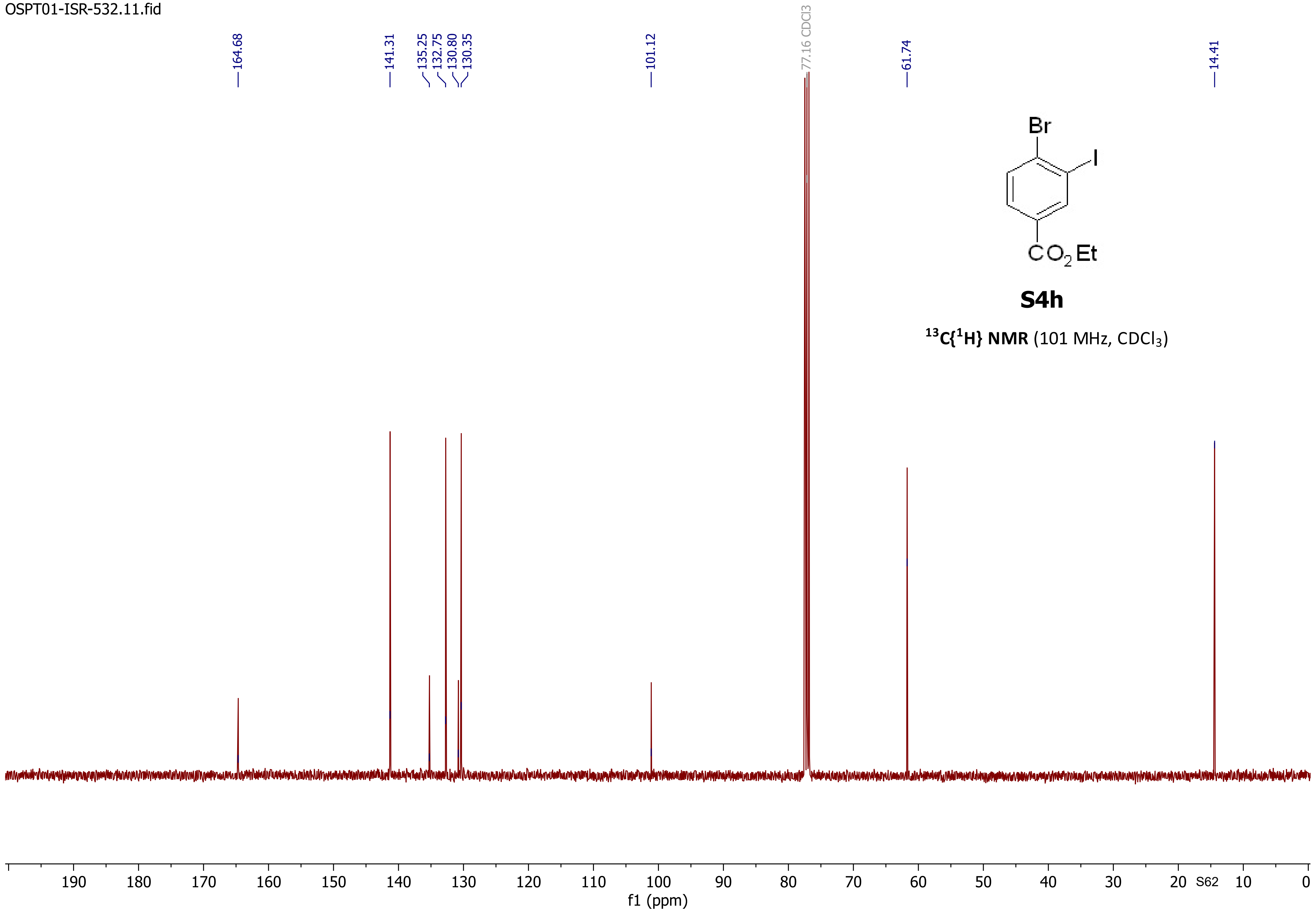
OSPT01-ISR-532.11.fid
164.68
141.31
135.25
132.75
130.80
130.35
101.12
77.16 CDCl3
61.74
14.41
Br
I
CO2Et
S4h
13C{1H} NMR (101 MHz, CDCl3)
190 180 170 160 150 140 130 120 110 100 90 80 70 60 50 40 30 20 10 0
f1 (ppm)

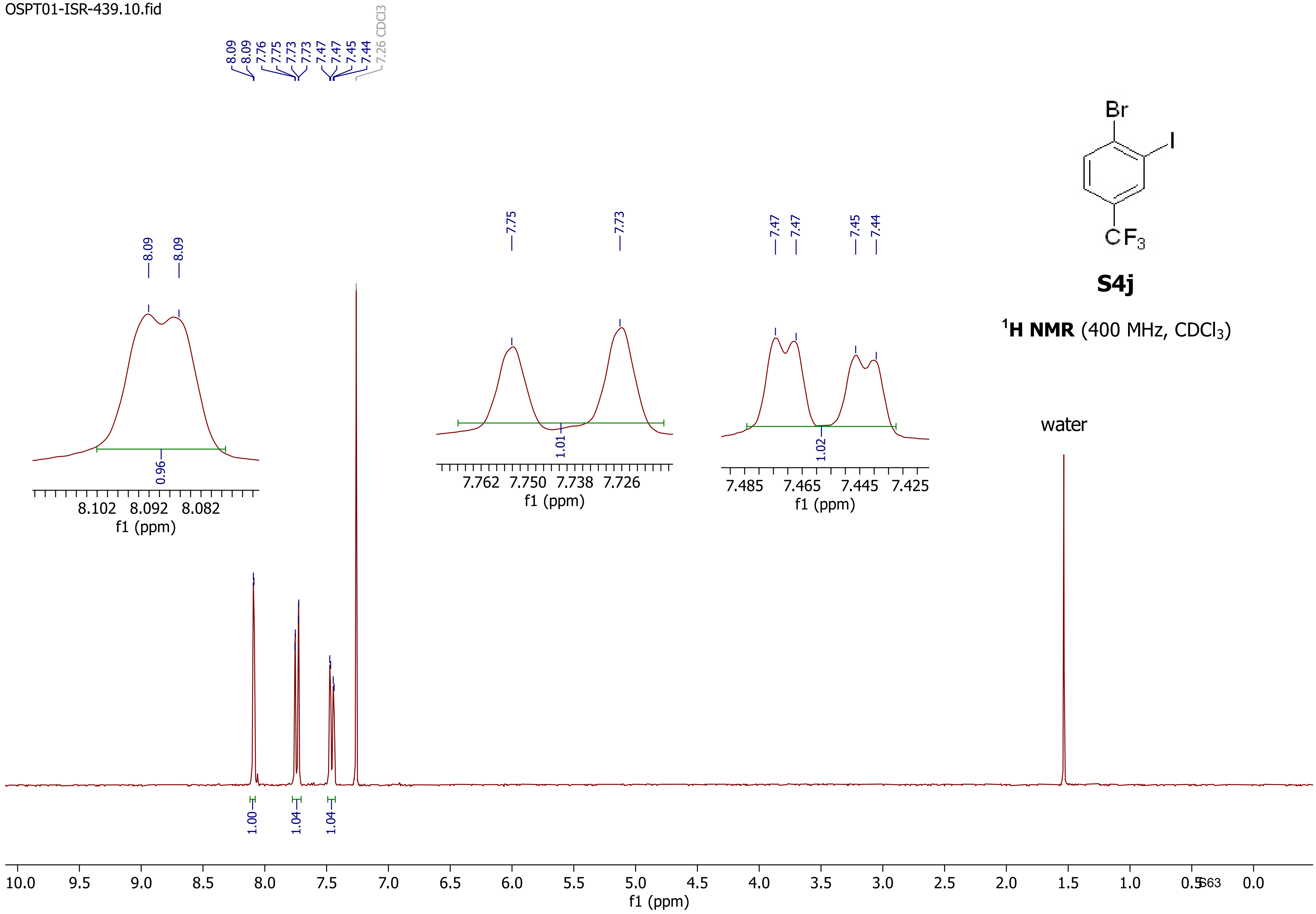
OSPT01-ISR-439.10.fid
8.09
8.09
7.76
7.75
7.73
7.73
7.47
7.47
7.45
7.44
7.26 CDCl3
Br
I
CF3
S4j
1H NMR (400 MHz, CDCl3)
water
0.96
1.01
1.02
1.00
1.04
1.04
f1 (ppm)

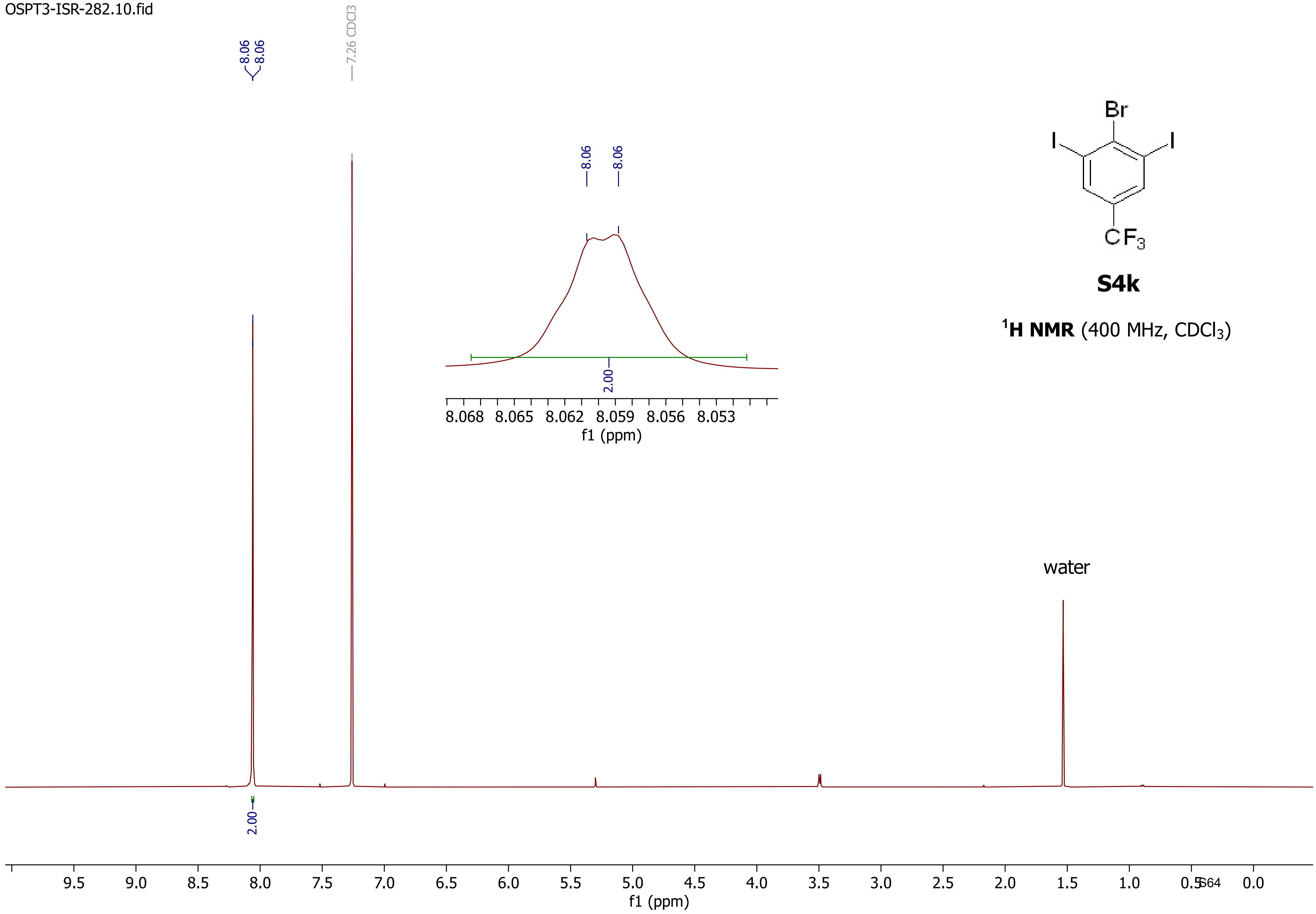

S4k

^{1}H NMR (400 MHz, $CDCl_3$)

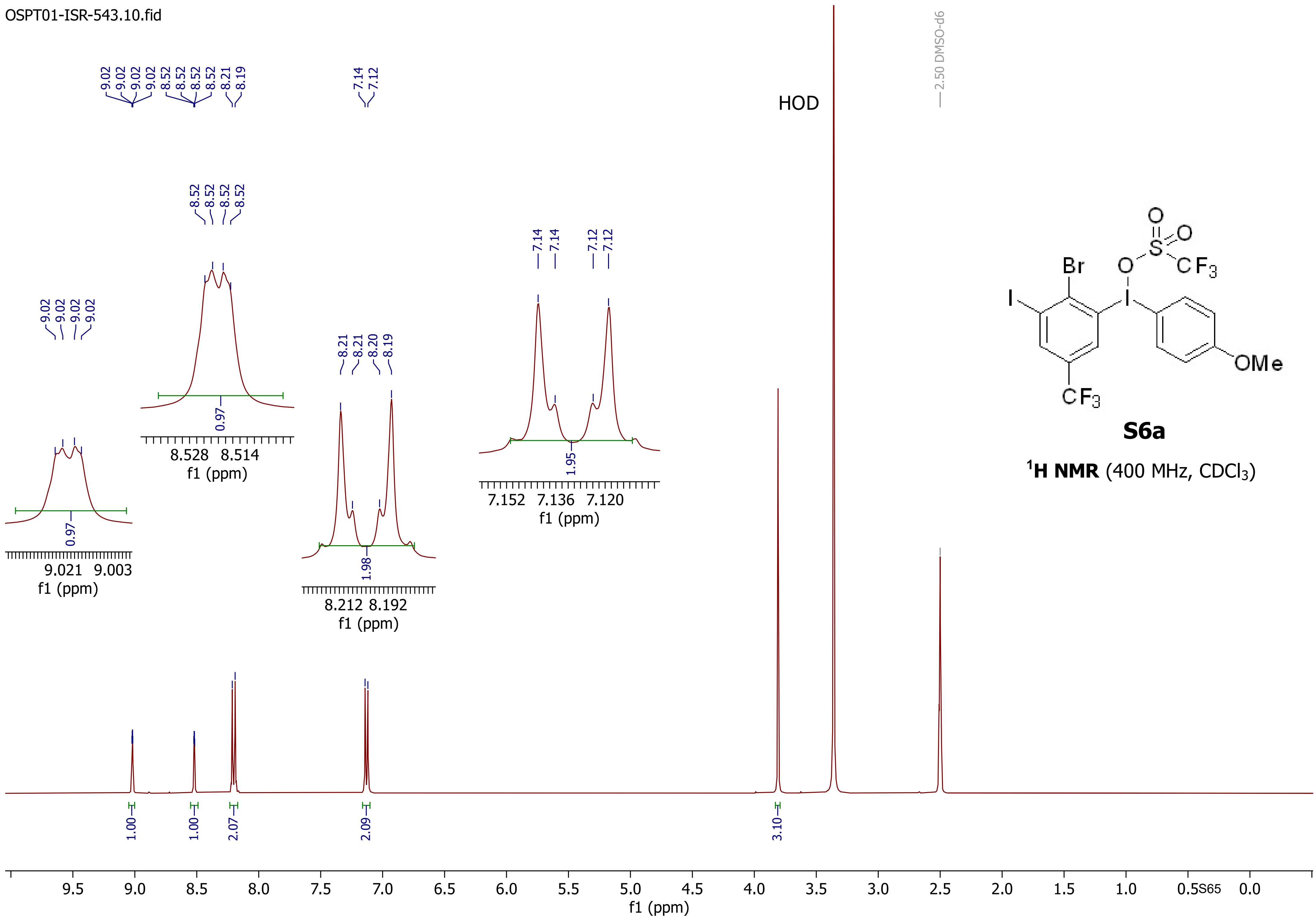

S6a

[1]**H NMR** (400 MHz, $CDCl_3$)

OSPT01-ISR-543.11.fid

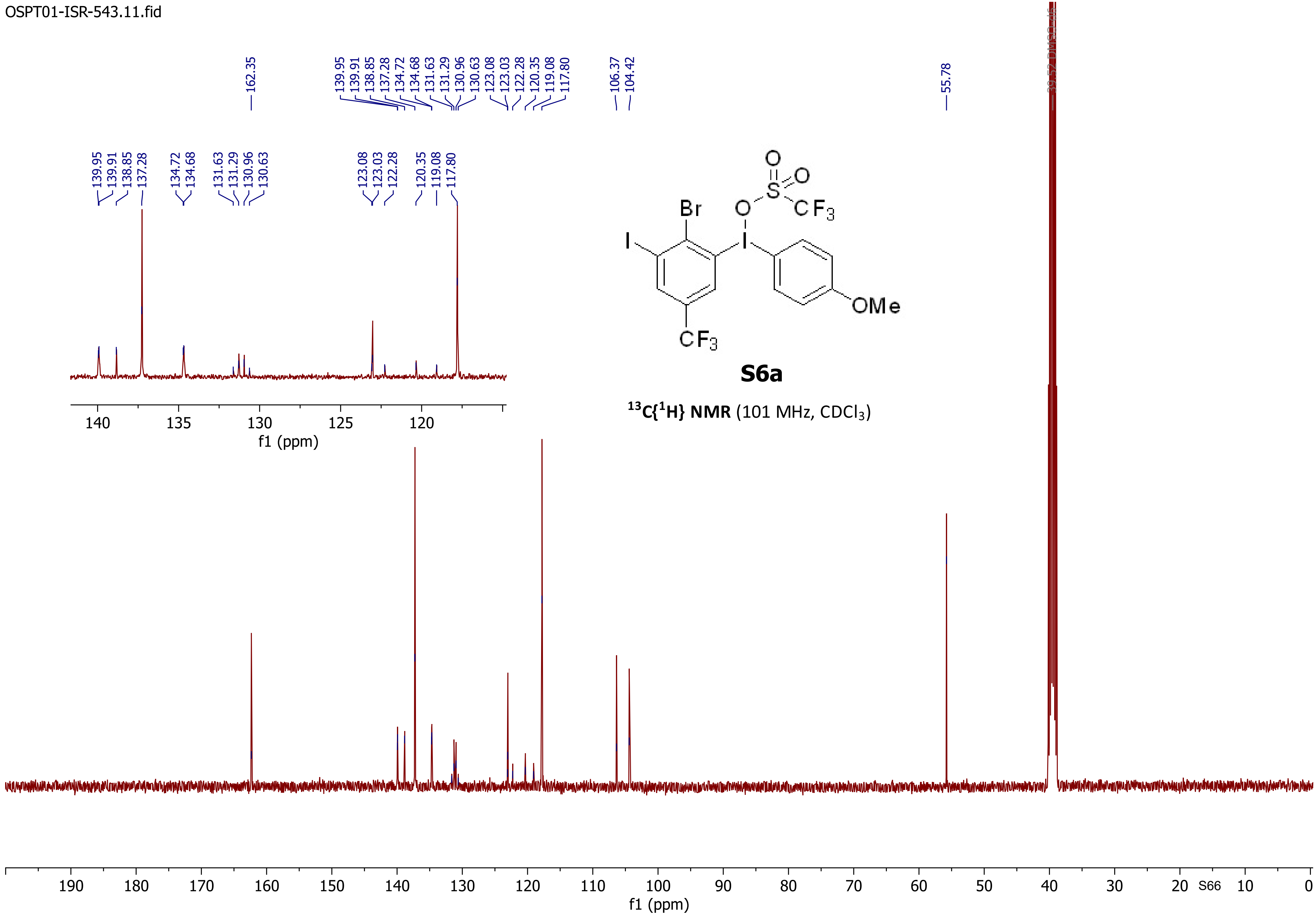

S6a

$^{13}C\{^{1}H\}$ NMR (101 MHz, $CDCl_3$)

OSPT01-ISR-543.12.1.1r

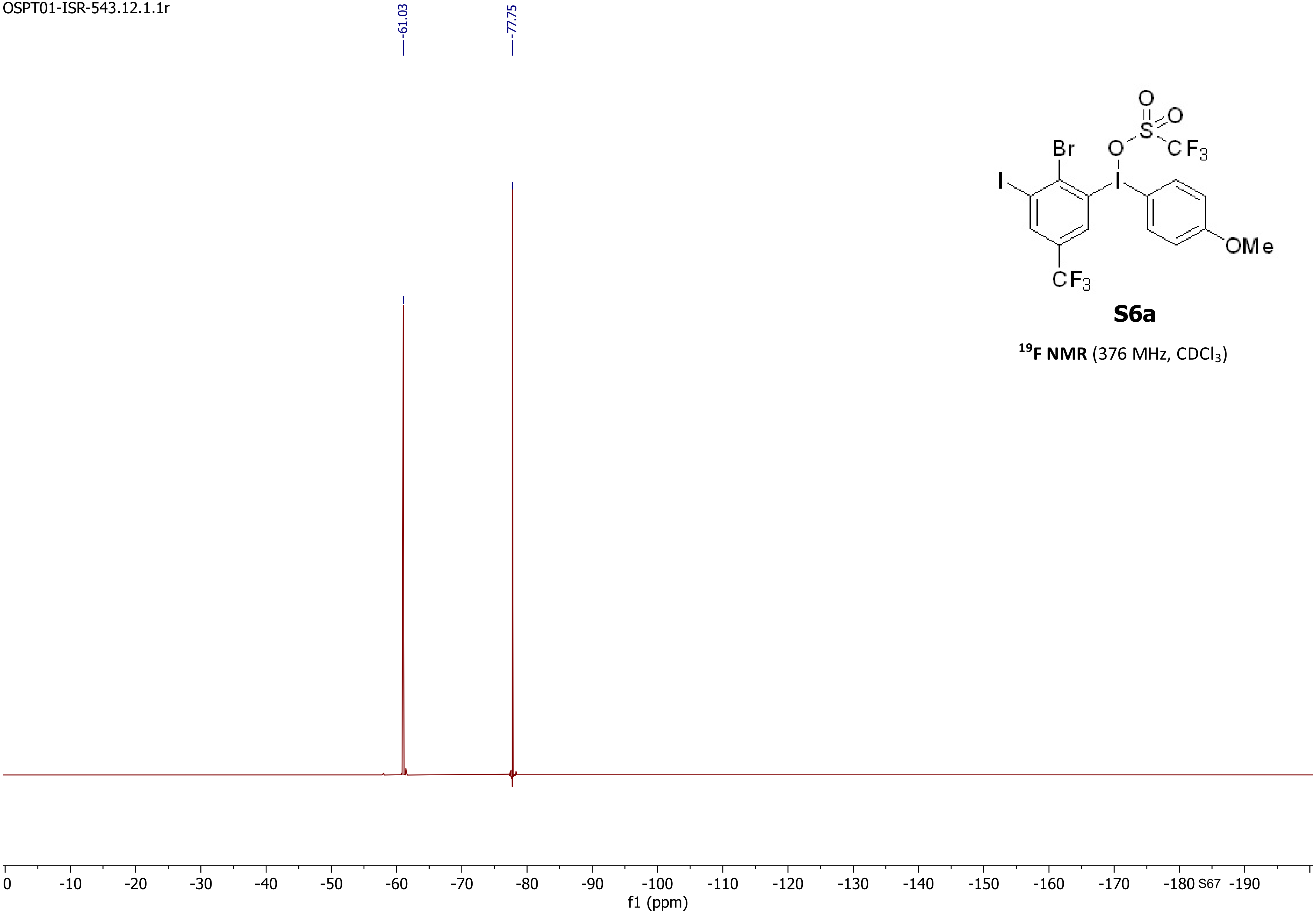

S6a

^{19}F NMR (376 MHz, $CDCl_3$)

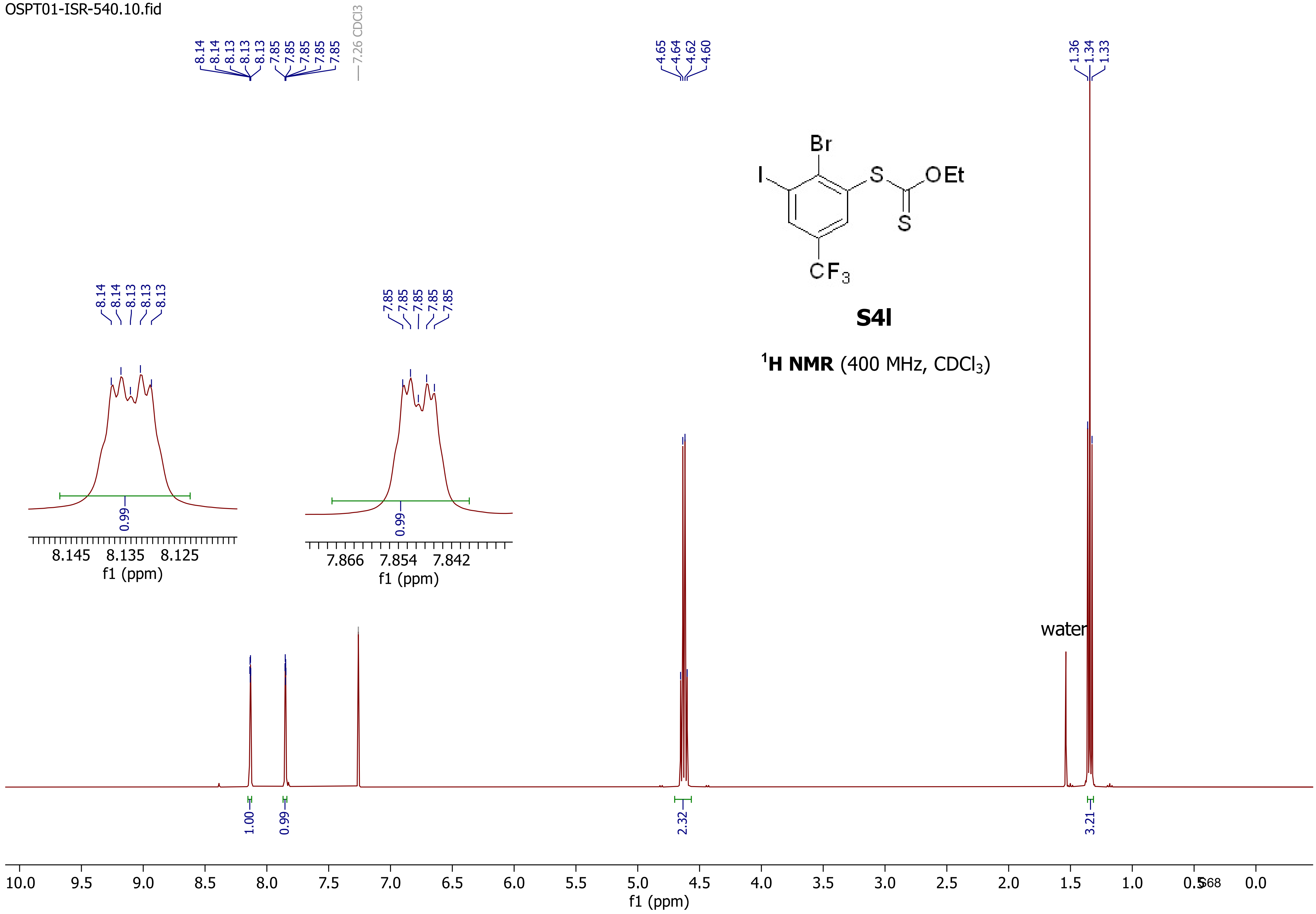
OSPT01-ISR-540.10.fid
8.14 8.14 8.13 8.13 8.13 7.85 7.85 7.85 7.85 7.85
7.26 CDCl3
4.65 4.64 4.62 4.60
1.36 1.34 1.33
Br
I
S
OEt
S
CF3
S4l
1H NMR (400 MHz, CDCl3)
0.99
8.145 8.135 8.125
f1 (ppm)
0.99
7.866 7.854 7.842
f1 (ppm)
water
1.00
0.99
2.32
3.21
10.0 9.5 9.0 8.5 8.0 7.5 7.0 6.5 6.0 5.5 5.0 4.5 4.0 3.5 3.0 2.5 2.0 1.5 1.0 0.5 0.0
f1 (ppm)

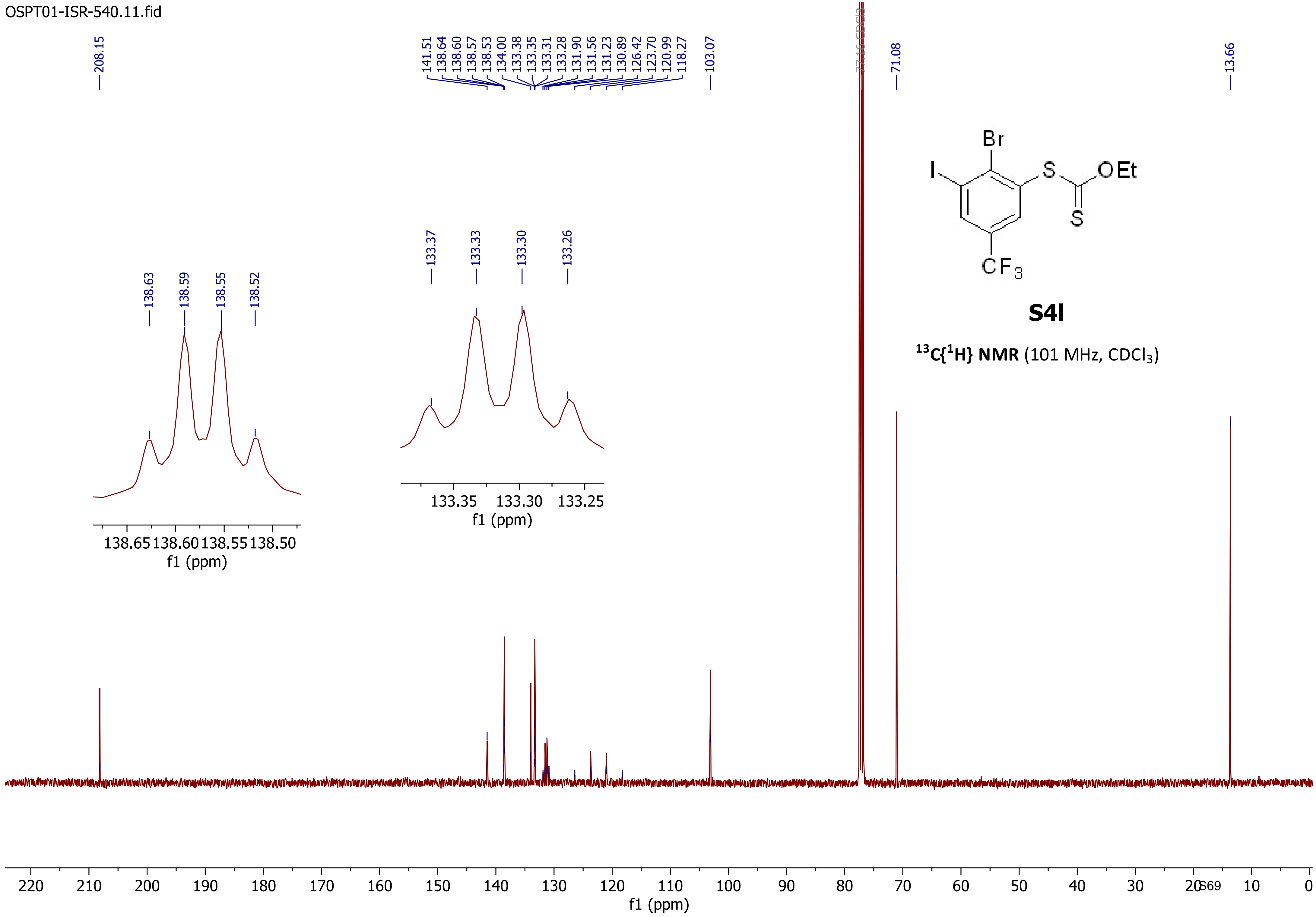

S4l

^{13}C{^{1}H} NMR (101 MHz, $CDCl_3$)

OSPT01-ISR-540.12.1.1r

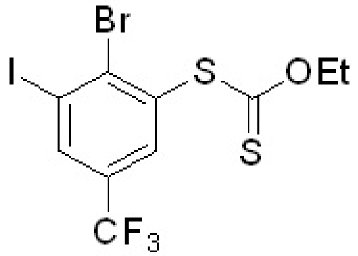

S4l

^{19}F NMR (376 MHz, $CDCl_3$)

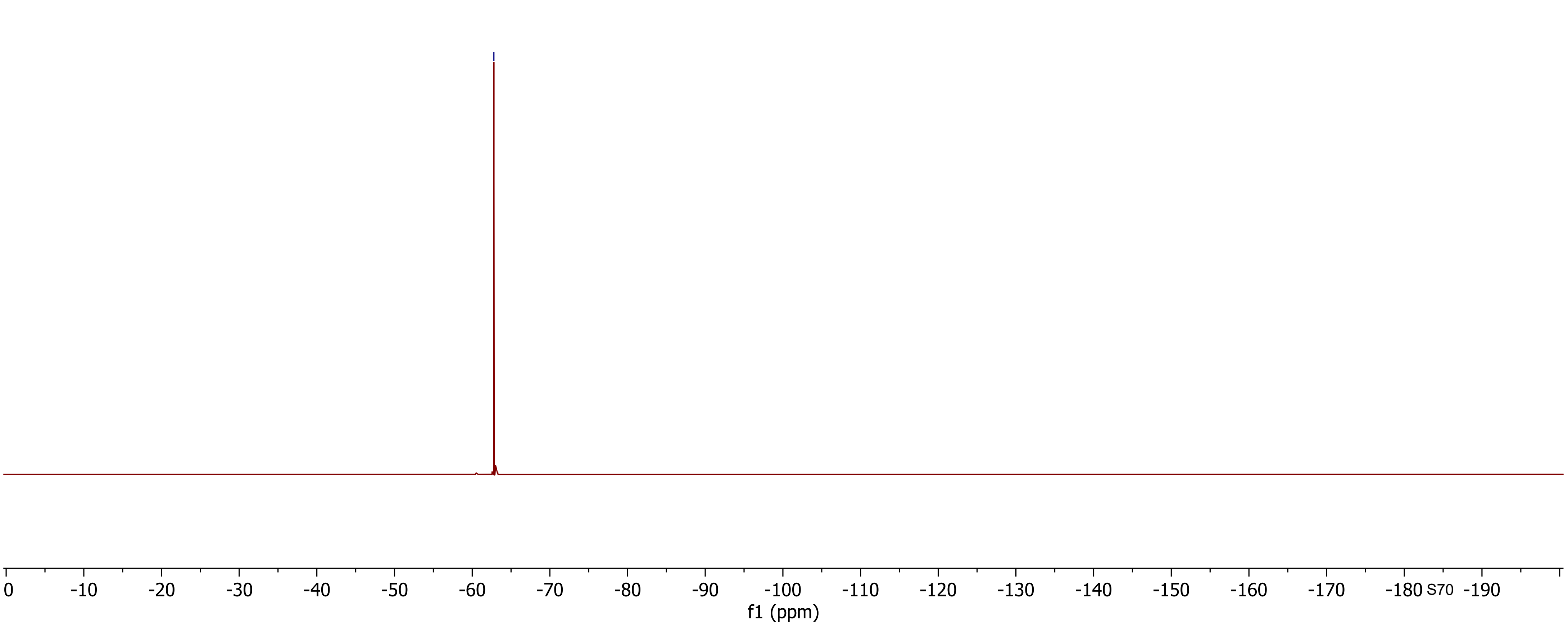

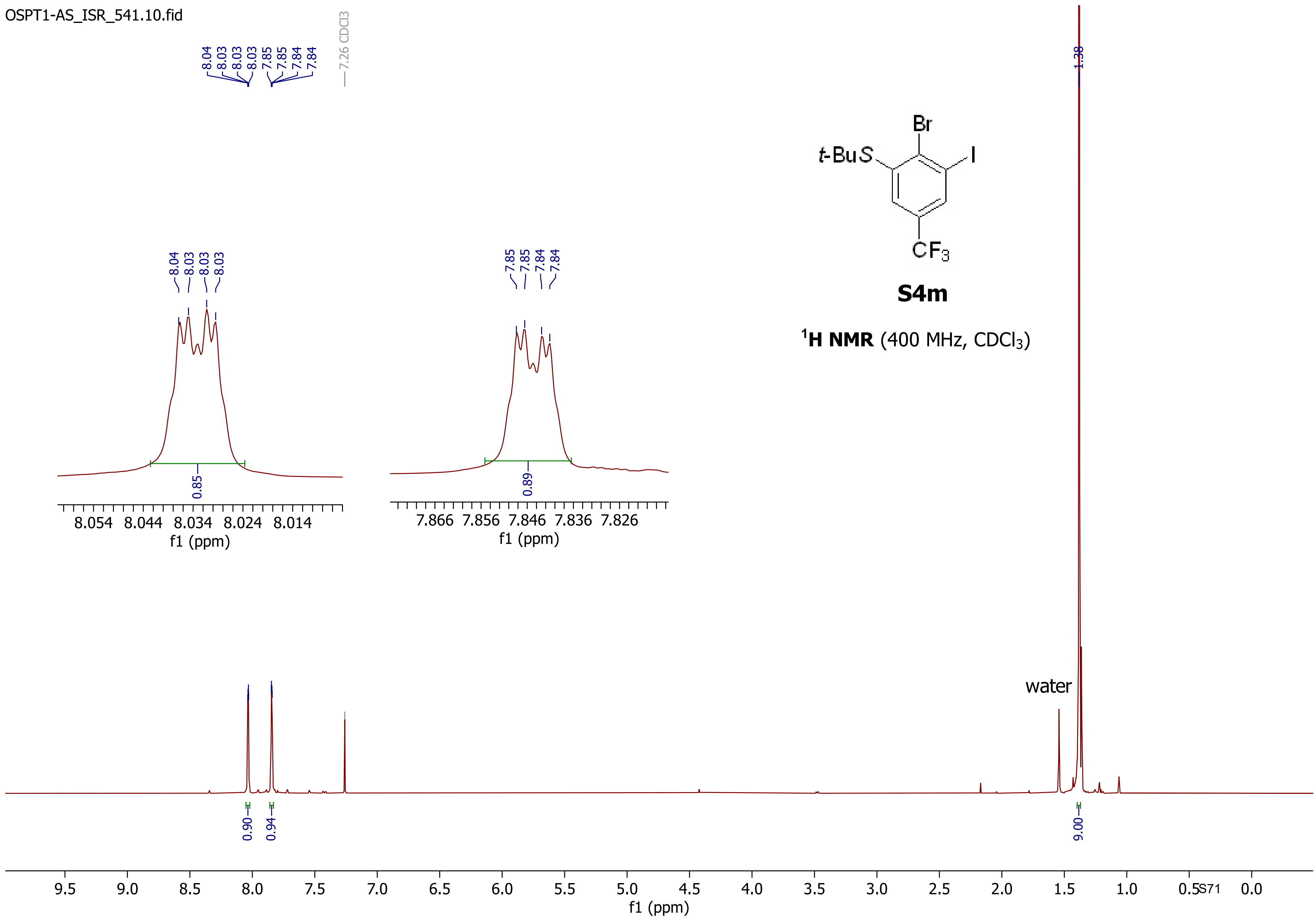
OSPT1-AS_ISR_541.10.fid
8.04
8.03
8.03
8.03
7.85
7.85
7.84
7.84
7.26 CDCl3
1.38
Br
t-BuS
I
CF3
S4m
1H NMR (400 MHz, CDCl3)
0.85
8.054 8.044 8.034 8.024 8.014
f1 (ppm)
0.89
7.866 7.856 7.846 7.836 7.826
f1 (ppm)
water
0.90
0.94
9.00
9.5 9.0 8.5 8.0 7.5 7.0 6.5 6.0 5.5 5.0 4.5 4.0 3.5 3.0 2.5 2.0 1.5 1.0 0.5 0.0
f1 (ppm)

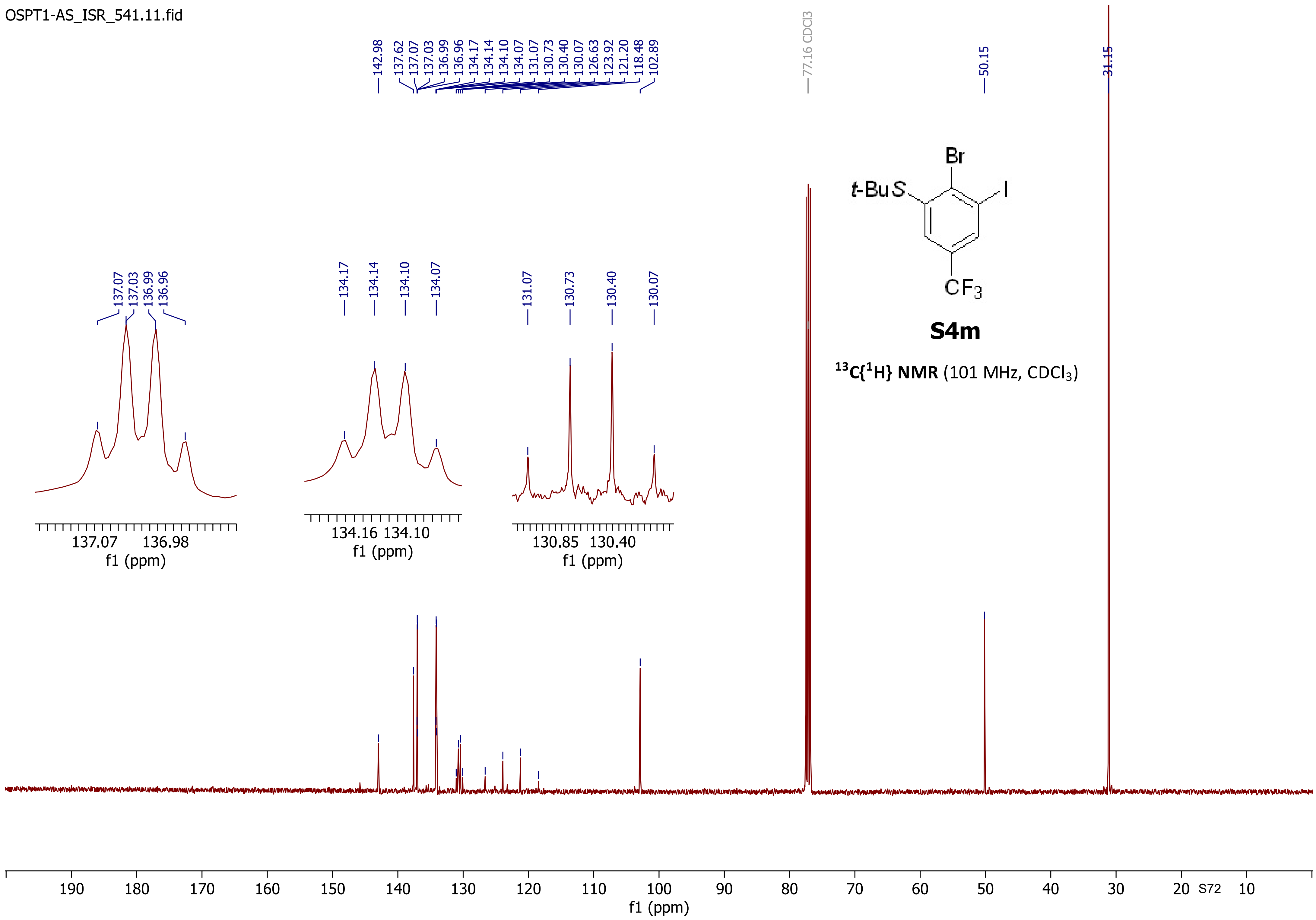
OSPT1-AS_ISR_541.11.fid
142.98
137.62
137.07
137.03
136.99
136.96
134.17
134.14
134.10
134.07
131.07
130.73
130.40
130.07
126.63
123.92
121.20
118.48
102.89
77.16 CDCl3
50.15
31.15
Br
t-BuS
I
CF3
S4m
13C{1H} NMR (101 MHz, CDCl3)
137.07
136.98
f1 (ppm)
134.16 134.10
f1 (ppm)
130.85 130.40
f1 (ppm)
190 180 170 160 150 140 130 120 110 100 90 80 70 60 50 40 30 20 10
f1 (ppm)

OSPT1-AS_ISR_541.12.1.1r

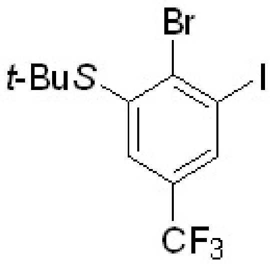

S4m

^{19}F NMR (376 MHz, $CDCl_3$)

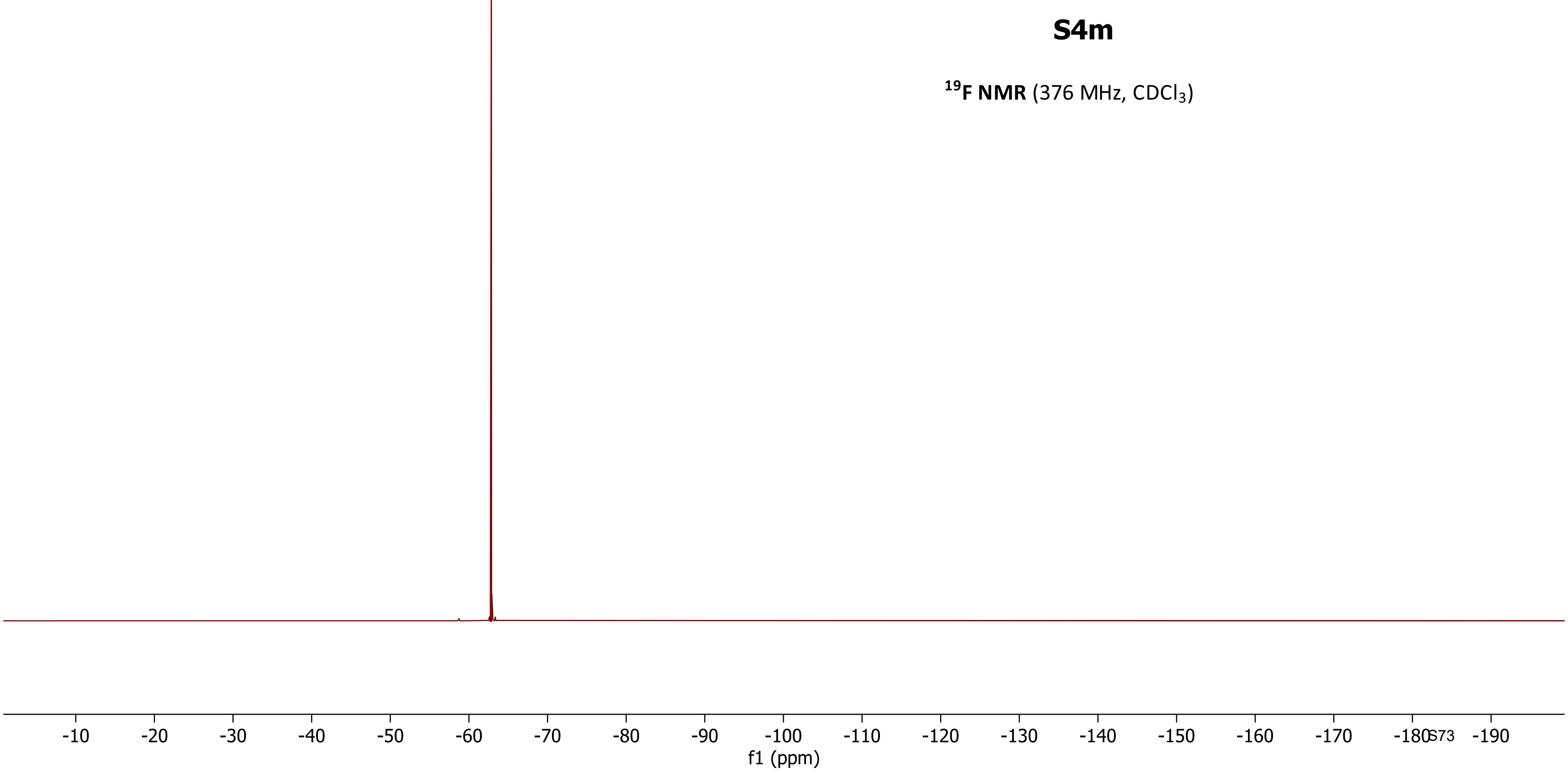

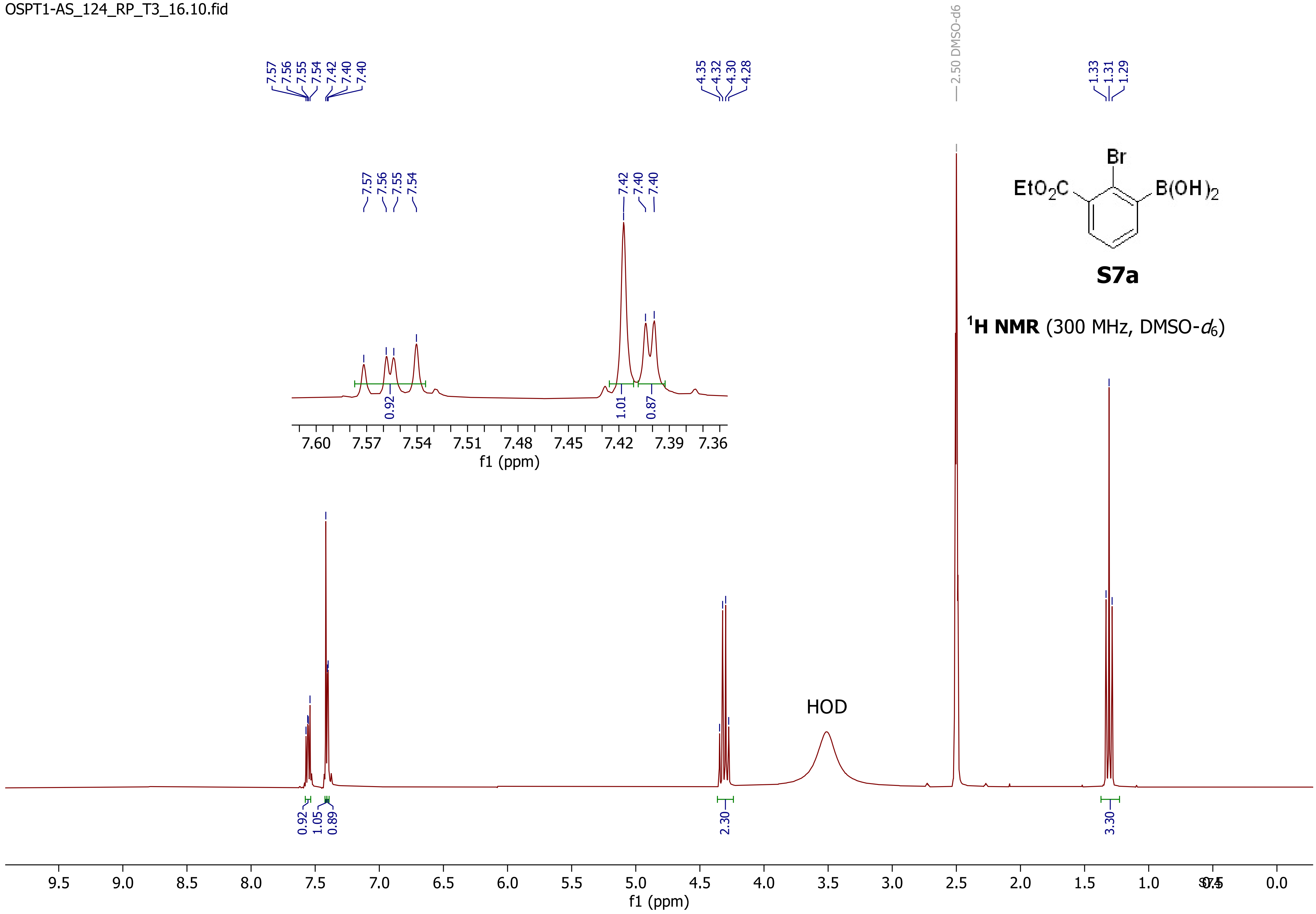
OSPT1-AS_124_RP_T3_16.10.fid
7.57
7.56
7.55
7.54
7.42
7.40
7.40
4.35
4.32
4.30
4.28
2.50 DMSO-d6
1.33
1.31
1.29
Br
EtO2C
B(OH)2
S7a
1H NMR (300 MHz, DMSO-d6)
0.92
1.01
0.87
7.60
7.57
7.54
7.51
7.48
7.45
7.42
7.39
7.36
f1 (ppm)
HOD
0.92
1.05
0.89
2.30
3.30
9.5
9.0
8.5
8.0
7.5
7.0
6.5
6.0
5.5
5.0
4.5
4.0
3.5
3.0
2.5
2.0
1.5
1.0
0.5
0.0
f1 (ppm)

as_70_extr_re.2.fid

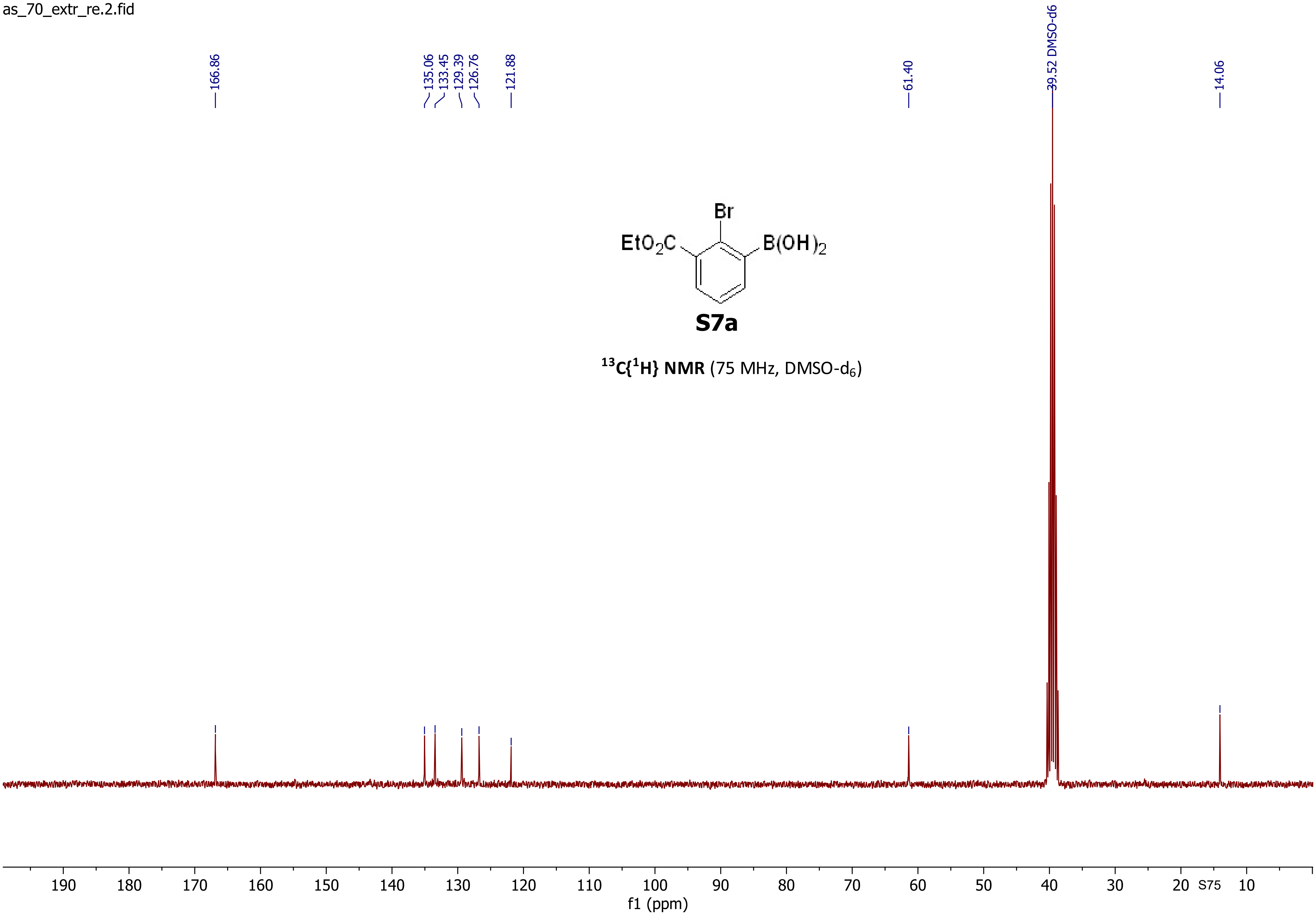

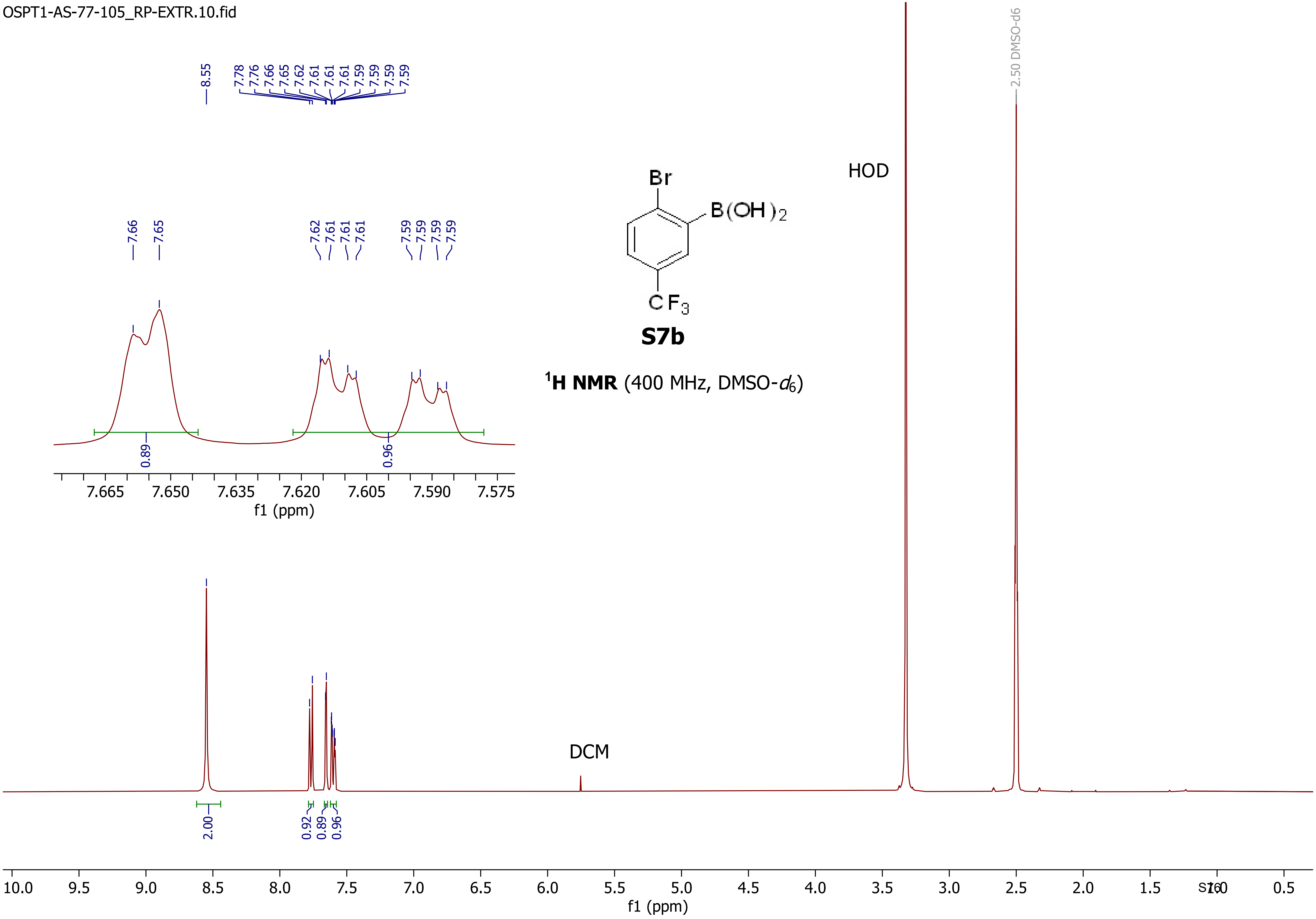

S7b

^{1}H NMR (400 MHz, DMSO-d_6)

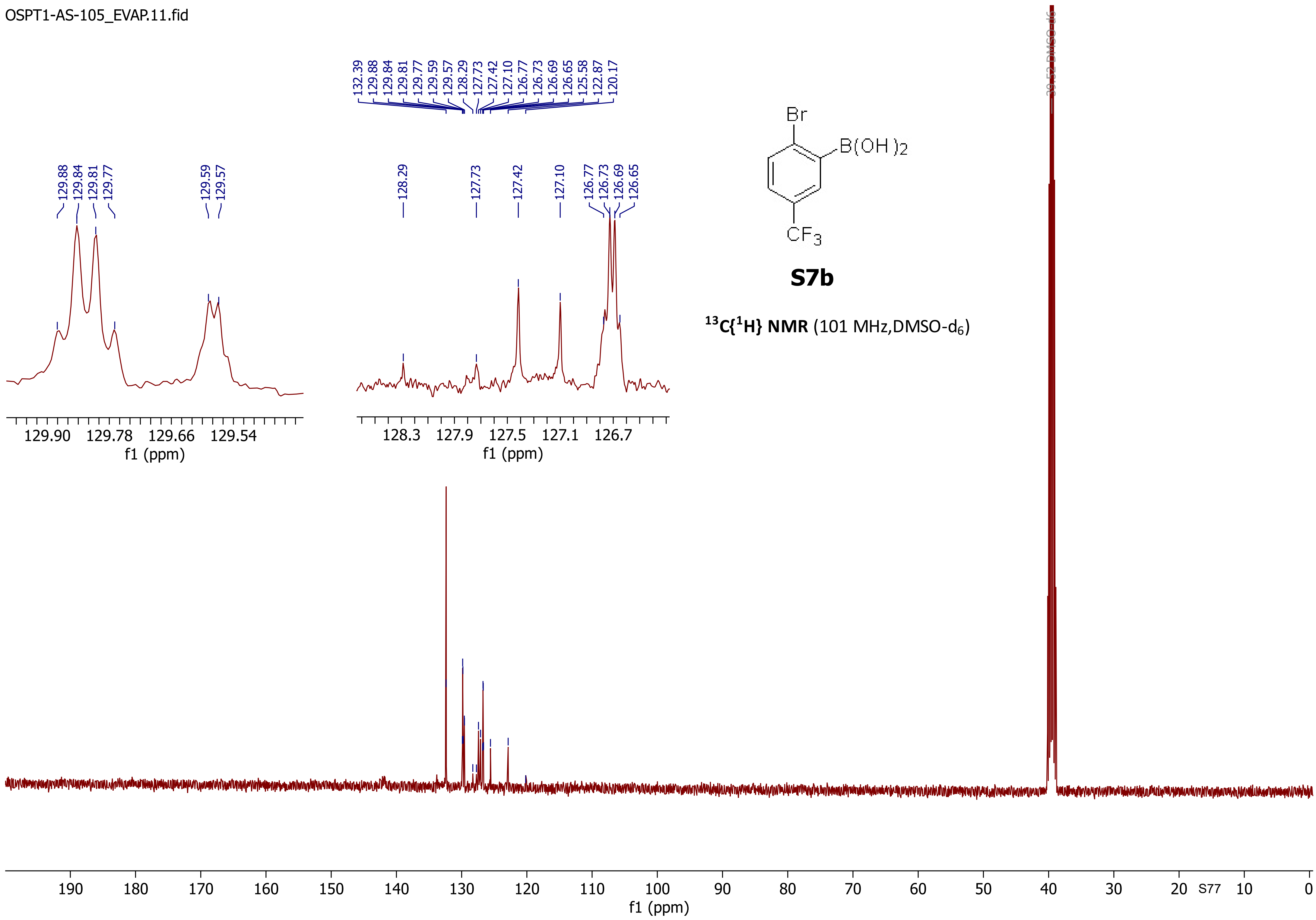

S7b

^{13}**C{^{1}H} NMR** (101 MHz,DMSO-d$_6$)

OSPT1-AS_77-105-RE.12.1.1r

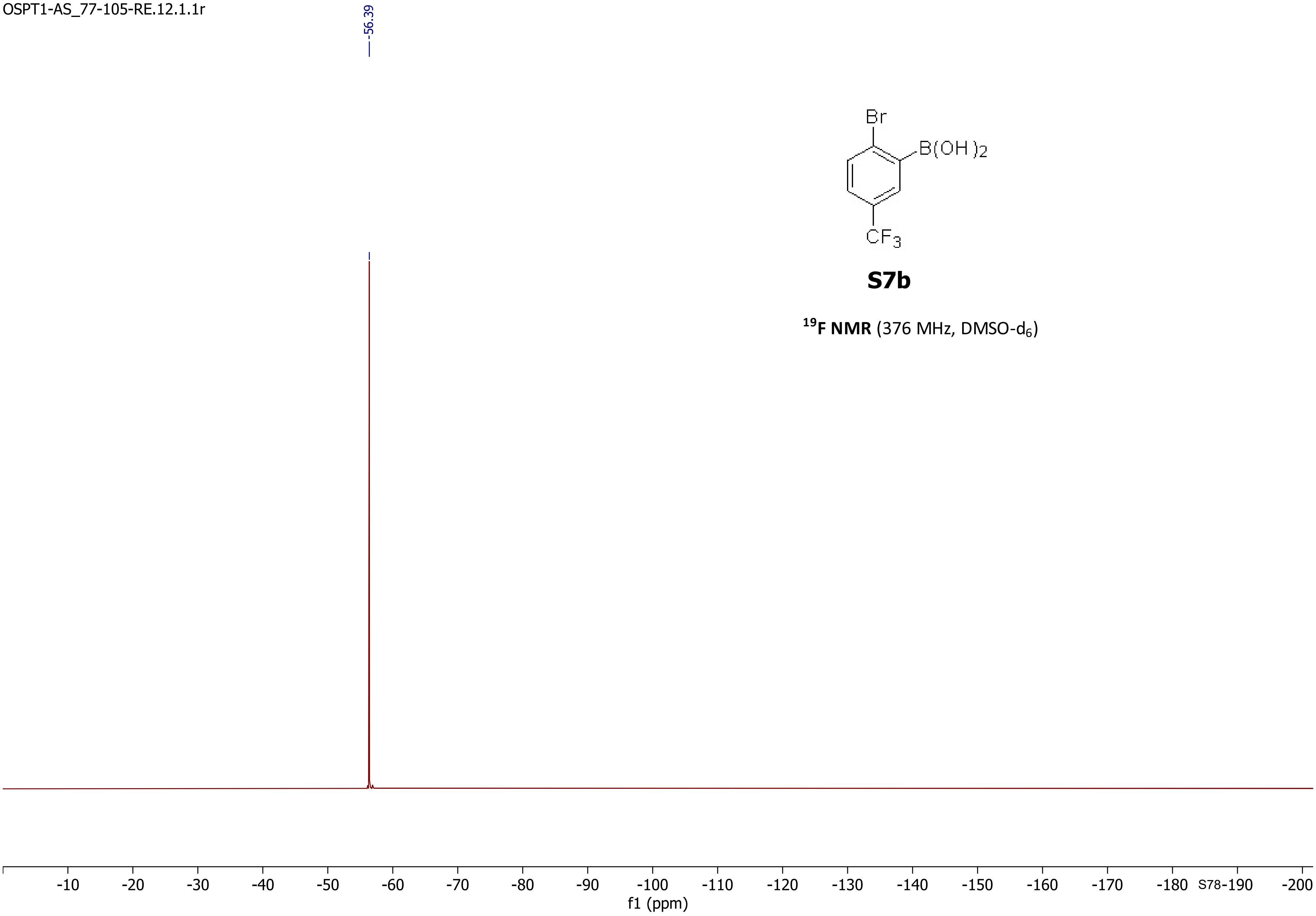

S7b

^{19}F NMR (376 MHz, DMSO-d_6)

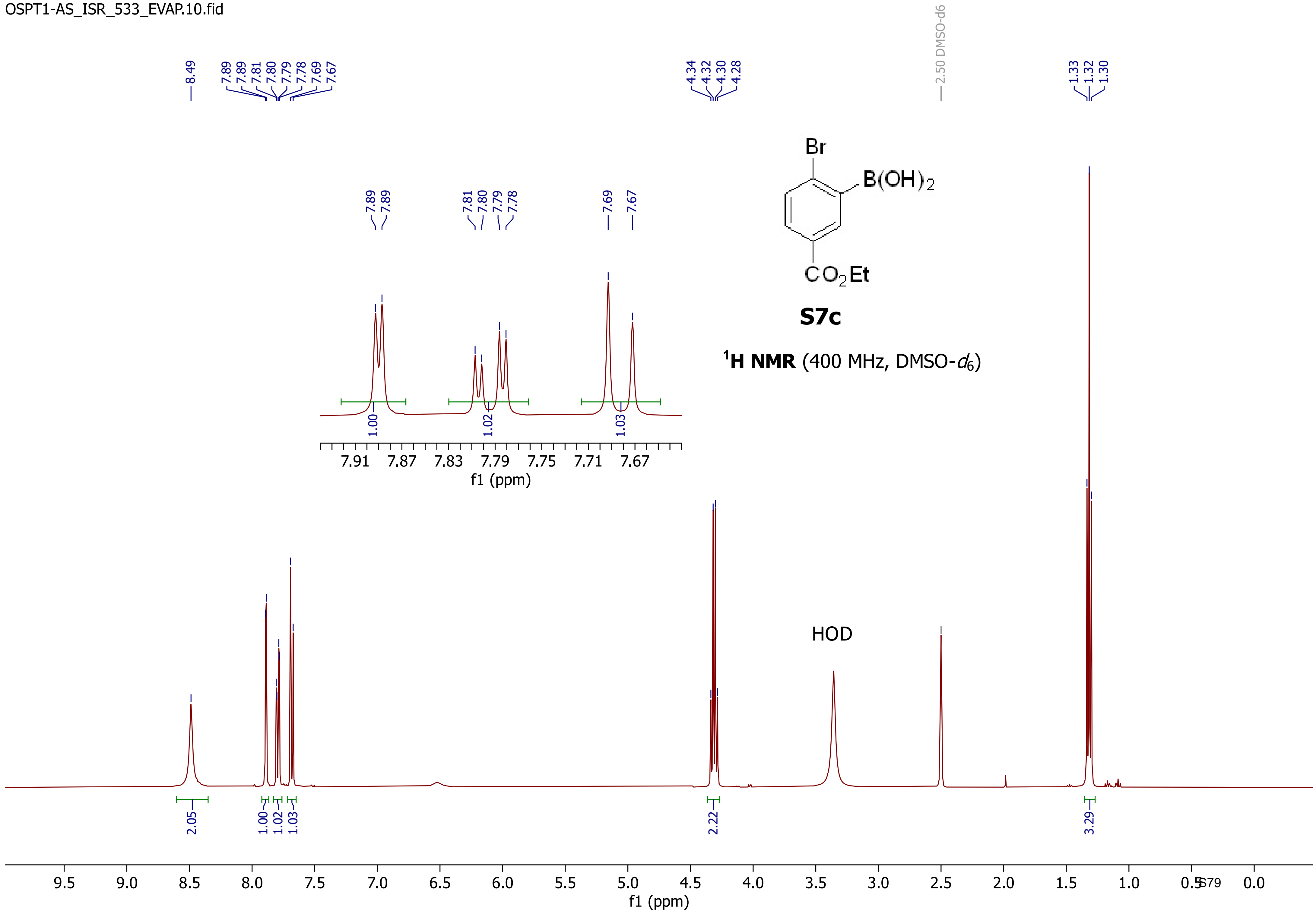
OSPT1-AS_ISR_533_EVAP.10.fid
8.49
7.89 7.89 7.81 7.80 7.79 7.78 7.69 7.67
4.34 4.32 4.30 4.28
2.50 DMSO-d6
1.33 1.32 1.30
Br
B(OH)2
CO2Et
S7c
1H NMR (400 MHz, DMSO-d6)
HOD
1.00
1.02
1.03
7.91 7.87 7.83 7.79 7.75 7.71 7.67
f1 (ppm)
2.05
2.22
3.29
9.5 9.0 8.5 8.0 7.5 7.0 6.5 6.0 5.5 5.0 4.5 4.0 3.5 3.0 2.5 2.0 1.5 1.0 0.5 0.0
f1 (ppm)

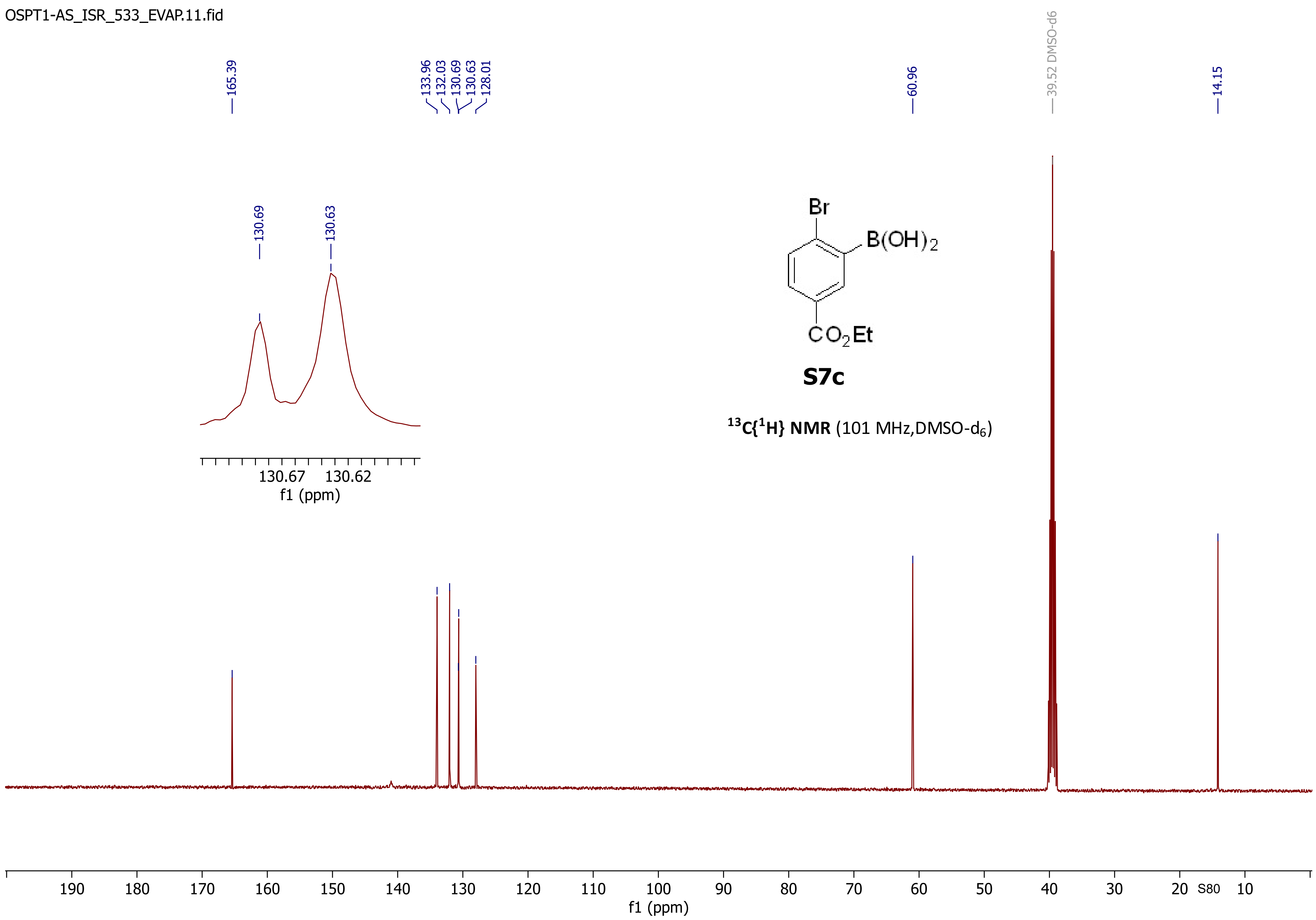
OSPT1-AS_ISR_533_EVAP.11.fid
165.39
133.96
132.03
130.69
130.63
128.01
60.96
39.52 DMSO-d6
14.15
130.69
130.63
130.67
130.62
f1 (ppm)
Br
B(OH)2
CO2Et
S7c
13C{1H} NMR (101 MHz,DMSO-d6)
190
180
170
160
150
140
130
120
110
100
90
80
70
60
50
40
30
20
10
f1 (ppm)

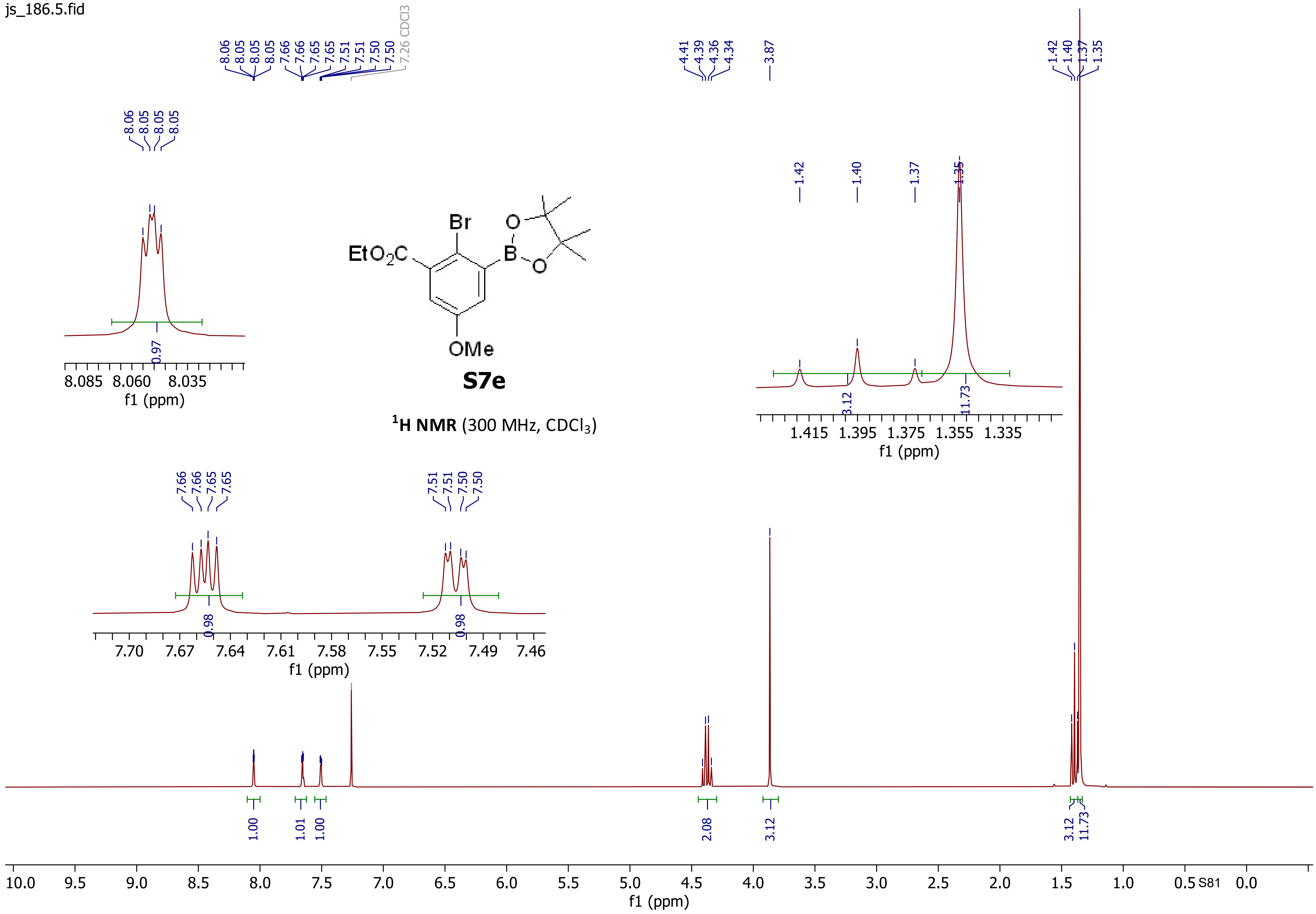
js_186.5.fid
8.06 8.05 8.05 8.05 7.66 7.66 7.65 7.65 7.51 7.51 7.50 7.50 7.26 CDCl3
4.41 4.39 4.36 4.34
3.87
1.42 1.40 1.37 1.35
Br
EtO2C
O
B
O
OMe
S7e
¹H NMR (300 MHz, CDCl3)
1.00 1.01 1.00 2.08 3.12 3.12 11.73
f1 (ppm)

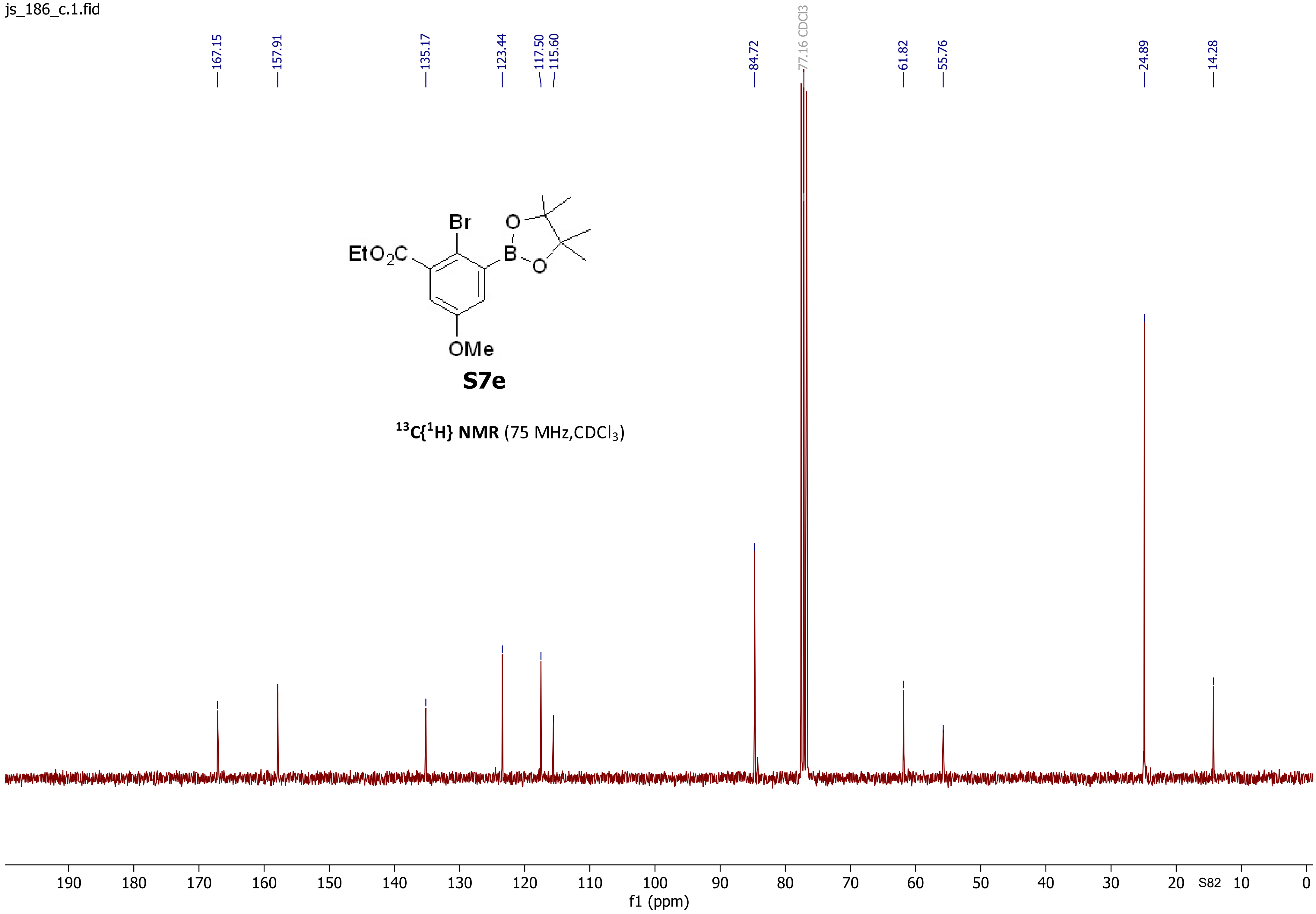
js_186_c.1.fid
167.15
157.91
135.17
123.44
117.50
115.60
84.72
77.16 CDCl3
61.82
55.76
24.89
14.28
Br
EtO2C
B
O
O
OMe
S7e
13C{1H} NMR (75 MHz,CDCl3)
190 180 170 160 150 140 130 120 110 100 90 80 70 60 50 40 30 20 10 0
f1 (ppm)

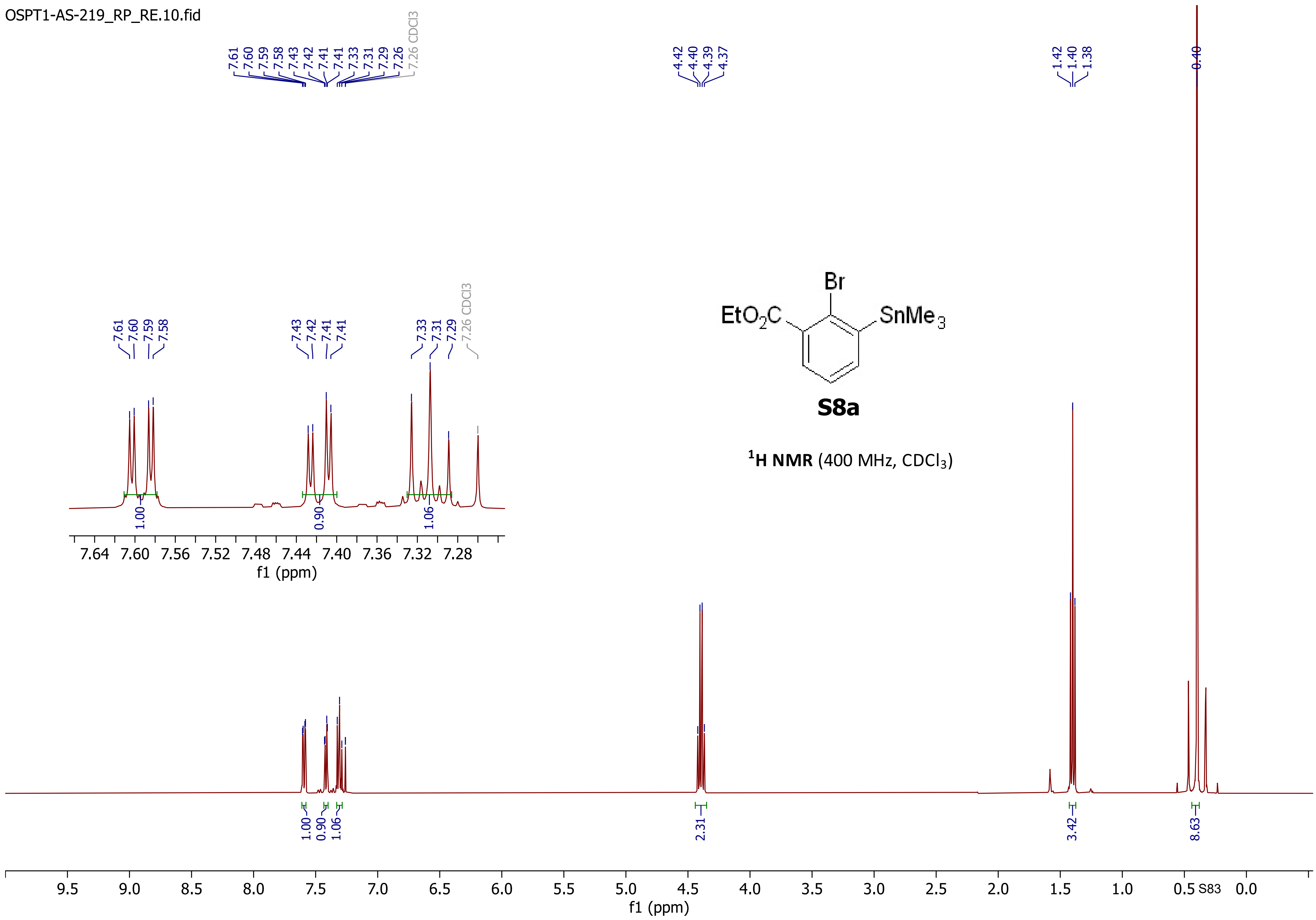
OSPT1-AS-219_RP_RE.10.fid
7.61
7.60
7.59
7.58
7.43
7.42
7.41
7.41
7.33
7.31
7.29
7.26
7.26 CDCl3
4.42
4.40
4.39
4.37
1.42
1.40
1.38
0.40
Br
EtO_2C
$SnMe_3$
S8a
^{1}H NMR (400 MHz, $CDCl_3$)
1.00
0.90
1.06
2.31
3.42
8.63
7.64 7.60 7.56 7.52 7.48 7.44 7.40 7.36 7.32 7.28
f1 (ppm)
9.5 9.0 8.5 8.0 7.5 7.0 6.5 6.0 5.5 5.0 4.5 4.0 3.5 3.0 2.5 2.0 1.5 1.0 0.5 0.0
f1 (ppm)

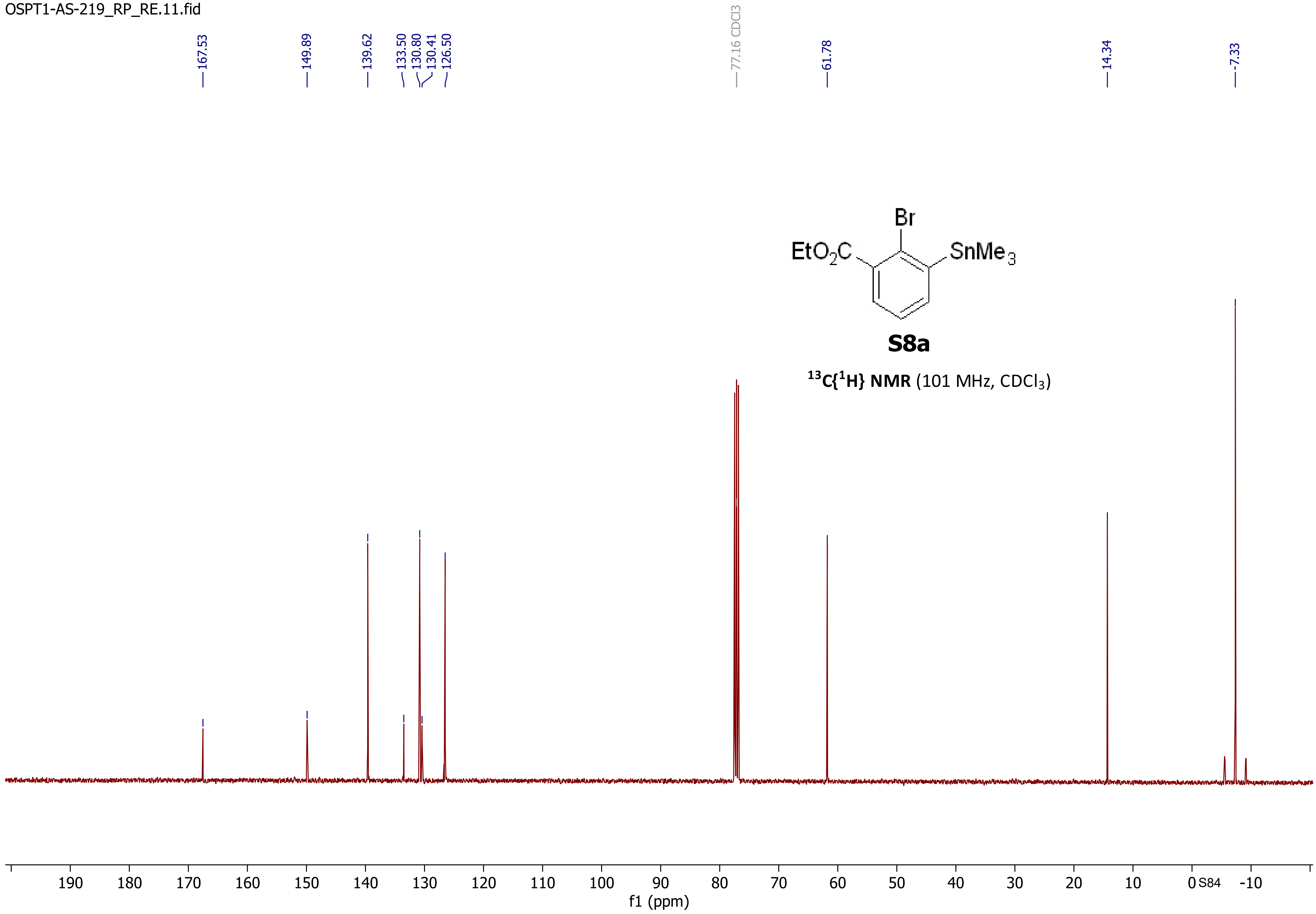
OSPT1-AS-219_RP_RE.11.fid
167.53
149.89
139.62
133.50
130.80
130.41
126.50
77.16 CDCl3
61.78
14.34
-7.33
Br
EtO2C
SnMe3
S8a
13C{1H} NMR (101 MHz, CDCl3)
190 180 170 160 150 140 130 120 110 100 90 80 70 60 50 40 30 20 10 0 -10
f1 (ppm)

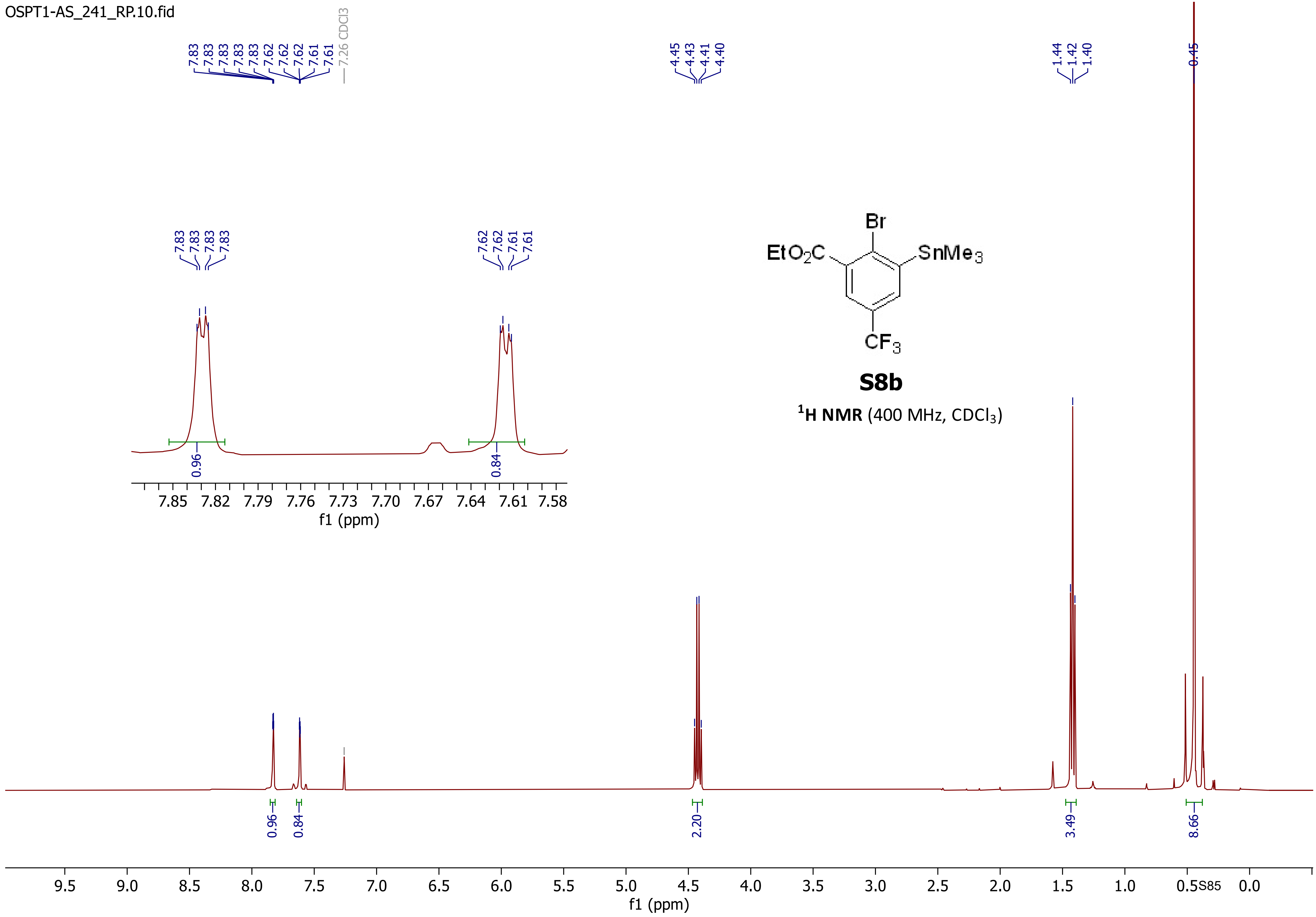
OSPT1-AS_241_RP.10.fid
7.83
7.83
7.83
7.83
7.83
7.62
7.62
7.62
7.61
7.61
7.26 CDCl3
4.45
4.43
4.41
4.40
1.44
1.42
1.40
0.45
Br
EtO2C
SnMe3
CF3
S8b
1H NMR (400 MHz, CDCl3)
0.96
0.84
2.20
3.49
8.66
7.85 7.82 7.79 7.76 7.73 7.70 7.67 7.64 7.61 7.58
f1 (ppm)
9.5 9.0 8.5 8.0 7.5 7.0 6.5 6.0 5.5 5.0 4.5 4.0 3.5 3.0 2.5 2.0 1.5 1.0 0.5 0.0
f1 (ppm)

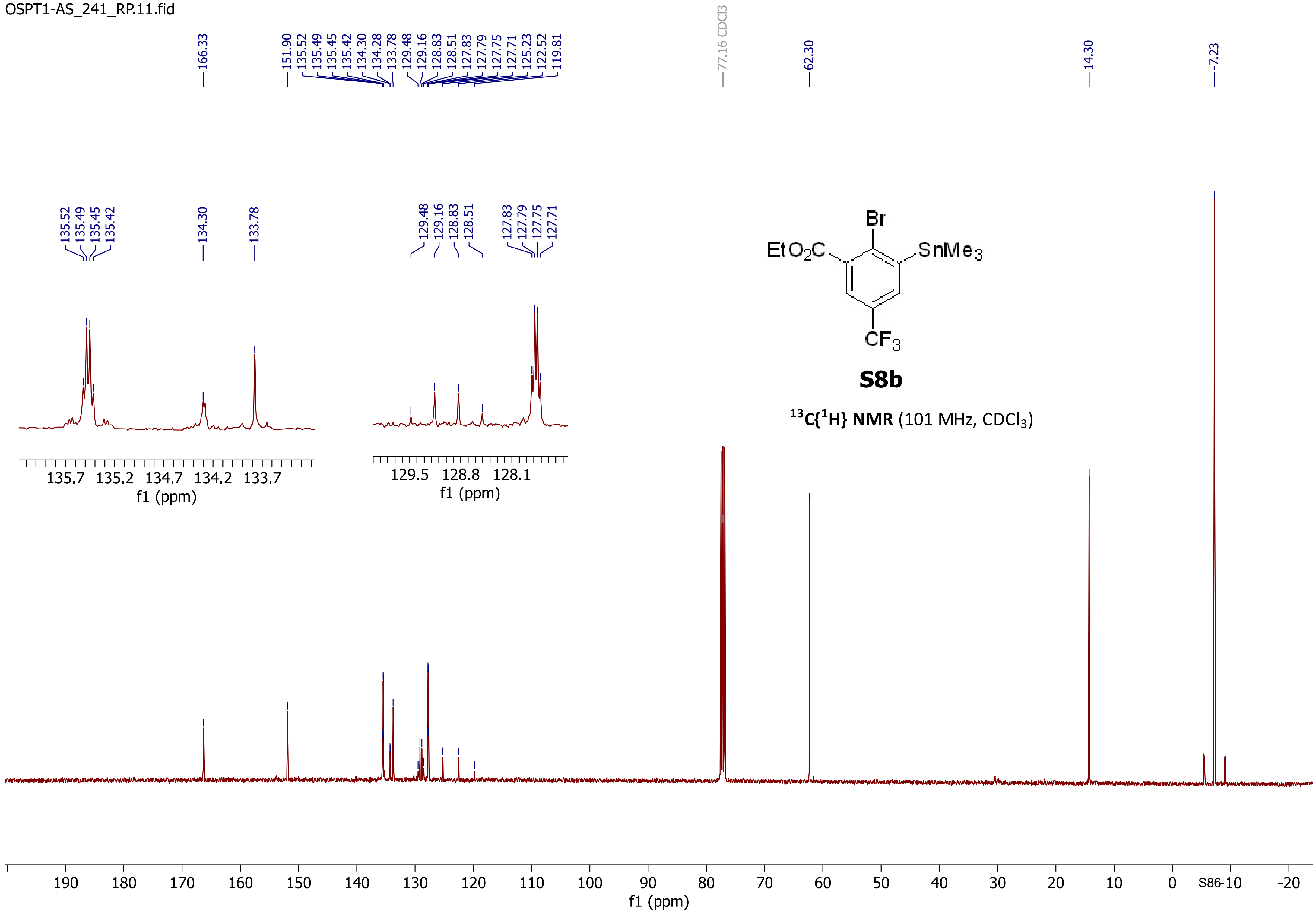
OSPT1-AS_241_RP.11.fid
166.33
151.90
135.52
135.49
135.45
135.42
134.30
134.28
133.78
129.48
129.16
128.83
128.51
127.83
127.79
127.75
127.71
125.23
122.52
119.81
77.16 CDCl3
62.30
14.30
-7.23
135.7 135.2 134.7 134.2 133.7
f1 (ppm)
129.5 128.8 128.1
f1 (ppm)
Br
EtO2C
SnMe3
CF3
S8b
13C{1H} NMR (101 MHz, CDCl3)
190 180 170 160 150 140 130 120 110 100 90 80 70 60 50 40 30 20 10 0 -10 -20
f1 (ppm)

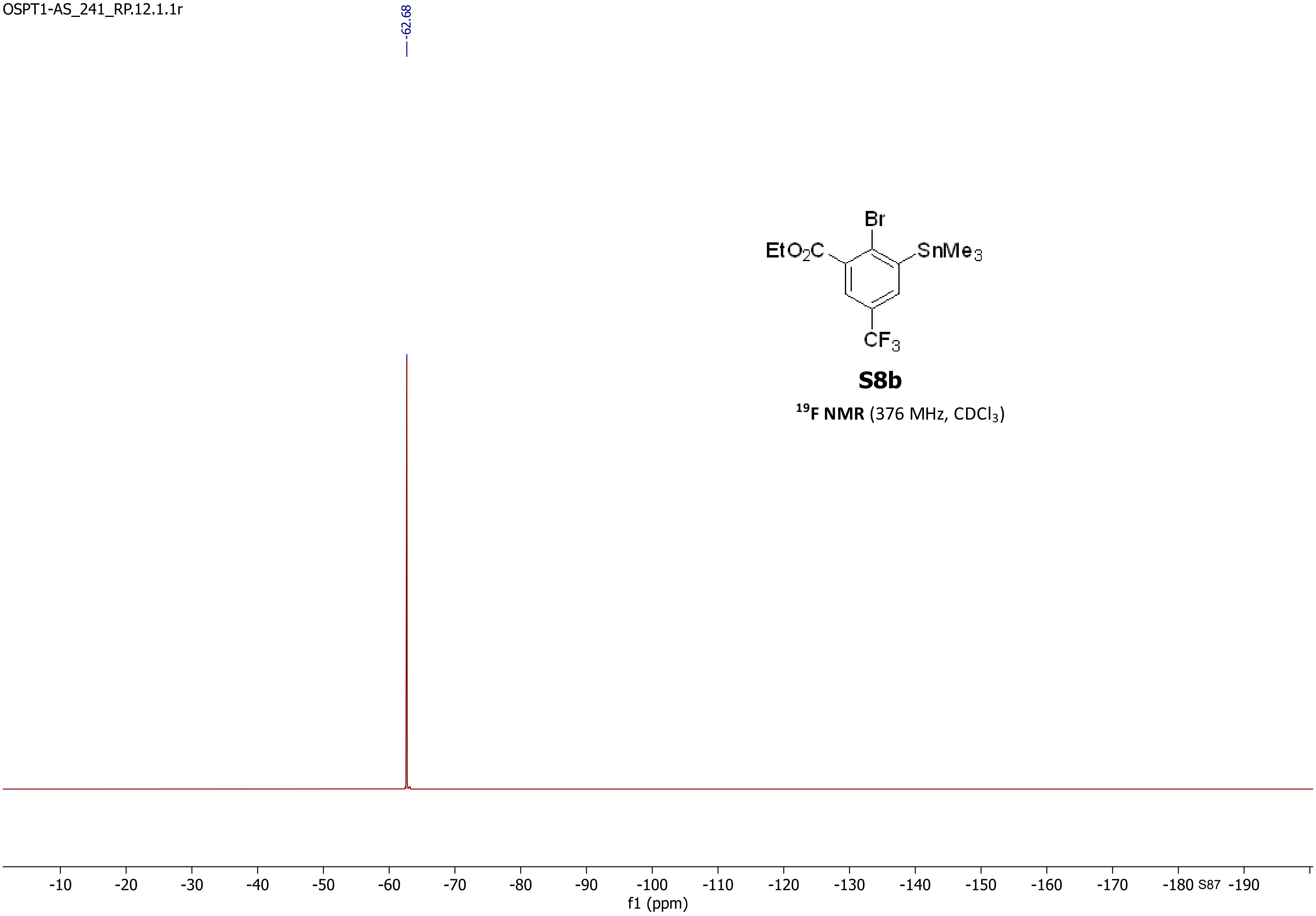
OSPT1-AS_241_RP.12.1.1r
-62.68
Br
EtO_2C
$SnMe_3$
CF_3
S8b
^{19}F NMR (376 MHz, $CDCl_3$)
-10 -20 -30 -40 -50 -60 -70 -80 -90 -100 -110 -120 -130 -140 -150 -160 -170 -180 -190
f1 (ppm)

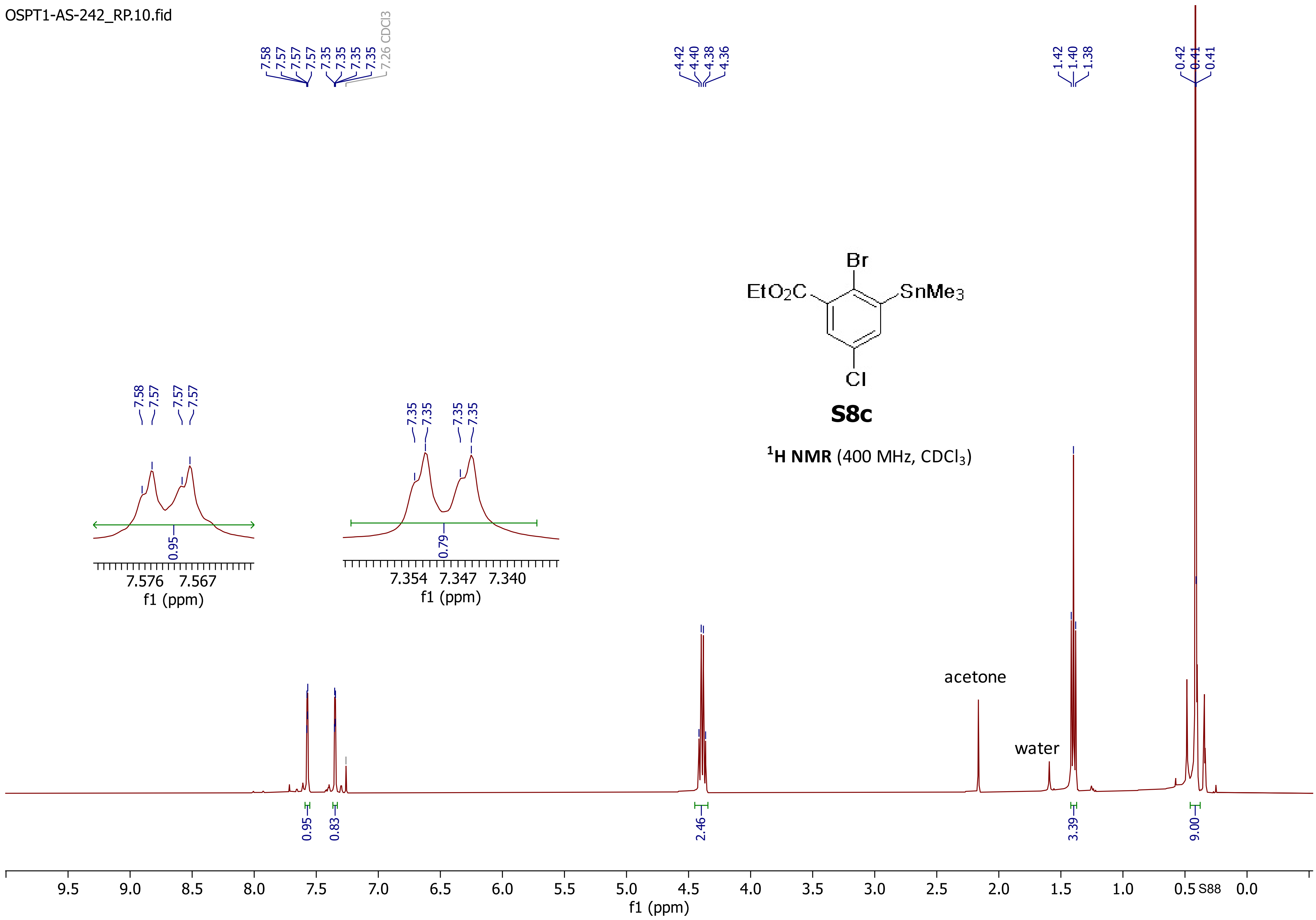
OSPT1-AS-242_RP.10.fid
7.58 7.57 7.57 7.57 7.35 7.35 7.35 7.35 7.26 CDCl3
4.42 4.40 4.38 4.36
1.42 1.40 1.38
0.42 0.41 0.41
Br
EtO2C
SnMe3
Cl
S8c
1H NMR (400 MHz, CDCl3)
7.58 7.57 7.57 7.57
0.95
7.576 7.567
f1 (ppm)
7.35 7.35 7.35 7.35
0.79
7.354 7.347 7.340
f1 (ppm)
acetone
water
0.95 0.83 2.46 3.39 9.00
9.5 9.0 8.5 8.0 7.5 7.0 6.5 6.0 5.5 5.0 4.5 4.0 3.5 3.0 2.5 2.0 1.5 1.0 0.5 0.0
f1 (ppm)

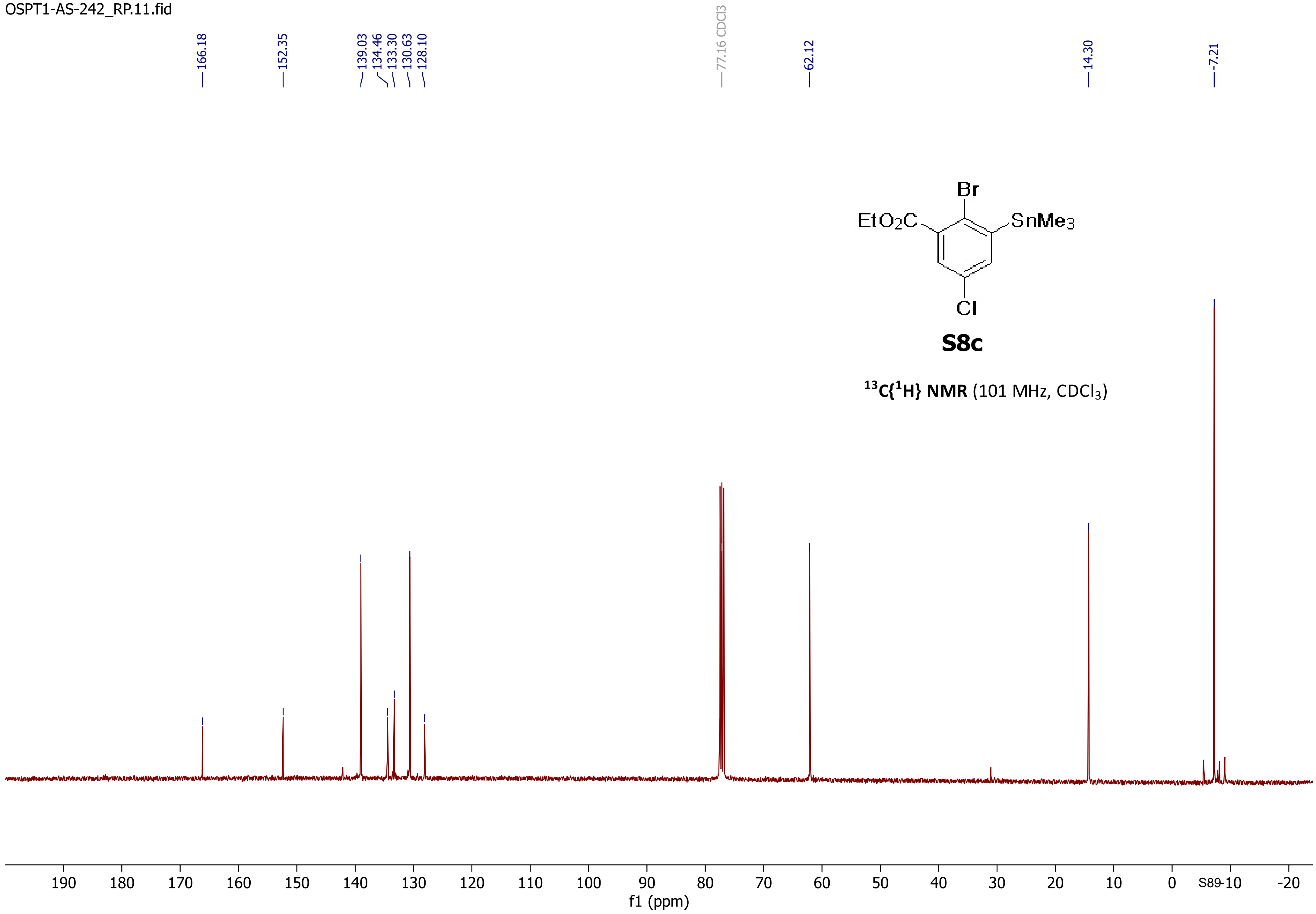
OSPT1-AS-242_RP.11.fid
166.18
152.35
139.03
134.46
133.30
130.63
128.10
77.16 CDCl3
62.12
14.30
-7.21
Br
EtO2C
SnMe3
Cl
S8c
13C{1H} NMR (101 MHz, CDCl3)
190 180 170 160 150 140 130 120 110 100 90 80 70 60 50 40 30 20 10 0 -10 -20
f1 (ppm)

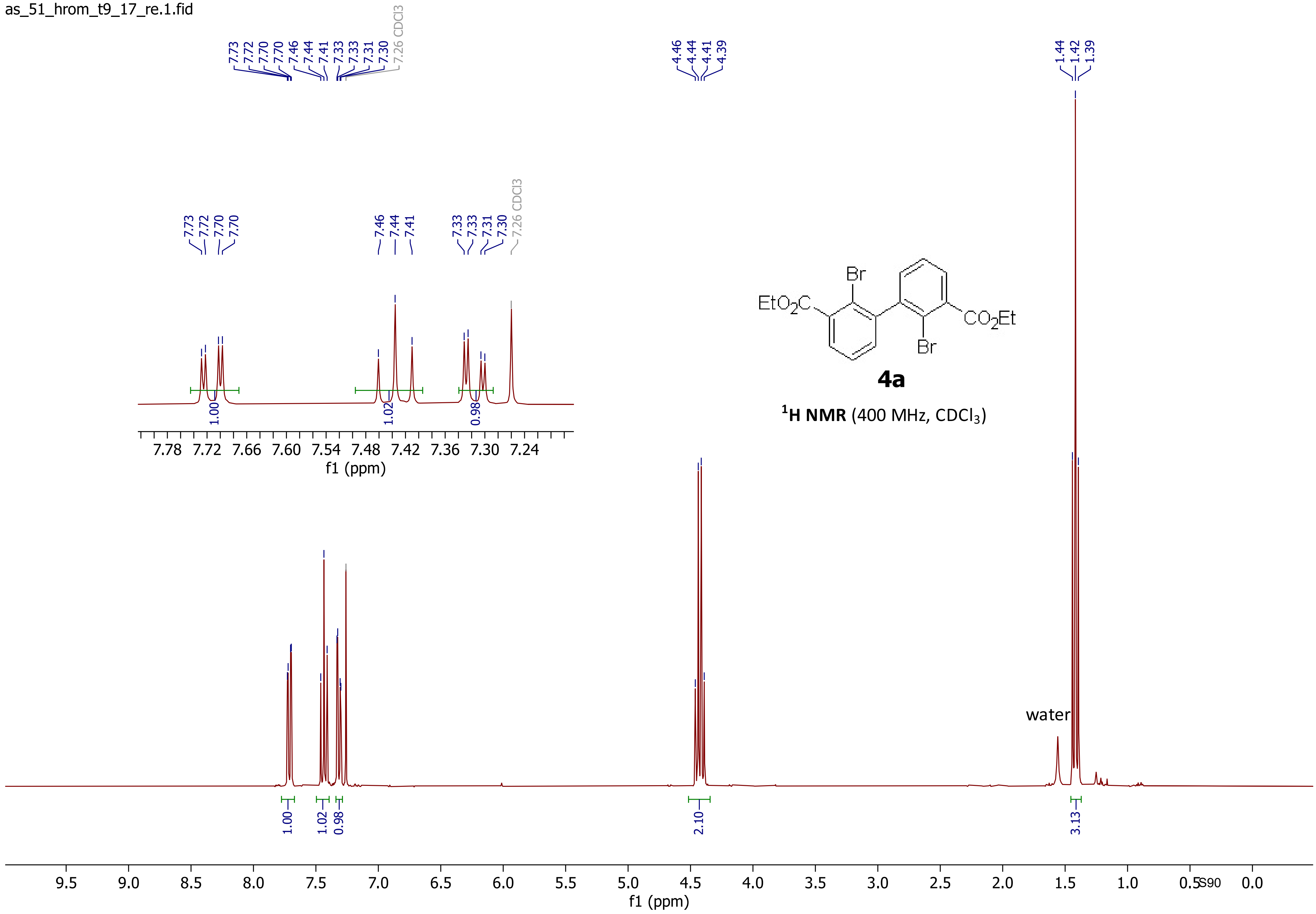
as_51_hrom_t9_17_re.1.fid
7.73 7.72 7.70 7.70 7.46 7.44 7.41 7.33 7.33 7.31 7.30 7.26 CDCl3
4.46 4.44 4.41 4.39
1.44 1.42 1.39
Br
EtO2C
CO2Et
Br
4a
1H NMR (400 MHz, CDCl3)
1.00
1.02
0.98
7.78 7.72 7.66 7.60 7.54 7.48 7.42 7.36 7.30 7.24
f1 (ppm)
water
1.00 1.02 0.98 2.10 3.13
9.5 9.0 8.5 8.0 7.5 7.0 6.5 6.0 5.5 5.0 4.5 4.0 3.5 3.0 2.5 2.0 1.5 1.0 0.5 0.0
f1 (ppm)

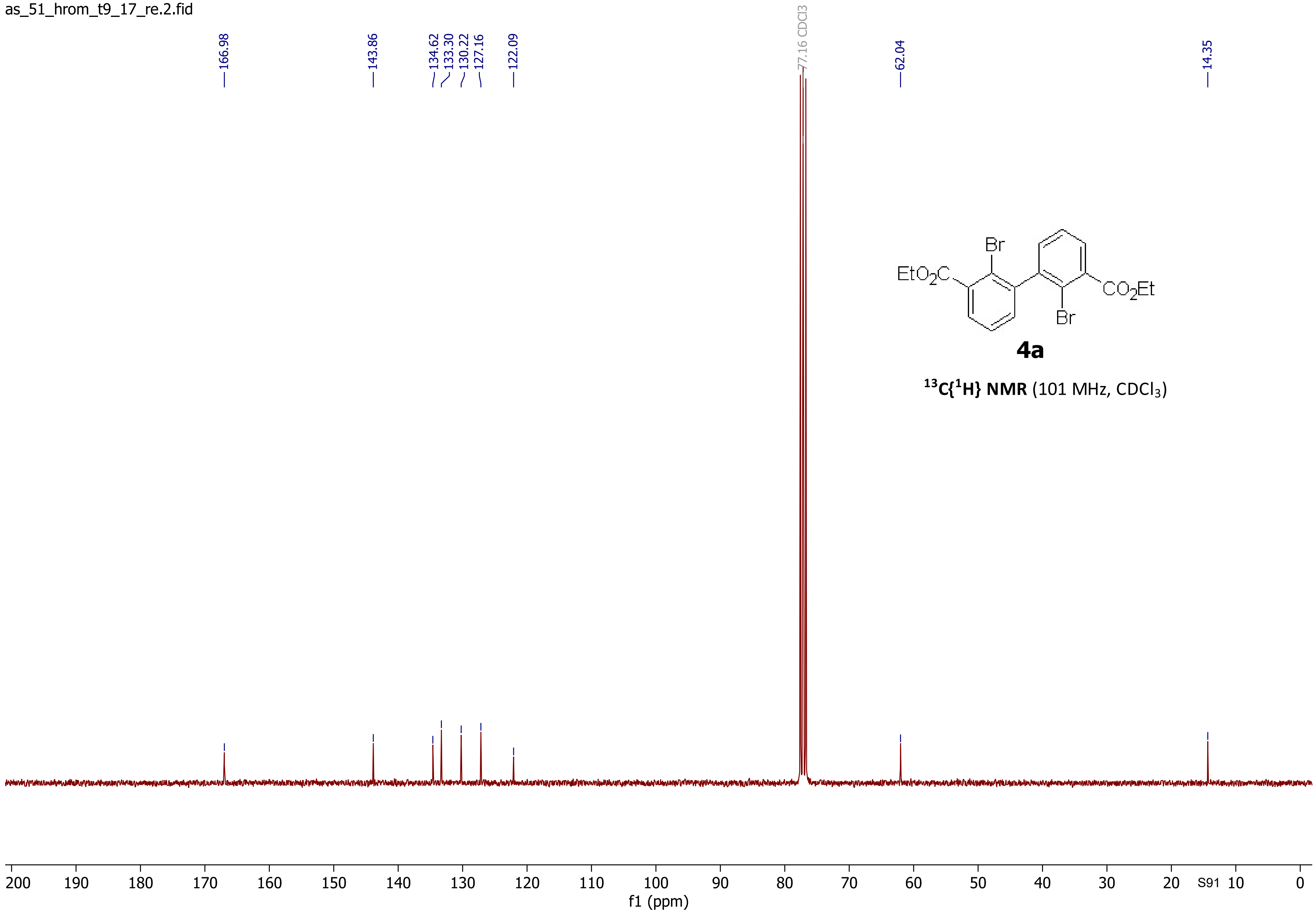
as_51_hrom_t9_17_re.2.fid
166.98
143.86
134.62
133.30
130.22
127.16
122.09
77.16 CDCl3
62.04
14.35
Br
EtO2C
CO2Et
Br
4a
13C{1H} NMR (101 MHz, CDCl3)
200 190 180 170 160 150 140 130 120 110 100 90 80 70 60 50 40 30 20 10 0
f1 (ppm)

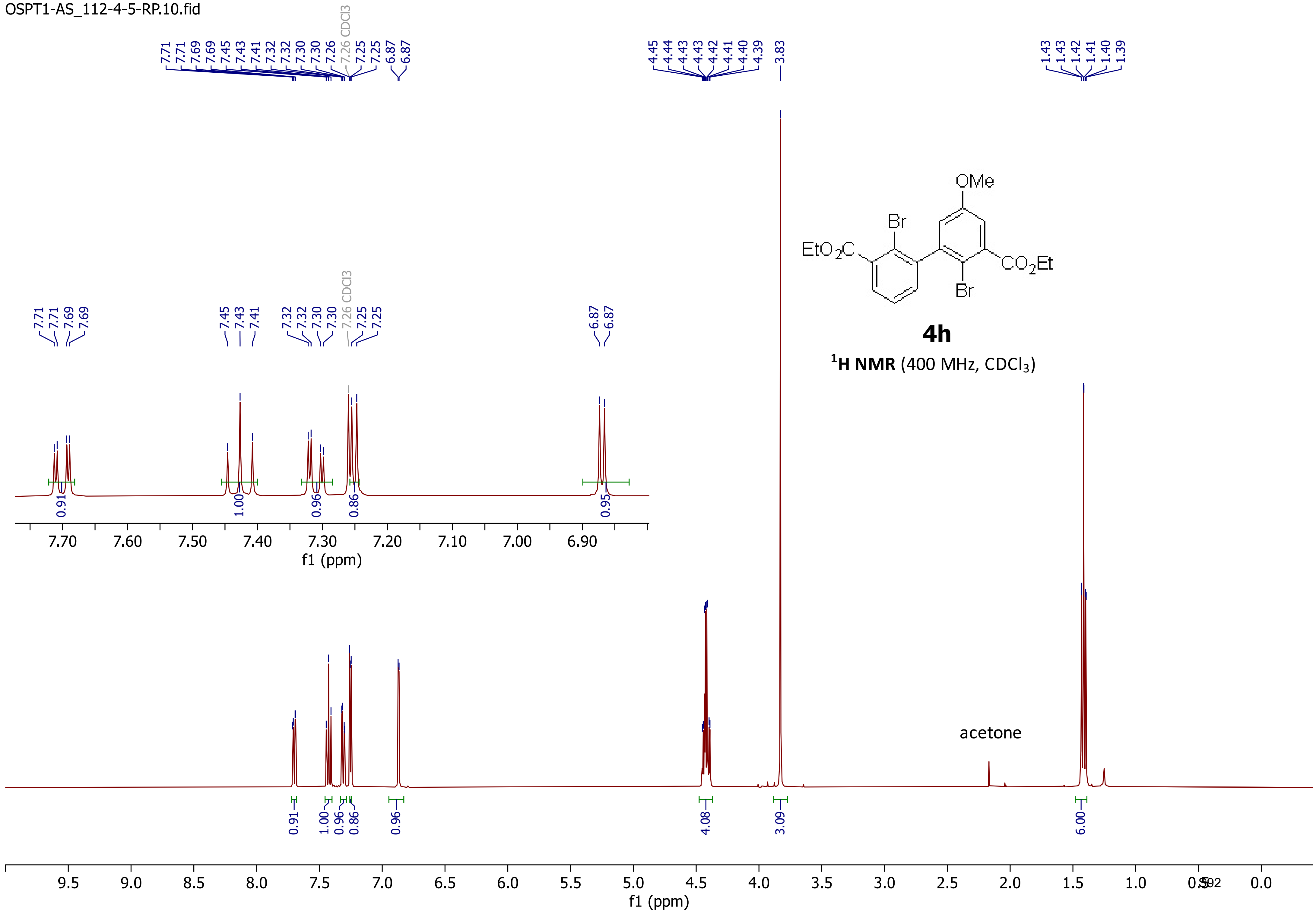
OSPT1-AS_112-4-5-RP.10.fid
7.71 7.71 7.69 7.69 7.45 7.43 7.41 7.32 7.32 7.30 7.30 7.26 7.26 CDCl3 7.25 7.25 6.87 6.87
4.45 4.44 4.43 4.43 4.42 4.41 4.40 4.39 3.83
1.43 1.43 1.42 1.41 1.40 1.39
OMe
Br
EtO2C
CO2Et
Br
4h
1H NMR (400 MHz, CDCl3)
0.91 1.00 0.96 0.86 0.95
7.70 7.60 7.50 7.40 7.30 7.20 7.10 7.00 6.90
f1 (ppm)
acetone
0.91 1.00 0.96 0.86 0.96 4.08 3.09 6.00
9.5 9.0 8.5 8.0 7.5 7.0 6.5 6.0 5.5 5.0 4.5 4.0 3.5 3.0 2.5 2.0 1.5 1.0 0.5 0.0
f1 (ppm)

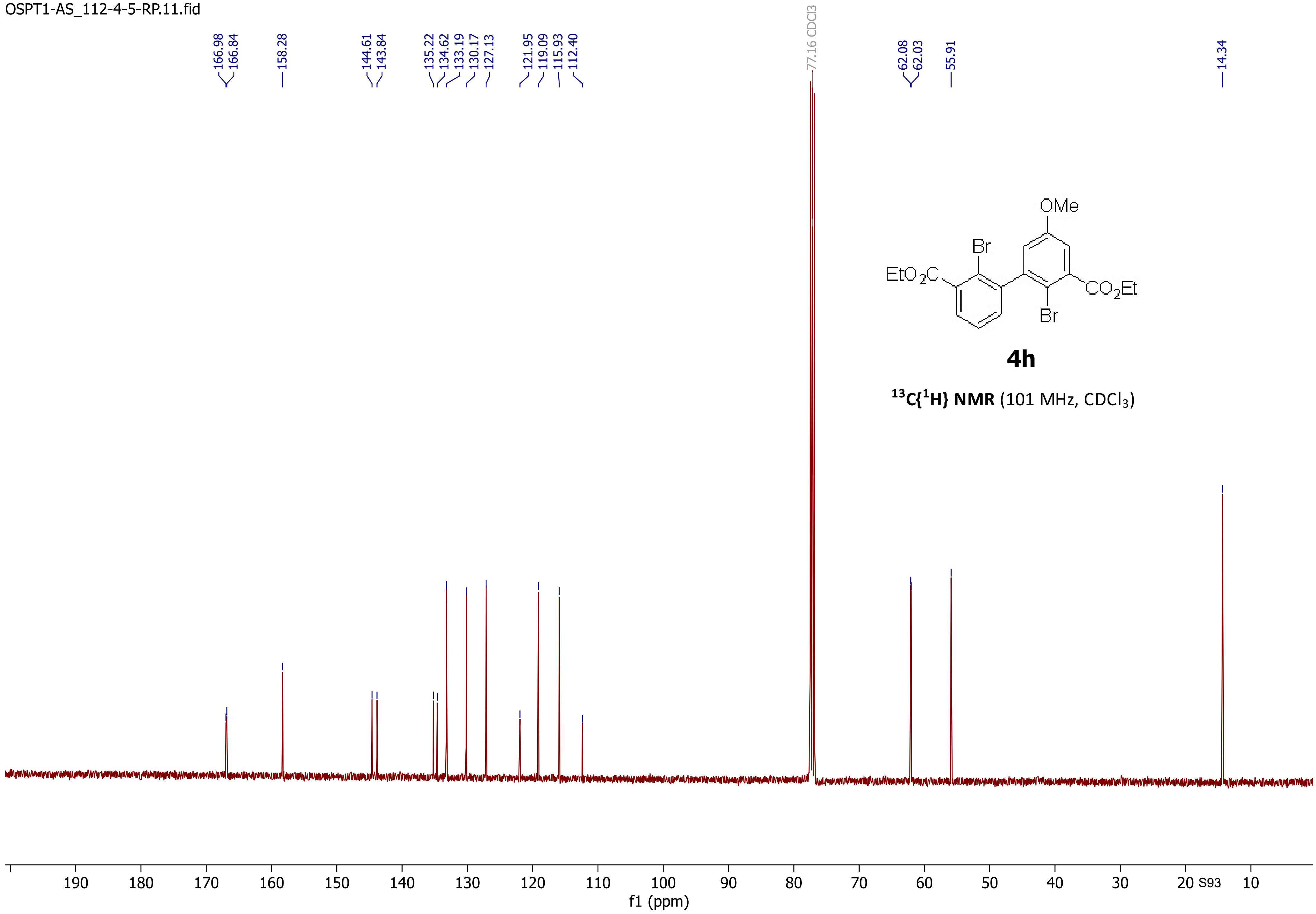

4h

$^{13}C\{^{1}H\}$ **NMR** (101 MHz, $CDCl_3$)

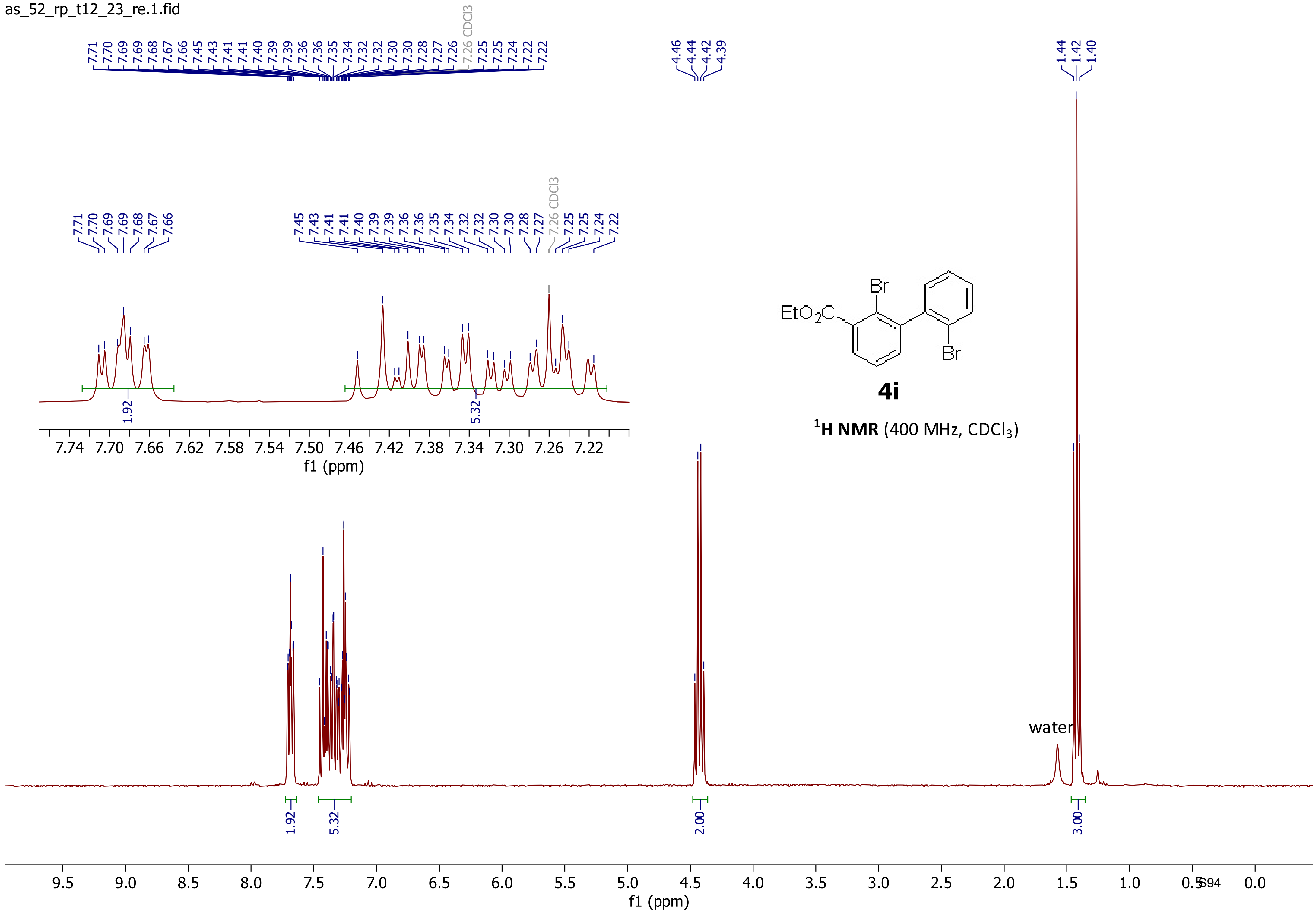
as_52_rp_t12_23_re.1.fid
7.71 7.70 7.69 7.69 7.68 7.67 7.66 7.45 7.43 7.41 7.41 7.40 7.39 7.39 7.36 7.36 7.35 7.34 7.32 7.32 7.30 7.30 7.28 7.27 7.26 7.26 CDCl3 7.25 7.25 7.24 7.22 7.22
4.46 4.44 4.42 4.39
1.44 1.42 1.40
1.92
5.32
Br
EtO2C
Br
4i
1H NMR (400 MHz, CDCl3)
7.74 7.70 7.66 7.62 7.58 7.54 7.50 7.46 7.42 7.38 7.34 7.30 7.26 7.22
f1 (ppm)
water
1.92
5.32
2.00
3.00
9.5 9.0 8.5 8.0 7.5 7.0 6.5 6.0 5.5 5.0 4.5 4.0 3.5 3.0 2.5 2.0 1.5 1.0 0.5 0.0
f1 (ppm)

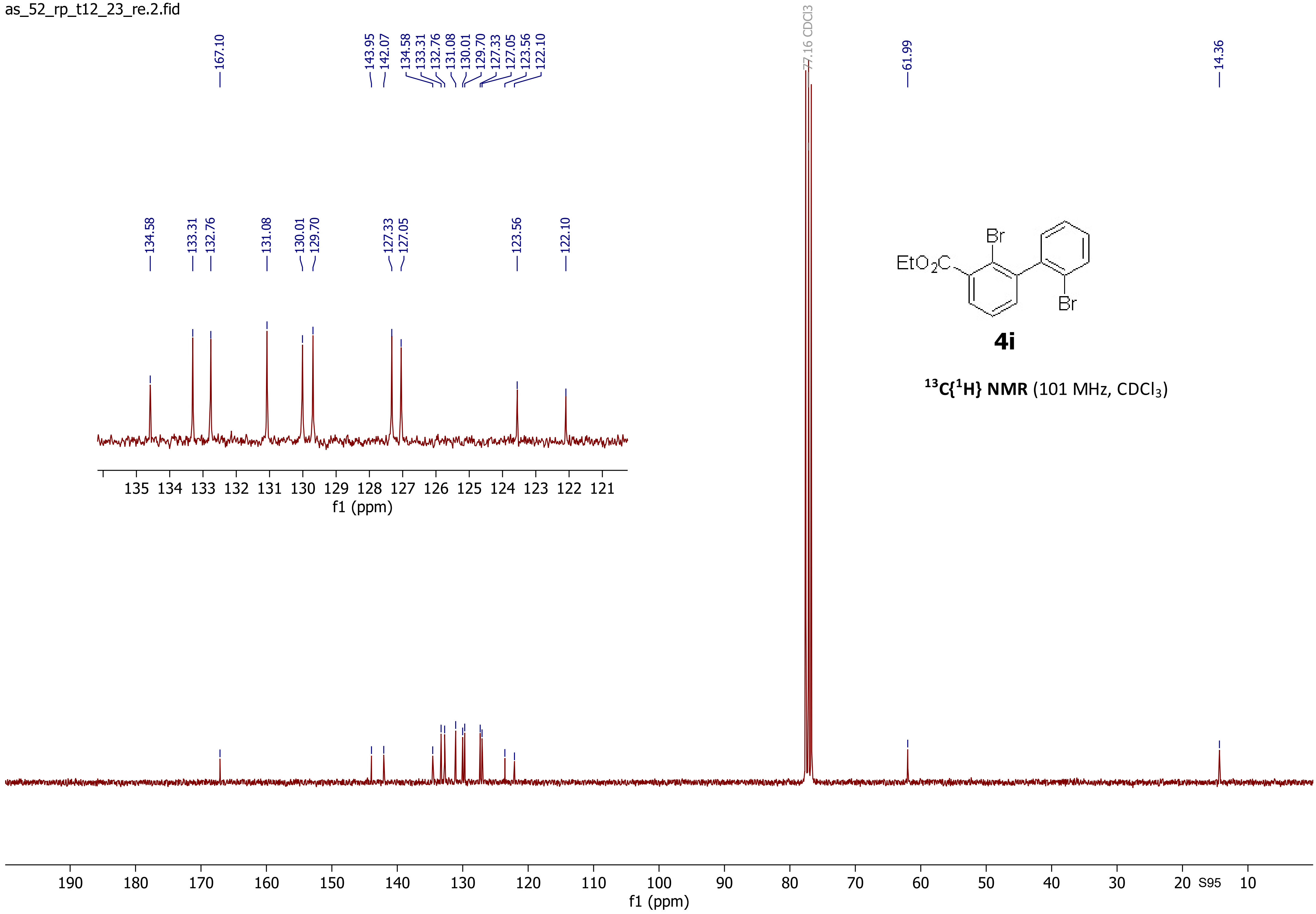
as_52_rp_t12_23_re.2.fid
167.10
143.95
142.07
134.58
133.31
132.76
131.08
130.01
129.70
127.33
127.05
123.56
122.10
77.16 CDCl3
61.99
14.36
f1 (ppm)
EtO2C
Br
Br
4i
13C{1H} NMR (101 MHz, CDCl3)

OSPT1-AS_211_RP.10.fid

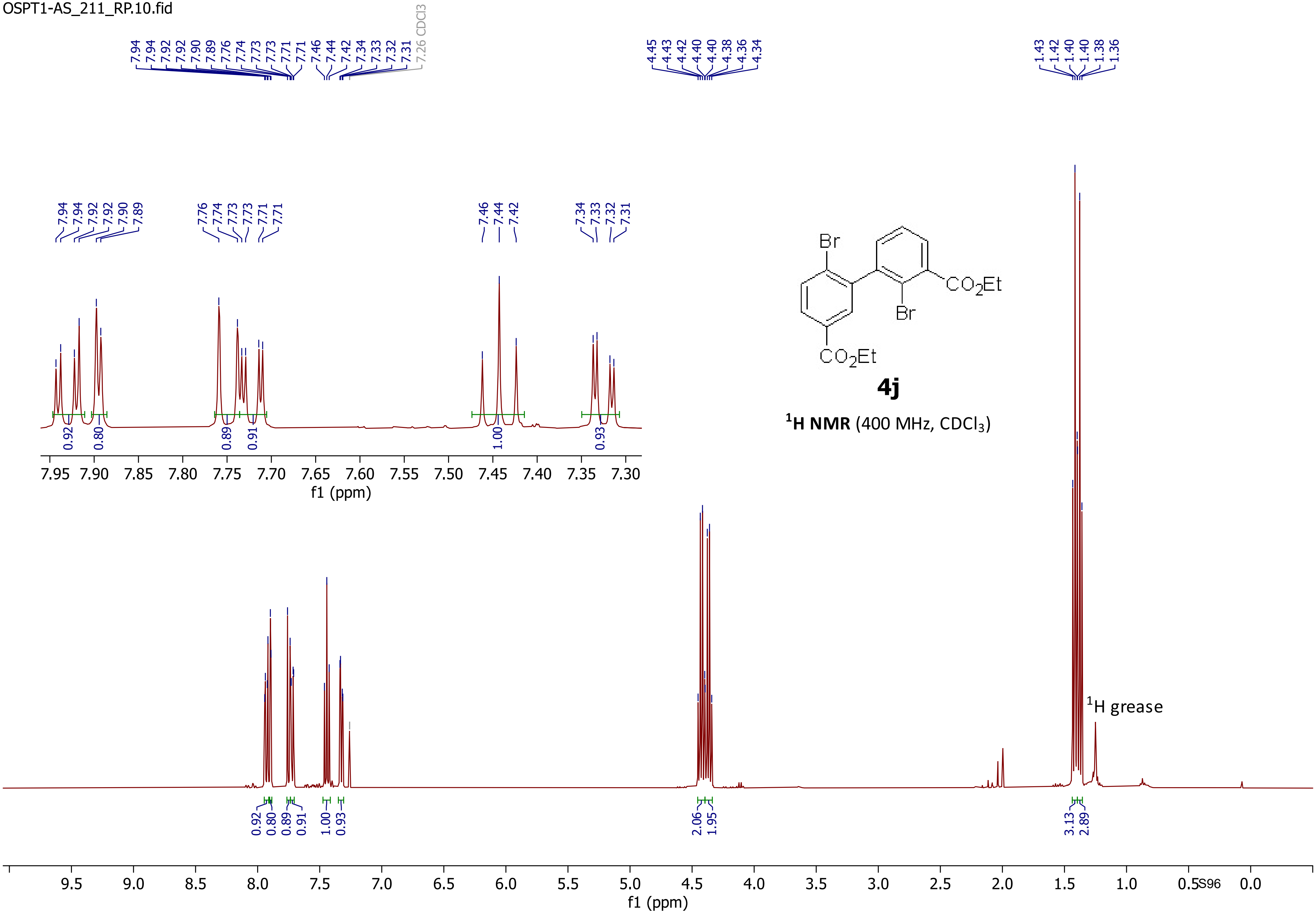

OSPT1-AS_211_RP.11.fid

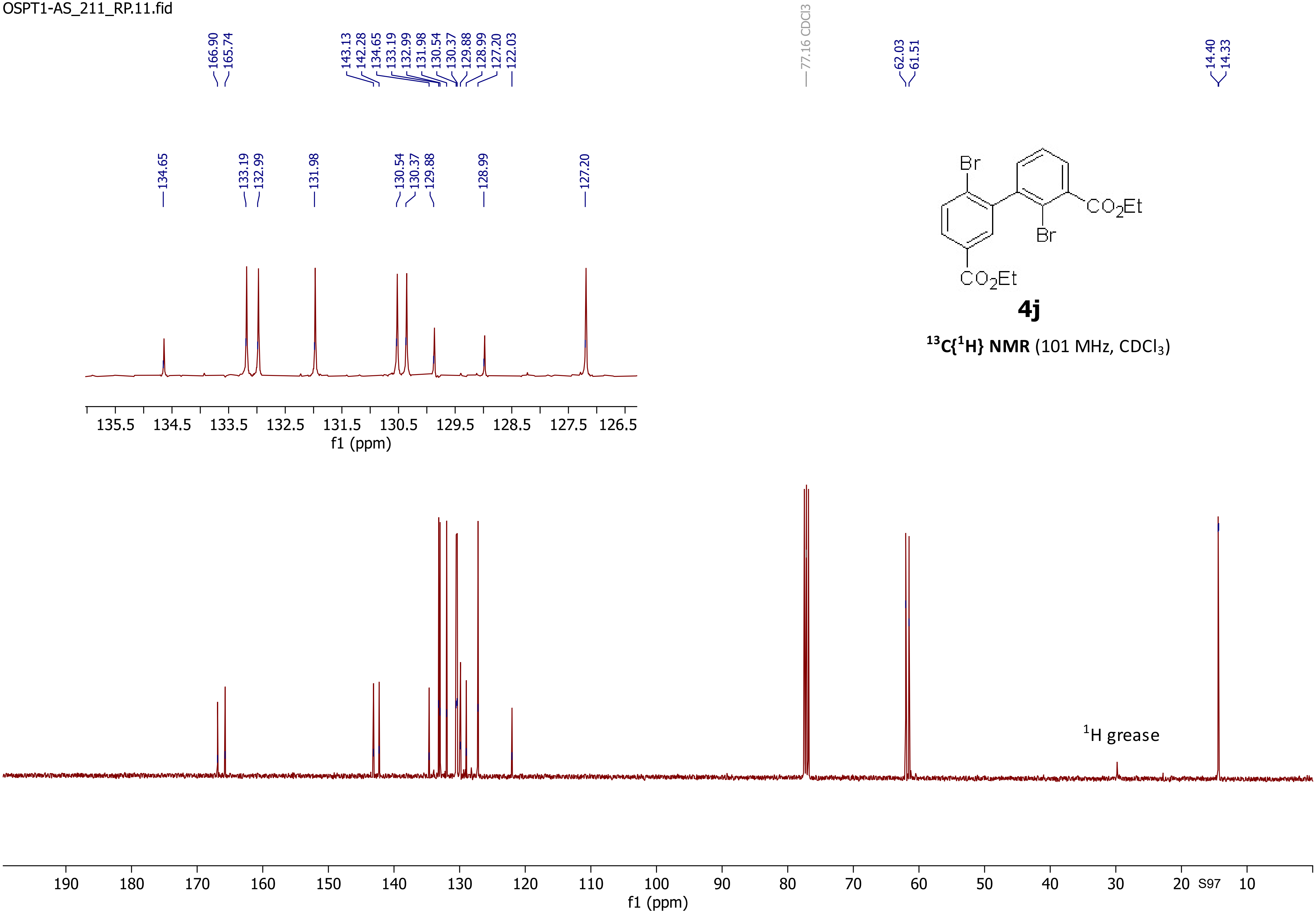

4j

$^{13}C\{^1H\}$ NMR (101 MHz, $CDCl_3$)

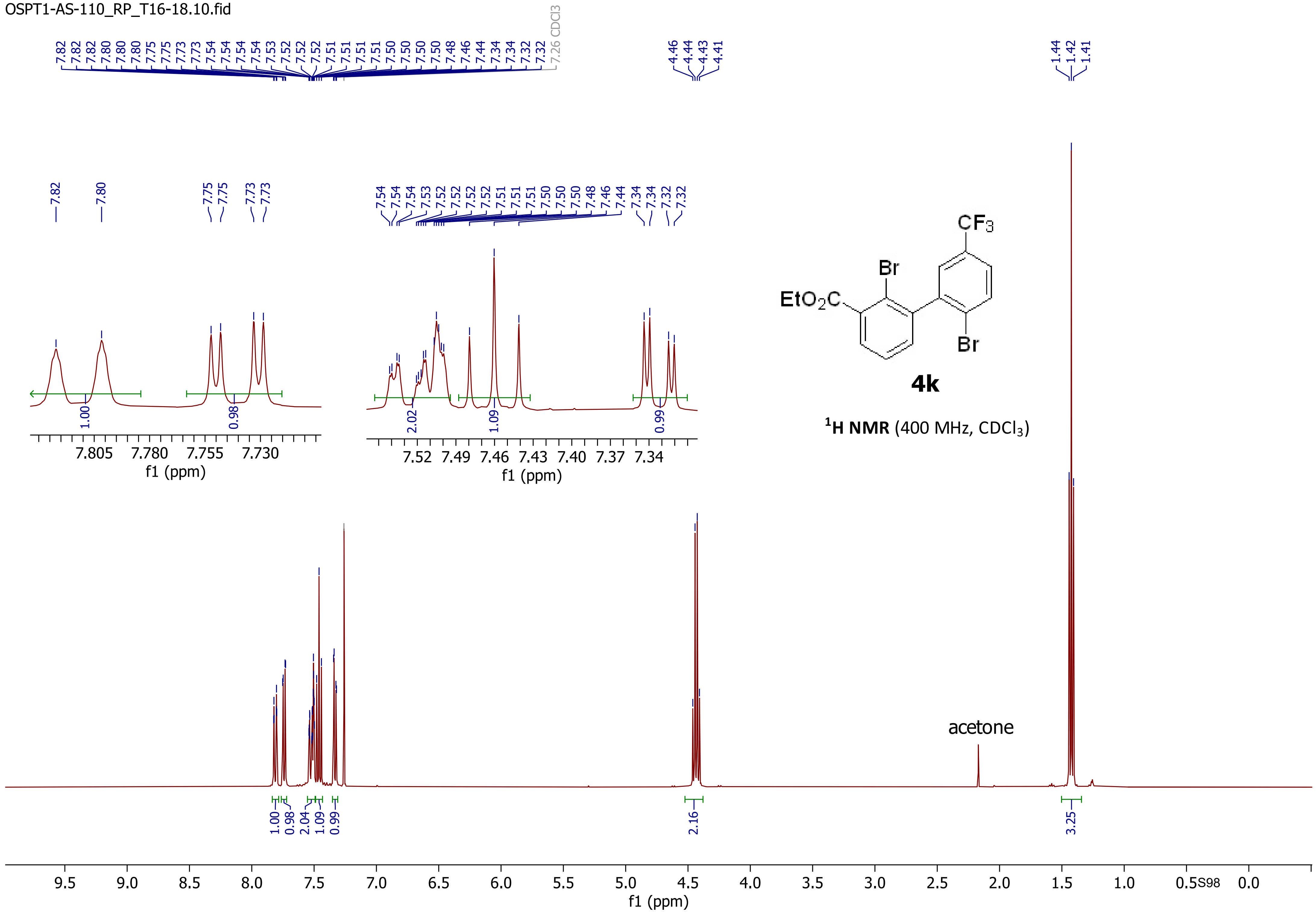
OSPT1-AS-110_RP_T16-18.10.fid
7.82 7.82 7.82 7.80 7.80 7.80 7.75 7.75 7.73 7.73 7.54 7.54 7.54 7.54 7.53 7.52 7.52 7.52 7.51 7.51 7.51 7.50 7.50 7.50 7.50 7.48 7.46 7.44 7.34 7.34 7.32 7.32 7.26 CDCl3
4.46 4.44 4.43 4.41
1.44 1.42 1.41
1.00 0.98 2.04 1.09 0.99 2.16 3.25
2.02
EtO2C
Br
CF3
Br
4k
1H NMR (400 MHz, CDCl3)
acetone
f1 (ppm)

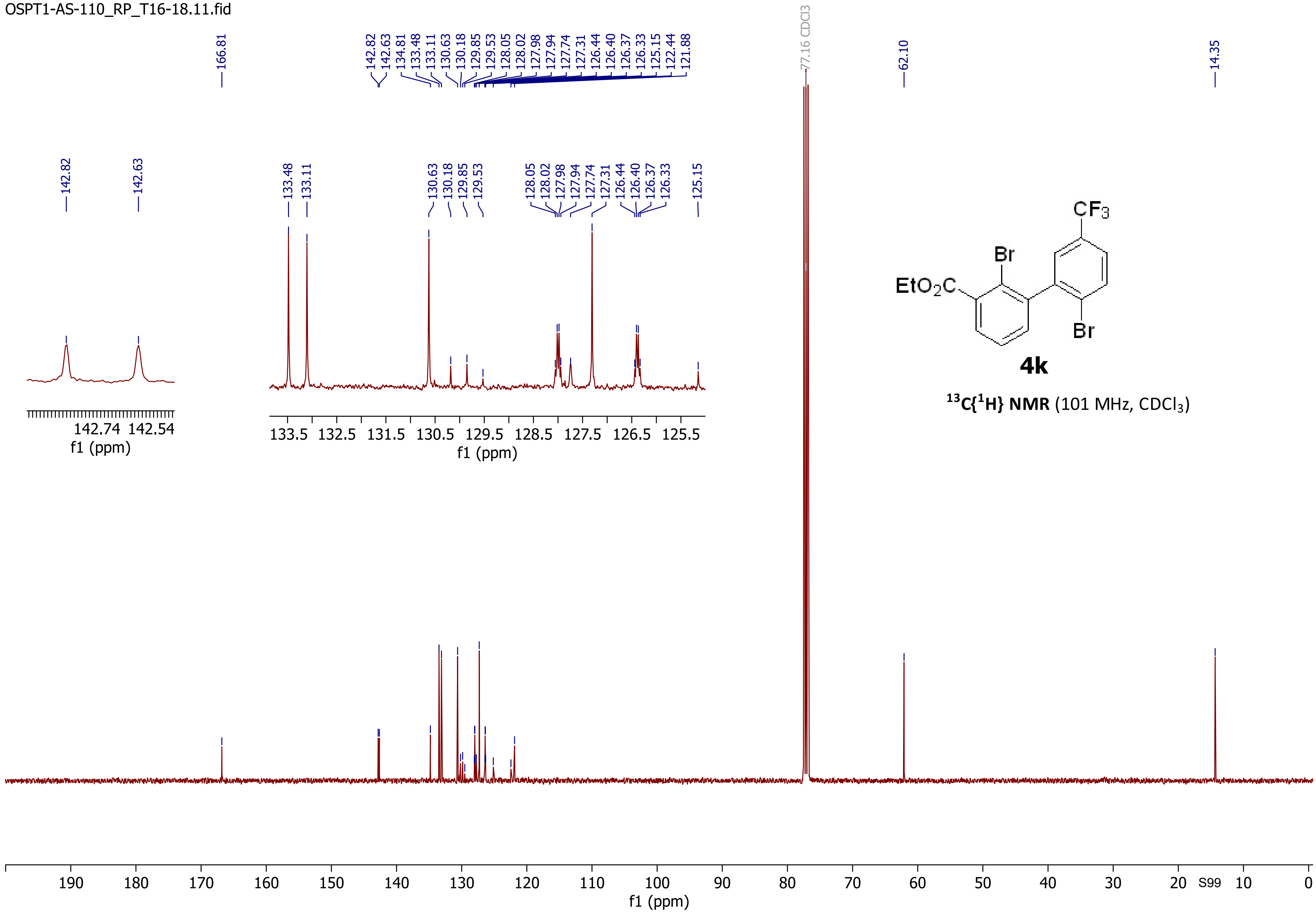
OSPT1-AS-110_RP_T16-18.11.fid
166.81
142.82
142.63
134.81
133.48
133.11
130.63
130.18
129.85
129.53
128.05
128.02
127.98
127.94
127.74
127.31
126.44
126.40
126.37
126.33
125.15
122.44
121.88
77.16 CDCl3
62.10
14.35
142.74 142.54
f1 (ppm)
133.5 132.5 131.5 130.5 129.5 128.5 127.5 126.5 125.5
f1 (ppm)
CF3
Br
EtO2C
Br
4k
13C{1H} NMR (101 MHz, CDCl3)
190 180 170 160 150 140 130 120 110 100 90 80 70 60 50 40 30 20 10 0
f1 (ppm)

OSPT1-AS-110_RP_T16-18.12.1.1r

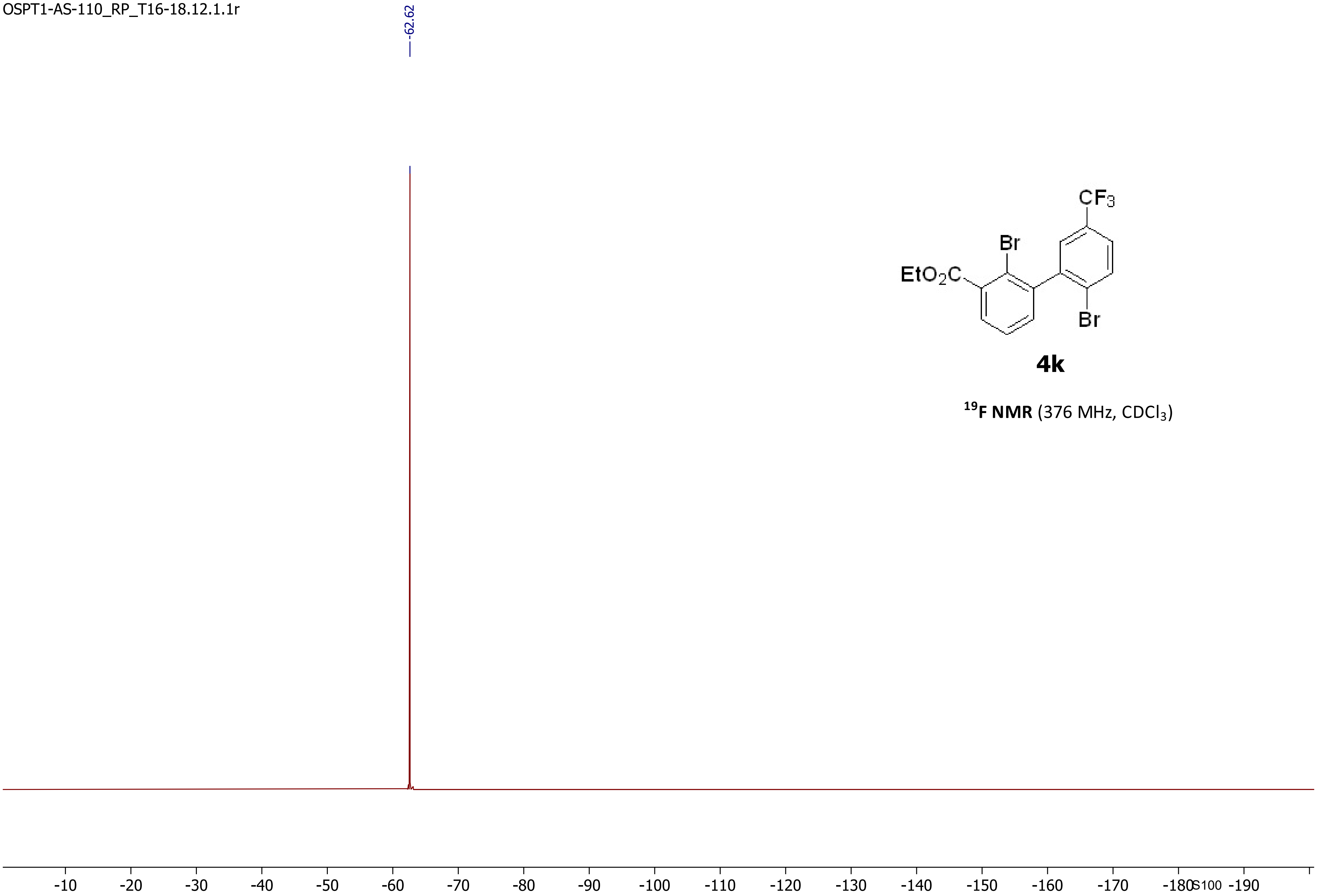

4k

^{19}F NMR (376 MHz, $CDCl_3$)

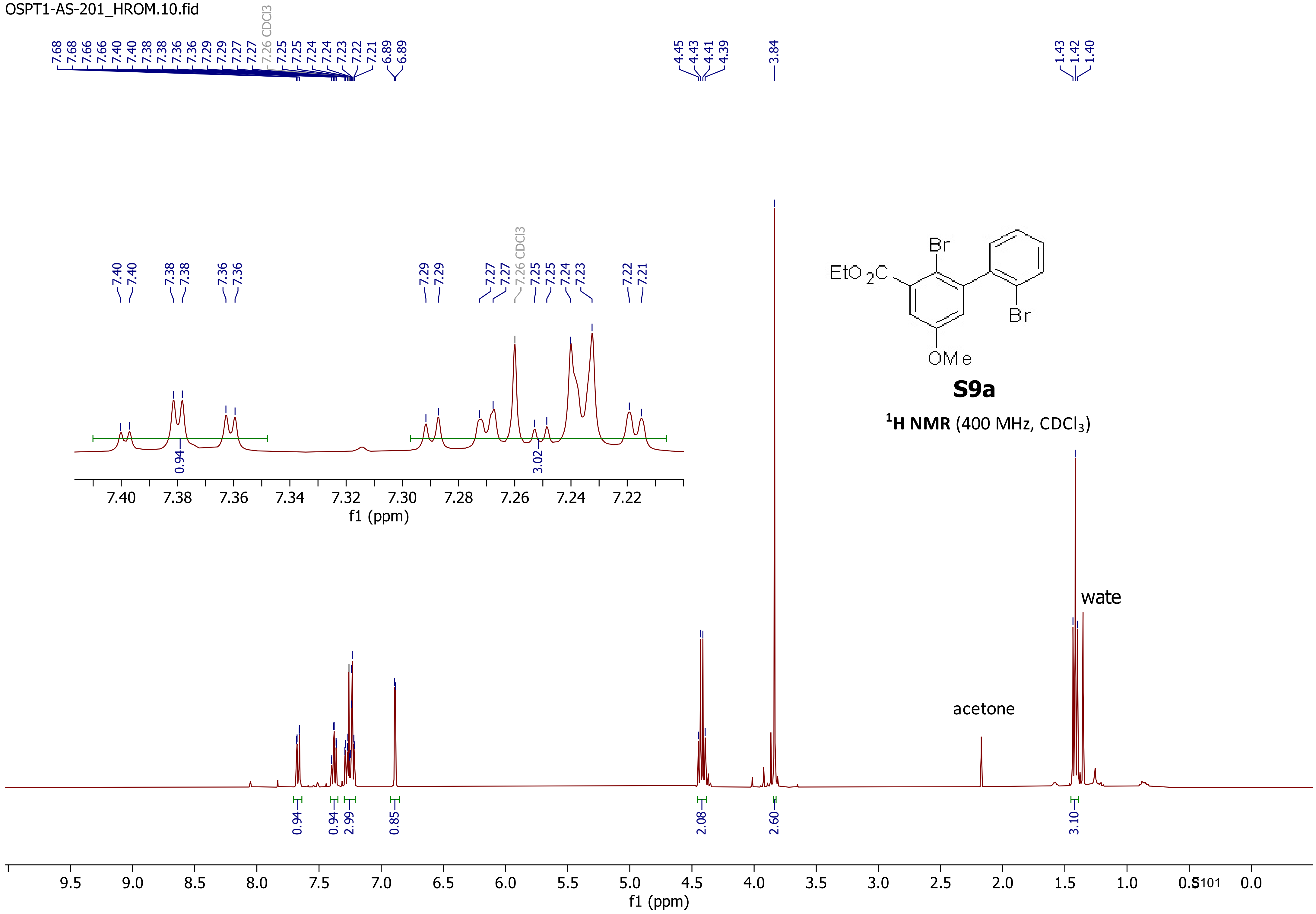
OSPT1-AS-201_HROM.10.fid
EtO2C
Br
Br
OMe
S9a
1H NMR (400 MHz, CDCl3)
acetone
wate
7.26 CDCl3
f1 (ppm)

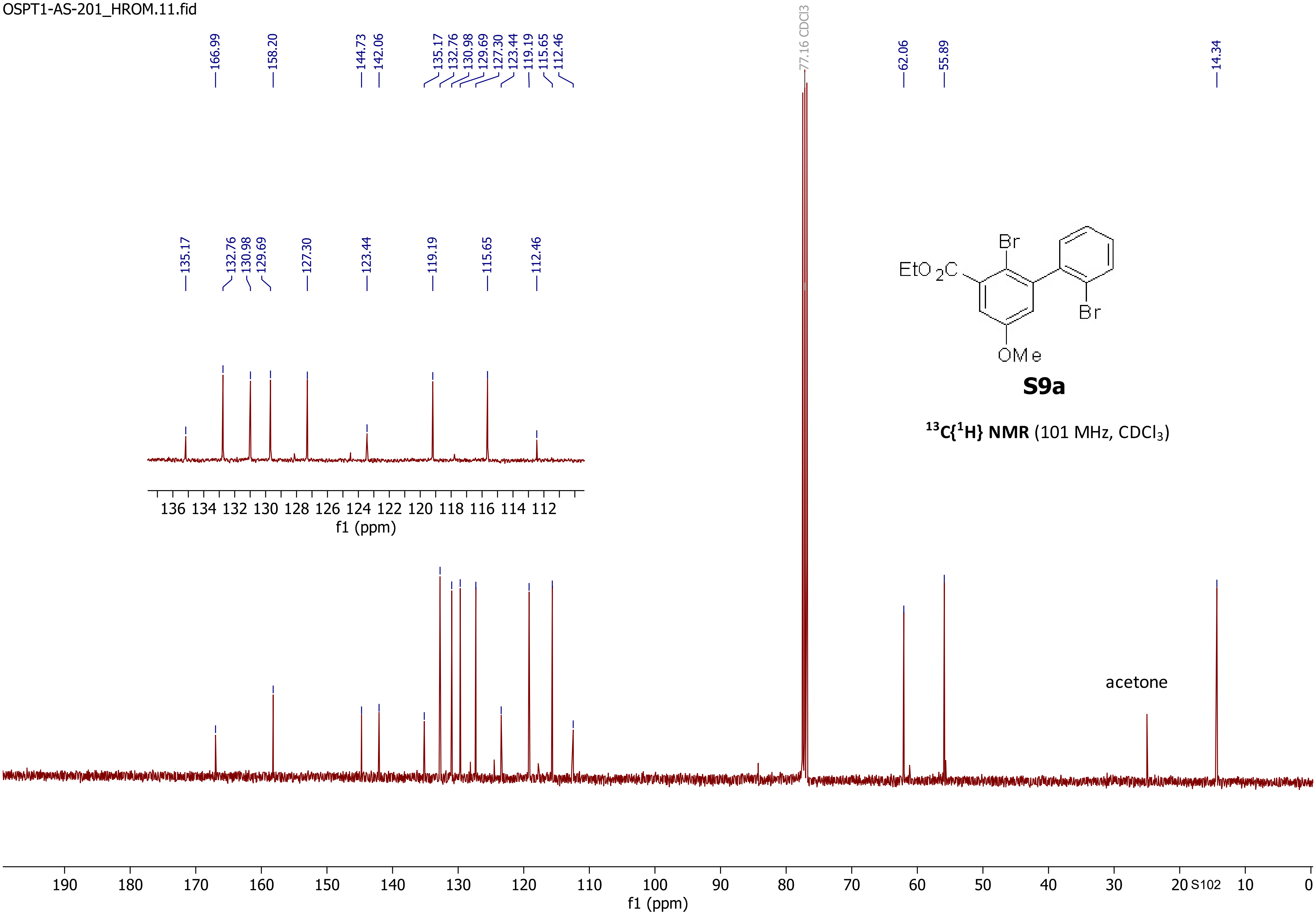
OSPT1-AS-201_HROM.11.fid
166.99
158.20
144.73
142.06
135.17
132.76
130.98
129.69
127.30
123.44
119.19
115.65
112.46
77.16 CDCl3
62.06
55.89
14.34
EtO2C
Br
Br
OMe
S9a
13C{1H} NMR (101 MHz, CDCl3)
acetone
190 180 170 160 150 140 130 120 110 100 90 80 70 60 50 40 30 20 10 0
f1 (ppm)
136 134 132 130 128 126 124 122 120 118 116 114 112
f1 (ppm)

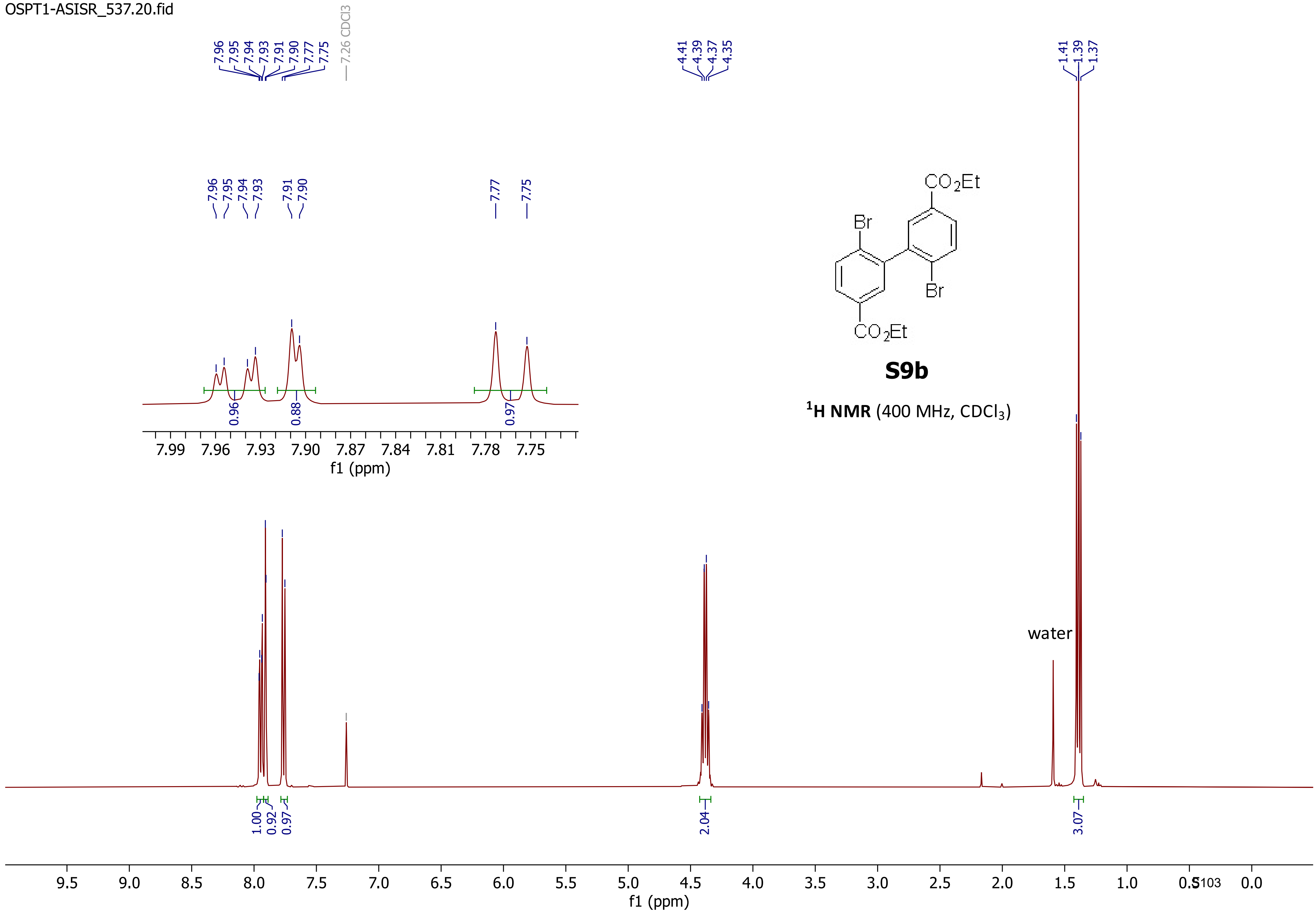
OSPT1-ASISR_537.20.fid
7.96
7.95
7.94
7.93
7.91
7.90
7.77
7.75
7.26 CDCl3
4.41
4.39
4.37
4.35
1.41
1.39
1.37
CO2Et
Br
Br
CO2Et
S9b
1H NMR (400 MHz, CDCl3)
water
0.96
0.88
0.97
1.00
0.92
0.97
2.04
3.07
f1 (ppm)

OSPT1-ASISR_537.21.fid

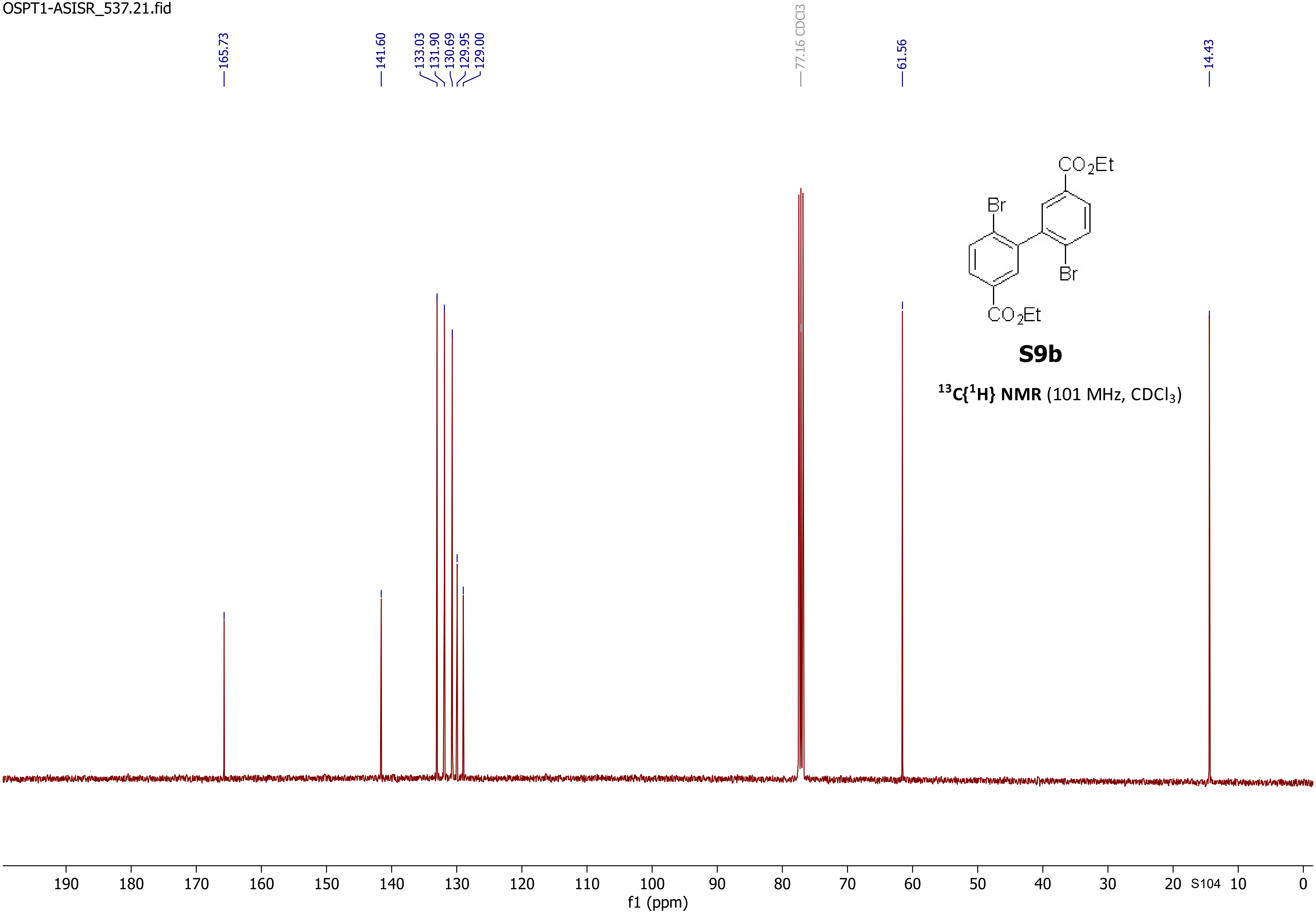

S9b

$^{13}C\{^{1}H\}$ NMR (101 MHz, $CDCl_3$)

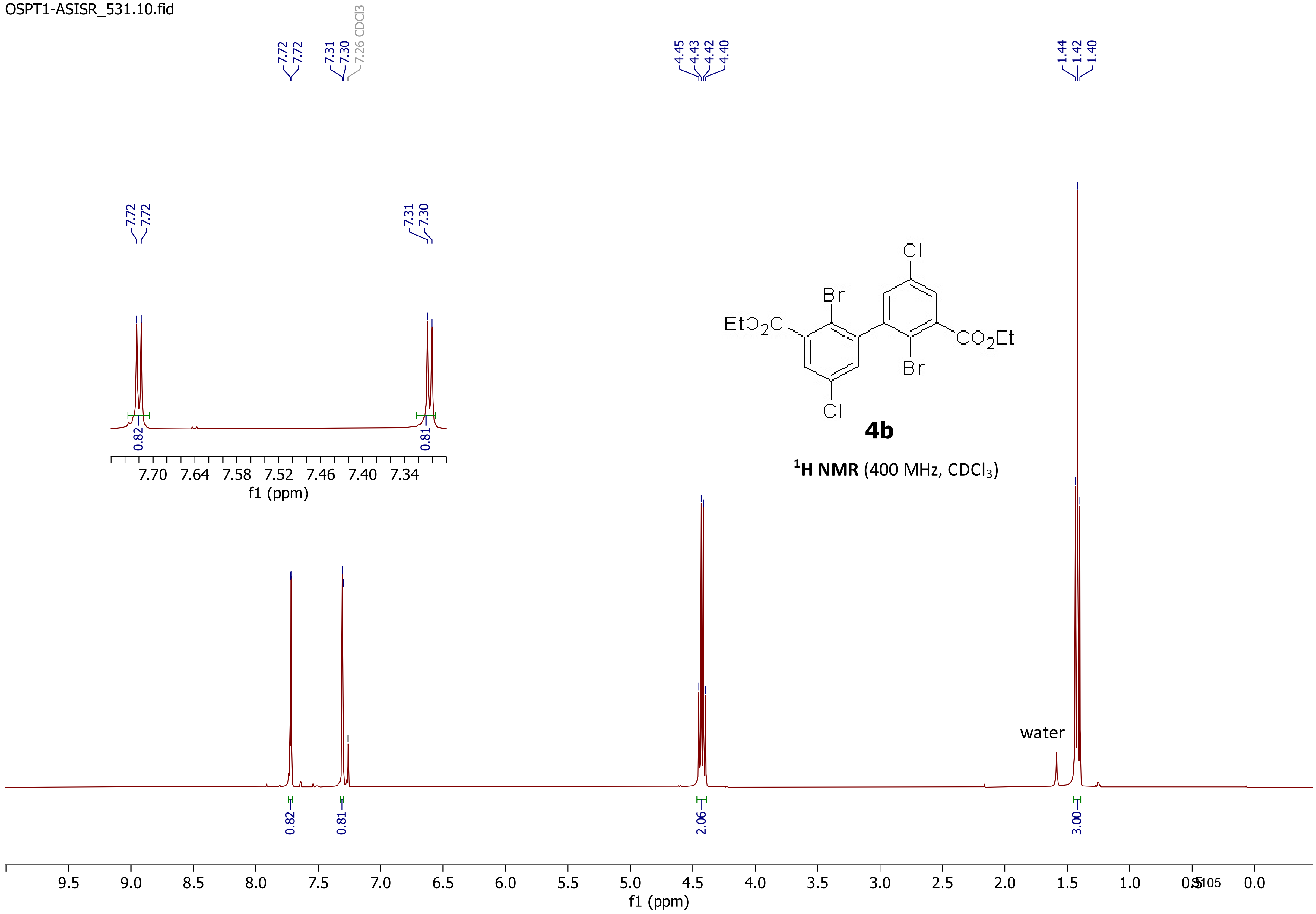

4b

[1]**H NMR** (400 MHz, $CDCl_3$)

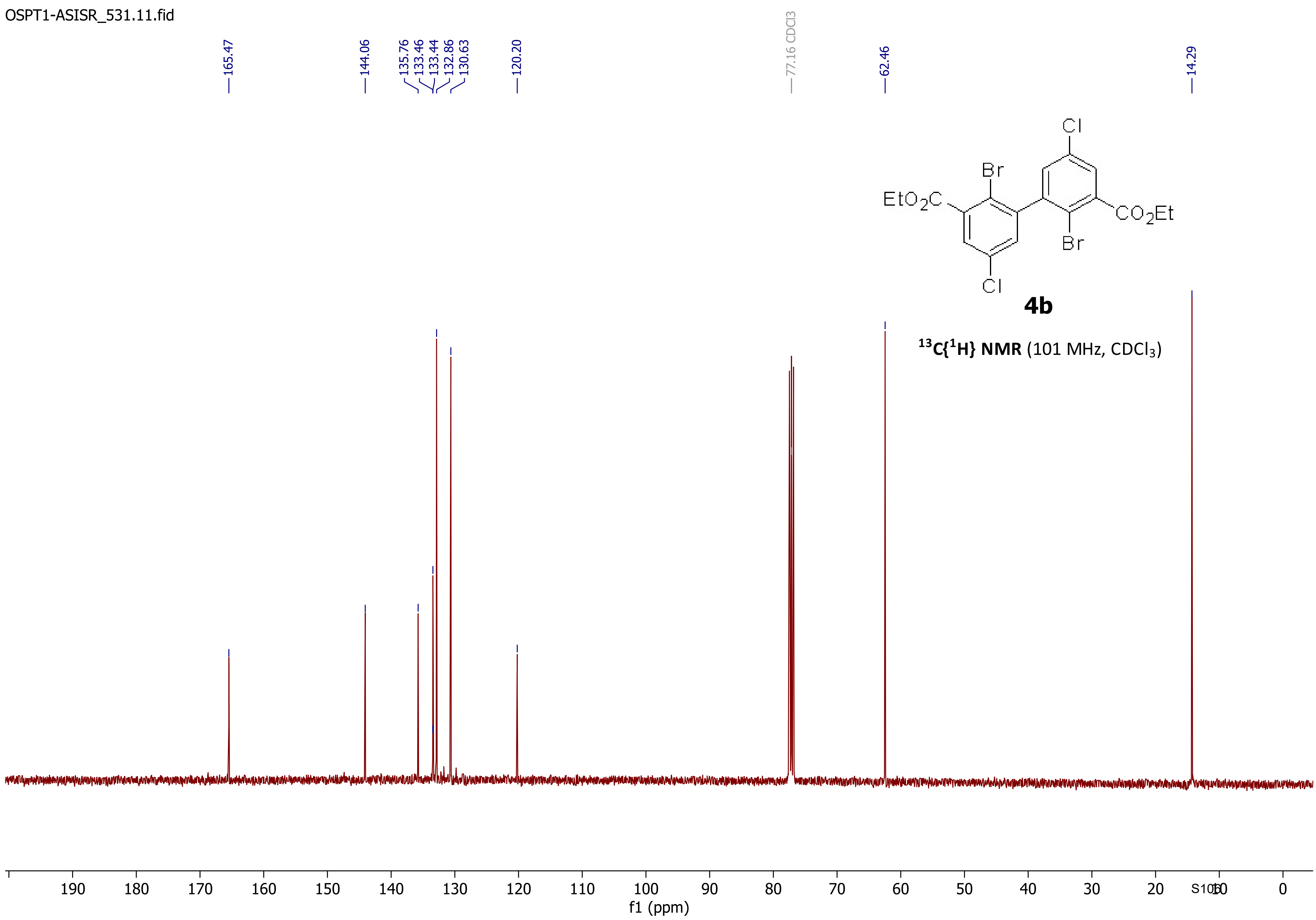
OSPT1-ASISR_531.11.fid
165.47
144.06
135.76
133.46
133.44
132.86
130.63
120.20
77.16 CDCl3
62.46
14.29
Cl
Br
EtO_2C
CO_2Et
Br
Cl
4b
$^{13}C\{^1H\}$ NMR (101 MHz, $CDCl_3$)
190 180 170 160 150 140 130 120 110 100 90 80 70 60 50 40 30 20 10 0
f1 (ppm)

OSPT1-ASISR_530.20.fid

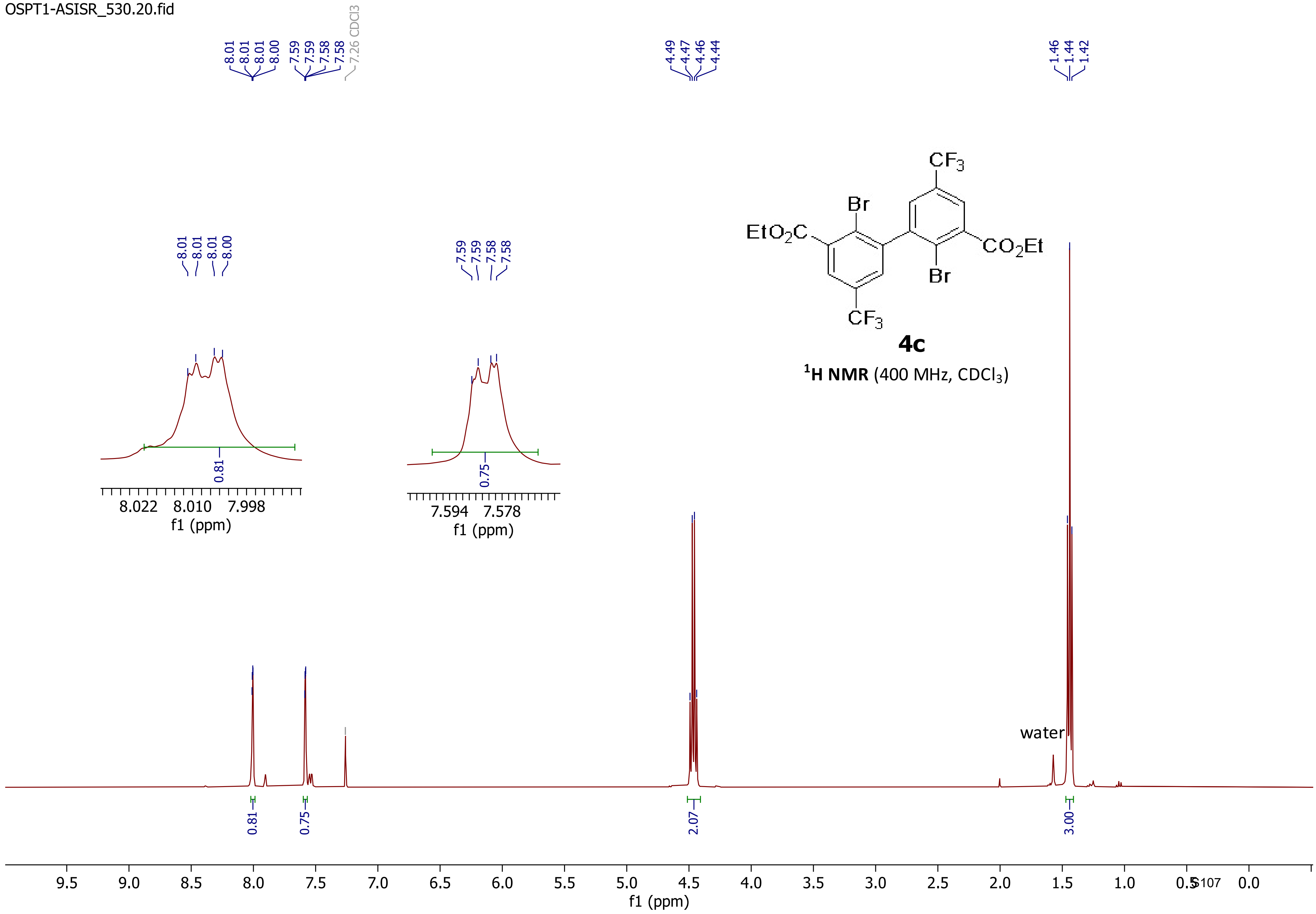

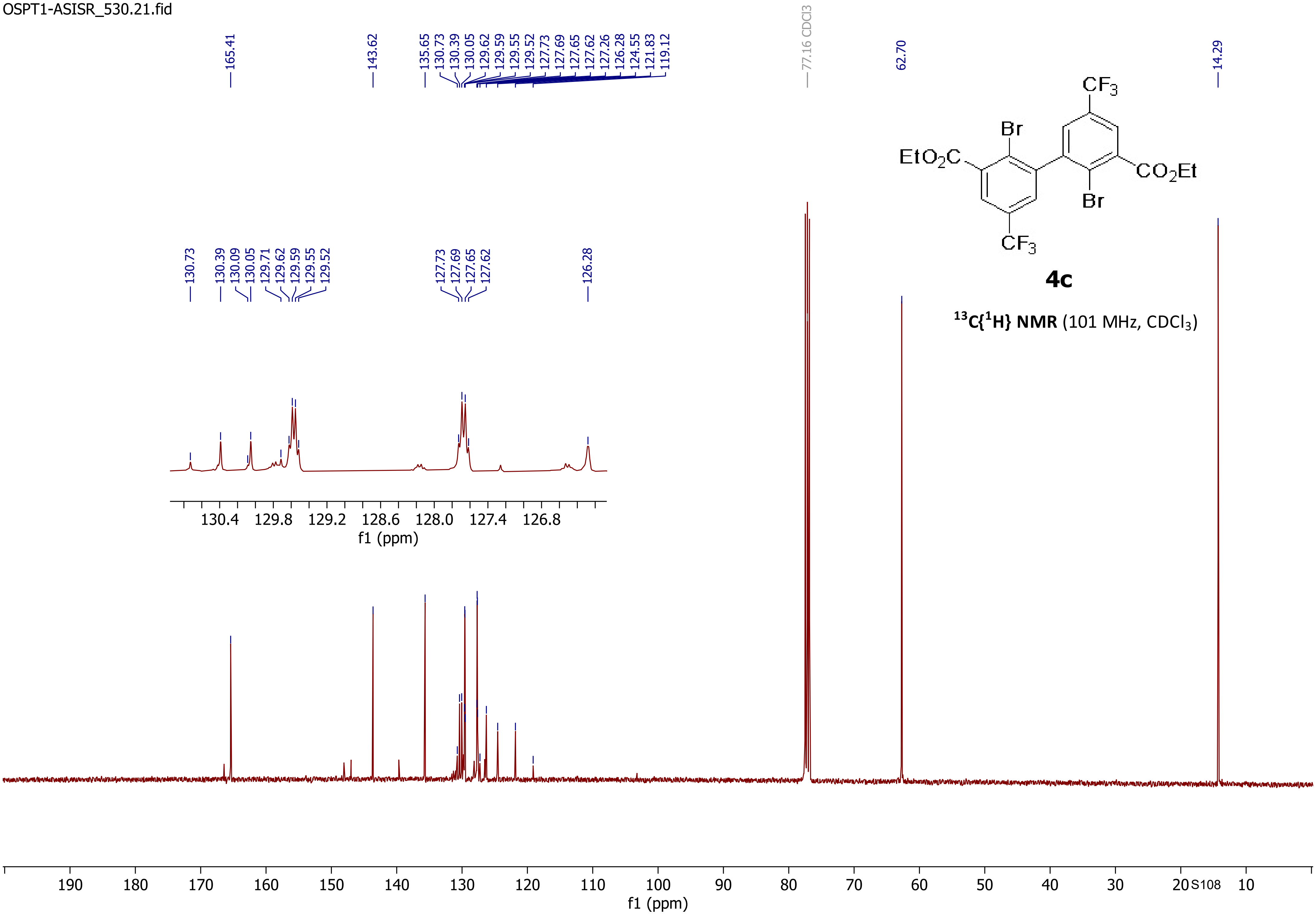
OSPT1-ASISR_530.21.fid
165.41
143.62
135.65
130.73
130.39
130.05
129.62
129.59
129.55
129.52
127.73
127.69
127.65
127.62
127.26
126.28
124.55
121.83
119.12
77.16 CDCl3
62.70
14.29
130.73
130.39
130.09
130.05
129.71
129.62
129.59
129.55
129.52
127.73
127.69
127.65
127.62
126.28
130.4 129.8 129.2 128.6 128.0 127.4 126.8
f1 (ppm)
CF3
Br
EtO2C
CO2Et
Br
CF3
4c
13C{1H} NMR (101 MHz, CDCl3)
190 180 170 160 150 140 130 120 110 100 90 80 70 60 50 40 30 20 10
f1 (ppm)

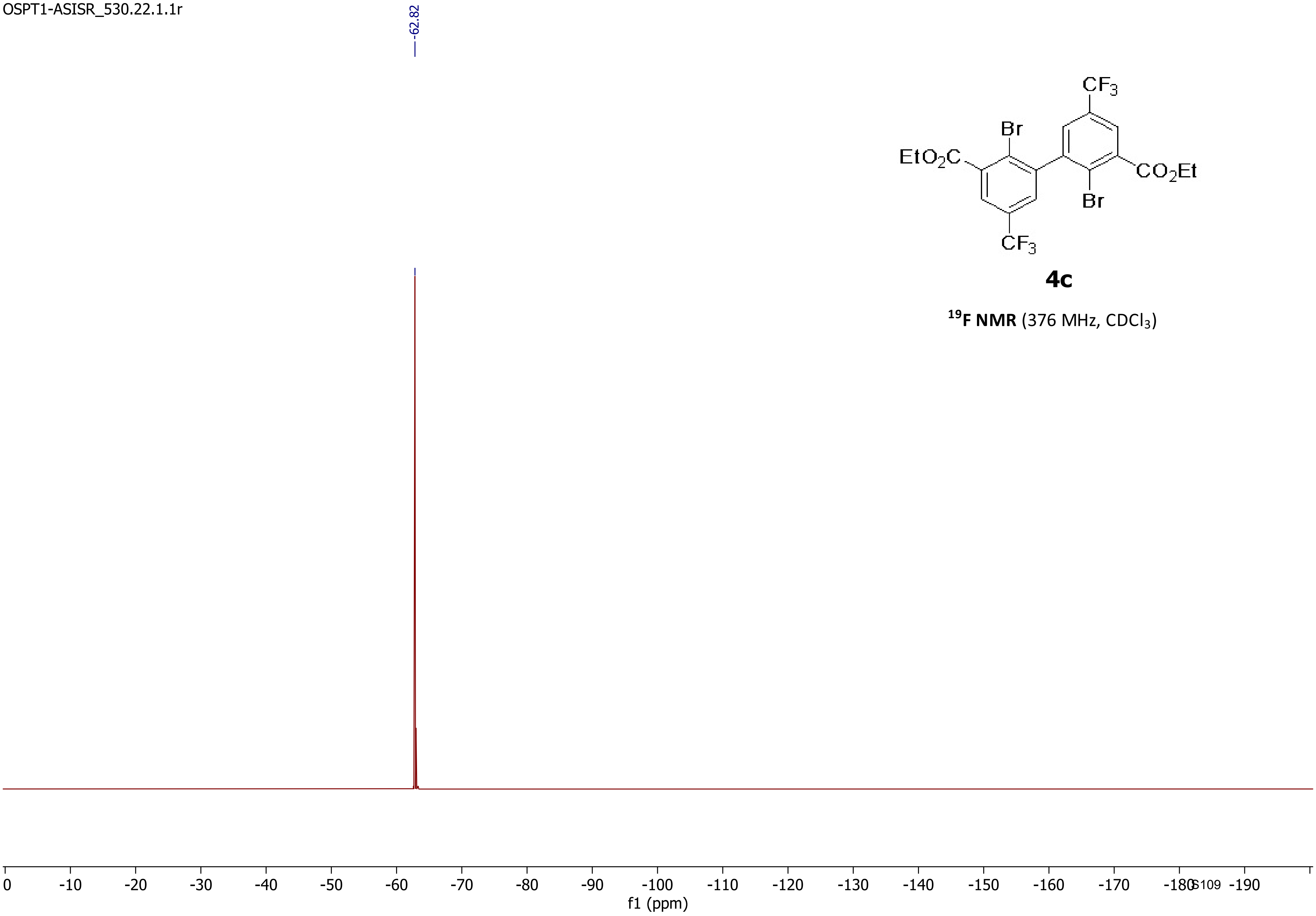

4c

^{19}F NMR (376 MHz, $CDCl_3$)

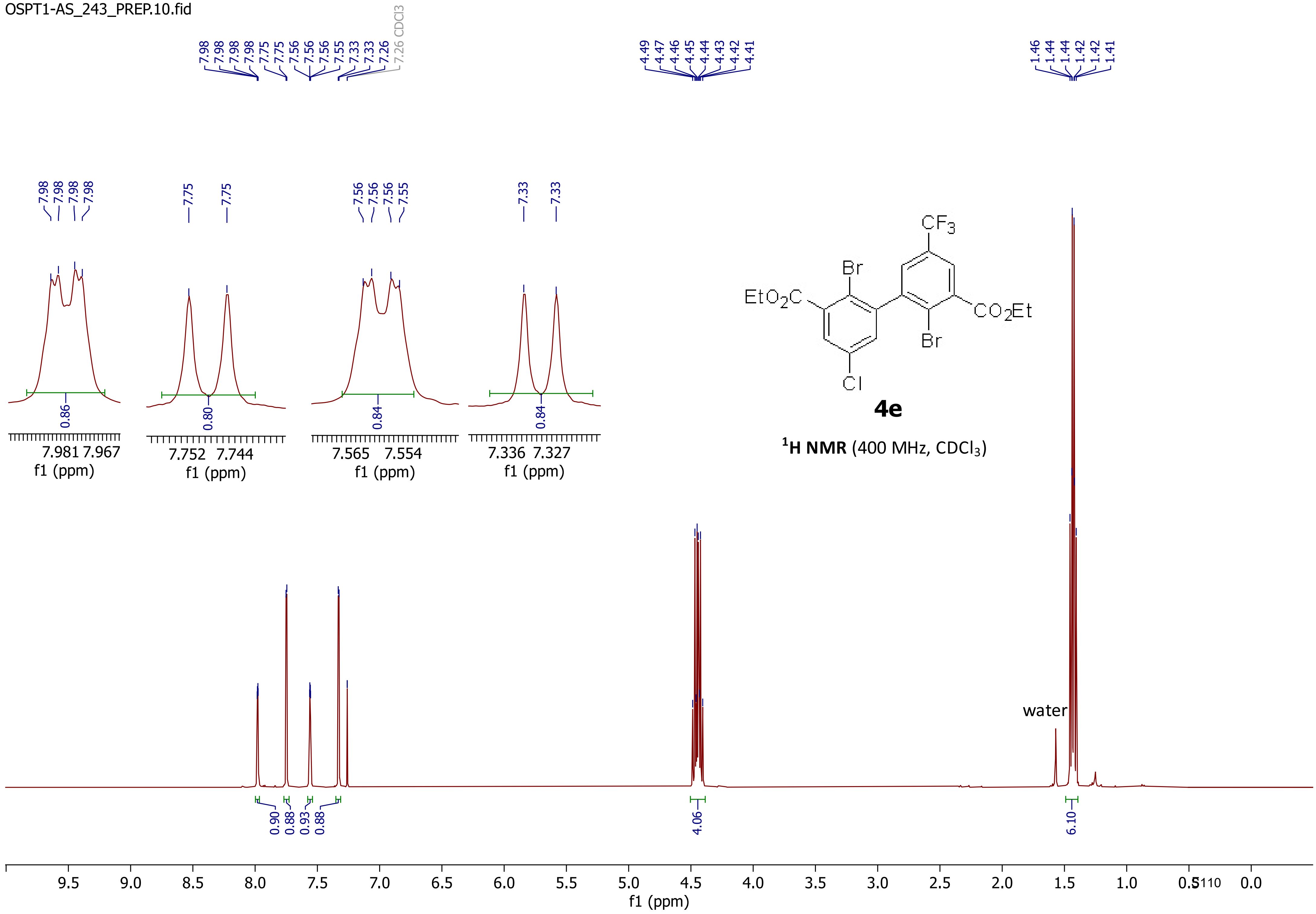
OSPT1-AS_243_PREP.10.fid
7.98 7.98 7.98 7.98 7.75 7.75 7.56 7.56 7.56 7.55 7.33 7.33 7.26 7.26 CDCl3
4.49 4.47 4.46 4.45 4.44 4.43 4.42 4.41
1.46 1.44 1.44 1.42 1.42 1.41
0.86
7.981 7.967
f1 (ppm)
0.80
7.752 7.744
f1 (ppm)
0.84
7.565 7.554
f1 (ppm)
0.84
7.336 7.327
f1 (ppm)
CF3
Br
EtO2C
CO2Et
Br
Cl
4e
1H NMR (400 MHz, CDCl3)
water
0.90 0.88 0.93 0.88
4.06
6.10
9.5 9.0 8.5 8.0 7.5 7.0 6.5 6.0 5.5 5.0 4.5 4.0 3.5 3.0 2.5 2.0 1.5 1.0 0.5 0.0
f1 (ppm)

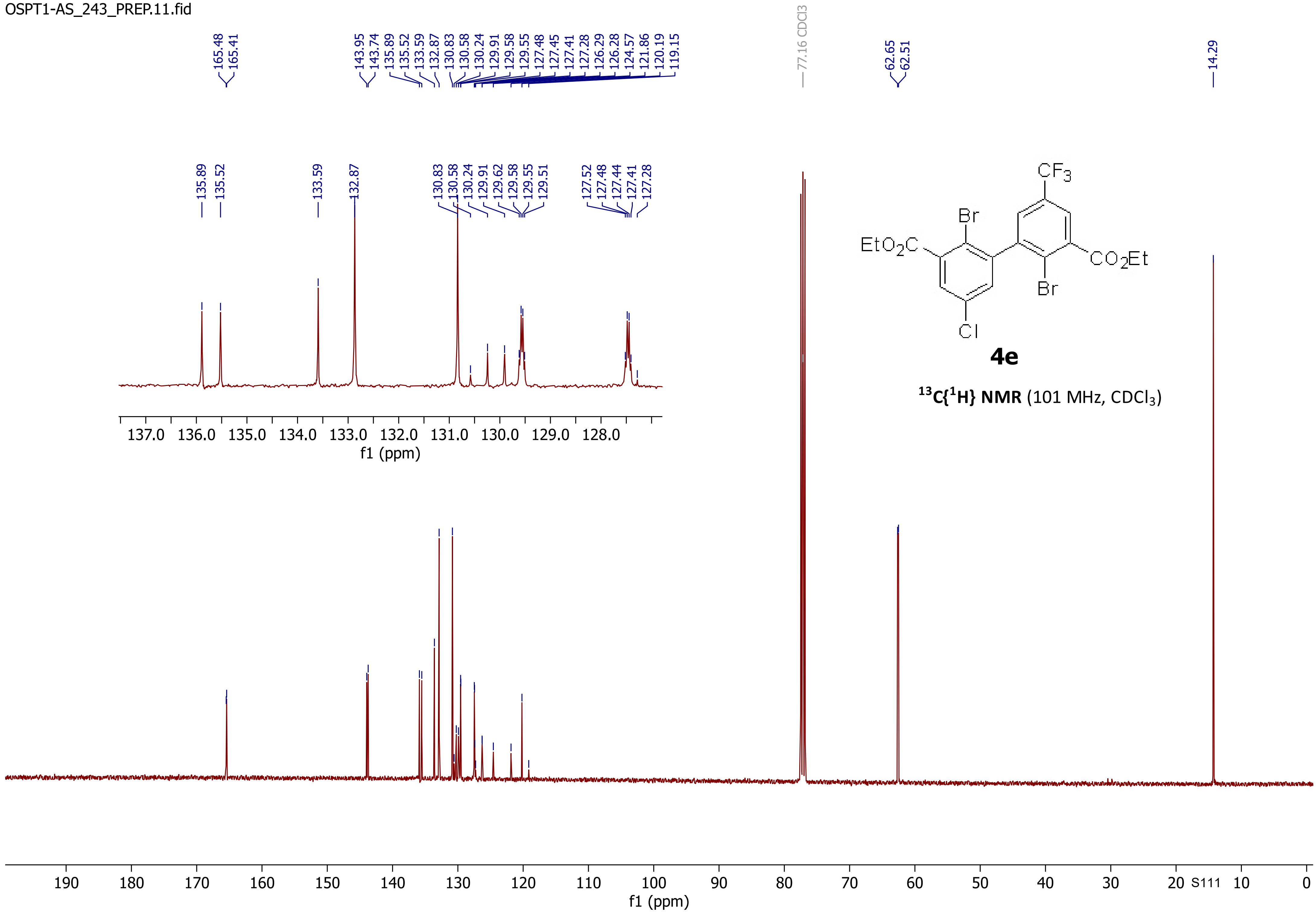
OSPT1-AS_243_PREP.11.fid
165.48
165.41
143.95
143.74
135.89
135.52
133.59
132.87
130.83
130.58
130.24
129.91
129.58
129.55
127.48
127.45
127.41
127.28
126.29
126.28
124.57
121.86
120.19
119.15
77.16 CDCl3
62.65
62.51
14.29
135.89
135.52
133.59
132.87
130.83
130.58
130.24
129.91
129.62
129.58
129.55
129.51
127.52
127.48
127.44
127.41
127.28
137.0 136.0 135.0 134.0 133.0 132.0 131.0 130.0 129.0 128.0
f1 (ppm)
CF3
Br
EtO2C
CO2Et
Br
Cl
4e
13C{1H} NMR (101 MHz, CDCl3)
190 180 170 160 150 140 130 120 110 100 90 80 70 60 50 40 30 20 10 0
f1 (ppm)

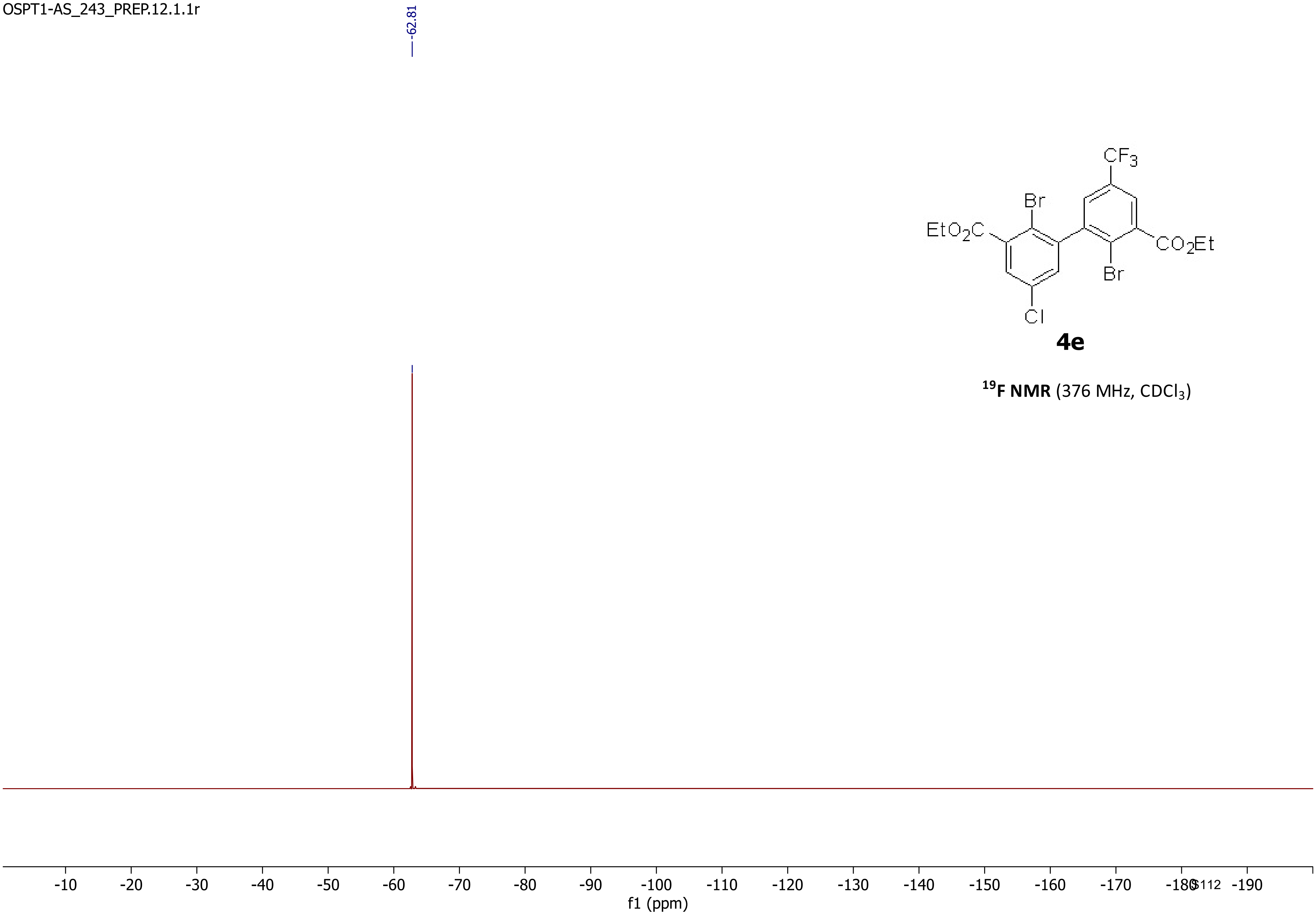

4e

^{19}F NMR (376 MHz, $CDCl_3$)

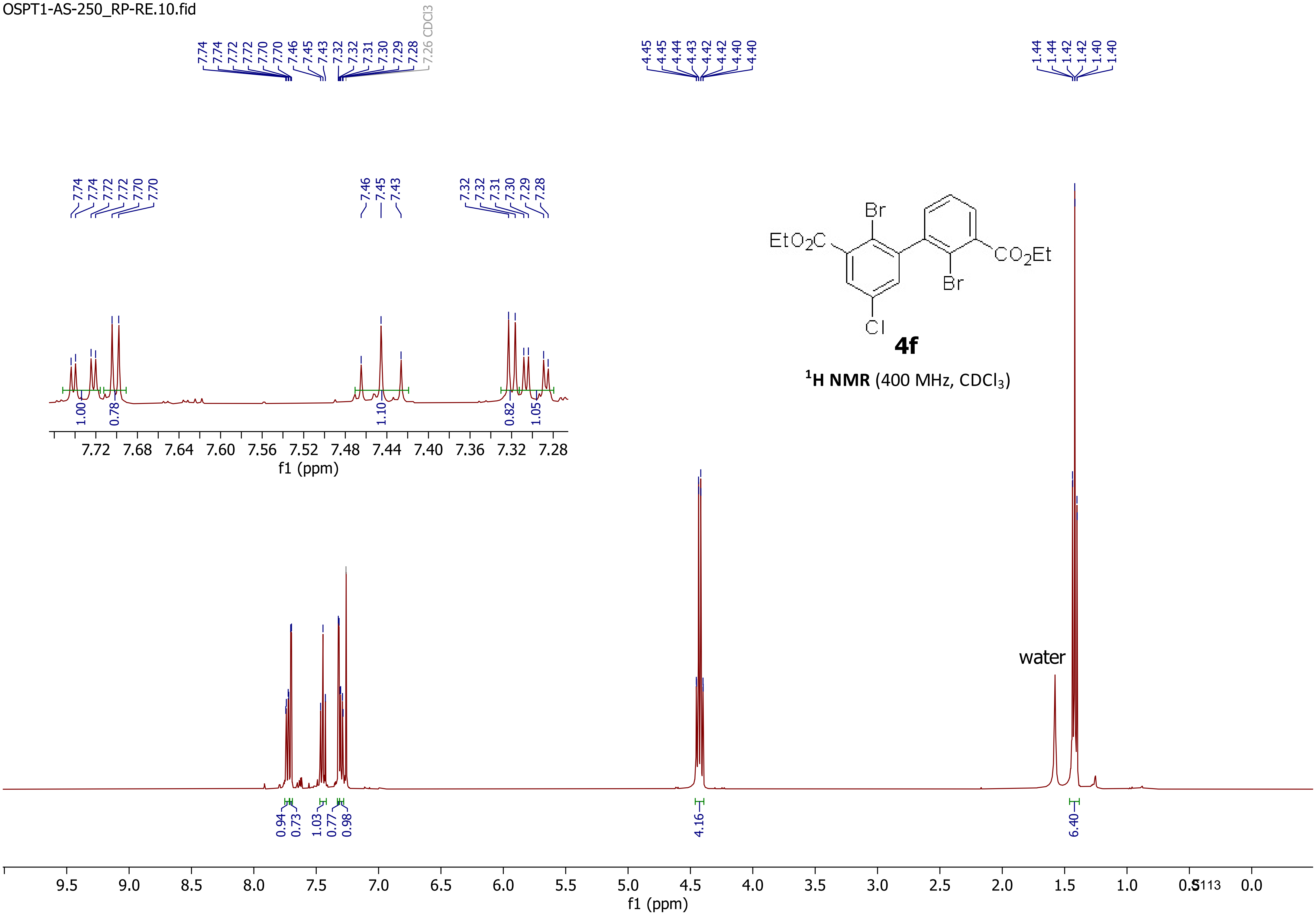
OSPT1-AS-250_RP-RE.10.fid
7.74 7.74 7.72 7.72 7.70 7.70 7.46 7.45 7.43 7.32 7.32 7.31 7.30 7.29 7.28 7.26 CDCl3
4.45 4.45 4.44 4.43 4.42 4.42 4.40 4.40
1.44 1.44 1.42 1.42 1.40 1.40
Br
EtO2C
CO2Et
Br
Cl
4f
1H NMR (400 MHz, CDCl3)
1.00 0.78 1.10 0.82 1.05
f1 (ppm)
water
0.94 0.73 1.03 0.77 0.98
4.16
6.40
f1 (ppm)

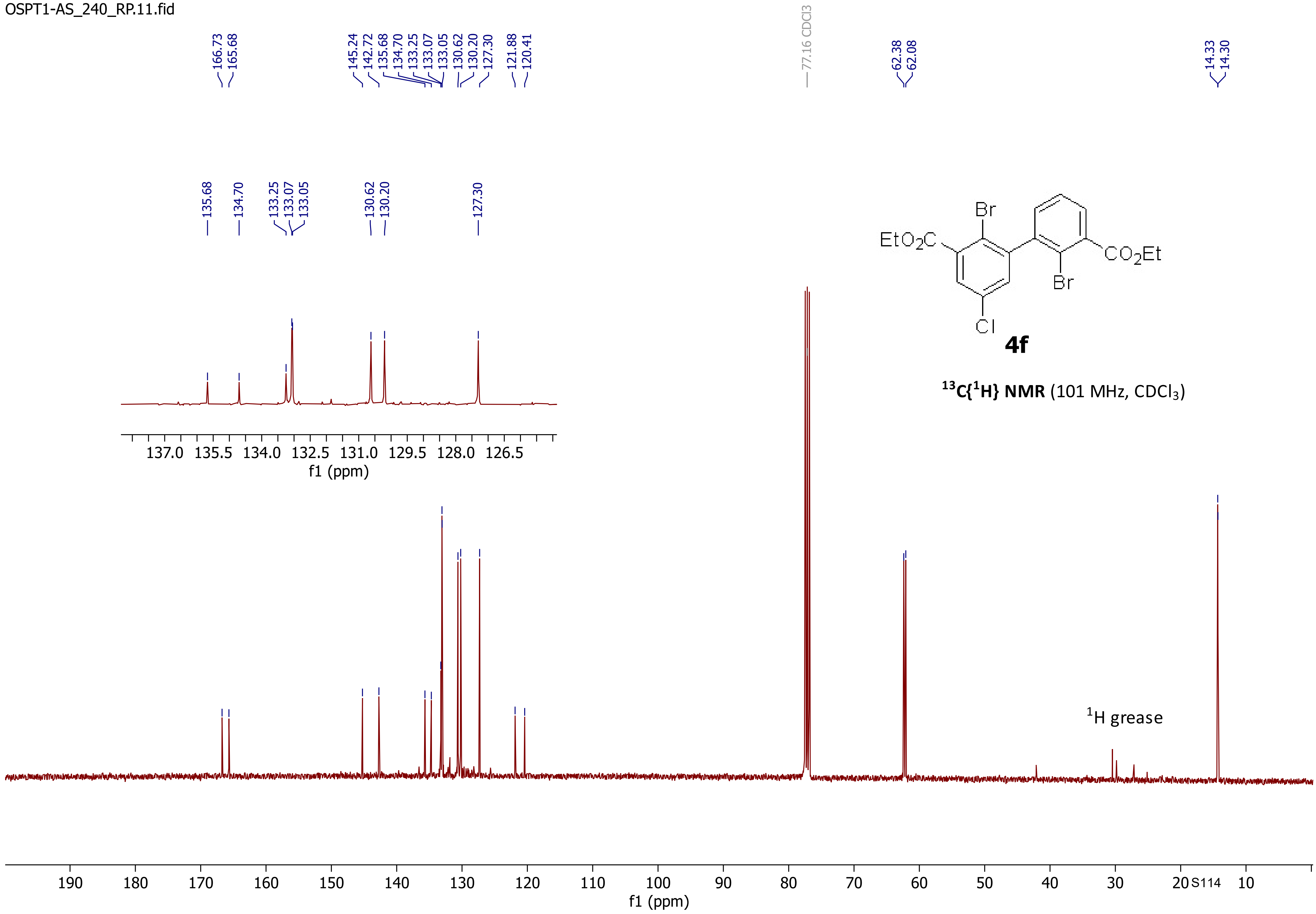

4f

^{13}**C{^{1}H} NMR** (101 MHz, $CDCl_3$)

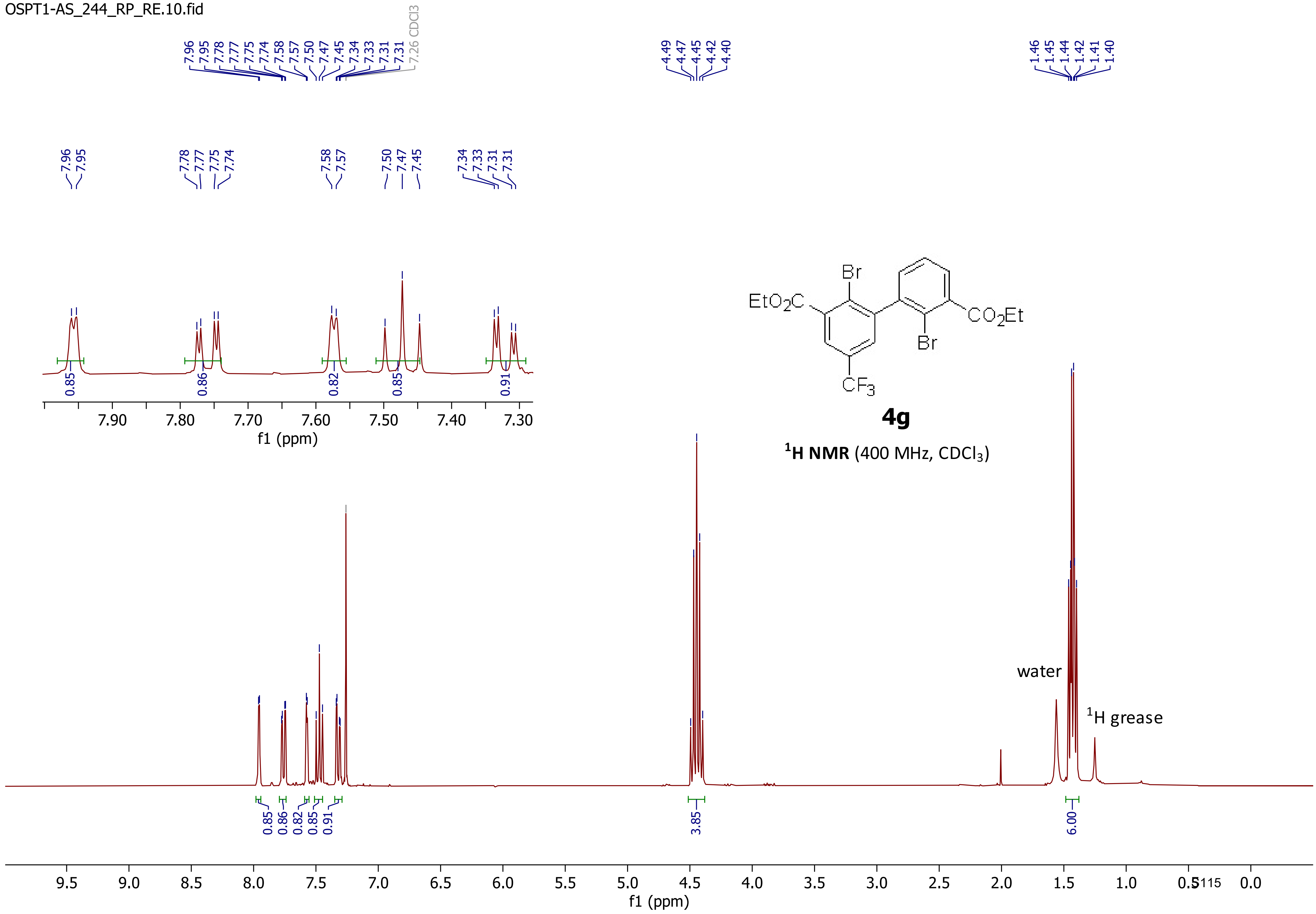
OSPT1-AS_244_RP_RE.10.fid
7.96 7.95 7.78 7.77 7.75 7.74 7.58 7.57 7.50 7.47 7.45 7.34 7.33 7.31 7.31 7.26 CDCl3
4.49 4.47 4.45 4.42 4.40
1.46 1.45 1.44 1.42 1.41 1.40
0.85 0.86 0.82 0.85 0.91
7.90 7.80 7.70 7.60 7.50 7.40 7.30
f1 (ppm)
Br
EtO2C
CO2Et
Br
CF3
4g
1H NMR (400 MHz, CDCl3)
water
1H grease
3.85
6.00
9.5 9.0 8.5 8.0 7.5 7.0 6.5 6.0 5.5 5.0 4.5 4.0 3.5 3.0 2.5 2.0 1.5 1.0 0.5 0.0
f1 (ppm)
115

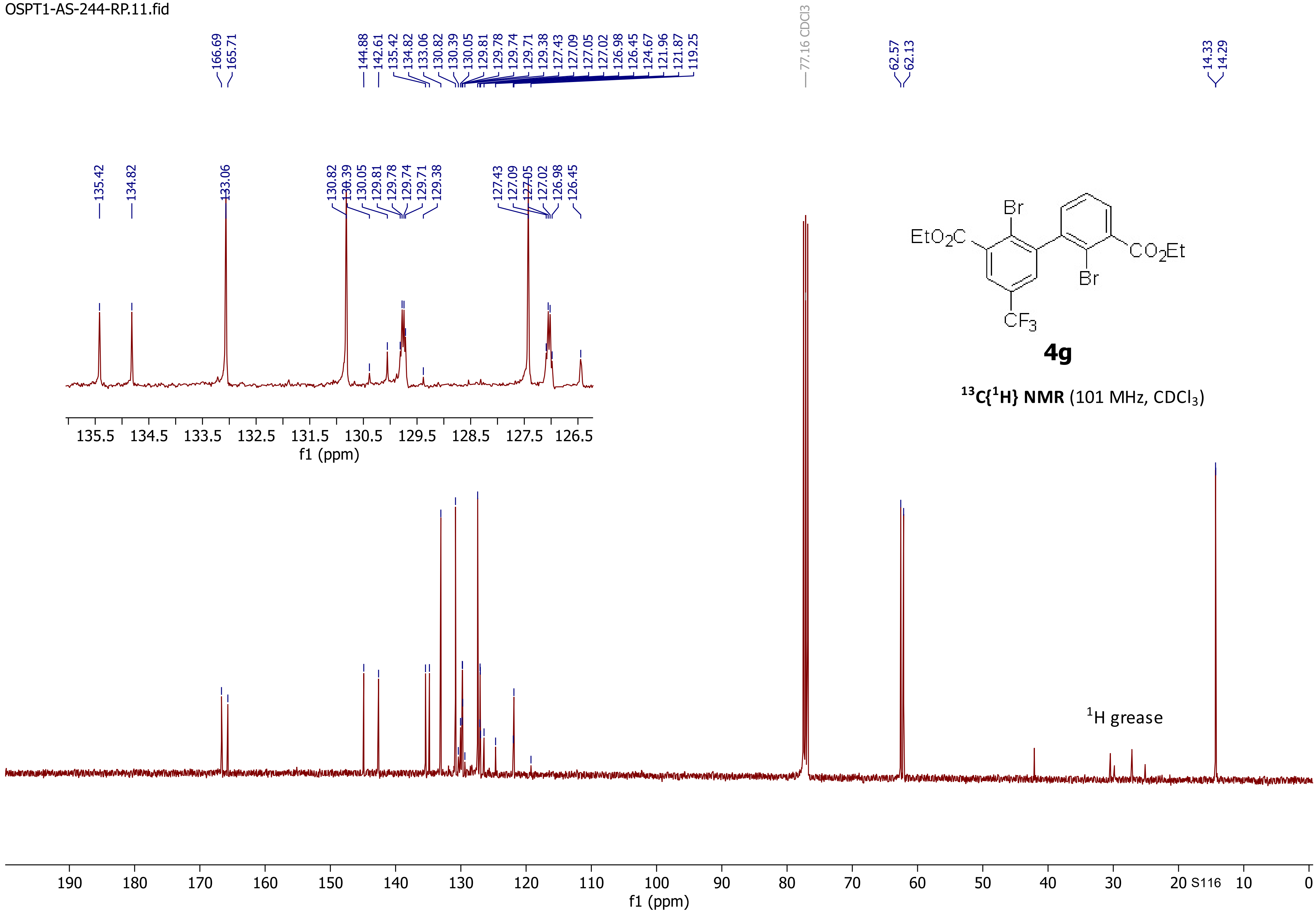
OSPT1-AS-244-RP.11.fid
166.69
165.71
144.88
142.61
135.42
134.82
133.06
130.82
130.39
130.05
129.81
129.78
129.74
129.71
129.38
127.43
127.09
127.05
127.02
126.98
126.45
124.67
121.96
121.87
119.25
77.16 CDCl3
62.57
62.13
14.33
14.29
Br
EtO2C
CO2Et
Br
CF3
4g
13C{1H} NMR (101 MHz, CDCl3)
1H grease
f1 (ppm)

OSPT1-AS-244-RP.12.1.1r

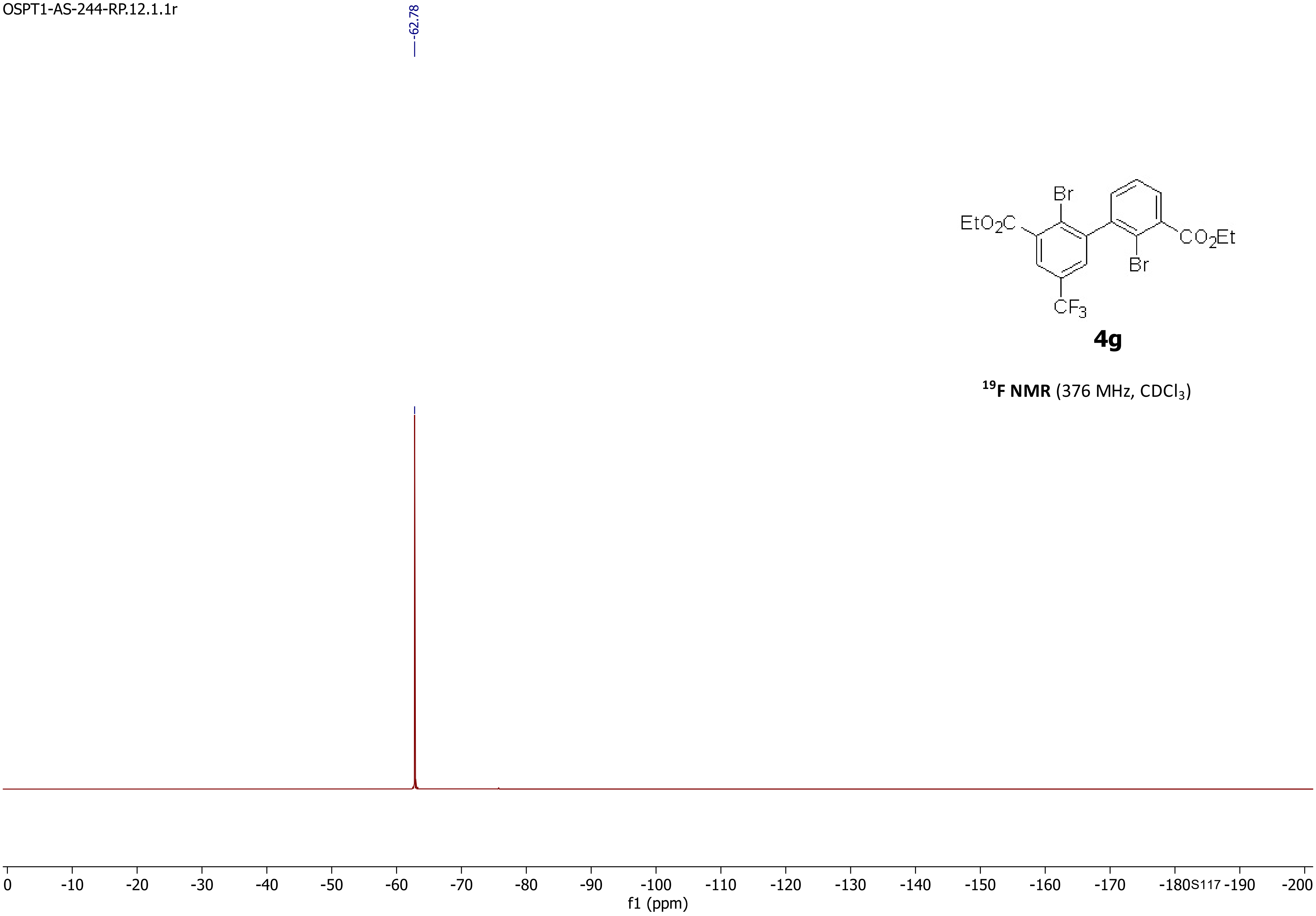

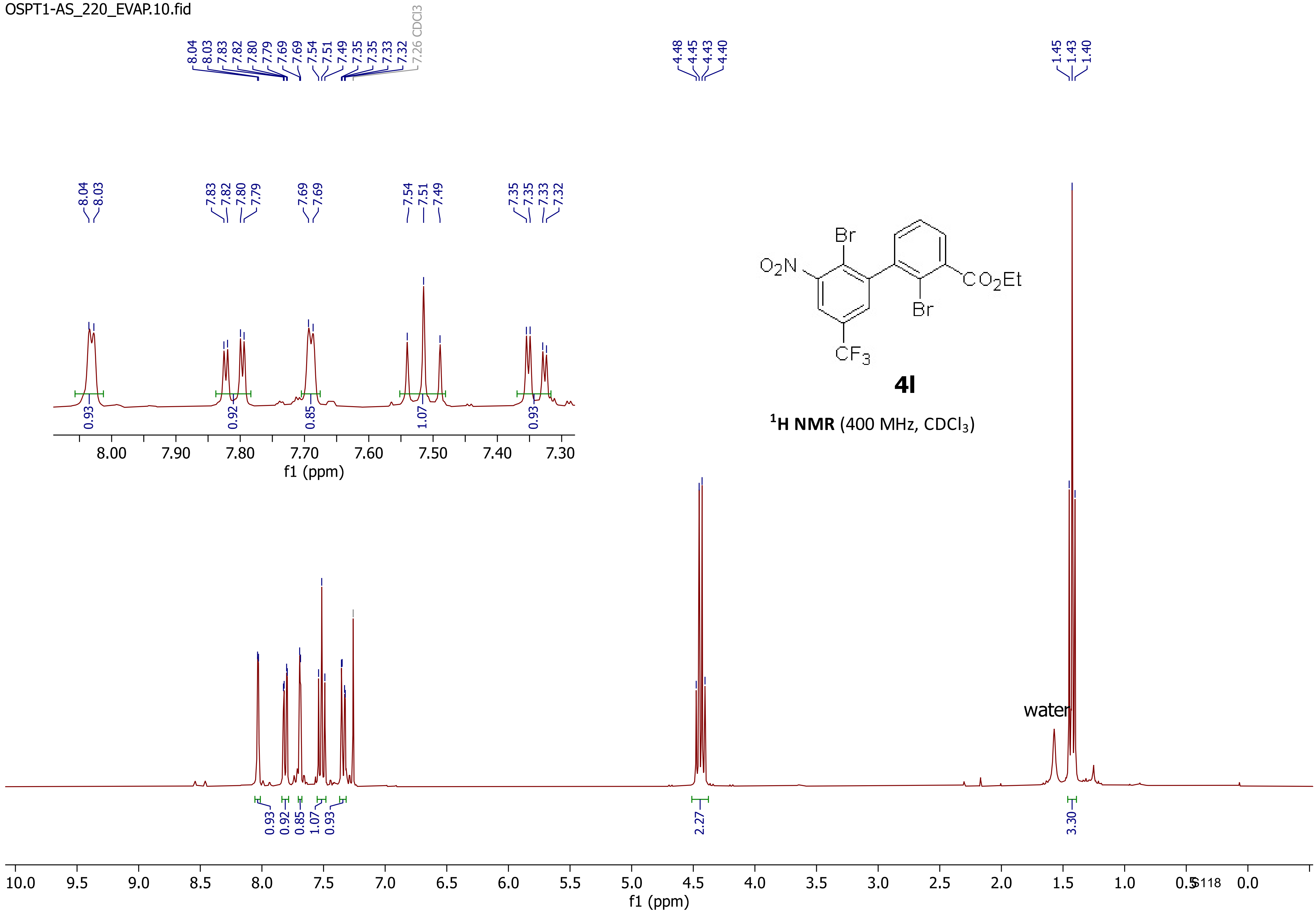
OSPT1-AS_220_EVAP.10.fid
8.04 8.03 7.83 7.82 7.80 7.79 7.69 7.69 7.54 7.51 7.49 7.35 7.35 7.33 7.32 7.26 CDCl3
4.48 4.45 4.43 4.40
1.45 1.43 1.40
0.93 0.92 0.85 1.07 0.93
f1 (ppm)
Br
O2N
CO2Et
Br
CF3
4l
1H NMR (400 MHz, CDCl3)
water
0.93 0.92 0.85 1.07 0.93 2.27 3.30
10.0 9.5 9.0 8.5 8.0 7.5 7.0 6.5 6.0 5.5 5.0 4.5 4.0 3.5 3.0 2.5 2.0 1.5 1.0 0.5 0.0
f1 (ppm)

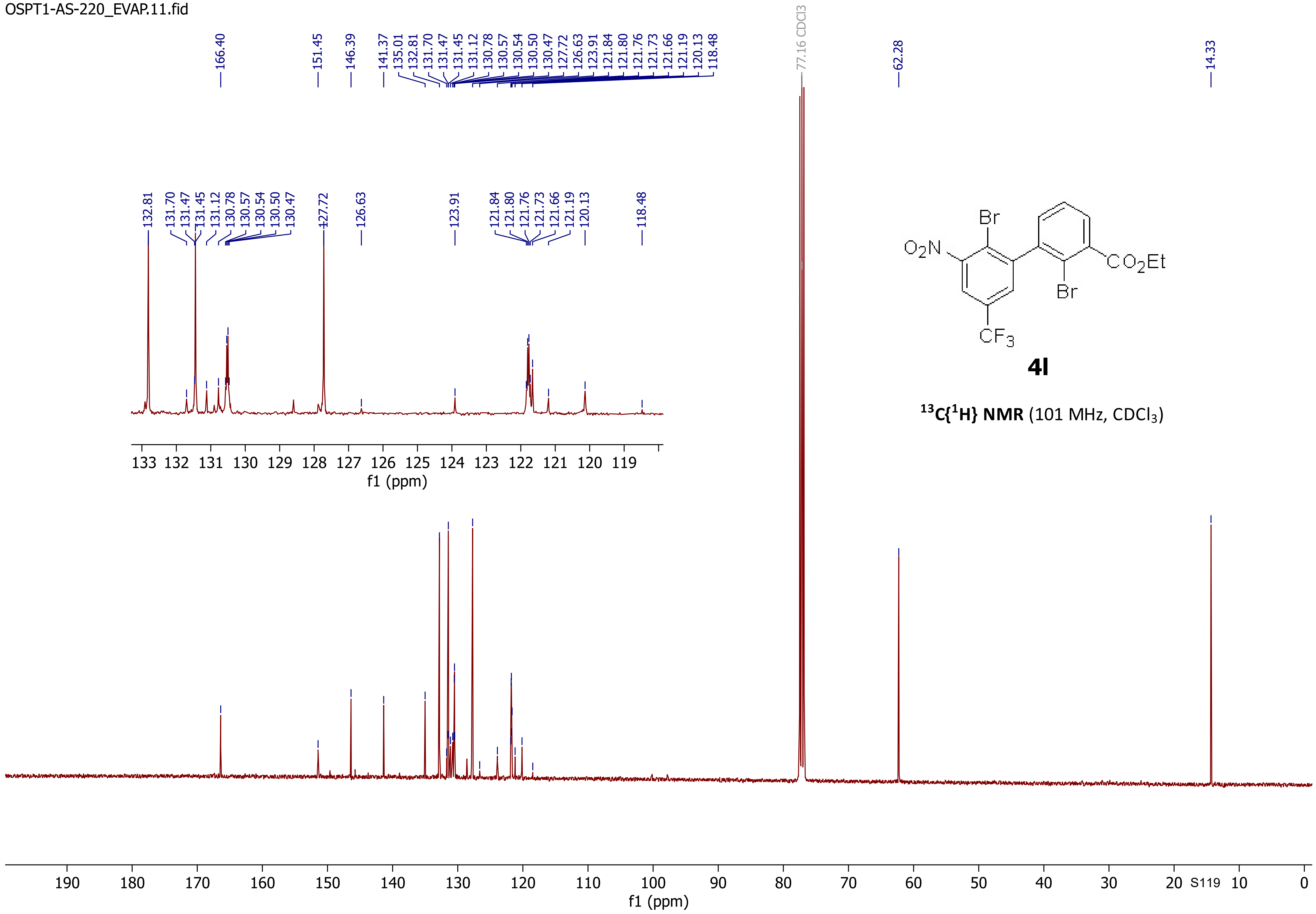
OSPT1-AS-220_EVAP.11.fid
166.40
151.45
146.39
141.37
135.01
132.81
131.70
131.47
131.45
131.12
130.78
130.57
130.54
130.50
130.47
127.72
126.63
123.91
121.84
121.80
121.76
121.73
121.66
121.19
120.13
118.48
77.16 CDCl3
62.28
14.33
133 132 131 130 129 128 127 126 125 124 123 122 121 120 119
f1 (ppm)
Br
O_2N
CO_2Et
Br
CF_3
4l
$^{13}C\{^1H\}$ NMR (101 MHz, $CDCl_3$)
190 180 170 160 150 140 130 120 110 100 90 80 70 60 50 40 30 20 10 0
f1 (ppm)

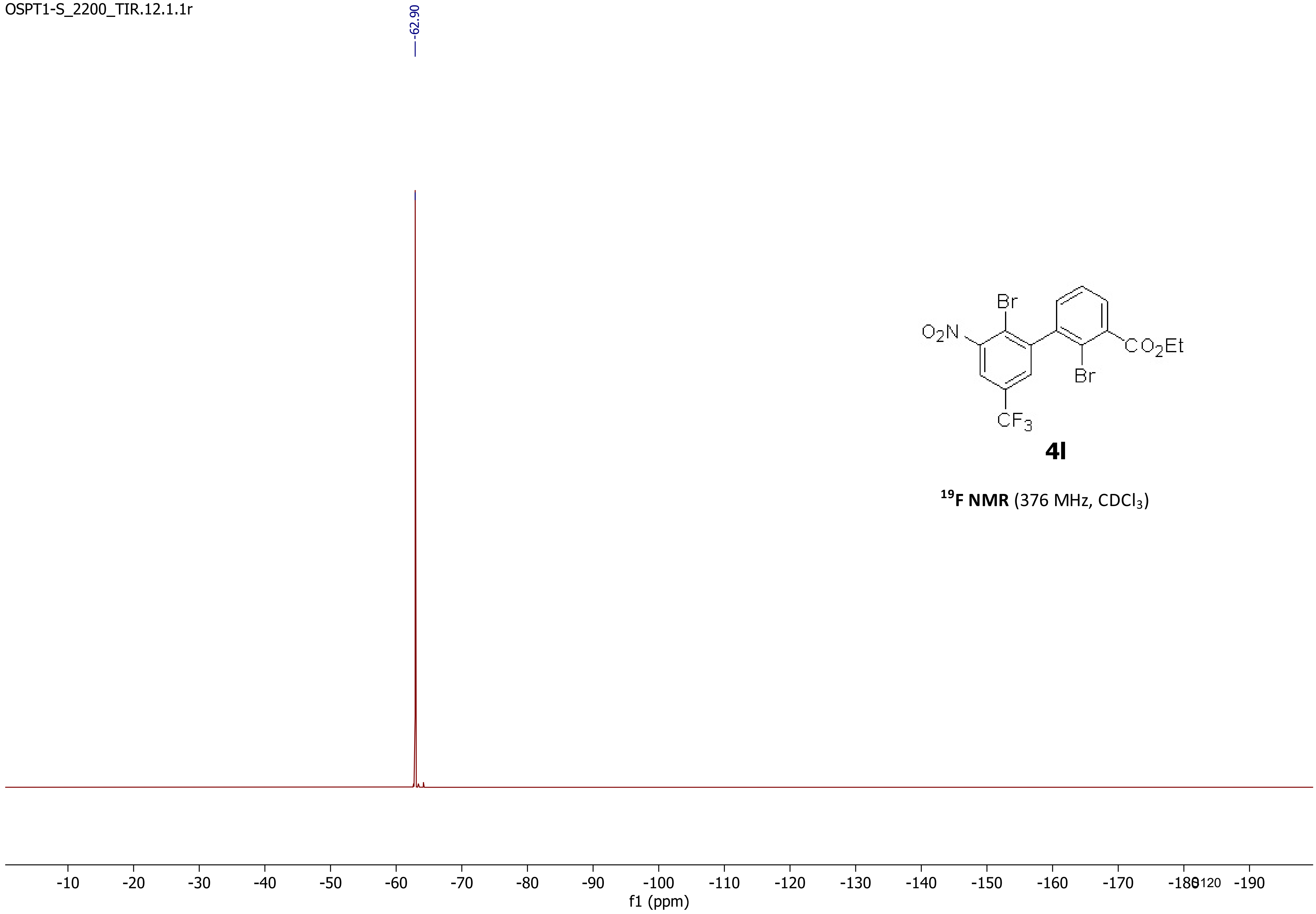

4l

^{19}F NMR (376 MHz, $CDCl_3$)

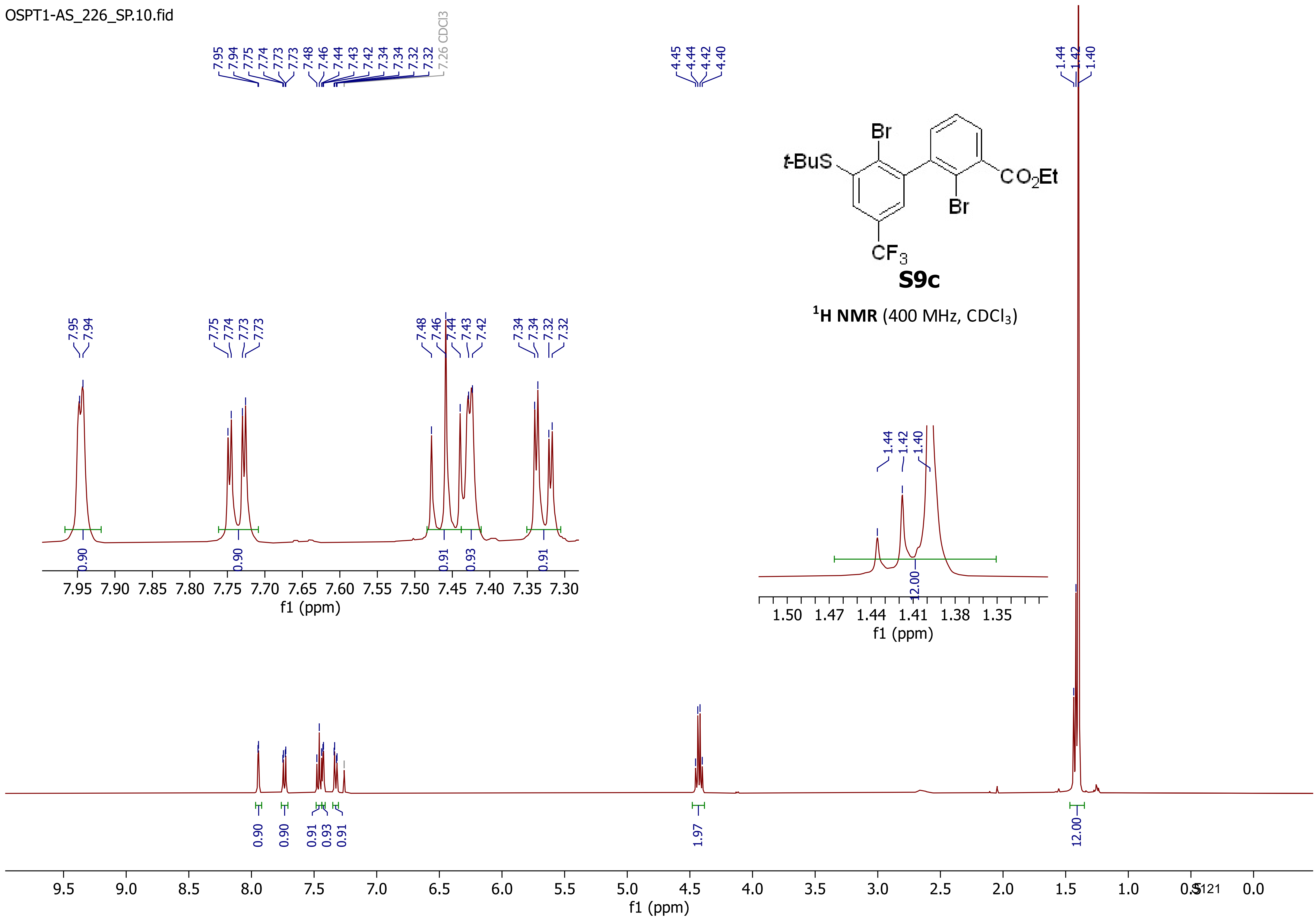
OSPT1-AS_226_SP.10.fid
7.95 7.94 7.75 7.74 7.73 7.73 7.48 7.46 7.44 7.43 7.42 7.34 7.34 7.32 7.32 7.26 CDCl3
4.45 4.44 4.42 4.40
1.44 1.42 1.40
Br
t-BuS
CO2Et
Br
CF3
S9c
1H NMR (400 MHz, CDCl3)
0.90 0.90 0.91 0.93 0.91
12.00
f1 (ppm)
0.90 0.90 0.91 0.93 0.91 1.97 12.00
f1 (ppm)

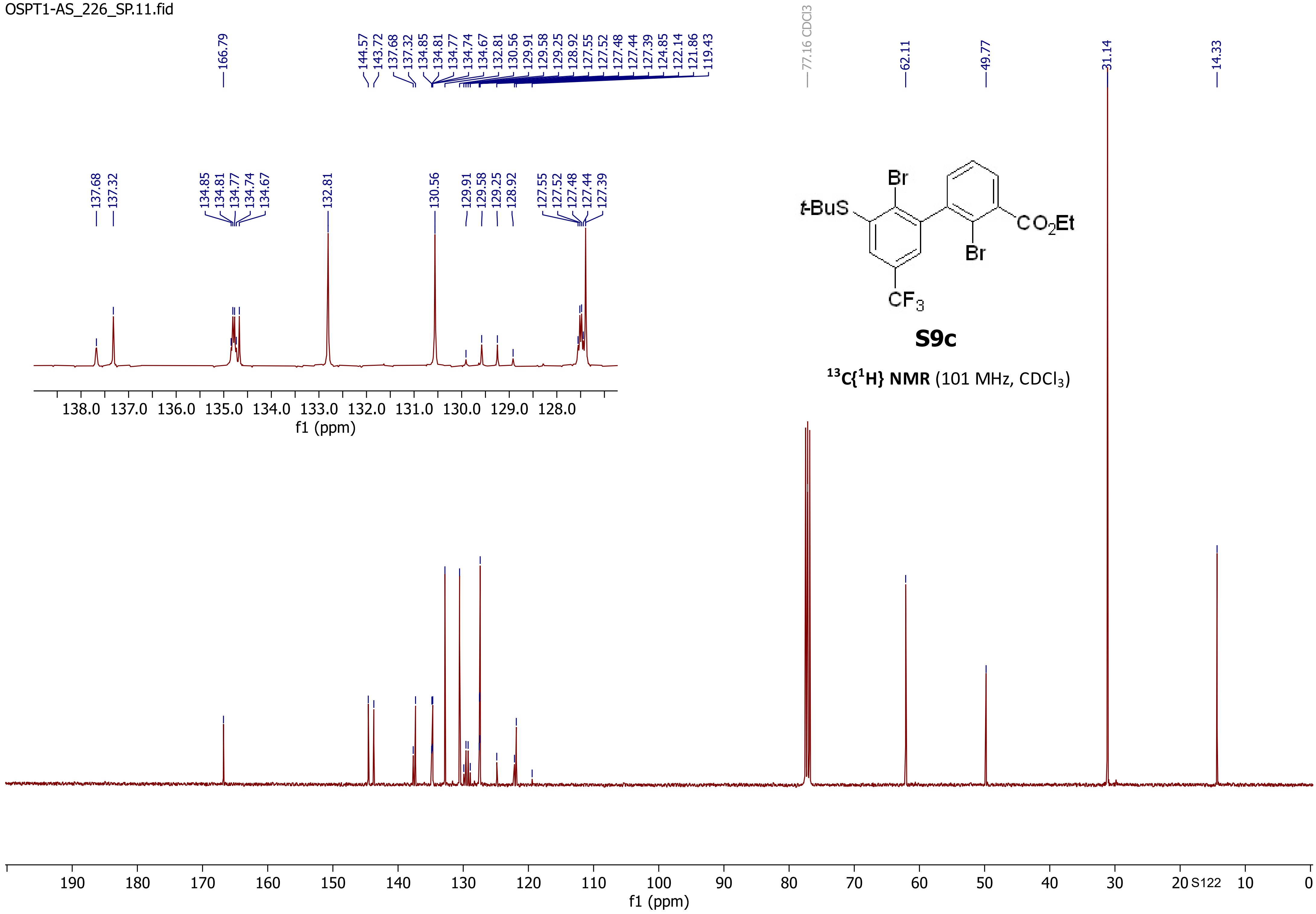

S9c

^{13}C{^{1}H} NMR (101 MHz, $CDCl_3$)

OSPT1-AS_226_SP.12.1.1r

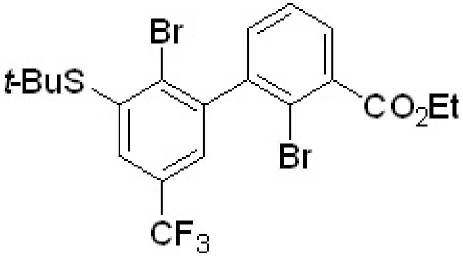

S9c

^{19}F NMR (376 MHz, $CDCl_3$)

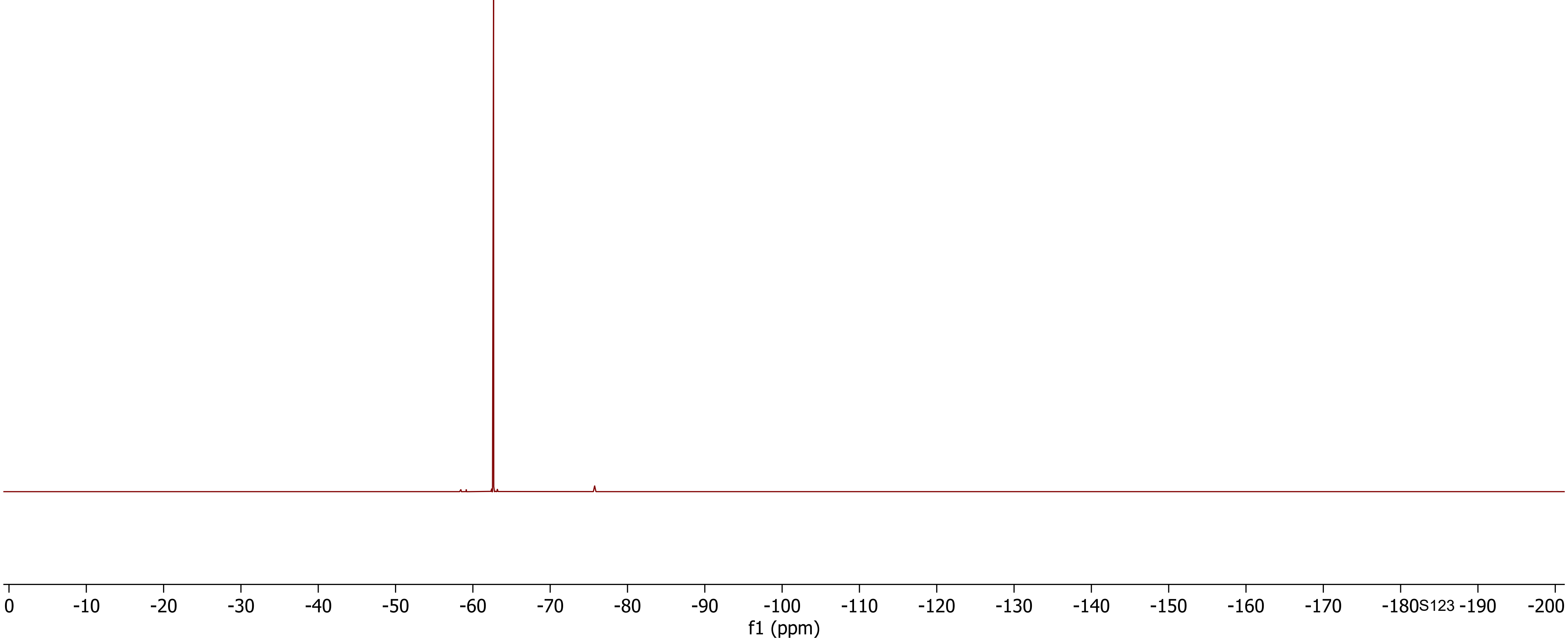

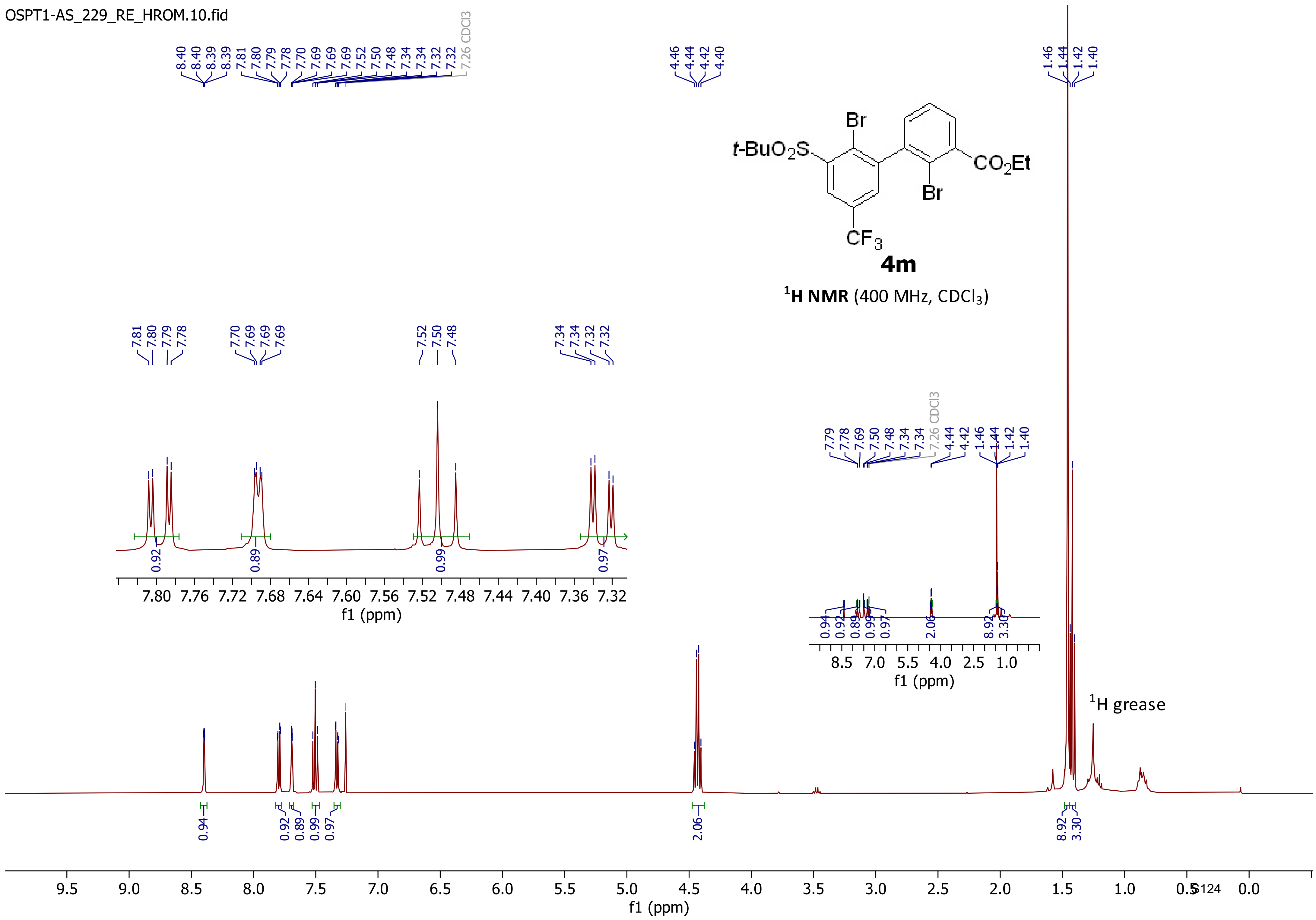
OSPT1-AS_229_RE_HROM.10.fid
8.40 8.40 8.39 8.39 7.81 7.80 7.79 7.78 7.70 7.69 7.69 7.69 7.52 7.50 7.48 7.34 7.34 7.32 7.32 7.26 CDCl3
4.46 4.44 4.42 4.40
1.46 1.44 1.42 1.40
Br
t-BuO2S
CO2Et
Br
CF3
4m
1H NMR (400 MHz, CDCl3)
1H grease
0.94 0.92 0.89 0.99 0.97 2.06 8.92 3.30
f1 (ppm)

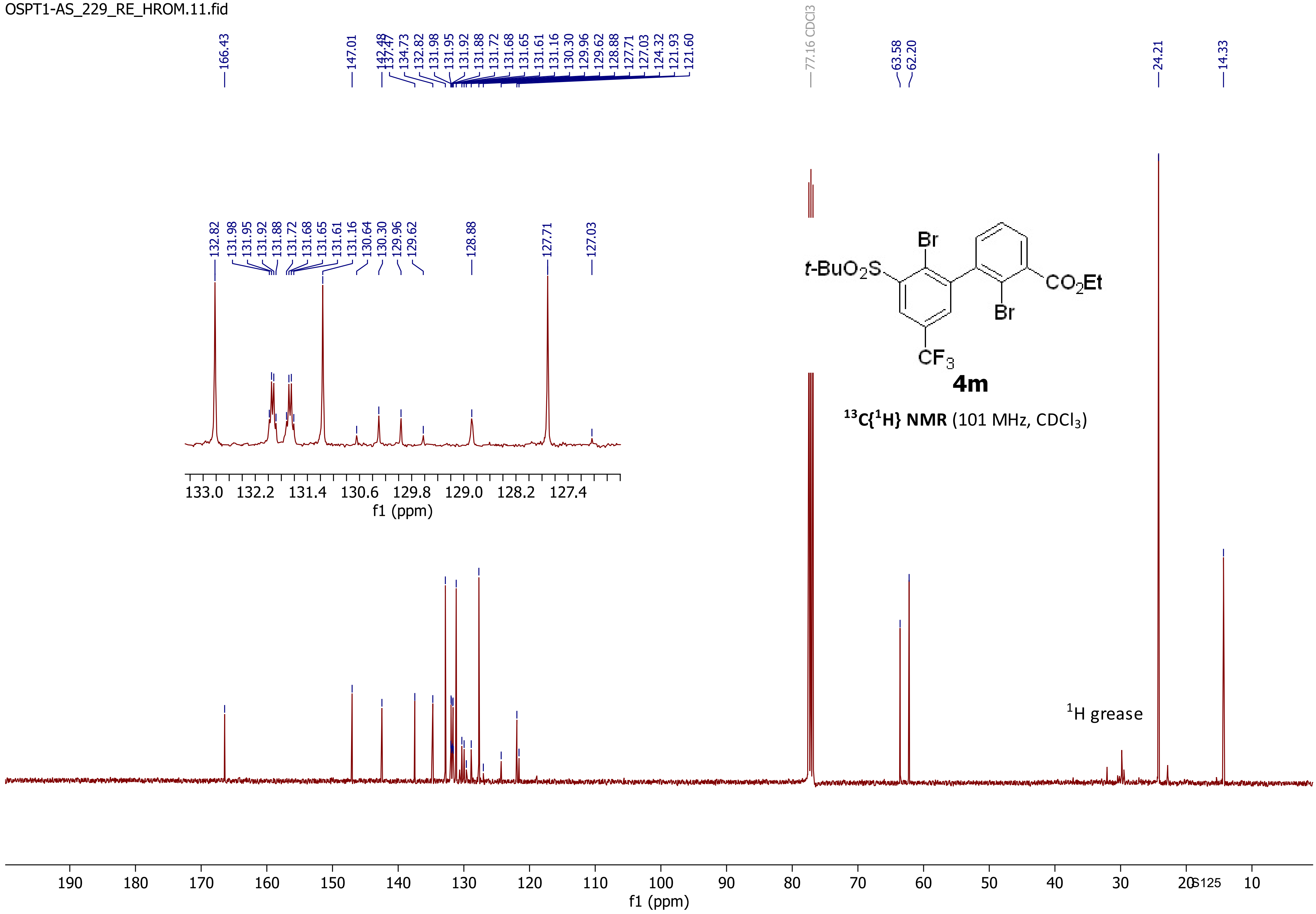
OSPT1-AS_229_RE_HROM.11.fid
166.43
147.01
142.48
137.47
134.73
132.82
131.98
131.95
131.92
131.88
131.72
131.68
131.65
131.61
131.16
130.30
129.96
129.62
128.88
127.71
127.03
124.32
121.93
121.60
77.16 CDCl3
63.58
62.20
24.21
14.33
130.64
t-BuO2S
Br
Br
CO2Et
CF3
4m
13C{1H} NMR (101 MHz, CDCl3)
1H grease
f1 (ppm)

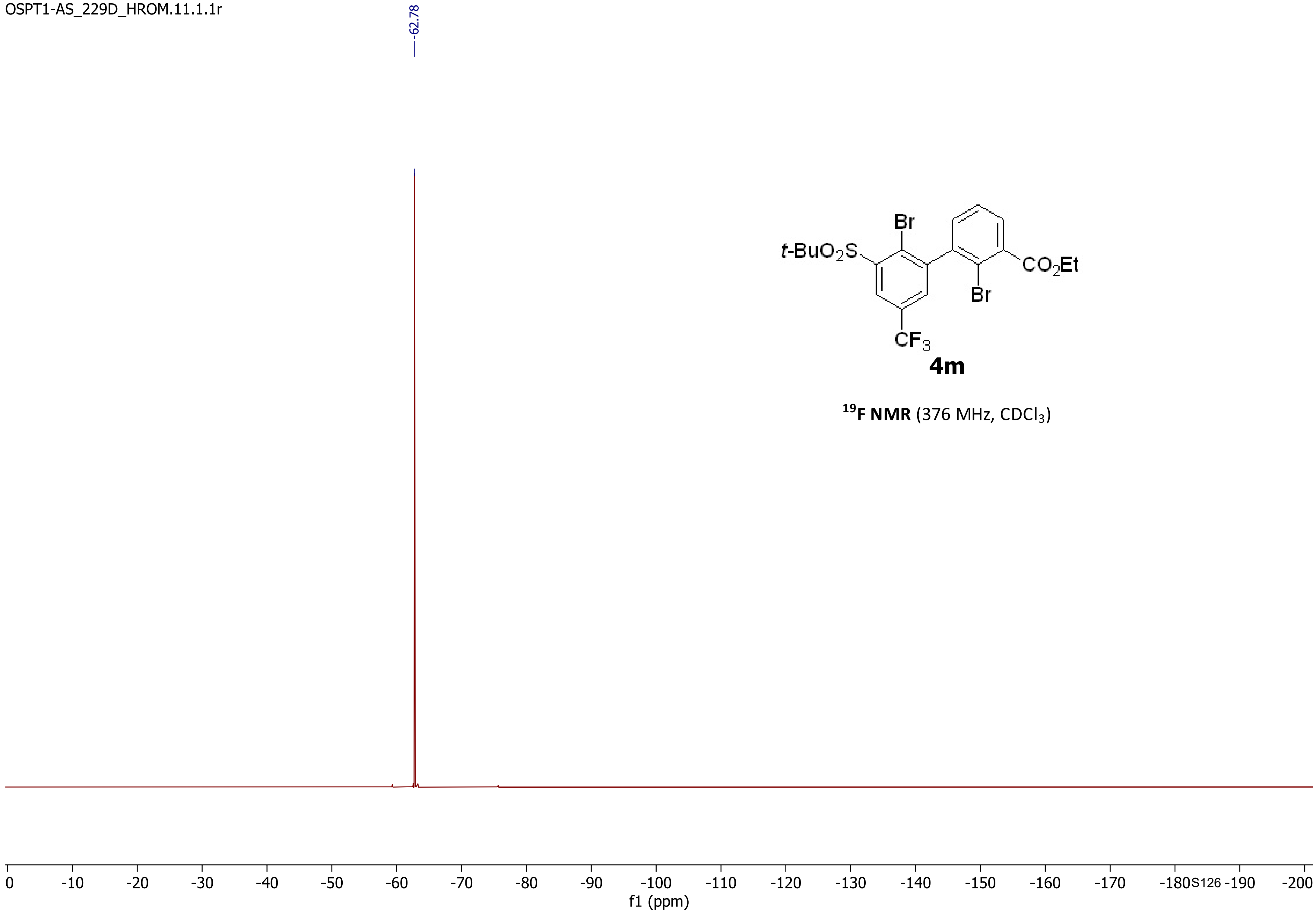

4m

^{19}F NMR (376 MHz, $CDCl_3$)

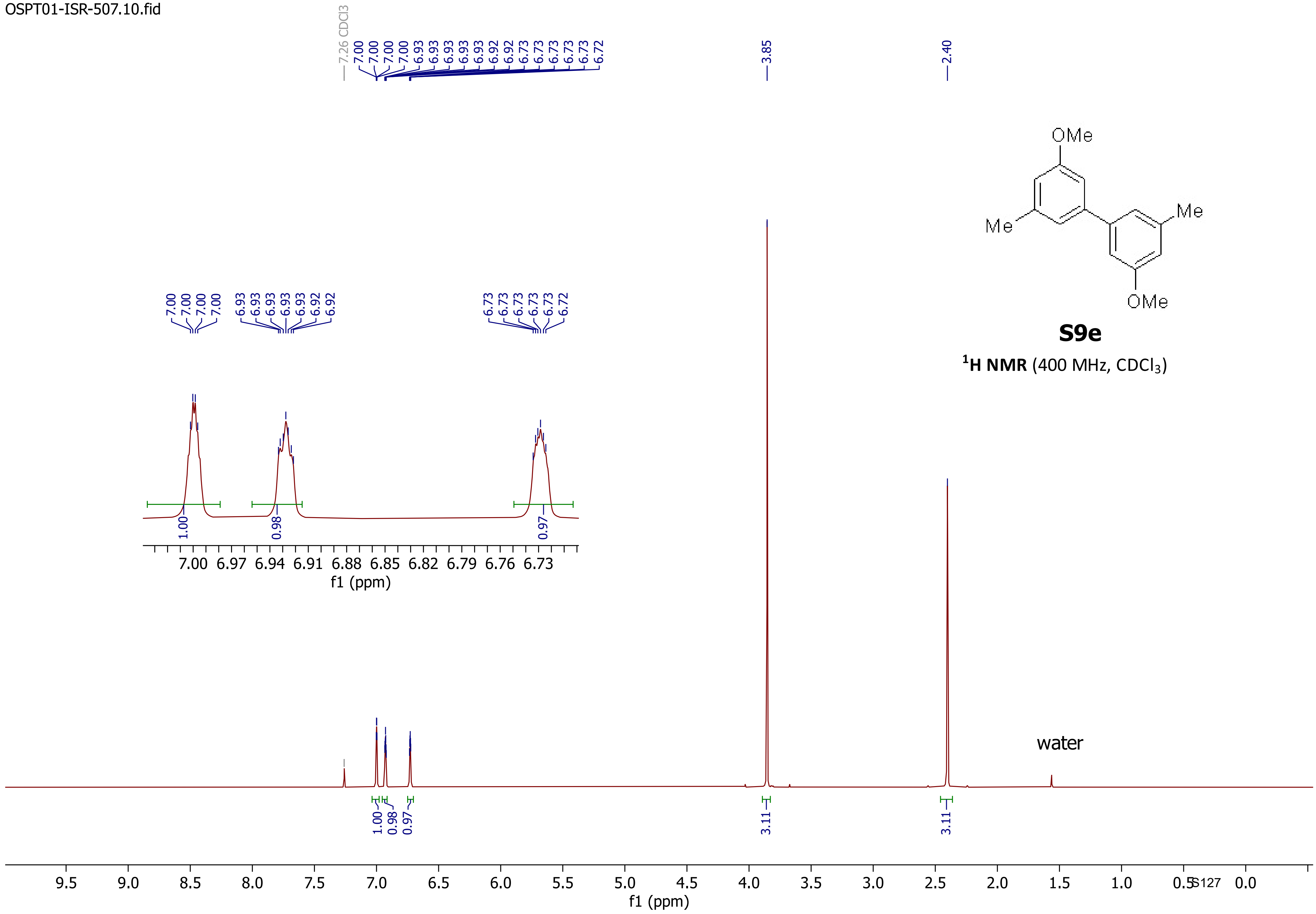

OSPT01-ISR-507.10.fid
7.26 CDCl3
7.00
7.00
7.00
7.00
6.93
6.93
6.93
6.93
6.93
6.92
6.92
6.73
6.73
6.73
6.73
6.73
6.72
3.85
2.40
OMe
Me
Me
OMe
S9e
1H NMR (400 MHz, CDCl3)
1.00
0.98
0.97
7.00 6.97 6.94 6.91 6.88 6.85 6.82 6.79 6.76 6.73
f1 (ppm)
water
3.11
3.11
9.5 9.0 8.5 8.0 7.5 7.0 6.5 6.0 5.5 5.0 4.5 4.0 3.5 3.0 2.5 2.0 1.5 1.0 0.5 0.0
f1 (ppm)

OSPT01-ISR-507.11.fid

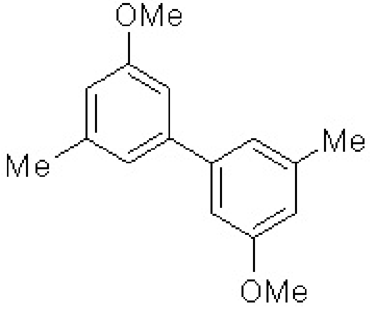

S9e

^{13}C{^{1}H} NMR (101 MHz, $CDCl_3$)

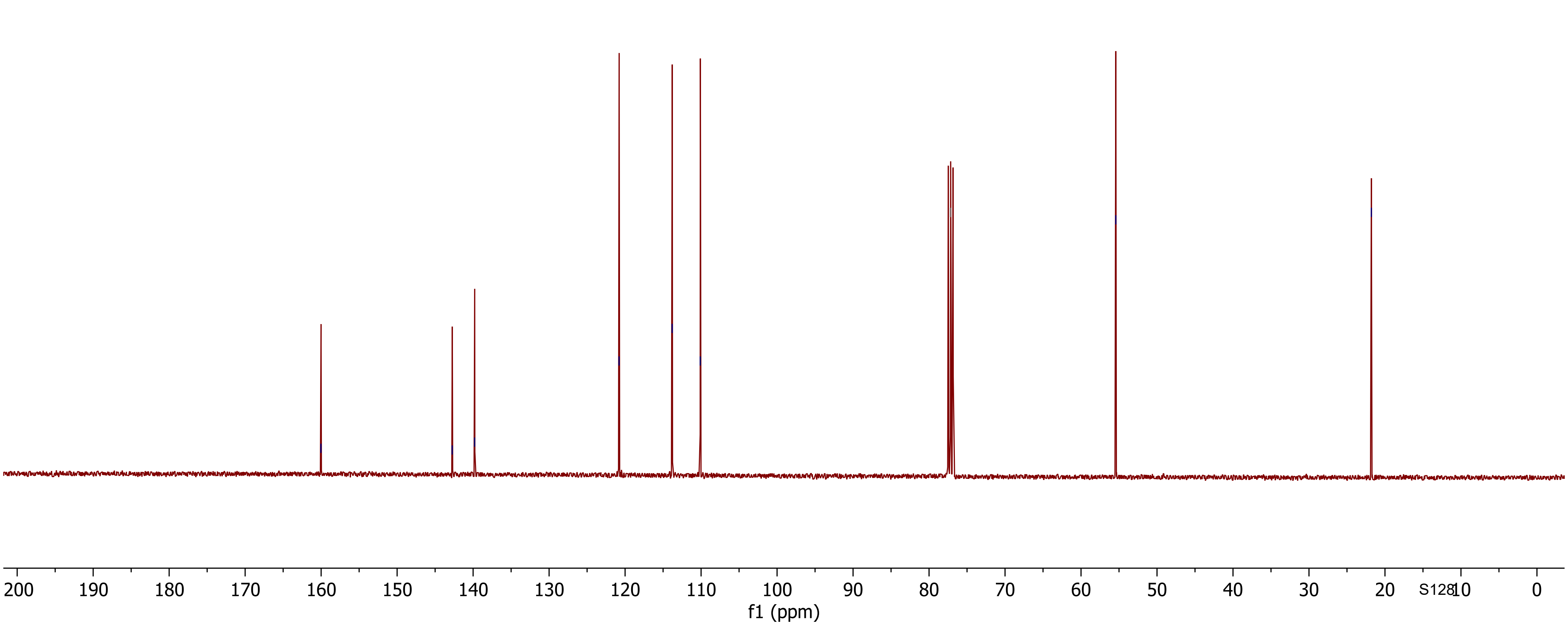

OSPT01-ISR-513.10.fid

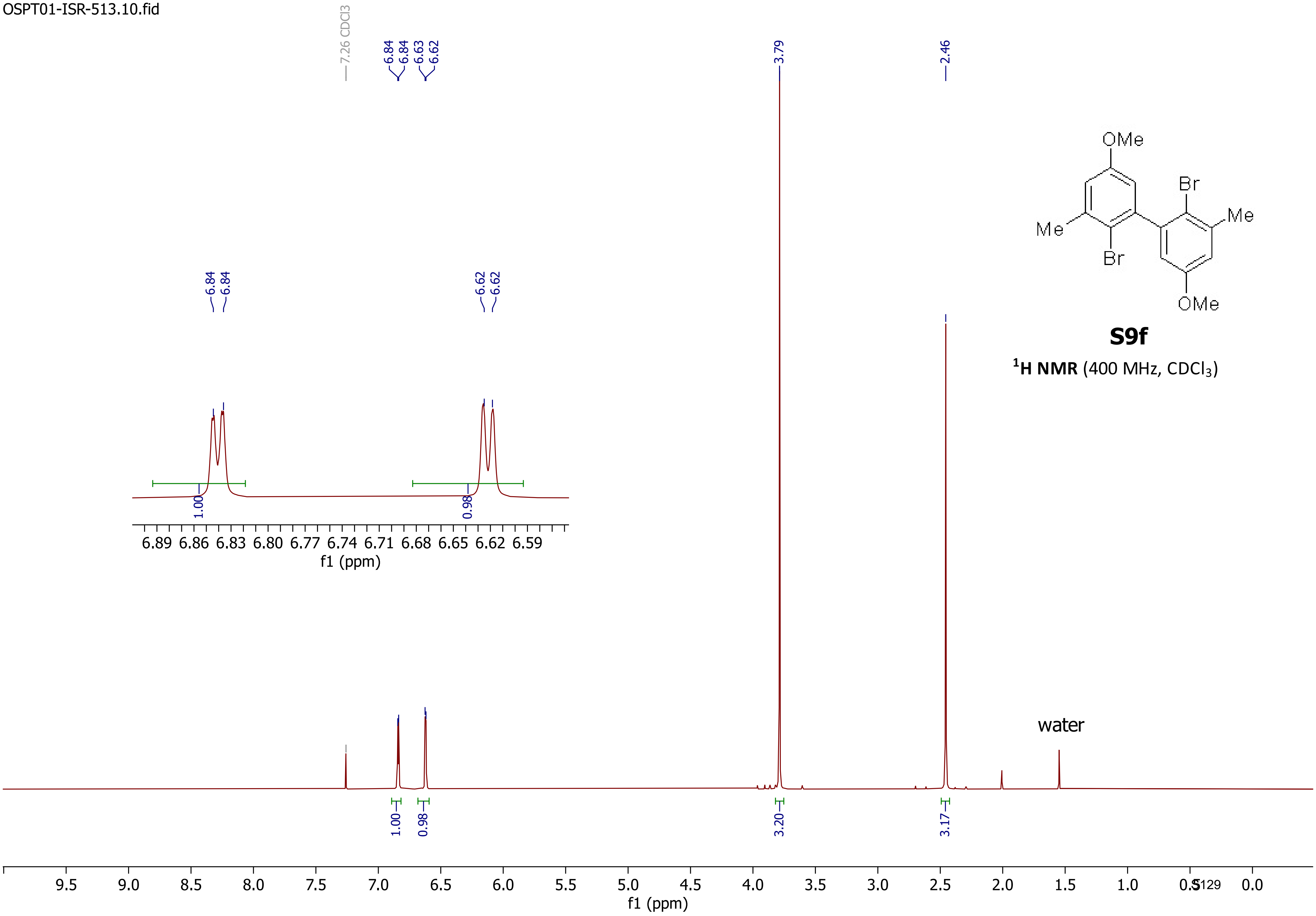

S9f

^{1}H NMR (400 MHz, $CDCl_3$)

OSPT01-ISR-513.11.fid

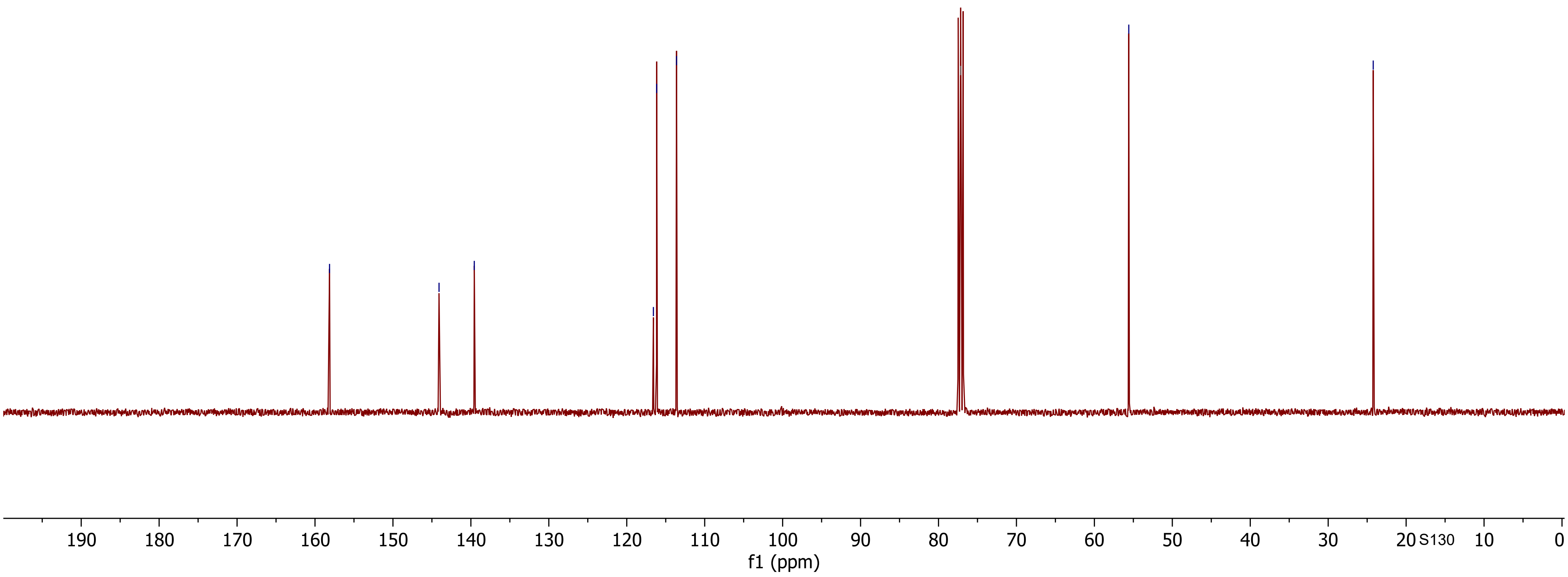

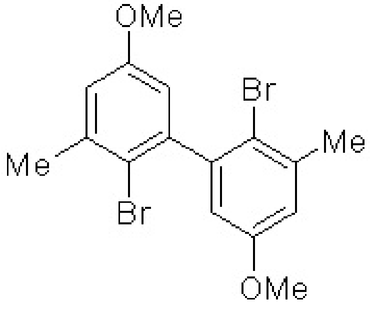

S9f

$^{13}C\{^{1}H\}$ NMR (101 MHz, $CDCl_3$)

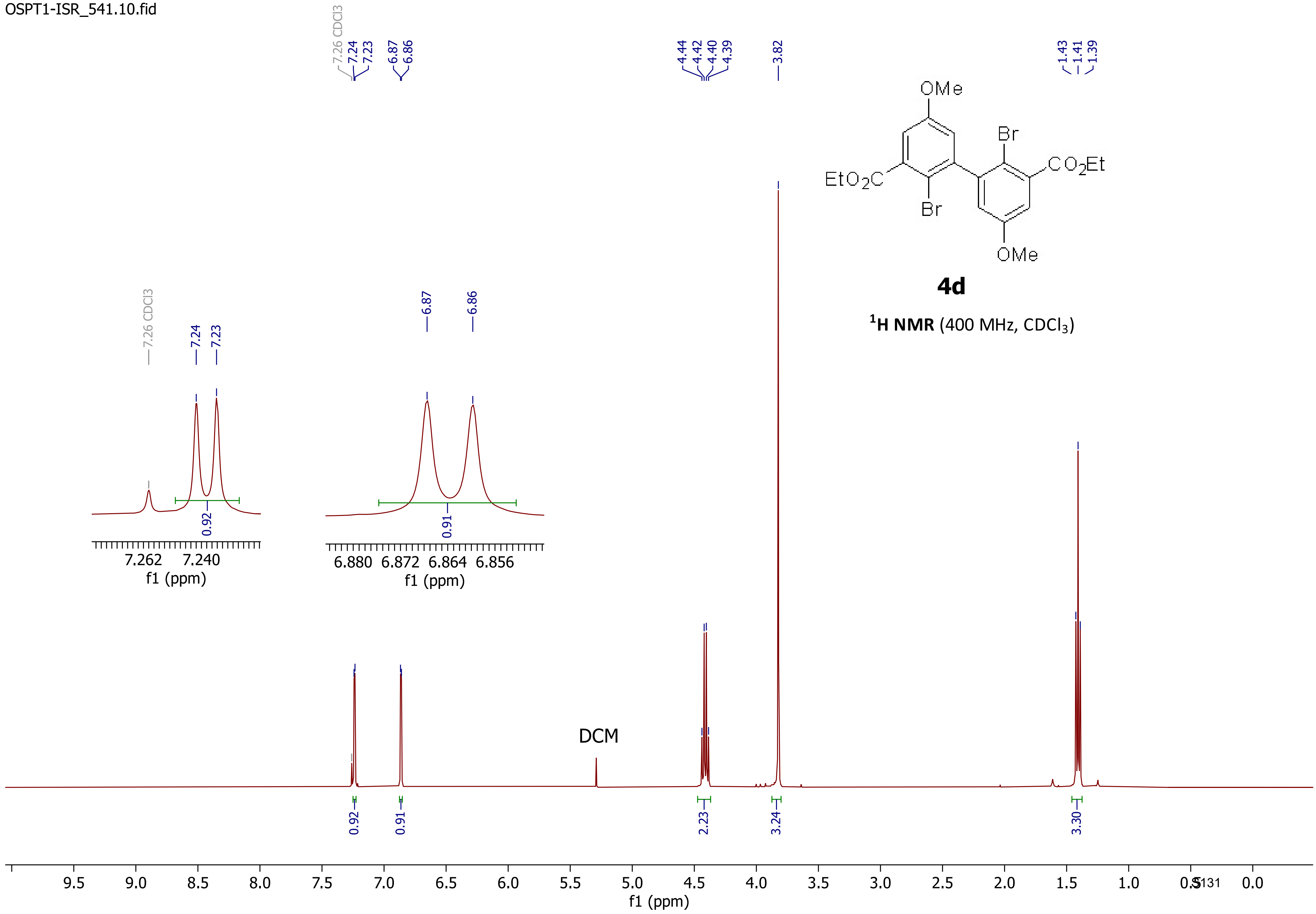
OSPT1-ISR_541.10.fid
7.26 CDCl3
7.24
7.23
6.87
6.86
4.44
4.42
4.40
4.39
3.82
1.43
1.41
1.39
OMe
Br
CO_2Et
EtO_2C
Br
OMe
4d
¹H NMR (400 MHz, $CDCl_3$)
0.92
7.262
7.240
f1 (ppm)
0.91
6.880
6.872
6.864
6.856
f1 (ppm)
DCM
0.92
0.91
2.23
3.24
3.30
9.5
9.0
8.5
8.0
7.5
7.0
6.5
6.0
5.5
5.0
4.5
4.0
3.5
3.0
2.5
2.0
1.5
1.0
0.5
0.0
f1 (ppm)

OSPT1-ISR_541.11.fid

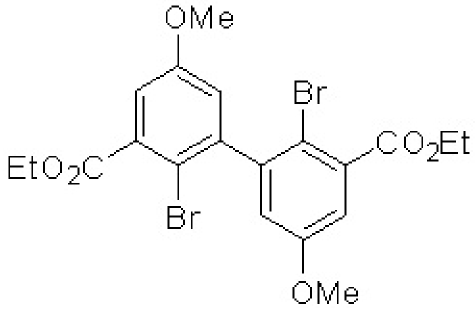

4d

$^{13}C\{^{1}H\}$ NMR (101 MHz, $CDCl_3$)

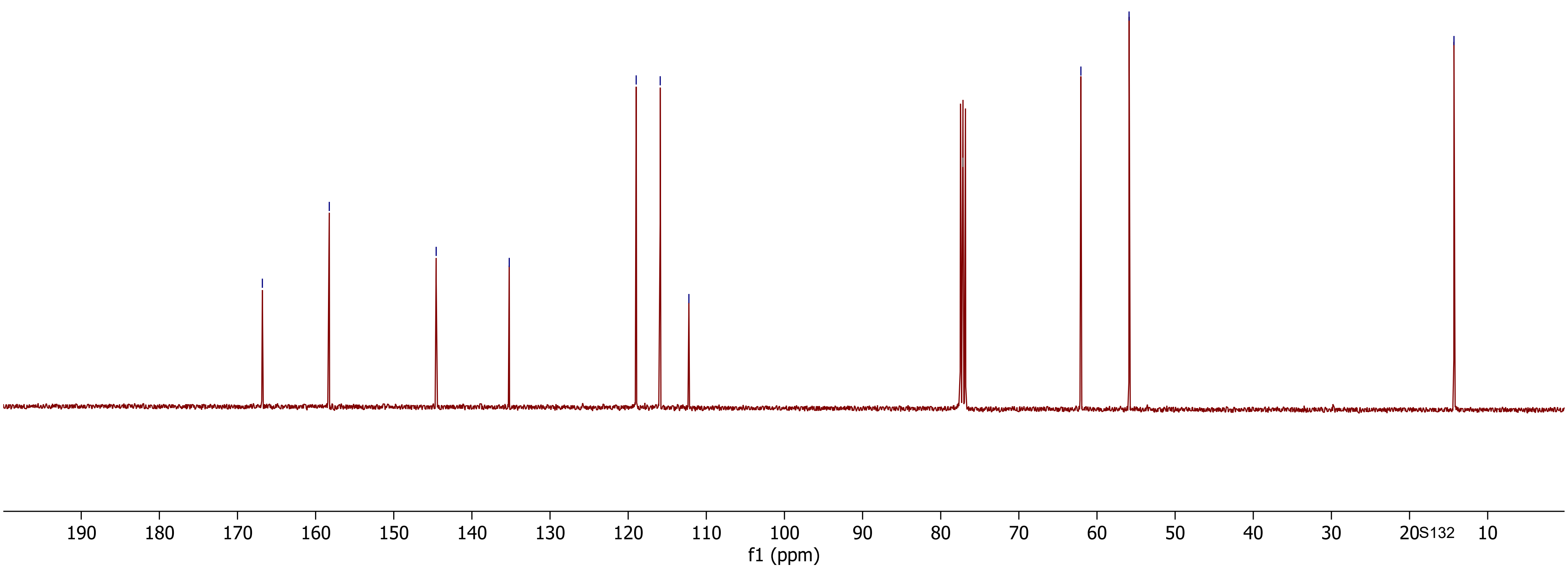

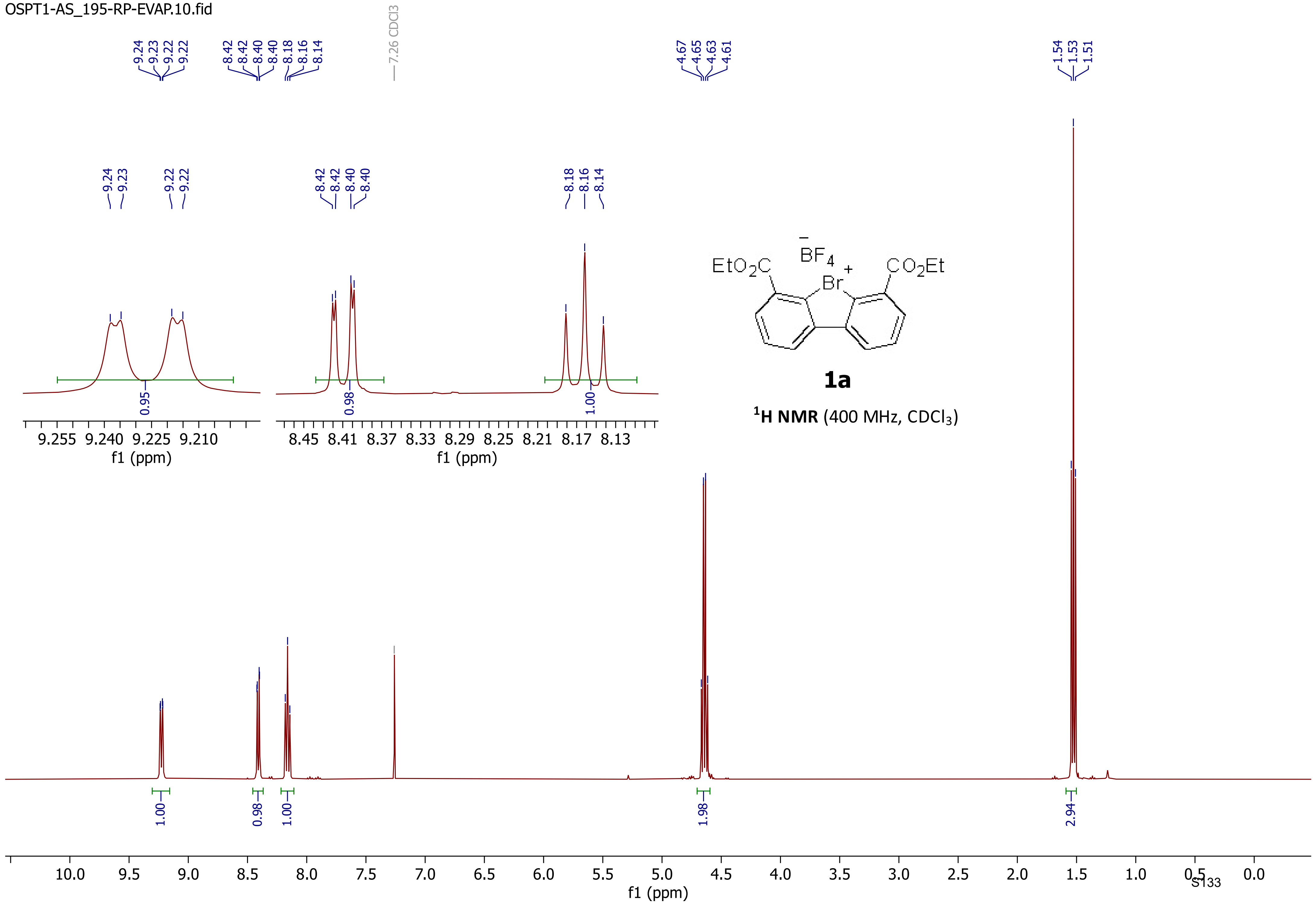
OSPT1-AS_195-RP-EVAP.10.fid
9.24
9.23
9.22
9.22
8.42
8.42
8.40
8.40
8.18
8.16
8.14
7.26 CDCl3
4.67
4.65
4.63
4.61
1.54
1.53
1.51
0.95
0.98
1.00
9.255 9.240 9.225 9.210
f1 (ppm)
8.45 8.41 8.37 8.33 8.29 8.25 8.21 8.17 8.13
f1 (ppm)
EtO2C
BF4
Br
CO2Et
1a
1H NMR (400 MHz, CDCl3)
1.00
0.98
1.00
1.98
2.94
10.0 9.5 9.0 8.5 8.0 7.5 7.0 6.5 6.0 5.5 5.0 4.5 4.0 3.5 3.0 2.5 2.0 1.5 1.0 0.5 0.0
f1 (ppm)

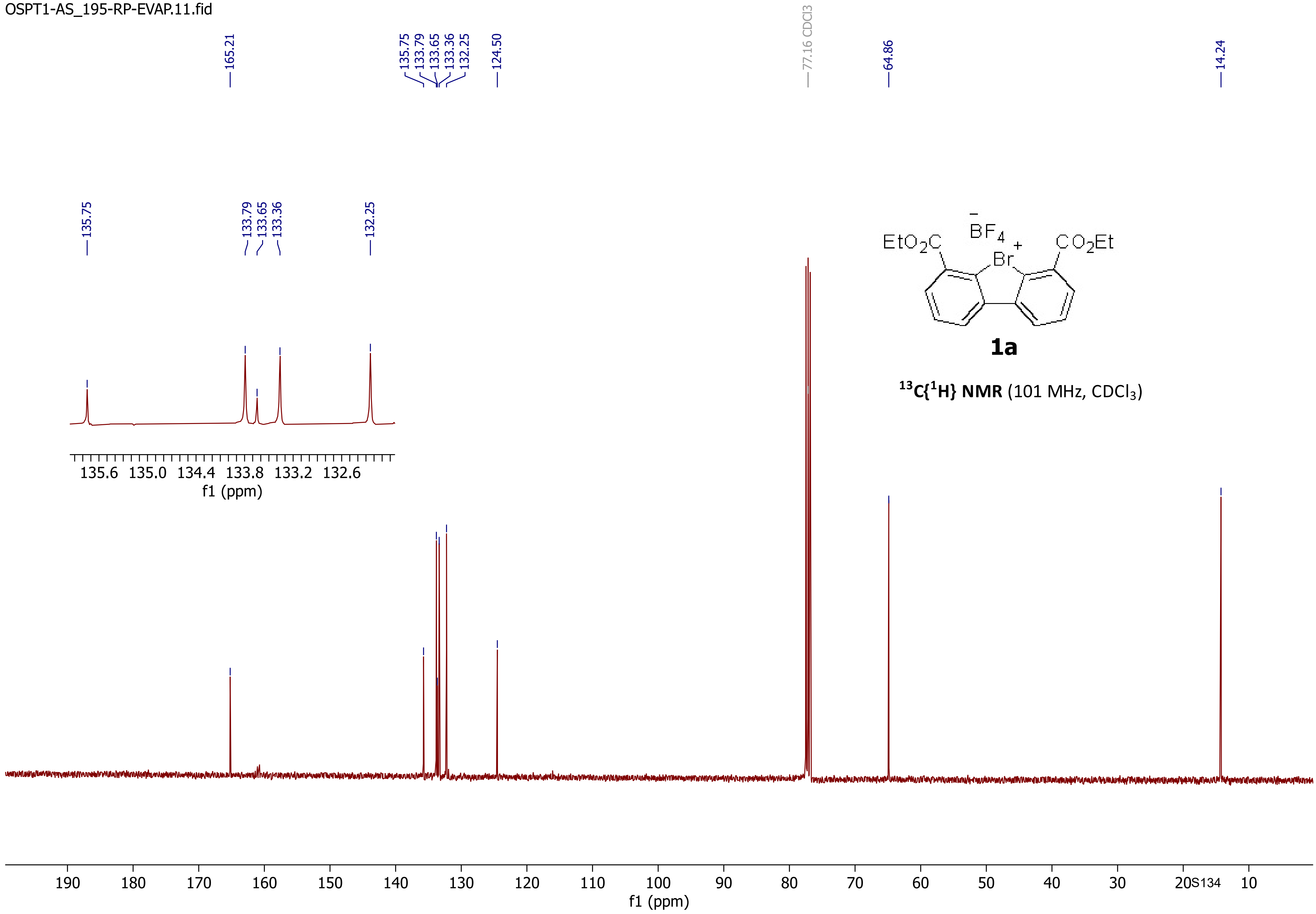
OSPT1-AS_195-RP-EVAP.11.fid
165.21
135.75
133.79
133.65
133.36
132.25
124.50
77.16 CDCl3
64.86
14.24
135.6 135.0 134.4 133.8 133.2 132.6
f1 (ppm)
EtO2C
BF4
Br
CO2Et
1a
13C{1H} NMR (101 MHz, CDCl3)
190 180 170 160 150 140 130 120 110 100 90 80 70 60 50 40 30 20 10
f1 (ppm)

OSPT1-AS_252_RE4.11.1.1r

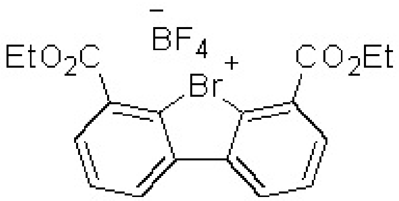

1a

^{19}F NMR (376 MHz, $CDCl_3$)

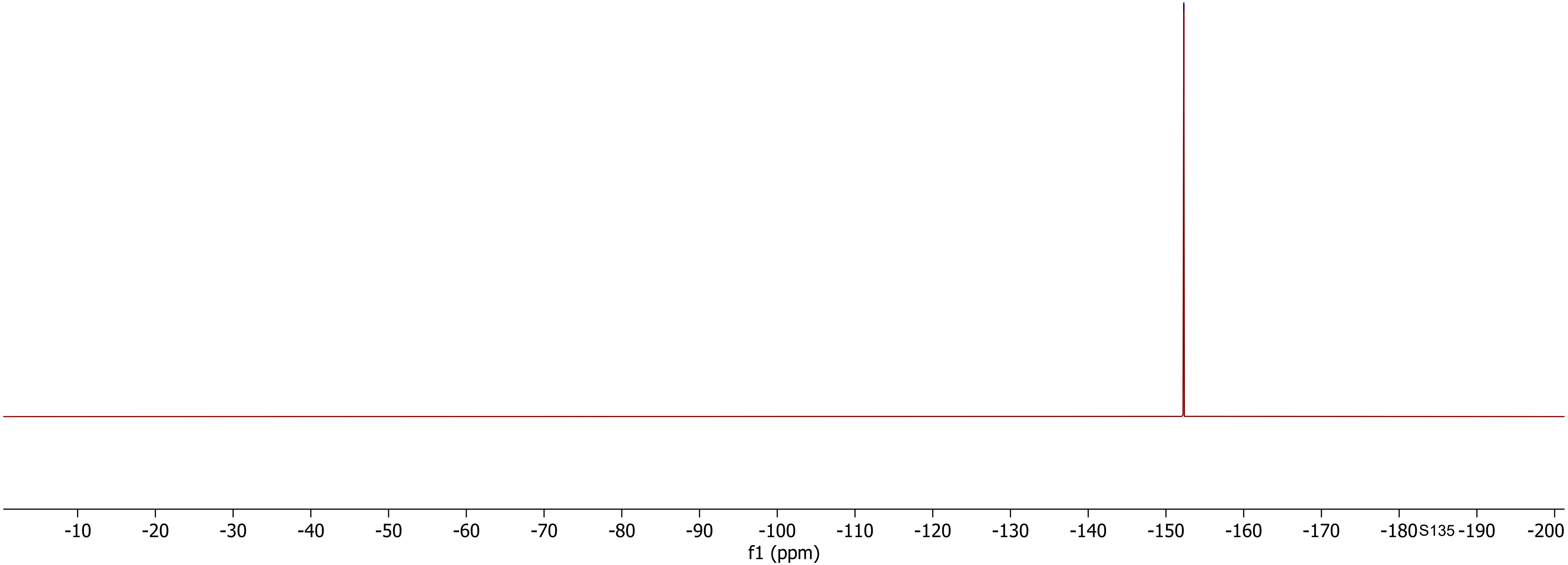

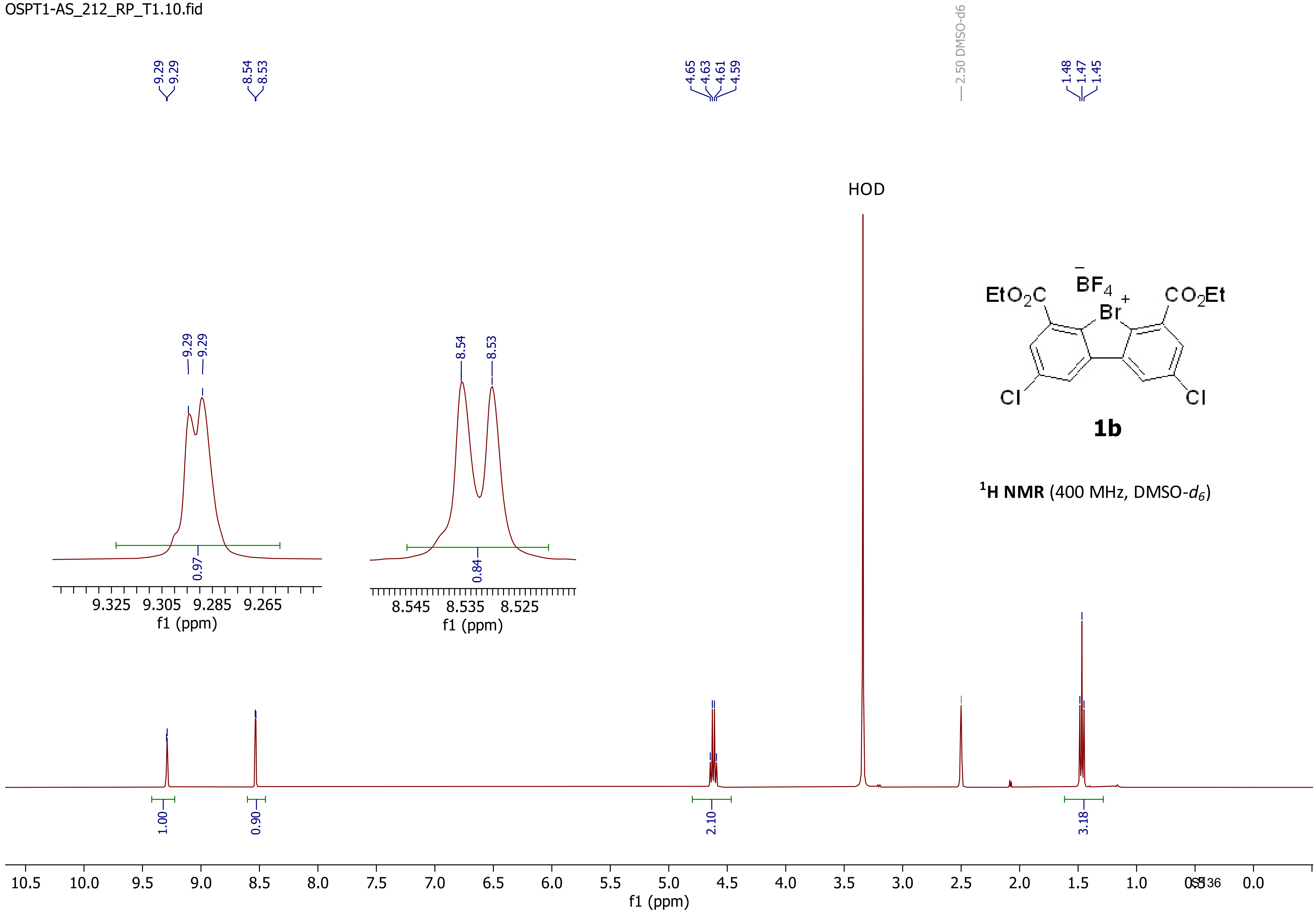
OSPT1-AS_212_RP_T1.10.fid
9.29
9.29
8.54
8.53
4.65
4.63
4.61
4.59
2.50 DMSO-d6
1.48
1.47
1.45
HOD
0.97
0.84
9.325 9.305 9.285 9.265
8.545 8.535 8.525
f1 (ppm)
EtO2C
BF4
Br+
CO2Et
Cl
Cl
1b
1H NMR (400 MHz, DMSO-d6)
1.00
0.90
2.10
3.18
10.5 10.0 9.5 9.0 8.5 8.0 7.5 7.0 6.5 6.0 5.5 5.0 4.5 4.0 3.5 3.0 2.5 2.0 1.5 1.0 0.5 0.0
f1 (ppm)

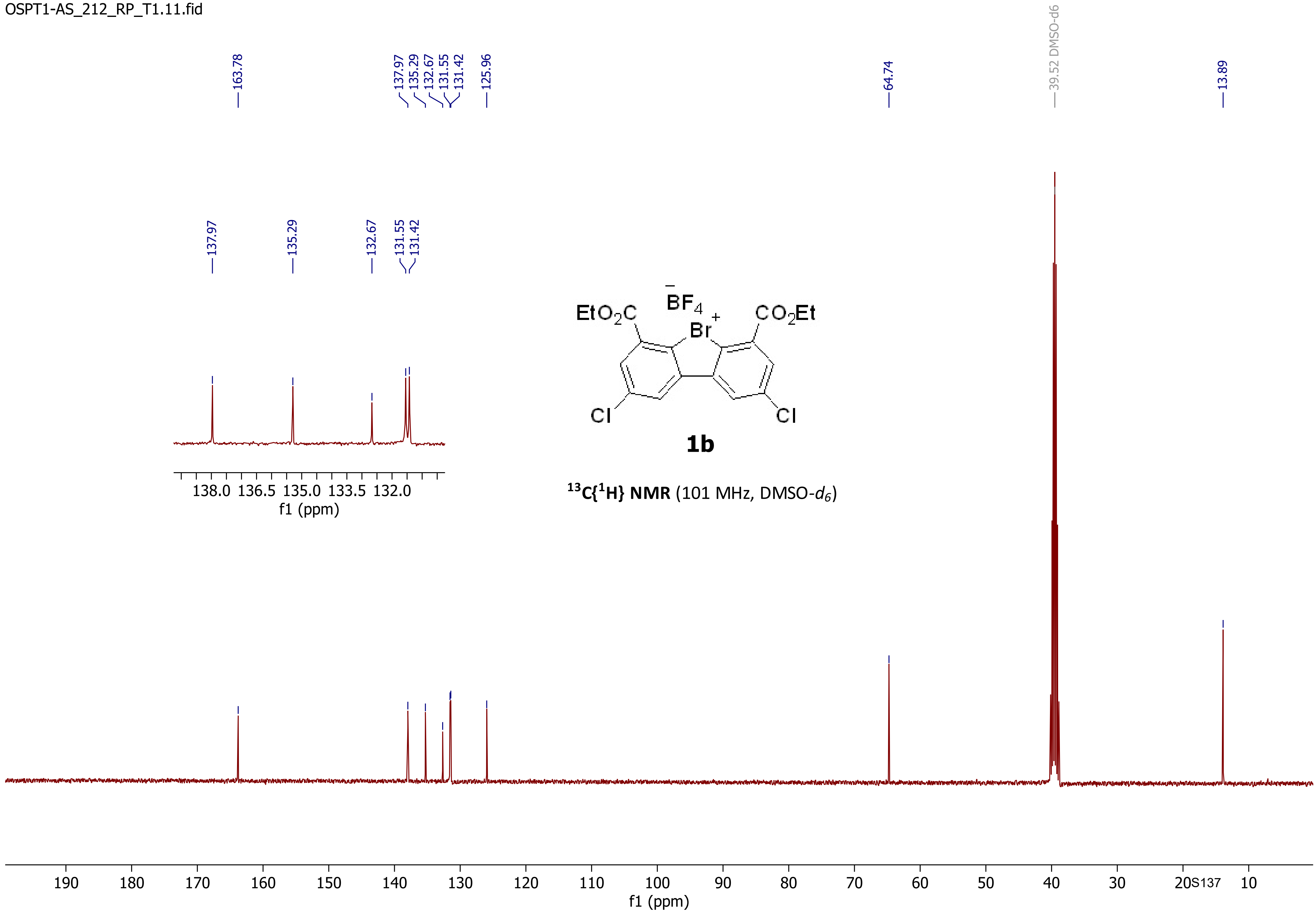
OSPT1-AS_212_RP_T1.11.fid
163.78
137.97
135.29
132.67
131.55
131.42
125.96
64.74
39.52 DMSO-d6
13.89
138.0 136.5 135.0 133.5 132.0
f1 (ppm)
EtO2C
BF4
Br
CO2Et
Cl
Cl
1b
13C{1H} NMR (101 MHz, DMSO-d6)
190 180 170 160 150 140 130 120 110 100 90 80 70 60 50 40 30 20 10
f1 (ppm)

OSPT1-AS_212_RP_T1.12.1.1r

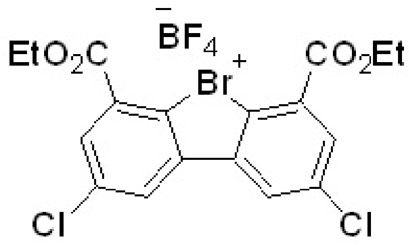

1b

^{19}F NMR (376 MHz, DMSO-*d*$_6$)

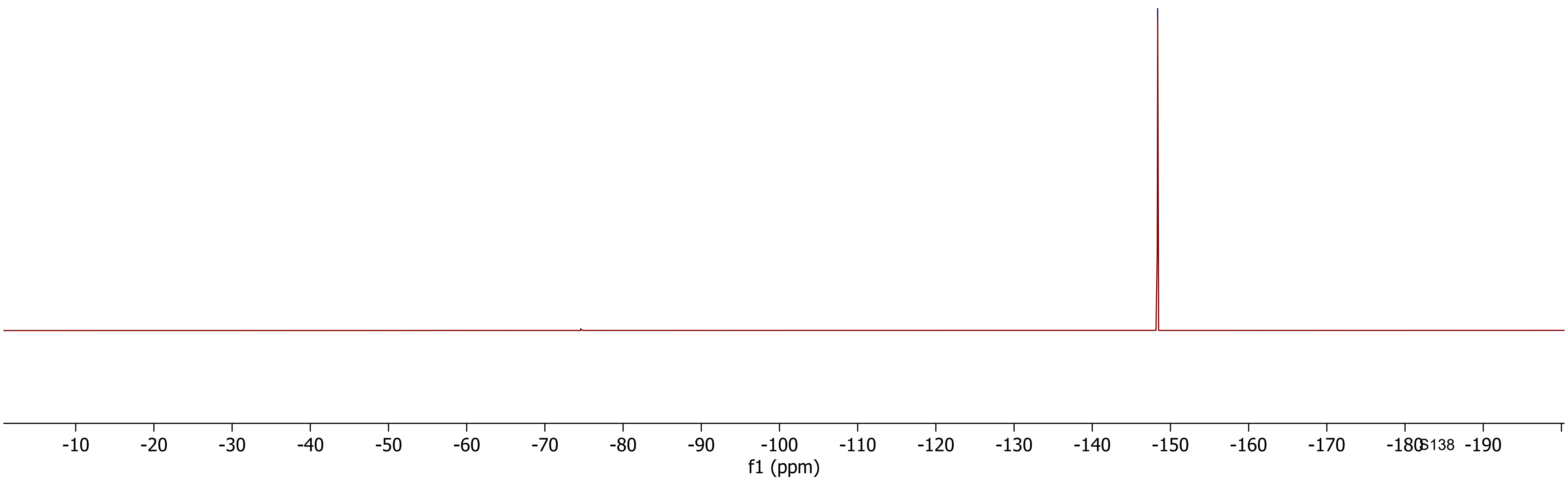

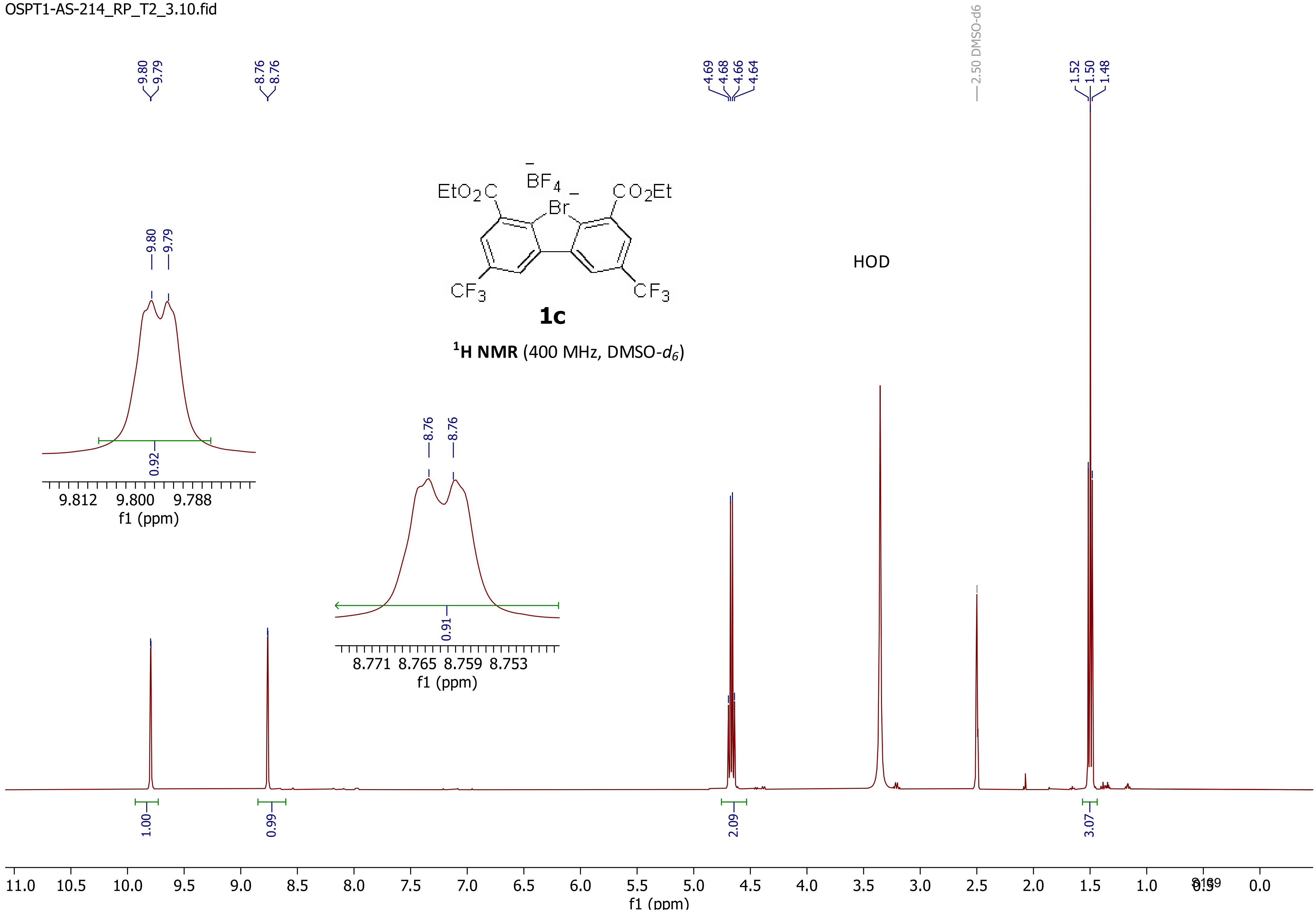
OSPT1-AS-214_RP_T2_3.10.fid
9.80
9.79
8.76
8.76
4.69
4.68
4.66
4.64
2.50 DMSO-d6
1.52
1.50
1.48
EtO2C
BF4
Br
CO2Et
CF3
CF3
1c
1H NMR (400 MHz, DMSO-d6)
HOD
0.92
9.812
9.800
9.788
f1 (ppm)
0.91
8.771
8.765
8.759
8.753
f1 (ppm)
1.00
0.99
2.09
3.07
11.0
10.5
10.0
9.5
9.0
8.5
8.0
7.5
7.0
6.5
6.0
5.5
5.0
4.5
4.0
3.5
3.0
2.5
2.0
1.5
1.0
0.0
f1 (ppm)

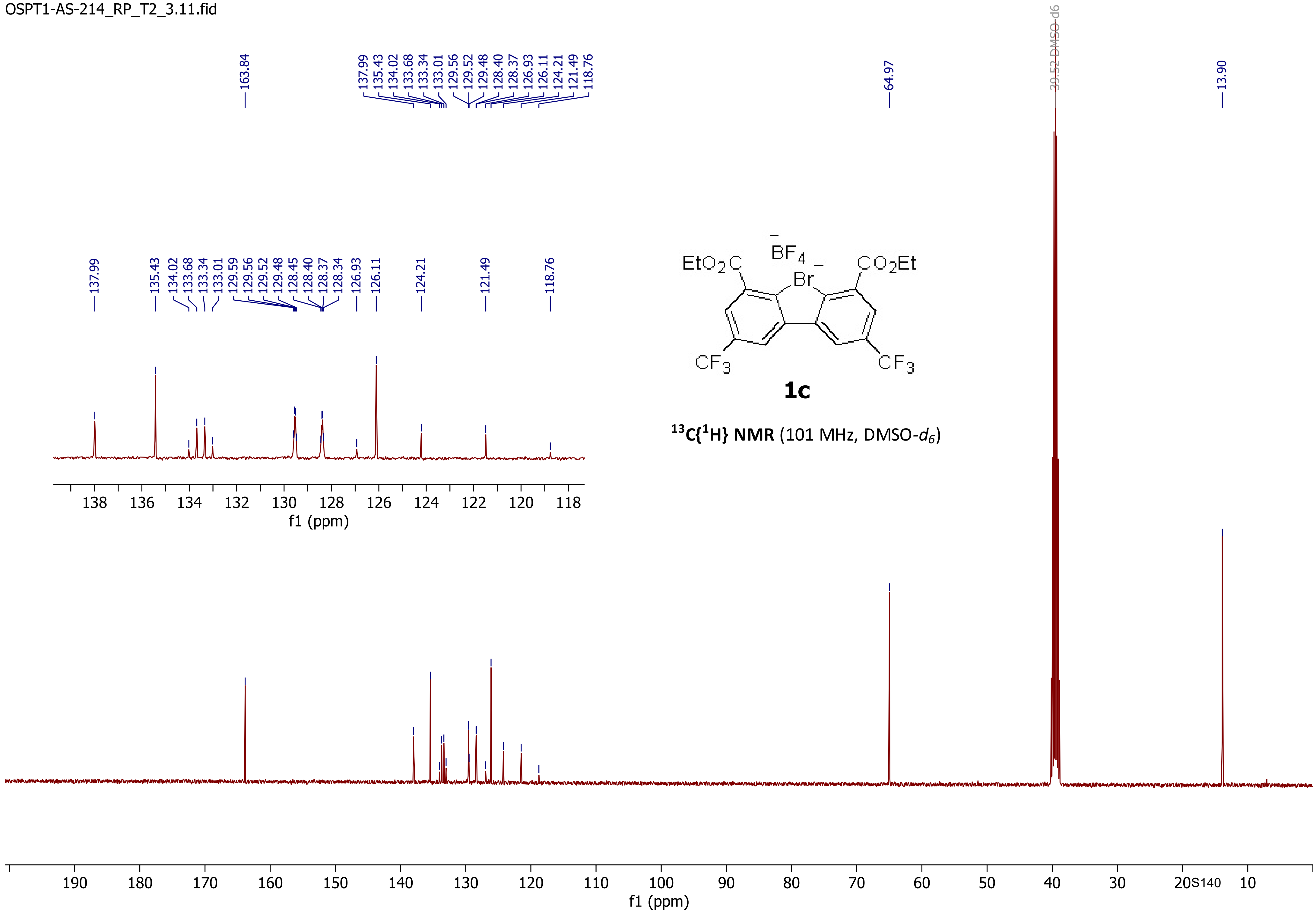
OSPT1-AS-214_RP_T2_3.11.fid
163.84
137.99
135.43
134.02
133.68
133.34
133.01
129.56
129.52
129.48
128.40
128.37
126.93
126.11
124.21
121.49
118.76
64.97
39.52 DMSO-d6
13.90
137.99
135.43
134.02
133.68
133.34
133.01
129.59
129.56
129.52
129.48
128.45
128.40
128.37
128.34
126.93
126.11
124.21
121.49
118.76
f1 (ppm)
EtO2C
BF4
Br
CO2Et
CF3
CF3
1c
13C{1H} NMR (101 MHz, DMSO-d6)
f1 (ppm)

OSPT1-AS-214_RP_T2_3.12.1.1r

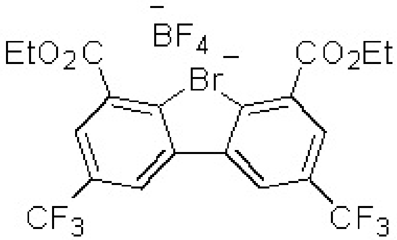

1c

^{19}F NMR (376 MHz, DMSO-d_6)

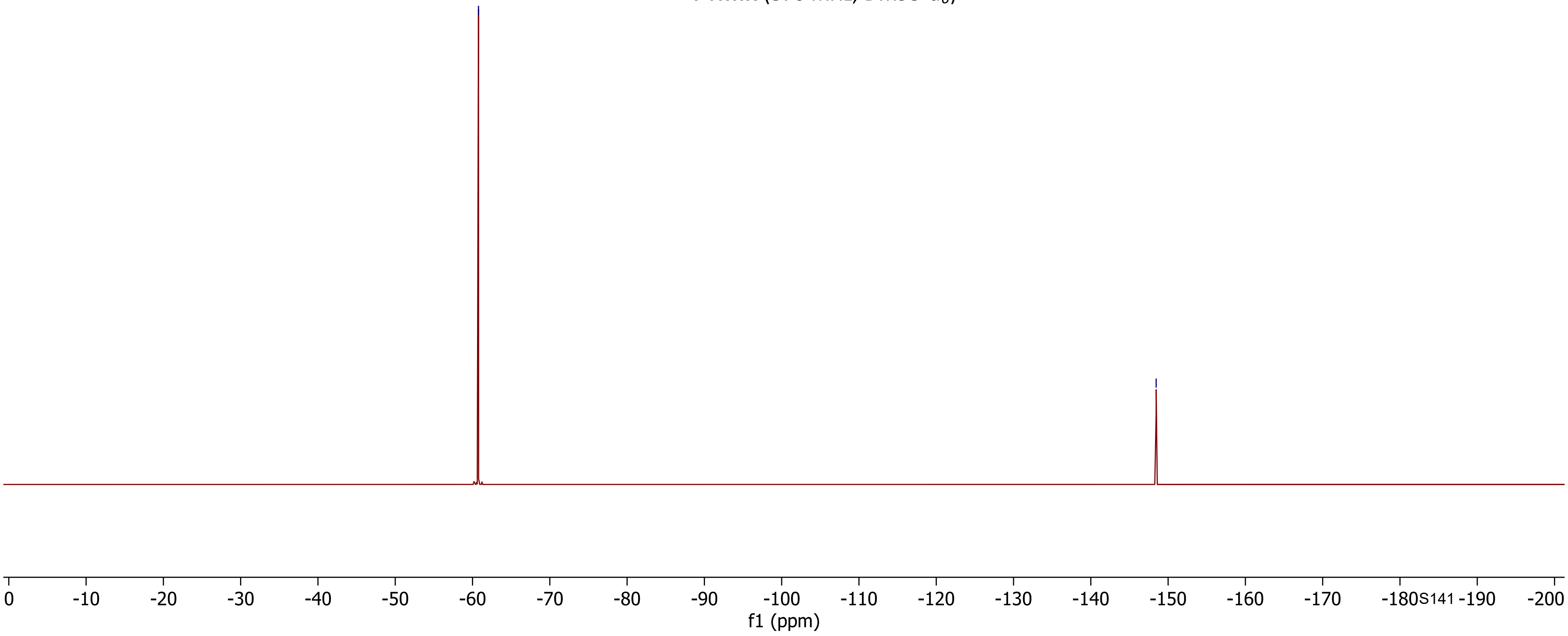

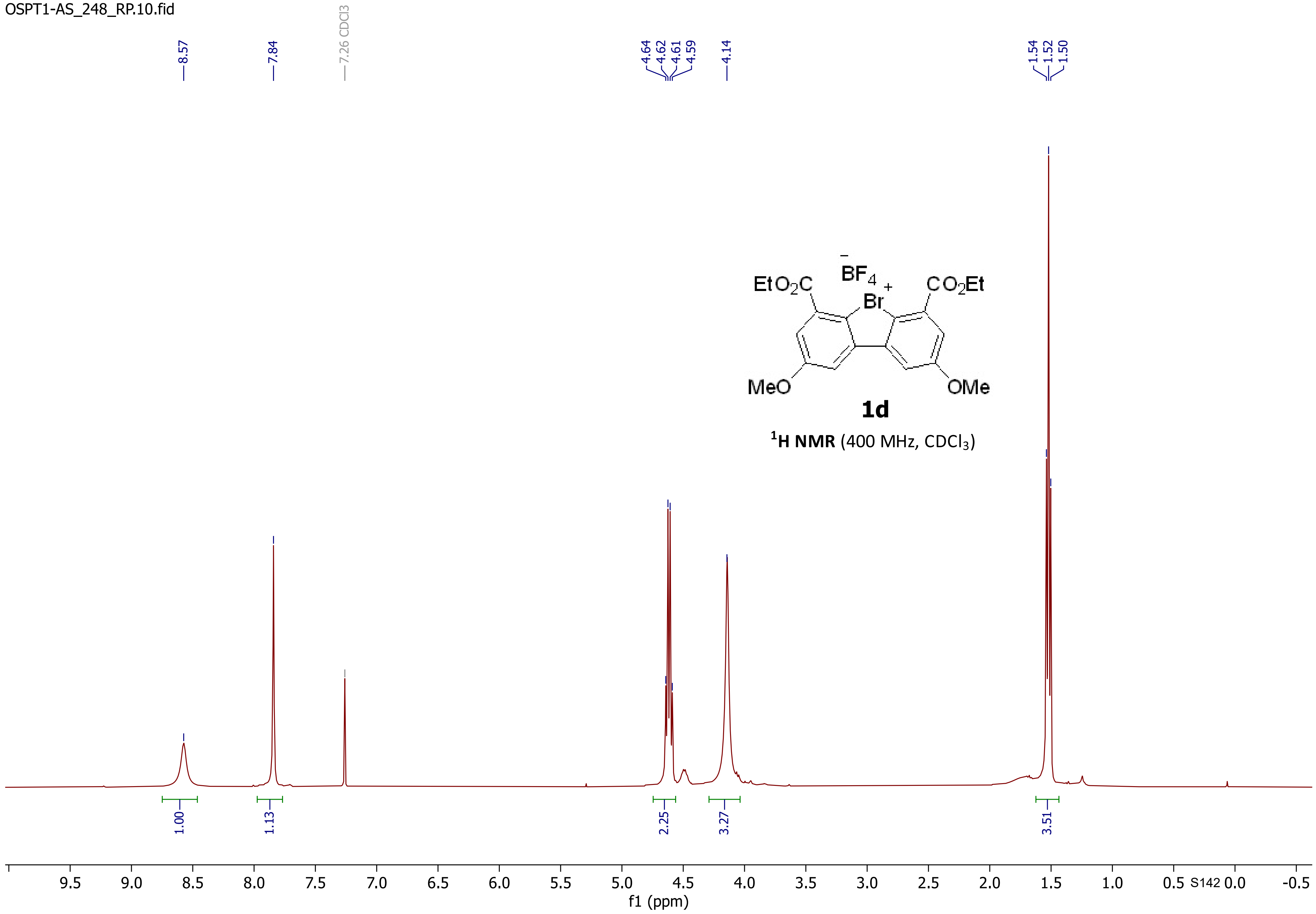
OSPT1-AS_248_RP.10.fid
8.57
7.84
7.26 CDCl3
4.64
4.62
4.61
4.59
4.14
1.54
1.52
1.50
EtO2C
BF4
Br
CO2Et
MeO
OMe
1d
1H NMR (400 MHz, CDCl3)
1.00
1.13
2.25
3.27
3.51
9.5 9.0 8.5 8.0 7.5 7.0 6.5 6.0 5.5 5.0 4.5 4.0 3.5 3.0 2.5 2.0 1.5 1.0 0.5 0.0 -0.5
f1 (ppm)

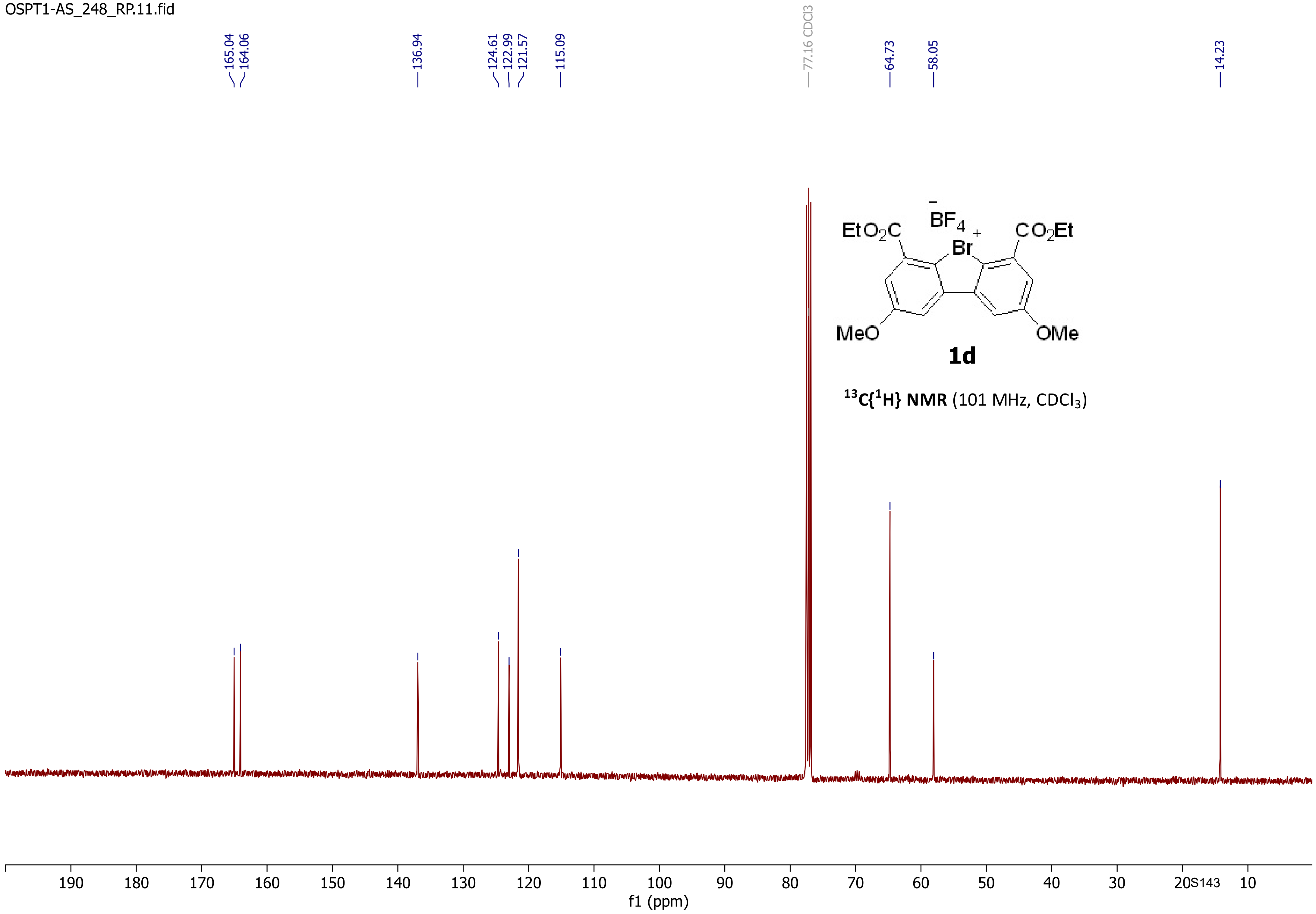
OSPT1-AS_248_RP.11.fid
165.04
164.06
136.94
124.61
122.99
121.57
115.09
77.16 CDCl3
64.73
58.05
14.23
BF4
Br
EtO2C
CO2Et
MeO
OMe
1d
13C{1H} NMR (101 MHz, CDCl3)
190 180 170 160 150 140 130 120 110 100 90 80 70 60 50 40 30 20 10
f1 (ppm)

OSPT1-AS-248_EXTR-6.11.1.1r

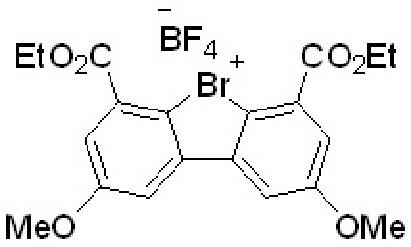

1d

^{19}F NMR (376 MHz, $CDCl_3$)

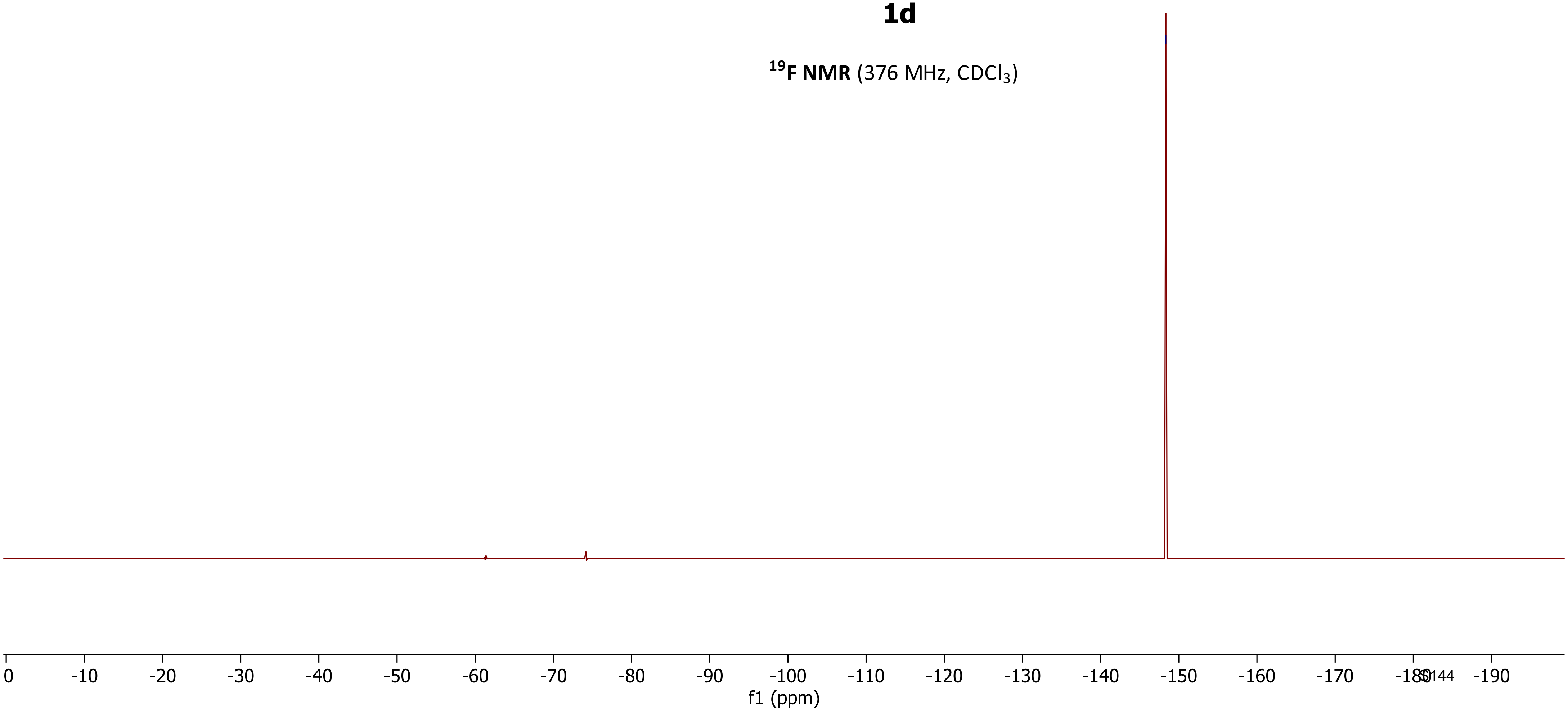

OSPT1-AS-251_EXTR-1.10.fid

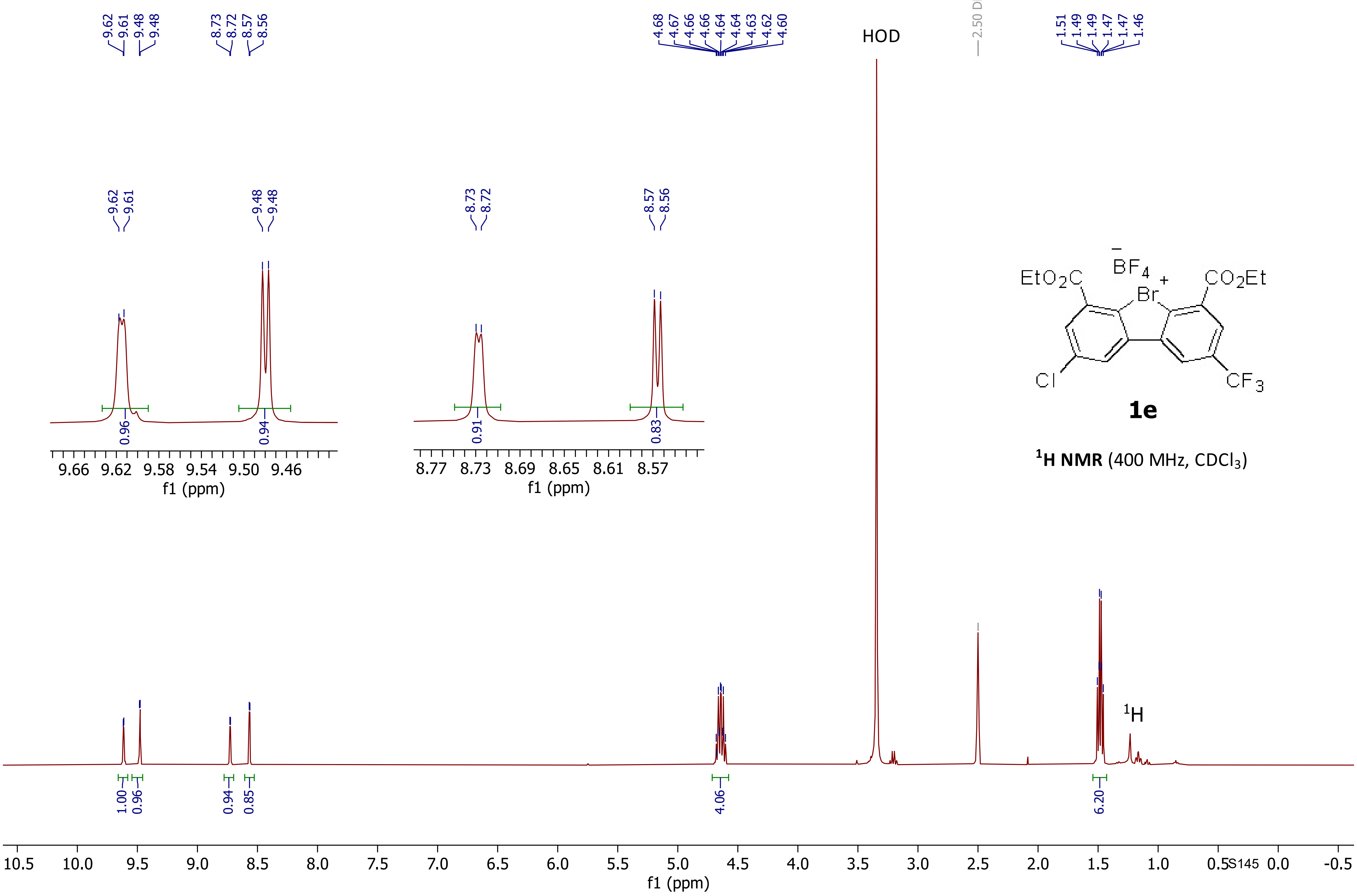

1e

^{1}H NMR (400 MHz, $CDCl_3$)

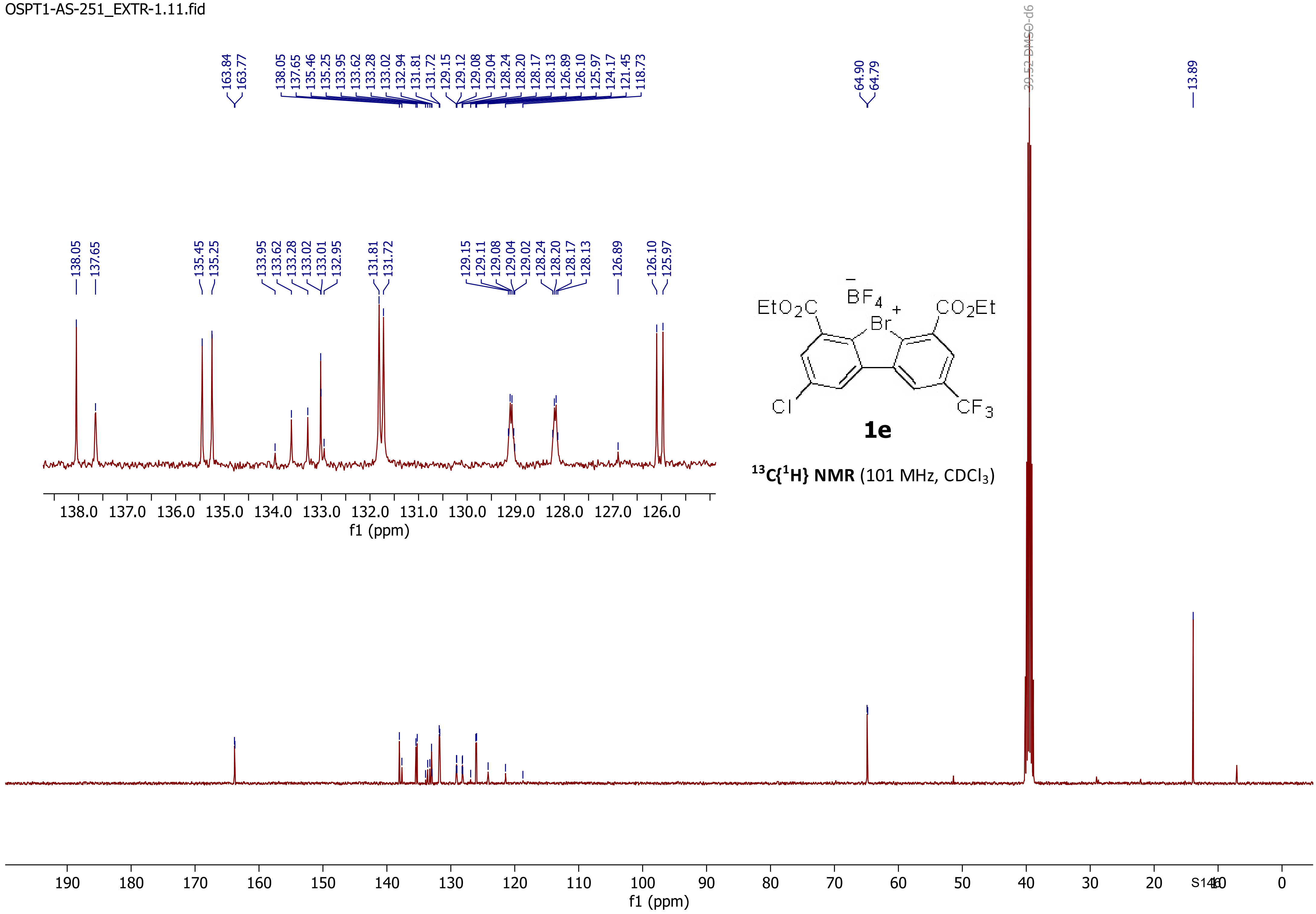

OSPT1-AS-251_EXTR-1.11.fid
163.84
163.77
138.05
137.65
135.46
135.25
133.95
133.62
133.28
133.02
132.94
131.81
131.72
129.15
129.12
129.08
129.04
128.24
128.20
128.17
128.13
126.89
126.10
125.97
124.17
121.45
118.73
64.90
64.79
39.52 DMSO-d6
13.89
138.05
137.65
135.45
135.25
133.95
133.62
133.28
133.02
133.01
132.95
131.81
131.72
129.15
129.11
129.08
129.04
129.02
128.24
128.20
128.17
128.13
126.89
126.10
125.97
f1 (ppm)
EtO2C
BF4
Br
CO2Et
Cl
CF3
1e
13C{1H} NMR (101 MHz, CDCl3)
f1 (ppm)

OSPT1-AS-251_EXTR-5.11.1.1r

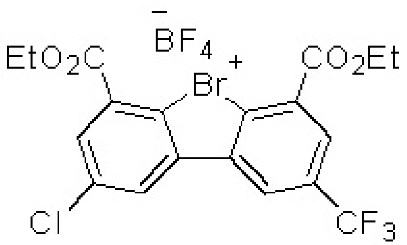

1e

^{19}F NMR (376 MHz, $CDCl_3$)

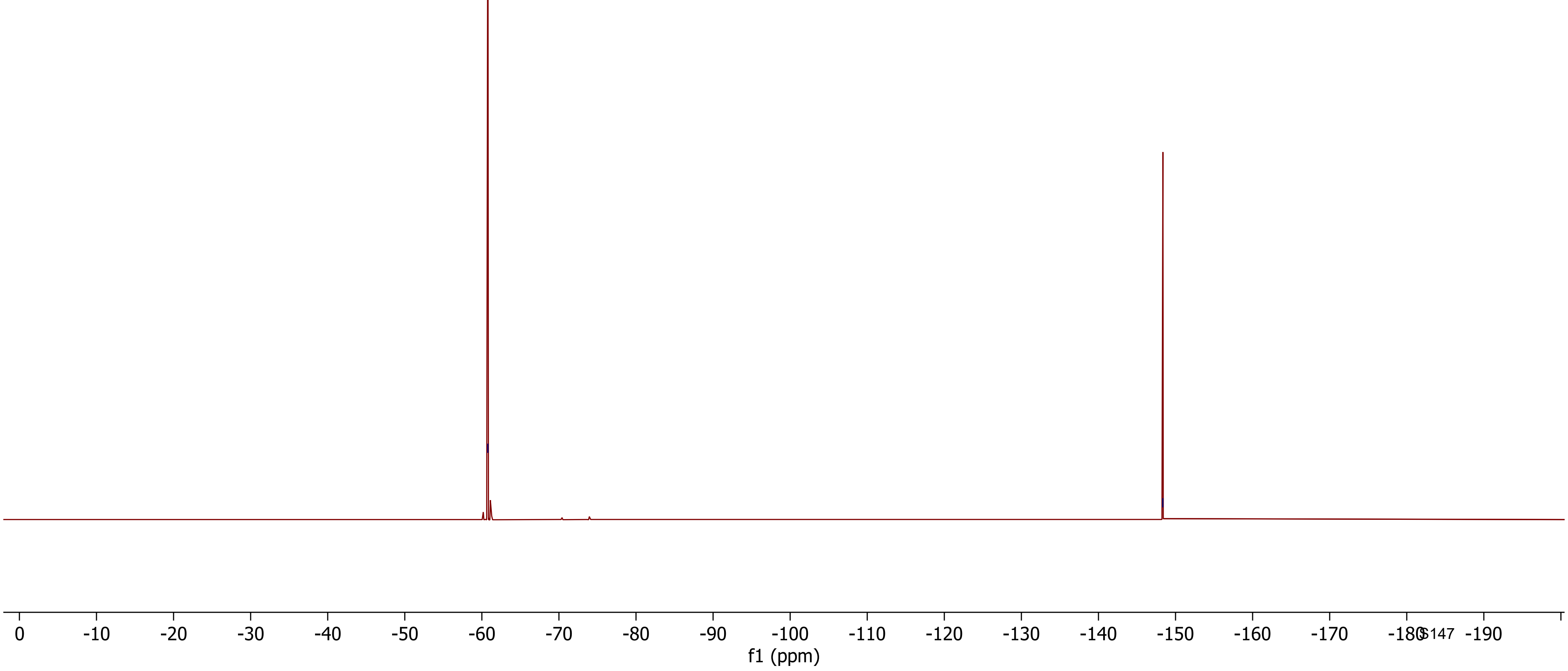

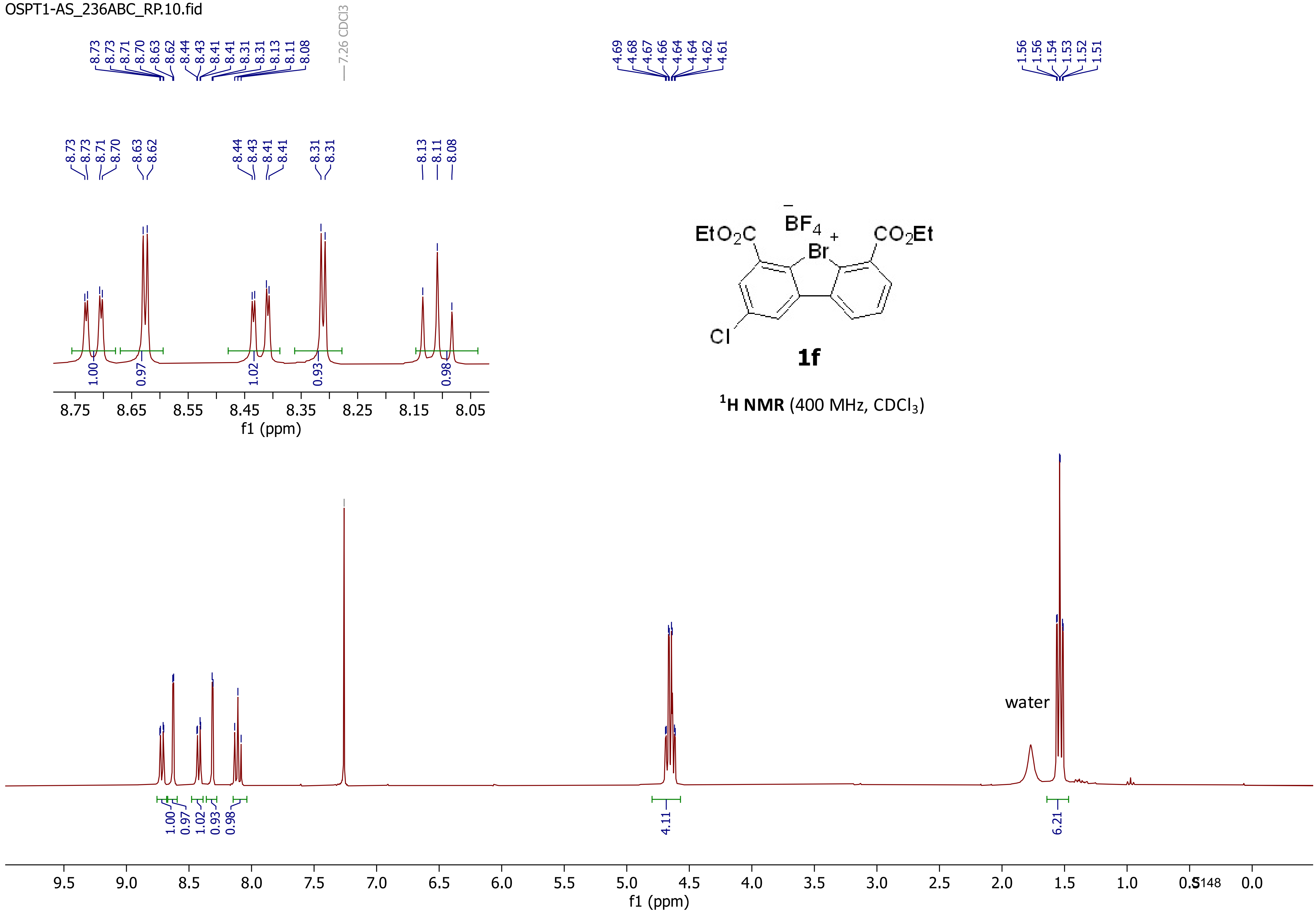
OSPT1-AS_236ABC_RP.10.fid
8.73 8.73 8.71 8.70 8.63 8.62 8.44 8.43 8.41 8.41 8.31 8.31 8.13 8.11 8.08
7.26 CDCl3
4.69 4.68 4.67 4.66 4.64 4.64 4.62 4.61
1.56 1.56 1.54 1.53 1.52 1.51
1.00 0.97 1.02 0.93 0.98
f1 (ppm)
EtO2C
BF4
Br
CO2Et
Cl
1f
1H NMR (400 MHz, CDCl3)
water
4.11
6.21
f1 (ppm)

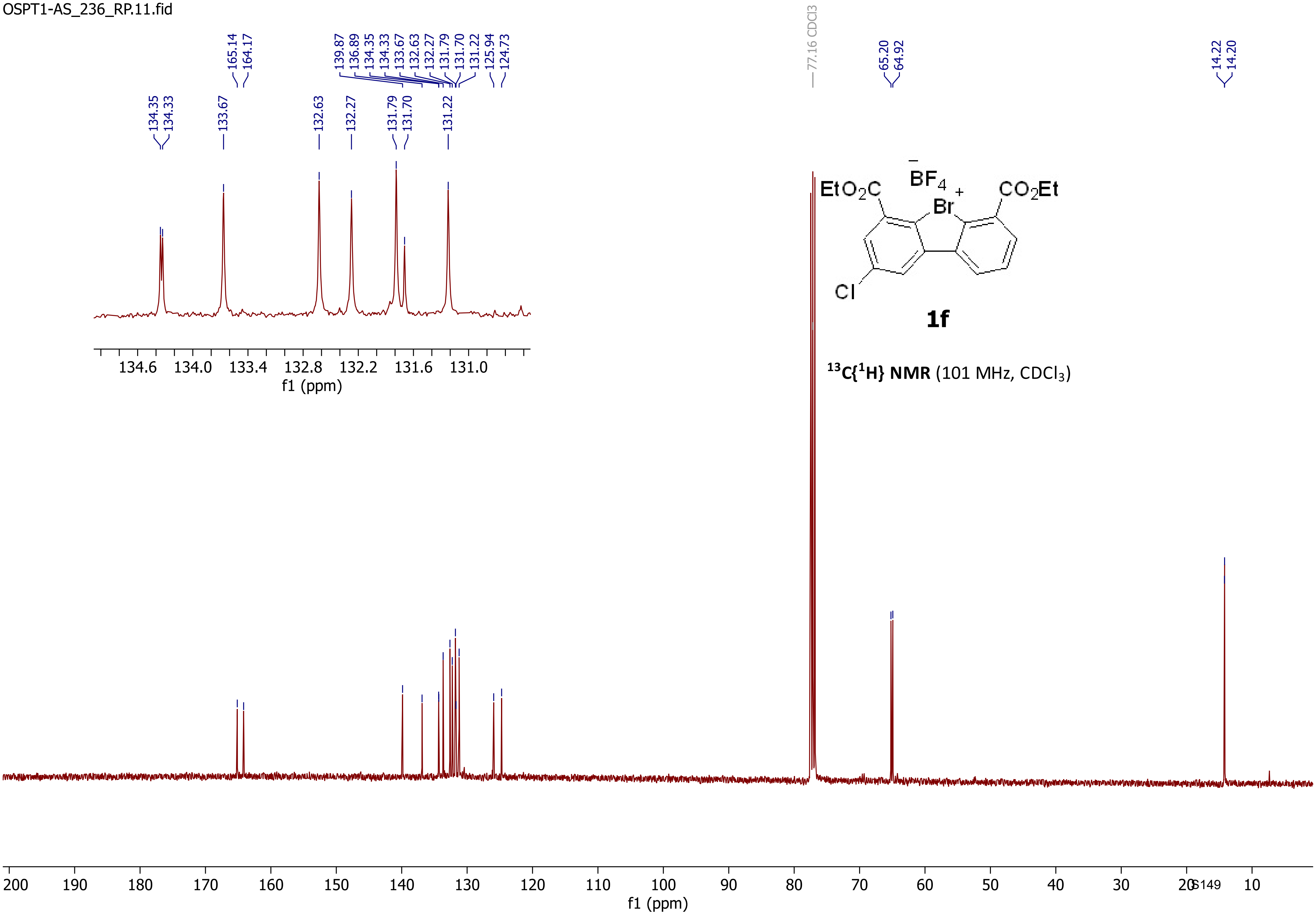
OSPT1-AS_236_RP.11.fid
165.14
164.17
139.87
136.89
134.35
134.33
133.67
132.63
132.27
131.79
131.70
131.22
125.94
124.73
77.16 CDCl3
65.20
64.92
14.22
14.20
1f
$^{13}C\{^1H\}$ NMR (101 MHz, $CDCl_3$)
f1 (ppm)

OSPT1-AS_236_EXTR.20.1.1r

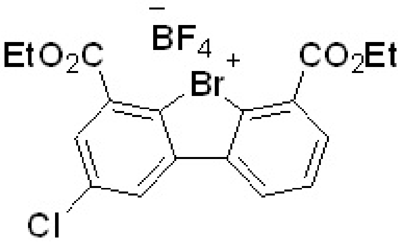

1f

^{19}F NMR (376 MHz, $CDCl_3$)

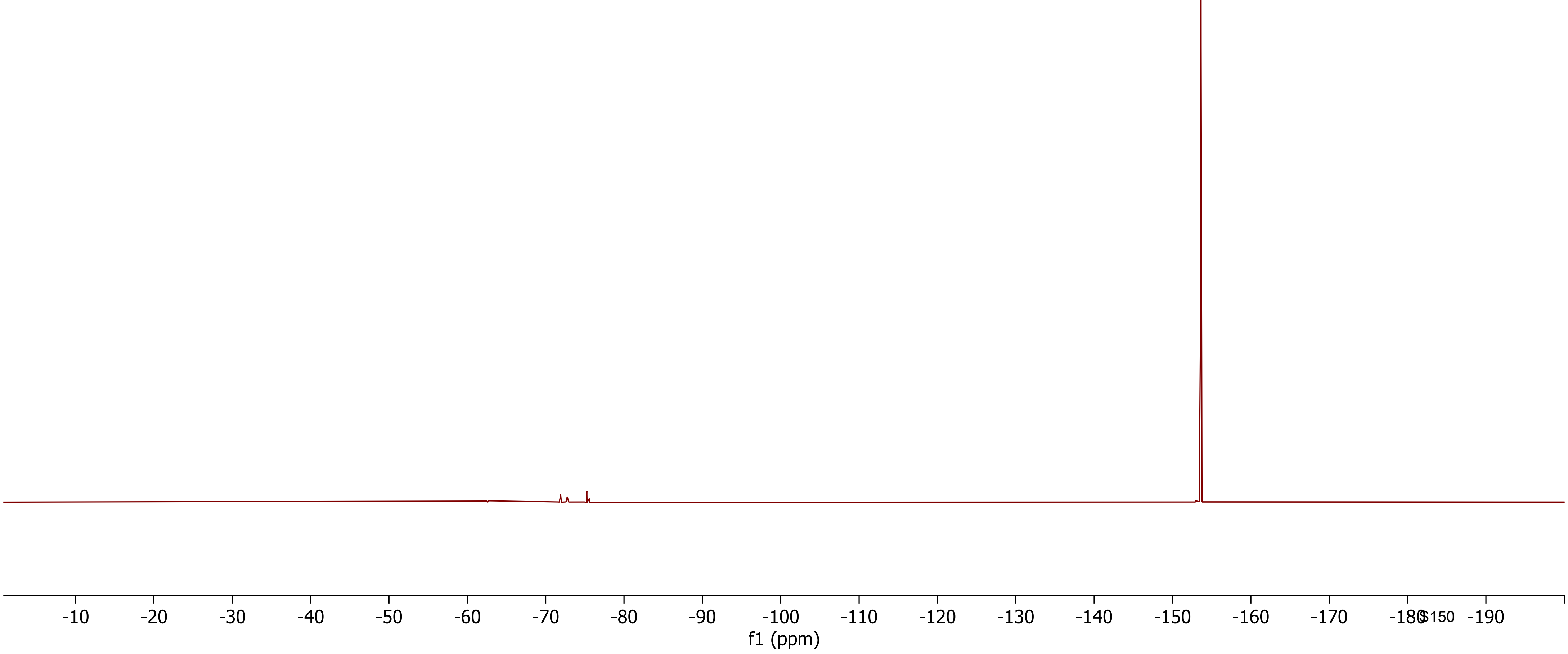

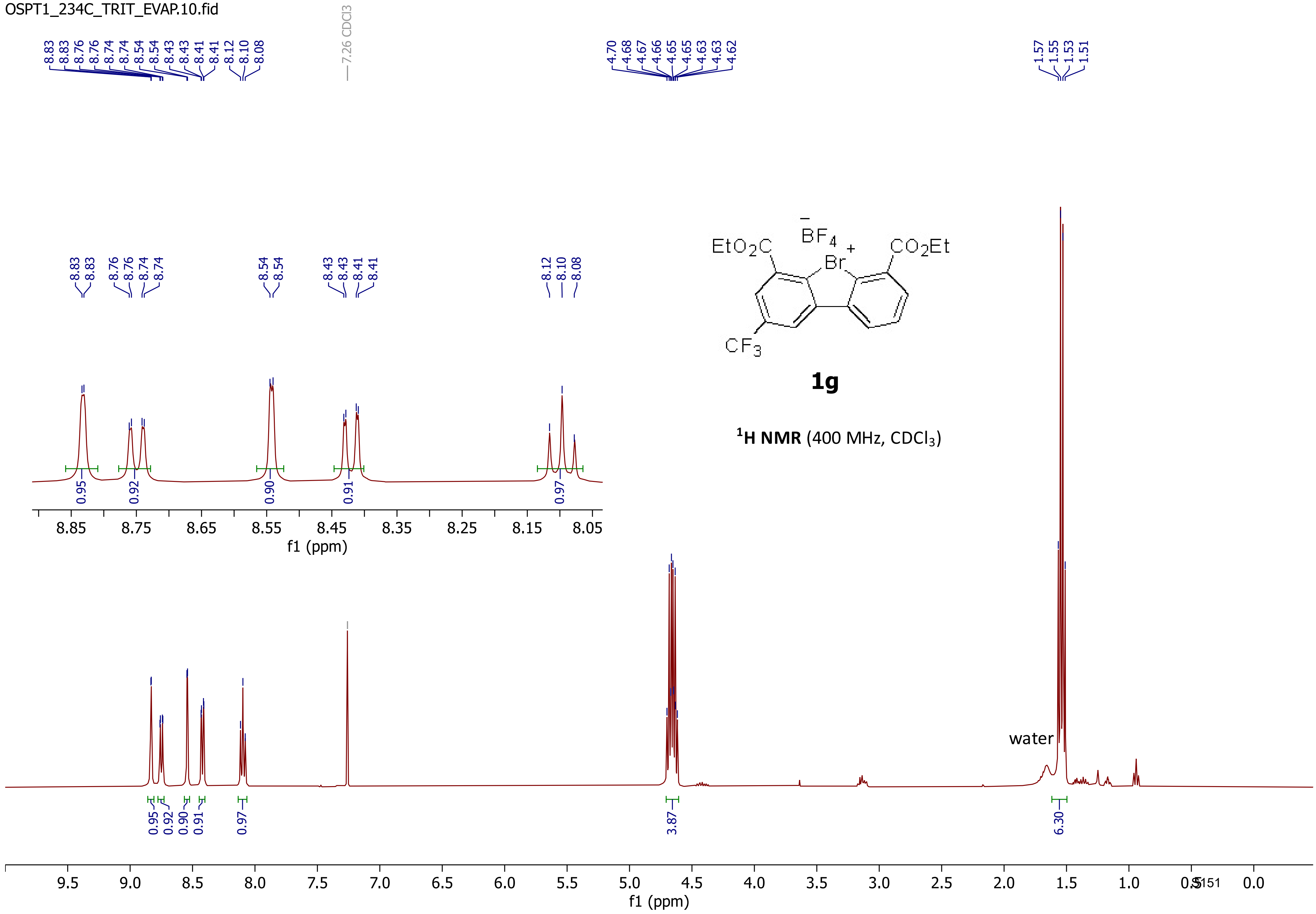
OSPT1_234C_TRIT_EVAP.10.fid
8.83 8.83 8.76 8.76 8.74 8.74 8.54 8.54 8.43 8.43 8.41 8.41 8.12 8.10 8.08
7.26 CDCl3
4.70 4.68 4.67 4.66 4.65 4.65 4.63 4.63 4.62
1.57 1.55 1.53 1.51
BF4
EtO2C
Br
CO2Et
CF3
1g
1H NMR (400 MHz, CDCl3)
water
0.95 0.92 0.90 0.91 0.97
3.87
6.30
f1 (ppm)

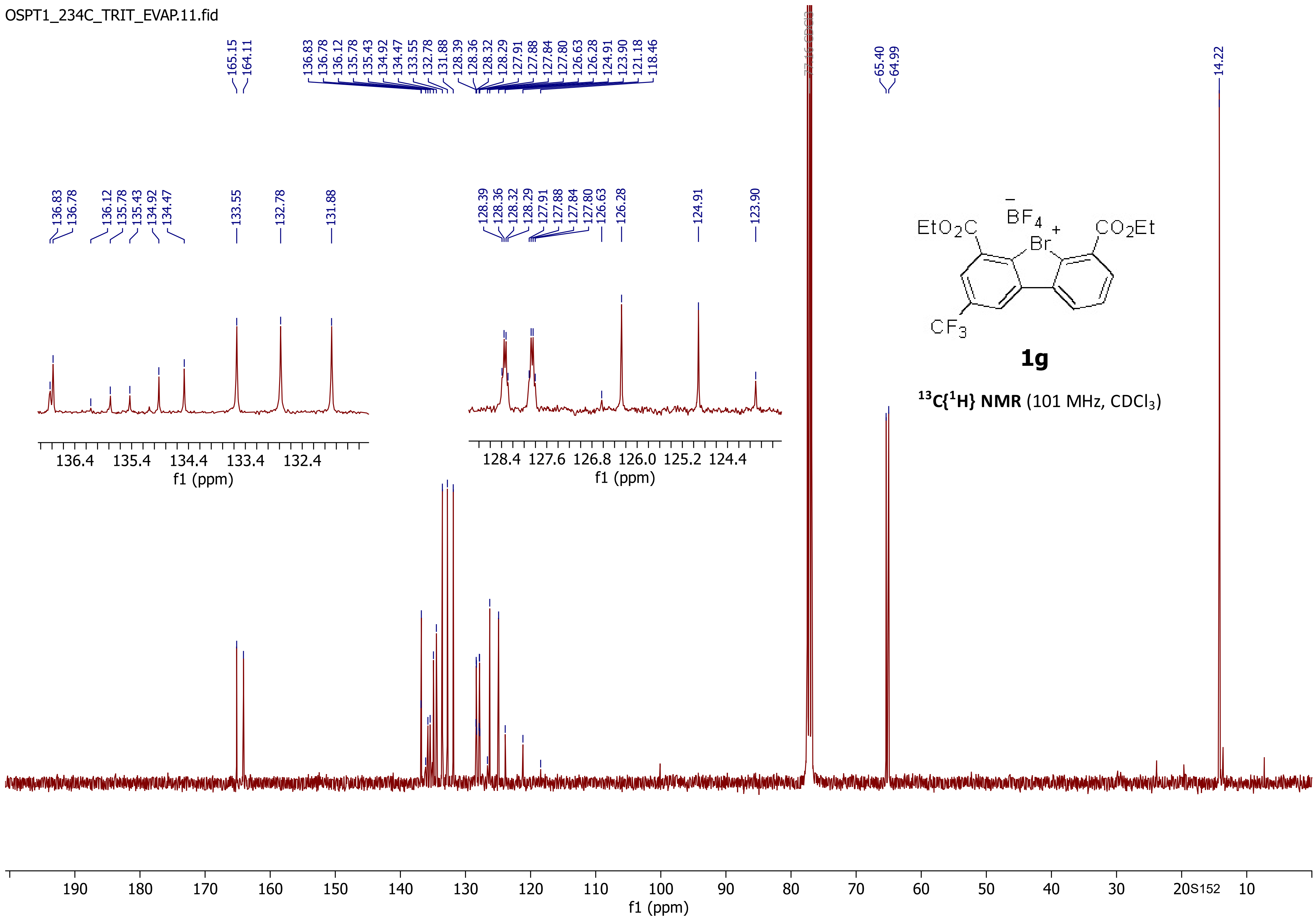
OSPT1_234C_TRIT_EVAP.11.fid
165.15
164.11
136.83
136.78
136.12
135.78
135.43
134.92
134.47
133.55
132.78
131.88
128.39
128.36
128.32
128.29
127.91
127.88
127.84
127.80
126.63
126.28
124.91
123.90
121.18
118.46
65.40
64.99
14.22
EtO2C
BF4
Br
CO2Et
CF3
1g
13C{1H} NMR (101 MHz, CDCl3)
136.4
135.4
134.4
133.4
132.4
128.4
127.6
126.8
126.0
125.2
124.4
f1 (ppm)
190
180
170
160
150
140
130
120
110
100
90
80
70
60
50
40
30
20
10
f1 (ppm)

OSPT1-AS-234B_EXTR-4.11.1.1r

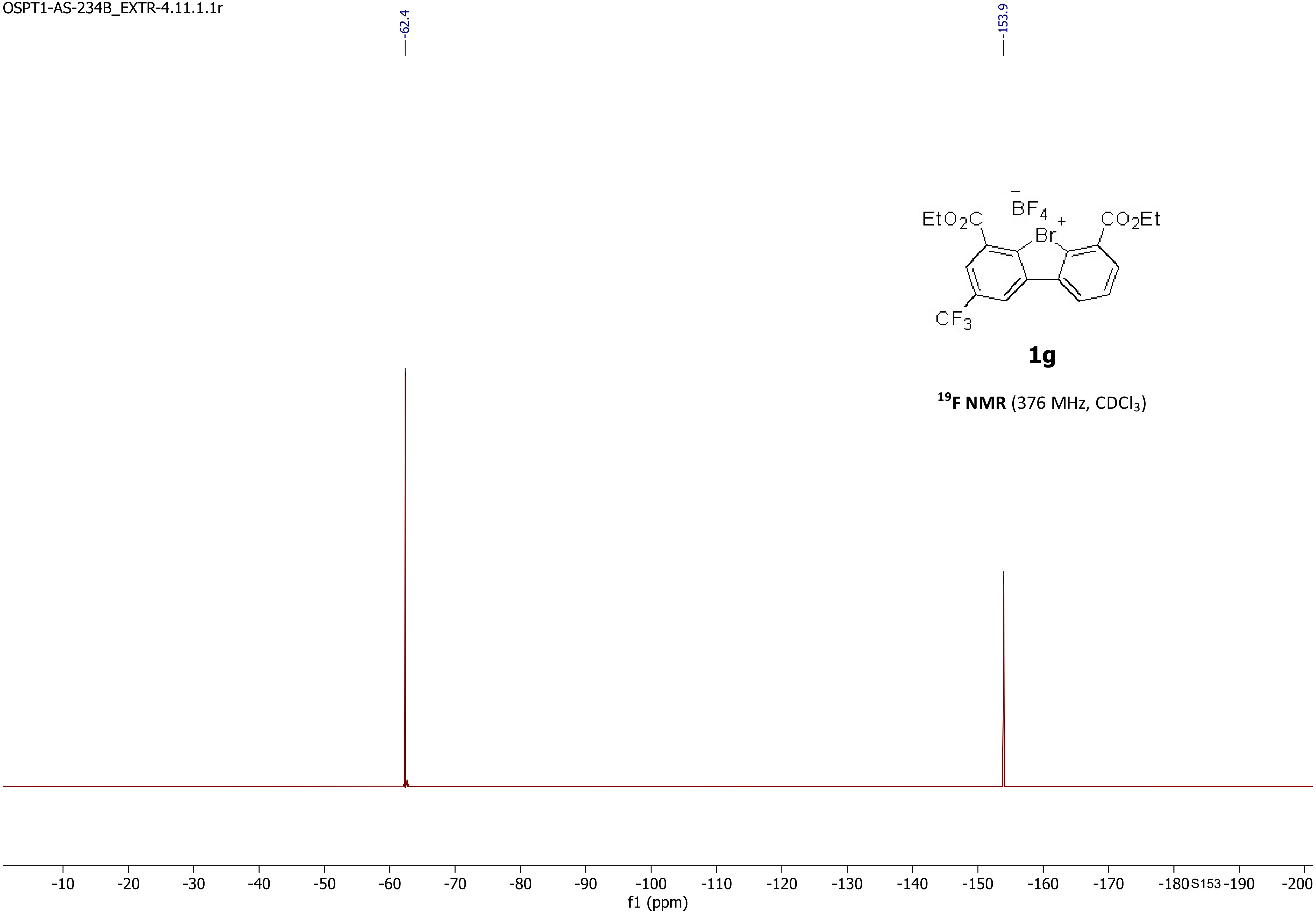

Supplement: File 1 — Experimental procedures, analytical and spectroscopic data for new compounds, copies of NMR spectra, and X-ray crystallographic data. [file Beilstein_J_Org_Chem-21-451-s001.pdf]
